# Global Patterns and Trends in Lifetime Risks of Developing and Dying from Cancer, 1990–2021

**DOI:** 10.34133/hds.0378

**Published:** 2026-09-10

**Authors:** Zhongge Ji, Yiming Song, Zeyu Li, Xiaojie Huang, Sijia Zhai, Qingran Liu, Ruitian Zeng, Ruijie Han, Yufei Xiao, Yuxin Yang, Xiaolu Lin, Qingwei Zhang, Xiaobo Li

**Affiliations:** ^1^ Division of Gastroenterology and Hepatology, Shanghai Institute of Digestive Disease, NHC Key Laboratory of Digestive Diseases, Renji Hospital, Shanghai Jiao Tong University School of Medicine, Shanghai, China.; ^2^ The First School of Medicine, School of Information and Engineering, Wenzhou Medical University, Wenzhou, China.; ^3^ Department of Digestive Endoscopy Center, Fujian Provincial Hospital, Fuzhou University Affiliated Provincial Hospital, Shengli Clinical Medical College of Fujian Medical University, Fuzhou, Fujian, China.; ^4^ School of Basic Medical Sciences, Fujian Medical University, Fuzhou, China.

## Abstract

**Background:** Cancer remains a major contributor to global disease burden, necessitating comprehensive understanding of cancer risk at global, regional, and national scales to inform cancer control planning and resource allocation. **Methods:** The Adjusted for Multiple Primaries analytical approach was employed to calculate lifetime cancer risks from 1990 to 2021, based on data from the Global Burden of Disease Study 2021. Joinpoint regression analysis was utilized to assess trends in lifetime cancer risks over this period. **Results:** Globally, the lifetime risks of developing and dying from cancer were 21.70% (95% confidence interval [CI], 21.63 to 21.78) and 14.23% (95% CI, 14.19 to 14.26), respectively, in 2021. Men had higher risks (22.15%, 95% CI: 22.07 to 22.23 for incidence and 15.19%, 95% CI: 15.16 to 15.23 for death) compared to women (21.03%, 95% CI: 20.88 to 21.19 for incidence and 13.06%, 95% CI: 13.00 to 13.12 for death). Both risks showed strong positive correlations with the sociodemographic index (*ρ* = 0.89 and 0.82, *P* < 0.001). Moreover, the lifetime incidence risk increased by 14.00% between 1990 (19.04%, 95% CI: 18.97 to 19.12) and 2021, while the death risk decreased by 1.86% between 1990 (14.50%, 95% CI: 14.48 to 14.52) and 2021. During the COVID-19 pandemic, the incidence risk decreased with an average annual percentage change of −4.73% (95% CI, −5.18 to −4.40) and death risk decreased with an average annual percentage change of −6.24% (95% CI, −6.74 to −5.92). The decline was most pronounced in low-sociodemographic-index regions. **Conclusions:** The global cancer burden is substantial and increasing, with marked geographic and gender disparities. These findings provide comprehensive estimates to guide targeted cancer control strategies and health system strengthening.

## Introduction

Cancer is increasingly recognized as a global challenge, yet it remains without a universal solution. Based on the estimates from the World Health Organization in 2022, around 20 million new cancer cases were diagnosed worldwide, resulting in approximately 9.7 million deaths. Projections indicate that the count of new cases will exceed 35 million by 2040, reflecting a 77% increase from the 2022 estimates [1]. This highlights the substantial and growing global cancer burden. Given its considerable health impact, it is crucial to develop population-level indicators that reflect the individual likelihood of developing and dying from cancer [2].

Lifetime risk offers a more intuitive and longitudinal perspective on cancer burden than traditional incidence and mortality rates [2]. By quantifying the cumulative probability of cancer incidence or mortality, lifetime risk reflects changing demographics and competing causes of death [3,4]. This metric plays a critical role in global cancer control planning and facilitates risk communication [5,6].

Although previous studies have used lifetime risk to assess the likelihood of being diagnosed with cancer during an individual’s lifetime, several critical gaps remain [3]. First, most prior estimates focus exclusively on cancer incidence, with limited quantification of the lifetime probability of dying from cancer—a metric equally essential for mortality reduction planning. Second, existing analyses largely rely on single-time-point data, precluding assessment of how lifetime risks have evolved over recent decades amid rapid demographic transitions, expanding screening programs, and shifting risk factor profiles. Third, no study to date has simultaneously examined lifetime incidence and death risks across the full sociodemographic index (SDI) spectrum at national, regional, and global levels using a unified methodological framework that accounts for competing causes of death and multiple primary cancers. Finally, the profound disruption of the COVID-19 pandemic on global healthcare systems warrants dedicated epidemiological evaluation of its impact on lifetime cancer risk trajectories—an analysis entirely absent from existing literature [7].

This study analyzed data from the Global Burden of Disease (GBD) Study 2021 to examine changes in the lifetime risks of developing (i.e., lifetime incidence risk) and dying from (i.e., lifetime death risk) cancer stratified by sex, geographic regions, and SDI levels between 1990 and 2021. We also investigated the trends separately for the period from 2019 to 2021 to evaluate the impact of the COVID-19 pandemic on these risks.

## Methods

### Data sources and process

Data on the incidence and mortality related to total cancers (excluding non-melanoma skin cancer) and 32 major cancer groups, all-cause mortality, and population statistics were extracted from GBD 2021 [8,9]. These data were stratified by age group (0–4, 5–9, …, 85–89, 90–94, and 95+ y) and sex, at national, regional, and global levels. Mortality estimates were derived employing the Cause of Death Ensemble Model, which combines data from civil registration and verbal autopsies [10]. Cancer incidence was estimated by combining mortality rates with the mortality-to-incidence ratio.

The cancers analyzed in the study were malignant neoplasms, as defined by the International Classification of Diseases, Tenth Revision (ICD-10), codes C00 to C96 [11]. Kaposi sarcoma was excluded from this analysis, as deaths were primarily redistributed to HIV/AIDS [12]. The 32 GBD cancer causes and their corresponding ICD-10 codes are provided in Table S1. In this study, the term “cancer” refers to total cancers, excluding non-melanoma skin cancer (NMSC), which was excluded due to its high prevalence, nonfatal nature, and incomplete registration, particularly among the elderly [13].

Additionally, countries were stratified into quintiles based on their SDI scores. The SDI quintile thresholds followed the classification framework established by the GBD study, categorizing locations into 5 groups (low, low-middle, middle, high-middle, and high SDI) according to a composite index incorporating income per capita, mean education, and total fertility rate. Spearman rank correlation coefficients (*ρ*) were used to assess the association between SDI and lifetime cancer risks across countries and territories, with corresponding *P* values derived from 2-tailed Spearman rank correlation tests. SDI serves as a composite metric of social and economic development, which facilitates the comprehensive analysis of disease burden patterns across diverse resource settings [14].

### Lifetime risk estimation

Lifetime risk was quantified utilizing the adjusted for multiple primaries (AMP) methodology, which accounts for competing causes of death and multiple primary cancers [4,15]. Cancer incidence, mortality, and all-cause mortality rates for each 5-year age group were used to estimate the lifetime risk of developing or dying from cancer at a given age. This method reflects the likelihood of a person being diagnosed or dying from cancer from that age afterward [16]. Detailed methodology is provided in Appendix S1.

Estimates were made for total cancer (excluding NMSC) at national, regional, and global levels, stratified by SDI quintile, cancer type, and sex, and within specific age intervals. The 95% confidence intervals (CIs) were calculated based on the assumption of a Poisson distribution for cancer incidence and mortality, using the corresponding variance. In the attributable risk analysis, the proportion of deaths attributed to each risk factor from GBD 2021 data was multiplied by lifetime death risks to derive the absolute attributable risk per risk factor, with confidence intervals estimated via the delta method [17]. To validate the robustness of our findings, sensitivity analyses were conducted by comparing lifetime cancer risks against cumulative risks of cancers in the 0-to-74 age group [15]. This conventional metric serves as a practical surrogate for lifetime risk, though it does not account for competing mortality from non-cancer causes, the occurrence of multiple primary tumors, or variations in life expectancy across populations.

Analyses were conducted using R version 4.4.1.

### Trend estimation

Temporal trends in lifetime risk were assessed through joinpoint regression to calculate average annual percentage changes (AAPCs) [18]. AAPCs were calculated as the weighted mean of the slope coefficients from the joinpoint regression model for 1990–2021. Regarding the determination of joinpoints, the number of joinpoints was not manually specified but was objectively determined by the Joinpoint Regression Program using a permutation-test-based model selection procedure, with a prespecified maximum of 5 joinpoints. The AAPC value indicates the direction and magnitude of change: Positive values (with corresponding 95% CIs > 0) suggest an upward trend, while negative values (with CIs < 0) indicate a downward trend [19]. Analyses were conducted using the Joinpoint Regression Program, version 5.0.2.

## Results

### Global, regional, and national lifetime cancer risks in 2021

**Fig. 1.**
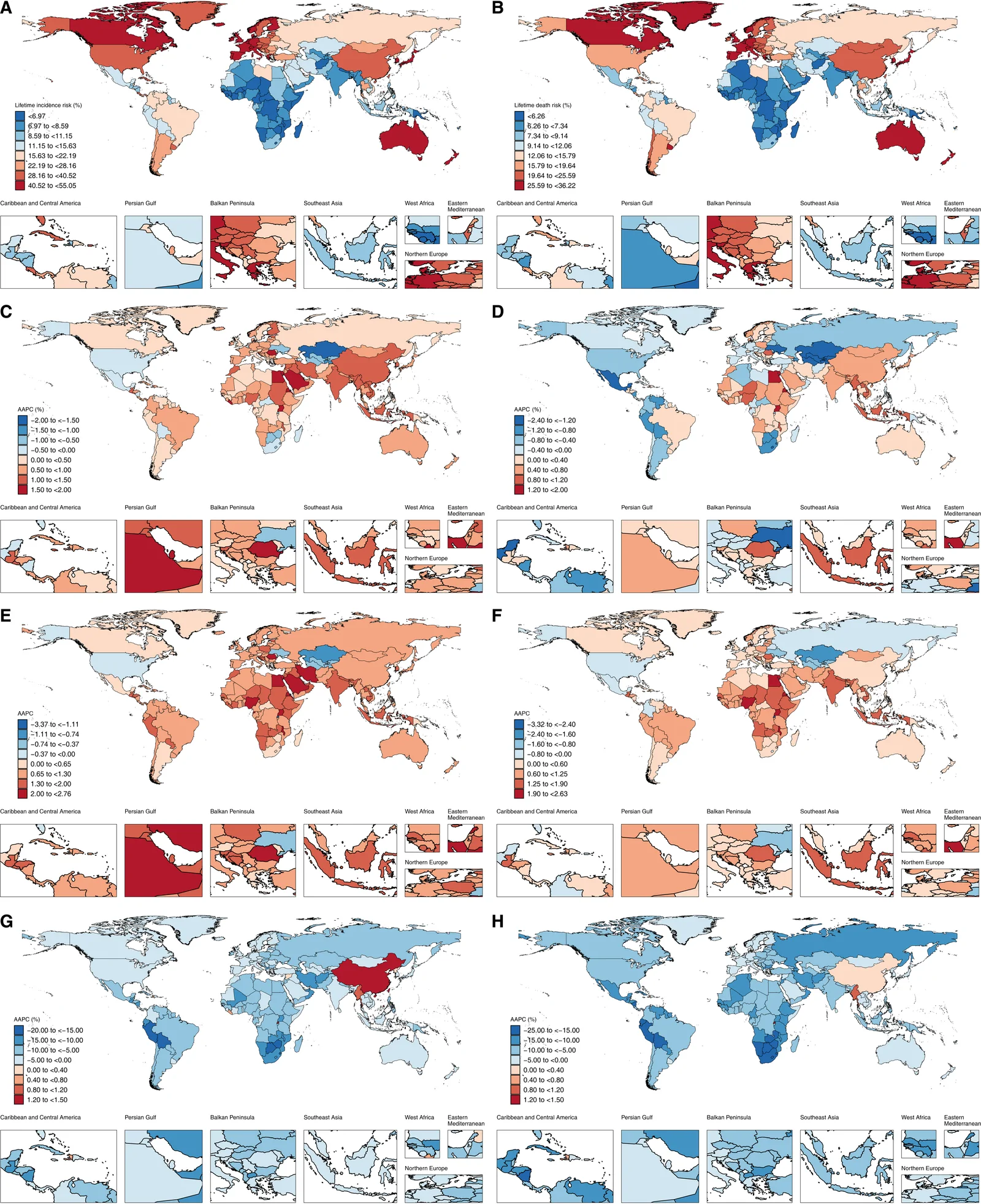
Lifetime risks and temporal trends of developing and dying from cancer at the national level for both sexes combined. (A) Lifetime incidence risks in 2021. (B) Lifetime death risks in 2021. (C) Average annual percentage changes (AAPCs) in lifetime incidence risk, 1990–2021. (D) AAPCs in lifetime death risk, 1990–2021. (E) AAPCs in lifetime incidence risk, 1990–2019. (F) AAPCs in lifetime death risk, 1990–2019. (G) AAPCs in lifetime incidence risk, 2019–2021. (H) AAPCs in lifetime death risk, 2019–2021.

In 2021, the global lifetime risk of developing cancer was estimated at 21.70% (95% CI, 21.63 to 21.78), while the lifetime risk of dying from cancer was 14.23% (95% CI, 14.19 to 14.26) (Table 1). The lifetime risks of both developing (*ρ* = 0.89, *P* < 0.001) and dying from (*ρ* = 0.82, *P* < 0.001) cancer showed a positive correlation with the SDI (Figs. S5 and S6). High-SDI countries exhibited the highest lifetime risks: 39.36% (95% CI, 38.76 to 39.97) for developing cancer and 22.73% (95% CI, 22.53 to 22.94) for dying from cancer. Conversely, low-SDI countries had the lowest lifetime risks: 7.95% (95% CI, 7.90 to 8.00) for developing cancer and 6.80% (95% CI, 6.76 to 6.83) for dying from cancer. Within the 21 GBD regions analyzed, Australasia had the highest lifetime risk of developing cancer (46.64%, 95% CI: 41.34 to 51.59), followed by High-income Asia Pacific (41.97%, 95% CI: 39.79 to 44.16) and Western Europe (41.45%, 95% CI: 40.05 to 42.85). Central Sub-Saharan Africa exhibited the lowest lifetime probability of cancer development (7.71%, 95% CI: 7.61 to 7.82), followed by Western Sub-Saharan Africa (7.99%, 95% CI: 7.92 to 8.06) and Oceania (8.52%, 95% CI: 8.05 to 8.99). High-income Asia Pacific also had the highest lifetime risk of dying from cancer (26.91%, 95% CI: 26.06 to 27.77), followed by Australasia (25.31%, 95% CI: 23.26 to 27.37) and Western Europe (24.30%, 95% CI: 23.81 to 24.80) (Table 1; Tables S5 and S6). At the national level, Monaco exhibited the highest estimated lifetime risk of developing cancer (53.43%, 95% CI: −128.25 to 235.11), followed by New Zealand and Australia. The highest estimated lifetime risk of dying from cancer was also reported in Monaco (33.79%, 95% CI: −38.25 to 105.84), with comparably high risks in Denmark and Japan (Fig. 1; Tables S7 and S8).

**Table 1.** Global, sociodemographic index (SDI)-stratified and national lifetime risks of developing and dying from cancer in 2021 and average annual percent changes (AAPCs) in lifetime risks from 1990 to 2021 by sex

Location	Male				Female				Both sexes			
	Lifetime incidence risk	AAPC	Lifetime death risk	AAPC	Lifetime incidence risk	AAPC	Lifetime death risk	AAPC	Lifetime incidence risk	AAPC	Lifetime death risk	AAPC
Global	22.15 (22.07 to 22.23)	0.32 (0.28 to 0.34)	15.19 (15.16 to 15.23)	−0.20 (−0.25 to −0.16)	21.03 (20.88 to 21.19)	0.46 (0.44 to 0.48)	13.06 (13.00 to 13.12)	0.00 (−0.02 to 0.02)	21.70 (21.63 to 21.78)	0.41 (0.38 to 0.44)	14.23 (14.19 to 14.26)	−0.08 (−0.12 to −0.06)
SDI quintiles												
High SDI	41.50 (40.82 to 42.18)	0.24 (0.18 to 0.29)	25.03 (24.78 to 25.27)	−0.20 (−0.28 to −0.12)	37.14 (36.00 to 38.28)	0.33 (0.29 to 0.36)	20.56 (20.17 to 20.95)	−0.02 (−0.07 to 0.03)	39.36 (38.76 to 39.97)	0.31 (0.27 to 0.35)	22.73 (22.53 to 22.94)	−0.11 (−0.19 to −0.04)
High-middle SDI	30.18 (29.75 to 30.60)	0.55 (0.53 to 0.58)	21.33 (21.14 to 21.52)	−0.10 (−0.13 to −0.08)	25.20 (24.44 to 25.97)	0.70 (0.68 to 0.71)	15.17 (14.89 to 15.45)	0.00 (−0.03 to 0.01)	27.73 (27.35 to 28.12)	0.64 (0.62 to 0.67)	18.29 (18.14 to 18.44)	−0.04 (−0.07 to −0.02)
Middle SDI	18.73 (18.60 to 18.87)	0.71 (0.69 to 0.73)	14.48 (14.40 to 14.55)	0.03 (0.00 to 0.04)	17.09 (16.80 to 17.38)	0.79 (0.76 to 0.80)	11.66 (11.54 to 11.77)	0.01 (−0.01 to 0.02)	18.07 (17.94 to 18.21)	0.77 (0.76 to 0.78)	13.24 (13.18 to 13.30)	0.04 (0.03 to 0.06)
Low-middle SDI	8.60 (8.55 to 8.64)	0.79 (0.74 to 0.81)	7.40 (7.37 to 7.43)	0.41 (0.35 to 0.44)	10.68 (10.54 to 10.83)	1.11 (1.08 to 1.13)	8.03 (7.96 to 8.10)	0.71 (0.67 to 0.73)	9.66 (9.60 to 9.72)	0.97 (0.94 to 1.00)	7.75 (7.71 to 7.78)	0.57 (0.53 to 0.60)
Low SDI	6.47 (6.44 to 6.51)	0.40 (0.22 to 0.55)	5.93 (5.90 to 5.95)	0.17 (−0.01 to 0.33)	9.48 (9.35 to 9.62)	0.80 (0.75 to 0.84)	7.70 (7.62 to 7.77)	0.57 (0.52 to 0.62)	7.95 (7.90 to 8.00)	0.63 (0.52 to 0.72)	6.80 (6.76 to 6.83)	0.39 (0.27 to 0.48)
GBD regions												
Andean Latin America	14.31 (13.70 to 14.92)	−0.21 (−0.55 to 0.08)	10.03 (9.75 to 10.31)	−1.43 (−1.84 to −1.07)	19.96 (18.13 to 21.79)	0.46 (0.33 to 0.59)	13.98 (13.19 to 14.78)	−0.50 (−0.66 to −0.38)	17.14 (16.40 to 17.87)	0.16 (−0.05 to 0.34)	11.95 (11.64 to 12.27)	−0.94 (−1.22 to −0.73)
Australasia	50.87 (44.39 to 57.36)	0.62 (0.56 to 0.70)	28.93 (26.42 to 31.43)	0.25 (0.22 to 0.29)	41.89 (33.10 to 50.67)	0.53 (0.49 to 0.58)	21.83 (18.11 to 25.54)	0.08 (0.02 to 0.13)	46.64 (41.34 to 51.95)	0.58 (0.53 to 0.63)	25.31 (23.26 to 27.37)	0.18 (0.15 to 0.22)
Caribbean	21.77 (21.02 to 22.53)	0.27 (−0.47 to 1.07)	13.93 (13.56 to 14.30)	−0.42 (−1.23 to 0.07)	21.55 (19.82 to 23.28)	0.78 (0.33 to 1.21)	13.27 (12.54 to 14.00)	0.42 (−0.04 to 0.88)	21.95 (21.14 to 22.76)	0.41 (−0.59 to 1.76)	13.79 (13.44 to 14.15)	−0.17 (−1.24 to 1.35)
Central Asia	11.86 (11.43 to 12.28)	−1.19 (−1.30 to −1.10)	10.15 (9.87 to 10.42)	−1.53 (−1.65 to −1.43)	12.91 (11.70 to 14.11)	−0.56 (−0.63 to −0.5)	9.12 (8.57 to 9.67)	−1.00 (−1.06 to −0.94)	12.39 (11.91 to 12.87)	−0.85 (−0.93 to −0.77)	9.65 (9.40 to 9.90)	−1.25 (−1.33 to −1.18)
Central Europe	29.40 (28.07 to 30.73)	0.80 (0.77 to 0.83)	21.14 (20.62 to 21.67)	0.16 (0.14 to 0.19)	27.48 (24.79 to 30.17)	0.87 (0.83 to 0.89)	17.57 (16.54 to 18.61)	0.35 (0.31 to 0.38)	28.49 (27.22 to 29.76)	0.83 (0.81 to 0.86)	19.41 (18.94 to 19.88)	0.26 (0.22 to 0.29)
Central Latin America	16.08 (15.77 to 16.39)	0.20 (−0.05 to 0.35)	9.83 (9.69 to 9.96)	−1.04 (−1.34 to −0.85)	18.45 (17.50 to 19.40)	0.11 (0.02 to 0.19)	11.53 (11.18 to 11.87)	−0.88 (−1.01 to −0.79)	17.48 (17.11 to 17.85)	0.18 (0.04 to 0.29)	10.75 (10.61 to 10.89)	−0.91 (−1.11 to −0.77)
Central Sub-Saharan Africa	6.12 (6.05 to 6.19)	0.20 (0.08 to 0.28)	5.72 (5.66 to 5.78)	−0.02 (−0.12 to 0.06)	9.36 (9.05 to 9.67)	0.75 (0.72 to 0.77)	7.52 (7.34 to 7.69)	0.45 (0.42 to 0.47)	7.71 (7.61 to 7.82)	0.46 (0.38 to 0.52)	6.61 (6.54 to 6.68)	0.19 (0.13 to 0.24)
East Asia	30.55 (30.06 to 31.04)	1.23 (1.21 to 1.25)	23.37 (23.10 to 23.64)	0.48 (0.46 to 0.50)	23.46 (22.62 to 24.30)	1.25 (1.22 to 1.27)	15.64 (15.28 to 16.01)	0.35 (0.33 to 0.36)	27.32 (26.89 to 27.75)	1.26 (1.24 to 1.28)	19.83 (19.62 to 20.04)	0.44 (0.43 to 0.46)
Eastern Europe	21.52 (21.06 to 21.97)	−0.31 (−0.41 to −0.21)	13.74 (13.56 to 13.92)	−1.19 (−1.29 to −1.10)	22.43 (21.29 to 23.58)	0.32 (0.27 to 0.37)	11.66 (11.32 to 11.99)	−0.54 (−0.59 to −0.50)	21.92 (21.45 to 22.39)	0.08 (−0.01 to 0.14)	12.63 (12.48 to 12.79)	−0.80 (−0.86 to −0.76)
Eastern Sub-Saharan Africa	7.03 (6.97 to 7.10)	0.44 (0.04 to 0.79)	6.31 (6.26 to 6.35)	0.18 (−0.23 to 0.53)	11.56 (11.31 to 11.80)	0.77 (0.65 to 0.88)	9.44 (9.30 to 9.58)	0.55 (0.41 to 0.68)	9.22 (9.13 to 9.31)	0.63 (0.34 to 0.89)	7.79 (7.73 to 7.84)	0.50 (0.06 to 0.74)
High-income Asia Pacific	47.15 (44.66 to 49.64)	0.64 (0.62 to 0.67)	31.77 (30.76 to 32.79)	0.36 (0.34 to 0.38)	36.75 (32.96 to 40.55)	0.86 (0.83 to 0.88)	22.44 (20.82 to 24.06)	0.49 (0.45 to 0.53)	41.97 (39.79 to 44.16)	0.75 (0.73 to 0.77)	26.91 (26.06 to 27.77)	0.42 (0.40 to 0.44)
High-income North America	39.57 (38.67 to 40.47)	−0.38 (−0.48 to −0.31)	20.04 (19.77 to 20.30)	−0.91 (−1.05 to −0.84)	38.75 (37.17 to 40.33)	−0.19 (−0.24 to −0.14)	18.63 (18.18 to 19.08)	−0.55 (−0.63 to −0.50)	39.25 (38.42 to 40.07)	−0.28 (−0.36 to −0.21)	19.33 (19.09 to 19.56)	−0.73 (−0.85 to −0.67)
North Africa and Middle East	14.24 (14.05 to 14.43)	0.66 (0.60 to 0.71)	10.22 (10.13 to 10.31)	−0.18 (−0.27 to −0.13)	14.44 (13.97 to 14.91)	1.59 (1.55 to 1.63)	8.54 (8.38 to 8.69)	0.46 (0.41 to 0.49)	14.42 (14.21 to 14.62)	1.05 (1.00 to 1.09)	9.53 (9.45 to 9.61)	0.08 (0.01 to 0.13)
Oceania	6.99 (6.68 to 7.29)	−0.09 (−0.22 to 0.08)	6.48 (6.25 to 6.72)	−0.25 (−0.40 to −0.05)	10.12 (8.77 to 11.46)	0.10 (0.04 to 0.17)	7.70 (7.01 to 8.38)	0.01 (−0.08 to 0.09)	8.52 (8.05 to 8.99)	0.00 (−0.10 to 0.11)	7.12 (6.84 to 7.40)	−0.11 (−0.19 to 0.03)
South Asia	7.90 (7.85 to 7.95)	0.83 (0.78 to 0.86)	6.89 (6.86 to 6.93)	0.50 (0.44 to 0.54)	9.69 (9.53 to 9.84)	1.13 (1.08 to 1.17)	7.28 (7.21 to 7.35)	0.77 (0.73 to 0.80)	8.79 (8.73 to 8.85)	1.05 (1.00 to 1.09)	7.11 (7.08 to 7.14)	0.63 (0.59 to 0.67)
Southeast Asia	12.59 (12.46 to 12.72)	0.91 (0.85 to 0.99)	10.73 (10.65 to 10.82)	0.48 (0.41 to 0.57)	14.40 (13.98 to 14.83)	1.07 (1.00 to 1.14)	10.51 (10.32 to 10.70)	0.61 (0.53 to 0.69)	13.52 (13.35 to 13.68)	0.99 (0.92 to 1.06)	10.67 (10.58 to 10.75)	0.55 (0.47 to 0.64)
Southern Latin America	25.90 (24.21 to 27.59)	0.22 (0.18 to 0.26)	19.74 (18.98 to 20.50)	−0.34 (−0.40 to −0.29)	24.58 (21.32 to 27.84)	0.18 (0.15 to 0.21)	17.86 (16.34 to 19.37)	−0.33 (−0.37 to −0.30)	25.26 (23.62 to 26.89)	0.20 (0.16 to 0.23)	18.80 (18.09 to 19.51)	−0.34 (−0.39 to −0.31)
Southern Sub-Saharan Africa	9.11 (8.98 to 9.24)	−0.98 (−1.07 to −0.90)	7.67 (7.58 to 7.76)	−1.46 (−1.58 to −1.37)	12.67 (12.28 to 13.06)	0.15 (0.06 to 0.23)	9.44 (9.24 to 9.64)	−0.43 (−0.54 to −0.33)	10.91 (10.76 to 11.07)	−0.38 (−0.46 to −0.30)	8.59 (8.50 to 8.68)	−0.95 (−1.03 to −0.88)
Tropical Latin America	17.71 (17.32 to 18.11)	0.48 (0.44 to 0.50)	14.29 (14.06 to 14.53)	−0.04 (−0.07 to −0.01)	18.50 (17.49 to 19.51)	0.56 (0.52 to 0.58)	13.73 (13.27 to 14.20)	0.09 (0.05 to 0.12)	18.18 (17.76 to 18.60)	0.51 (0.49 to 0.53)	14.08 (13.86 to 14.29)	0.00 (−0.03 to 0.03)
Western Europe	44.56 (42.90 to 46.22)	0.49 (0.42 to 0.55)	27.46 (26.88 to 28.04)	−0.13 (−0.23 to −0.07)	38.27 (35.89 to 40.65)	0.50 (0.46 to 0.54)	21.37 (20.42 to 22.31)	−0.01 (−0.06 to 0.03)	41.45 (40.05 to 42.85)	0.53 (0.48 to 0.57)	24.30 (23.81 to 24.80)	−0.04 (−0.09 to 0.01)
Western Sub-Saharan Africa	6.80 (6.75 to 6.85)	0.59 (0.48 to 0.67)	6.27 (6.23 to 6.31)	0.31 (0.19 to 0.38)	8.96 (8.79 to 9.14)	1.11 (1.07 to 1.14)	7.27 (7.17 to 7.36)	0.88 (0.82 to 0.91)	7.99 (7.92 to 8.06)	0.96 (0.89 to 1.01)	6.88 (6.83 to 6.92)	0.68 (0.59 to 0.73)

GBD, Global Burden of Disease

In 2021, tobacco emerged as the dominant attributable risk factor for lifetime cancer death risk across most cancer types and regions globally. For tracheal, bronchus, and lung (TBL) cancer, tobacco demonstrated the highest attributable lifetime death risks, with particularly pronounced burdens in High-income Asia Pacific (3.17%, 95% CI: 2.86 to 3.49), East Asia (3.93%, 95% CI: 3.52 to 4.34), Central Europe (2.96%, 95% CI: 2.73 to 3.19), Western Europe (2.94%, 95% CI: 2.68 to 3.20), and High-income North America (2.86%, 95% CI: 2.58 to 3.15). Air pollution constituted another major contributor to TBL cancer lifetime death risk, with the highest burden observed in East Asia (1.53%, 95% CI: 1.00 to 2.06) and Australasia (2.16%, 95% CI: 1.88 to 2.44), reflecting substantial regional variation in environmental exposures. For cervical cancer, unsafe sex represented the predominant risk factor, with the highest attributable lifetime death risks recorded in Central Sub-Saharan Africa (1.02%, 95% CI: 1.01 to 1.02) and Southern Sub-Saharan Africa (0.90%, 95% CI: 0.89 to 0.90). High body mass index (BMI) emerged as a key contributor across multiple cancer types, including colon and rectum cancer, breast cancer, uterine cancer, and kidney cancer, with the highest regional burdens generally observed in high- and high-middle-SDI regions such as Australasia and High-income North America. High fasting plasma glucose demonstrated notable contributions to TBL cancer, colon and rectum cancer, and pancreatic cancer lifetime death risks, with the most substantial regional burdens observed in East Asia and Southeast Asia. Occupational risks contributed most prominently to mesothelioma lifetime death risk across most regions, with relatively consistent patterns globally. For liver cancer, drug use showed notable attributable risks in specific regions, while high BMI and high fasting plasma glucose contributed substantially in high-SDI regions such as High-income North America and Western Europe. Tobacco use was identified as a major contributor to stomach cancer lifetime death risk, with the highest burden observed in East Asia (0.47%, 95% CI: 0.38 to 0.56) (Fig. S7). As a sensitivity analysis, correlations between lifetime risks and cumulative risks (ages 0 to 74) of developing and dying from cancer presented in Fig. S8. Notably, these 2 indicators differed substantially with varying life expectancy levels. Among the 133 countries or territories with a life expectancy below 74 years, the cumulative risk of developing or dying from cancer in the 0-to-74 age group exceeded the corresponding lifetime risk in 67 of these nations. In contrast, across all 71 countries or territories where life expectancy surpassed 74 years, lifetime risks of cancer development and cancer-related death consistently exceeded the cumulative risk estimated for the 0-to-74 age range. For example, in Japan, where life expectancy reaches 85.15 years, the cumulative risks of developing (21.73%, 95% CI: 21.69 to 21.76) and dying from (8.43%, 95% CI: 8.41 to 8.44) cancer among those aged 0 to 74 years were substantially lower than the corresponding lifetime risks of developing (43.51%, 95% CI: 40.71 to 46.31) and dying from (27.70%, 95% CI: 26.61 to 28.79) cancer. Conversely, in Eswatini, where mean life expectancy is as low as 52.51 years, the cumulative risk for the 0-to-74 age group proved to be an overestimate relative to the lifetime risk, both for developing cancer (19.64%, 95% CI: 19.46 to 19.82 vs. 8.44%, 95% CI: 7.76 to 9.12) and dying from cancer (16.55%, 95% CI: 16.38 to 16.64 vs. 6.77%, 95% CI: 6.63 to 7.21).

### Lifetime risks of developing and dying from cancer by sex and age

Globally, lifetime risks of both developing and dying from cancer were elevated among men compared with women, with notable regional and national variations (Figs. S1 and S2). The global lifetime risk of developing cancer was 22.15% (95% CI, 22.07 to 22.23) for men and 21.03% (95% CI, 20.88 to 21.19) for women, while the lifetime risk of dying from cancer was 15.19% (95% CI: 15.16 to 15.23) for men and 13.06% (95% CI: 13.00 to 13.12) for women (Tables S3 and S4). Among the 21 GBD regions, 8 regions showed a higher lifetime risk of developing cancer in men, while the remaining 13 regions exhibited a higher risk in women (Table 1). Notably, East Asia, High-income Asia Pacific, and Australasia showed more than 20% higher risk of developing cancer in men compared with women, whereas Eastern Sub-Saharan Africa, Central Sub-Saharan Africa, and Oceania had more than 40% higher risk in women compared with men (Table S5).

In middle- and higher-SDI countries, men had higher lifetime risks of developing and dying from cancer compared with women. In contrast, women in low-middle- and low-SDI countries faced higher lifetime risks (Tables S3 and S4). Moreover, marked variations in sex-specific cancer risks were noted between countries. Monaco exhibited the highest incidence and death risks for both men and women, while the lowest incidence risks were recorded in the Central African Republic for men and Niger for women. The lowest death risks were observed in Somalia for men and Oman for women (Tables S7 and S8).

**Fig. 2.**
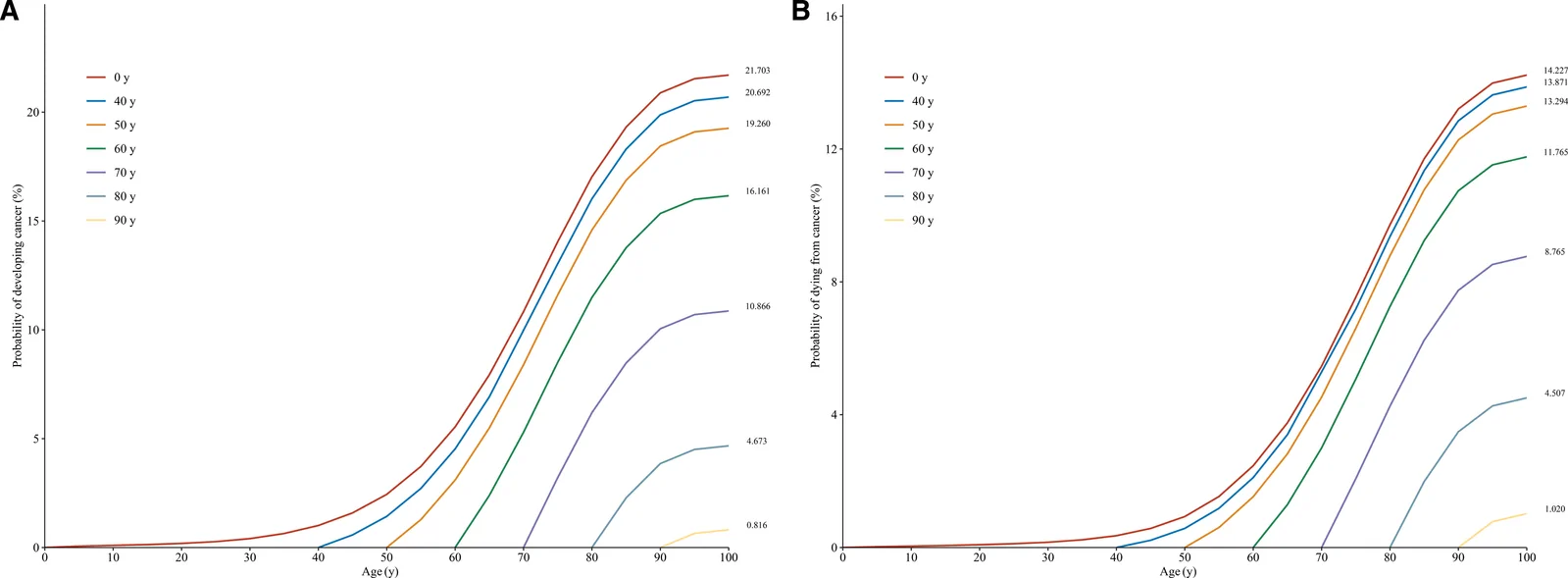
Global lifetime risks of cancer for both sexes by age at diagnosis in 2021. (A) Lifetime incidence risk. (B) Lifetime death risk. Note: The curves depict the probability of developing or dying from cancer across different baseline ages (at birth, 40, 50, 60, 70, 80, and 90 y) for individuals with cancer-free status at those respective time points. The cumulative risk through death represents the remaining lifetime probability from each baseline age.

In 2021, the remaining lifetime risks of developing and dying from cancer at specific ages (40, 50, …, 90 y) varied by sex (Tables S15 and S16; Figs. S3 and S4). Globally, the probability of developing cancer before age 50 was relatively low, estimated at 2.44% (95% CI, 2.41 to 2.46), leading to nearly comparable lifetime risks when assessed from birth to death versus from age 50 onward for both sexes. The risk of dying from cancer showed a similar trend. Overall, the remaining lifetime risks of developing and dying from cancer decreased as the starting age increased, reflecting the progressively shorter remaining life expectancy at older ages. Individuals who were cancer-free at age 70 had a 4.67% (95% CI, 4.67 to 4.68) probability of developing cancer and a 4.51% (95% CI, 4.50 to 4.51) probability of dying from it over their remaining lifetime (Fig. 2; Table S14).

### Lifetime risks of developing and dying from different cancer types in 2021

**Fig. 3.**
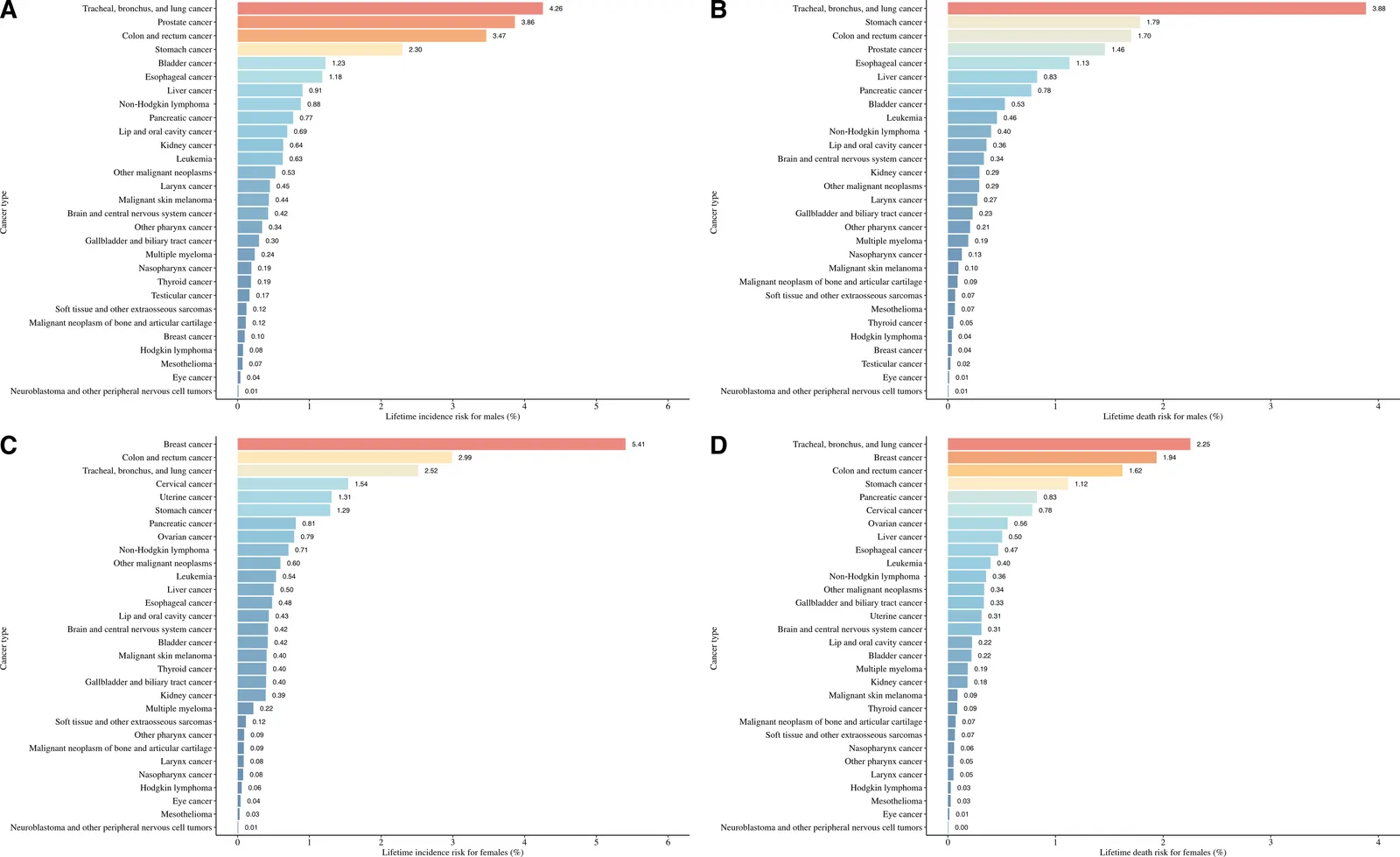
Global lifetime risks of different cancer types for males and females in 2021. (A) Lifetime incidence risk for males. (B) Lifetime death risk for males. (C) Lifetime incidence risk for females. (D) Lifetime death risk for females.

**Fig. 4.**
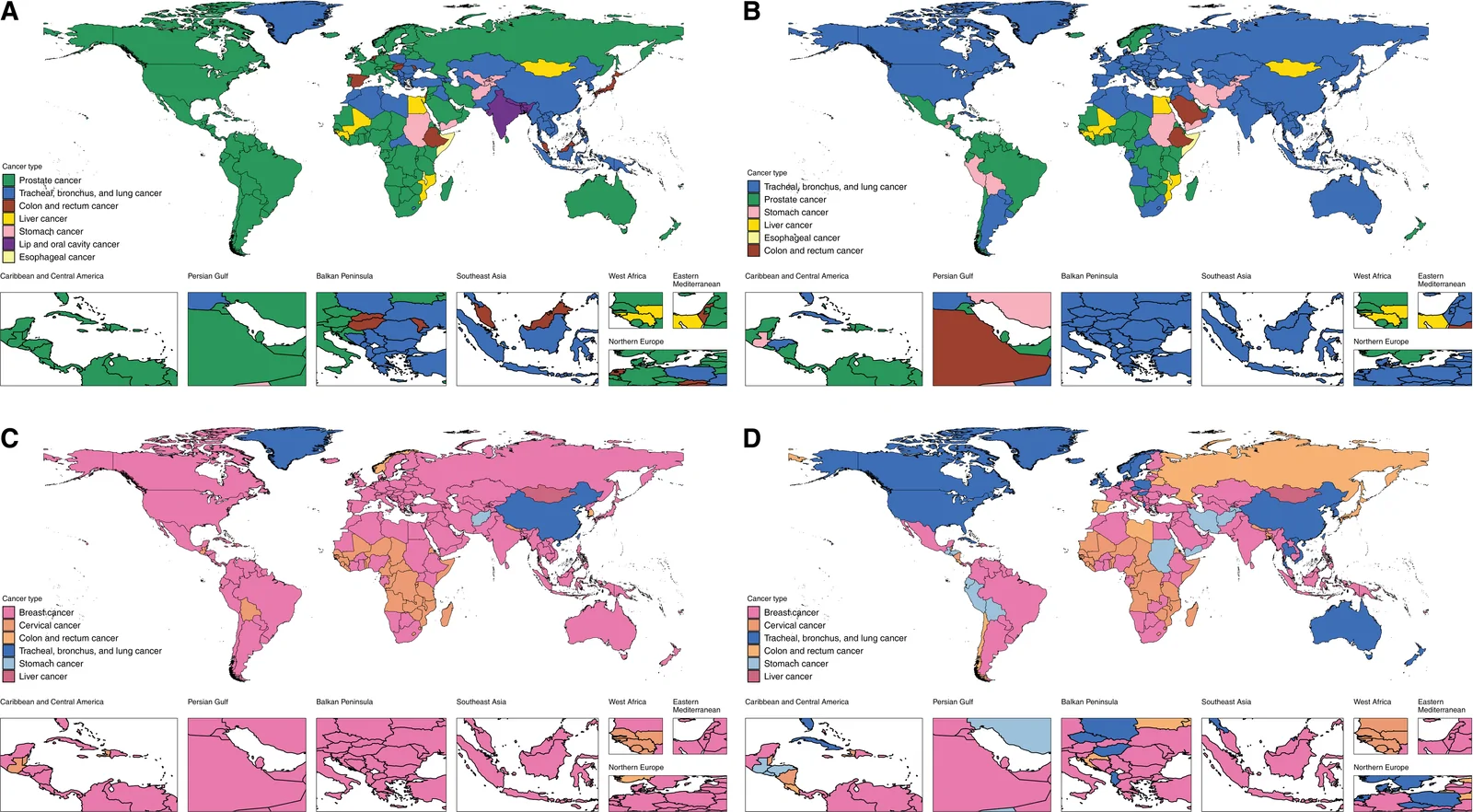
The most common type of lifetime risks of cancer at national level for males and females in 2021. (A) Lifetime incidence risk for males. (B) Lifetime death risk for males. (C) Lifetime incidence risk for females. (D) Lifetime death risk for females.

Globally, among males, TBL presented the greatest risks for both incidence (4.26%, 95% CI: 4.25 to 4.26) and death (3.88%, 95% CI: 3.88 to 3.89). In females, breast cancer ranked highest in lifetime incidence risk (1.94%, 95% CI: 1.93 to 1.94), whereas TBL remained the leading cause of lifetime death risk (2.25%, 95% CI: 2.25 to 2.26) (Fig. 3; Table S9). Across SDI quintiles, high-SDI regions exhibited prostate cancer as the predominant cancer in males, accounting for 21.2% of the cancer incidence risk, with its proportion progressively declining across the SDI spectrum from high to low regions. Regarding cancer death risk, TBL represents the highest risk across all SDI quintiles, with its burden representing the highest intensity in high-SDI regions (24.1%). For females, breast cancer exhibited the largest proportion of cancer incidence and death risk in high-SDI regions (22.2% and 24.5%, respectively), progressively decreasing in lower-SDI regions, while cervical cancer demonstrated an inverse relationship with development—accounting for only 2.6% and 2.0% of incidence and death risk in high-SDI regions but increasing to 21.7% and 18.4% in low-SDI regions (Tables S12 and S13; Figs. S9 and S10). At the national level, marked worldwide differences in dominant cancer forms were observable. Particularly, in men, prostate cancer accounted for the highest lifetime incidence and death risks in 147 and 177 nations, respectively, followed by colon and rectum cancer [20,21], stomach cancer [13,22], liver cancer [8,10], lip and oral cavity cancer [2,5], esophageal cancer [3,4], and bladder cancer [1]. Among women, breast cancer had the greatest incidence and death risks in 161 and 131 nations, respectively, followed by cervical cancer [23,24], colon and rectum cancer [3,25], stomach cancer [1,14] and liver cancer [1,2] (Fig. 4; Tables S12 and S13).

### Global trends in lifetime risks of developing and dying from cancer

Globally, the lifetime risk of developing cancer increased by 14.0% between 1990 (19.04%, 95% CI: 18.97 to 19.12) and 2021, while the risk of dying from cancer slightly decreased from 14.50% (95% CI, 14.48 to 14.52) in 1990 to 2021. During the COVID-19 pandemic, the lifetime risk of developing cancer sharply decreased with an AAPC of −4.73% (95% CI, −5.18 to −4.40). Similarly, the lifetime risk of dying from cancer decreased with an AAPC of −6.24% (95% CI, −6.74 to −5.92) (Fig. 1; Tables S3 and S4). Sex-stratified data from 1990 to 2021 showed a more pronounced increase in lifetime risk of developing cancer in women than in men (AAPC: 0.46%, 95% CI: 0.44 to 0.48 vs. 0.32%, 95% CI: 0.28 to 0.34). Conversely, the lifetime risk of dying from cancer exhibited different patterns between the sexes, with men experiencing a decrease (AAPC: −0.20%, 95% CI: −0.25 to −0.16), while women’s risk remained stable. During the COVID-19 pandemic, both sexes showed marked decreases in the lifetime risks of developing and dying from cancer, with men experiencing steeper declines in both metrics (AAPC in incidence risk: −6.27%, 95% CI: −6.88 to −5.83 vs. −3.31%, 95% CI: −3.69 to −3.05; AAPC in death risk: −8.11%, 95% CI: −8.88 to −7.70 vs. −4.66%, 95% CI −5.06 to −4.39) (Figs. S11 and S12; Tables S18 and S19).

### Trends in lifetime risks of developing and dying from cancer by SDI quintile, region, nation, and cancer type

**Fig. 5.**
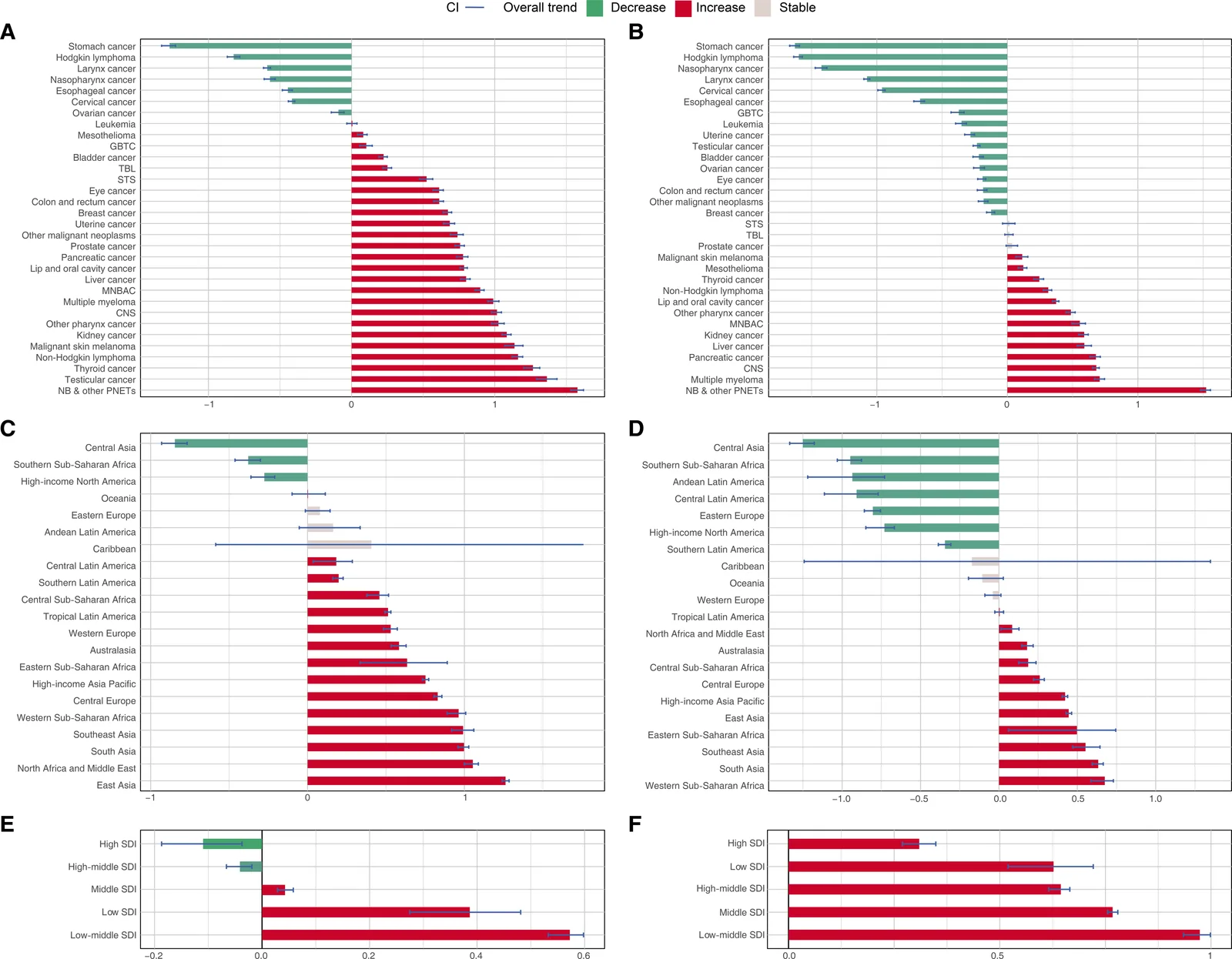
AAPCs in lifetime risks of cancer for both sexes from 1990 to 2021. (A) Lifetime incidence risk by cancer type. (B) Lifetime death risk by cancer type. (C) Lifetime incidence risk by Global Burden of Disease (GBD) region. (D) Lifetime death risk by GBD region. (E) Lifetime incidence risk by sociodemographic index (SDI) quintile. (F) Lifetime death risk by SDI quintile. In the figure, CI, refers to confidence interval, CNS refers to brain and central nervous system cancer, GBTC refers to gallbladder and biliary tract cancer, MNBAC refers to malignant neoplasm of bone and articular cartilage, NB & other PNETs refers to neuroblastoma and other peripheral nervous cell tumors, STS refers to soft tissue and other extraosseous sarcomas, and TBL refers to tracheal bronchus and lung cancer.

Temporal trends in lifetime cancer risk varied considerably across SDI levels. From 1990 to 2021, the lifetime risk of developing cancer rose in all SDI quintiles, with the most pronounced increase observed in low-middle-SDI countries (AAPC: 0.97%, 95% CI: 0.94 to 1.00). For lifetime death risk, a distinct socioeconomic gradient emerged. Countries in middle- (AAPC: 0.39%, 95% CI: 0.27 to 0.48), low-middle- (0.04%, 95% CI: 0.03 to 0.06), and low-SDI (0.57%, 95% CI: 0.53 to 0.60) categories experienced increasing risks, whereas those in high- and high-middle-SDI quintiles showed declining trends (AAPC: −0.11%, 95% CI: −0.19 to −0.04 and −0.04%, 95% CI: −0.07 to −0.02, respectively). During the COVID-19 pandemic, a universal decrease was observed across all SDI levels, with countries in the low-SDI category experiencing the most substantial reductions in both incidence risk (AAPC: −7.11%, 95% CI: −8.63 to −4.20) and death risk (AAPC: −9.48%, 95% CI: −11.06 to −8.38) (Fig. 5; Table S19). Similar patterns were observed between sexes (Figs. S13 and S14).

Among the 21 GBD regions, most showed increases in the lifetime risk of developing cancer, with 3 regions—Central Asia, Southern Sub-Saharan Africa, and High-income North America—exhibiting decreases. East Asia experienced the highest increase (AAPC: 1.26%, 95% CI: 1.24 to 1.29). Regarding the lifetime risk of dying from cancer, 10 regions showed increases, 7 experienced decreases, and 4 regions maintained stable trends. Western Sub-Saharan Africa recorded the largest increase in lifetime death risk (AAPC: 0.68%, 95% CI: 0.59 to 0.73), while Central Asia exhibited the most substantial decrease (AAPC: −1.25%, 95% CI: −1.33 to −1.18) (Fig. 5; Tables S18 and S19). Similar trends were evident for both males and females after stratification (Figs. S14 and S15). During the COVID-19 pandemic, most regions experienced declines in both lifetime incidence and death risks. However, East Asia remained an exception, continuing to show increases in lifetime risk of developing (AAPC: 1.23%, 95% CI: 1.10 to 1.54) and dying from cancer (AAPC: 0.32%, 95% CI: 0.24 to 0.50) for both sexes. The Caribbean also stood out, with a rising trend in the risk of developing cancer specifically among women (AAPC: 0.78%, 95% CI: 0.33 to 1.21) (Tables S20 and S21).

At the national level, between 1990 and 2021, Egypt recorded the most pronounced increase in lifetime risk of developing cancer, with an AAPC of 1.91% (95% CI: 1.87 to 1.94), while Kazakhstan experienced the largest decline (AAPC: −1.57%, 95% CI: −1.65 to −1.49). For lifetime death risk, Uganda exhibited the highest increase (AAPC: 1.60%, 95% CI: 1.48 to 1.71), whereas Kazakhstan reported the steepest decline (AAPC: −2.32%, 95% CI: −2.45 to −2.21). During the COVID-19 period, most countries displayed stable or decreasing trends, with only 20 countries and territories reporting an increase in lifetime incidence risk and 14 showing a rise in lifetime death risk (Tables S22 and S23).

Neuroblastoma and other peripheral nervous cell tumors showed the most rapid increases in both lifetime incidence and death risk (AAPC: 1.58%, 95% CI: 1.53 to 1.62 and 1.52%, 95% CI: 1.48 to 1.56, respectively), while stomach cancer exhibited the steepest declines in both metrics (AAPC: −1.27%, 95% CI: −1.33 to −1.23 and −1.63%, 95% CI: −1.67 to −1.59, respectively) (Fig. 4; Table S24). Sex-stratified data revealed that, among men, breast cancer demonstrated the fastest increase in lifetime incidence risk (AAPC: 2.22%, 95% CI: 2.16 to 2.27), while Hodgkin lymphoma exhibited the most substantial decrease in lifetime death risk (AAPC: −1.69%, 95% CI: −1.74 to −1.66). Among women, neuroblastoma and other peripheral nervous cell tumors presented the fastest increase in lifetime incidence risk (AAPC: 1.35%, 95% CI: 1.30 to 1.39), while nasopharyngeal cancer showed the most pronounced decrease in lifetime death risk (AAPC: −1.77%, 95% CI: −1.82 to −1.72) (Figs. S13 and S14).

## Discussion

### Global patterns and socioeconomic gradients in lifetime cancer risk

In 2021, the lifetime risk of developing cancer was estimated at 1 in 5, and the risk of dying from cancer at 1 in 7 for both sexes, globally. Notably, both lifetime risks show a strong positive correlation with SDI. High-SDI regions, including Australasia and High-income Asia Pacific, exhibit the highest risks, while the lowest risks are observed in Central Sub-Saharan Africa. These geographic variations reflect disparities in healthcare infrastructure and population demographics. Specifically, high-SDI countries benefit from advanced screening systems and aging populations with longer life expectancies, which increase the likelihood of developing and dying from cancer [26]. Consistent with our findings, prior research using incidence rates has demonstrated a higher cancer burden in regions as SDI levels rise. However, higher mortality rates were reported in low- and low-middle-SDI regions [25]. The higher estimate of lifetime death risk in high-SDI regions, despite their lower cancer mortality rates, highlights the importance of continued focus on early screening and prevention in these areas to reduce lifetime death risks [27].

### Comparison with prior estimates and sensitivity analysis

Compared with the global lifetime cancer incidence risk of 25.10% (95% CI, 25.08 to 25.11) reported by Zheng et al. [3] using GLOBOCAN 2020 data, our GBD 2021-based estimate of 21.70% is lower. Several factors may explain this discrepancy. First, systematic differences exist between the 2 databases: GLOBOCAN relies primarily on population-based cancer registries and tends to yield higher incidence estimates, whereas GBD 2021 employs a Bayesian ensemble modeling approach integrating multiple data sources [28]. For the same reference year 2020, GLOBOCAN estimated approximately 18.1 million new cancer cases globally (excluding NMSC), compared with 16.85 million estimated by GBD 2021, which would lead to a higher estimate of lifetime risk when used. Second, our analysis extends to 2021, fully capturing the COVID-19 pandemic’s impact of elevated all-cause mortality, which mathematically reduces the estimated lifetime cancer risk [9]. Despite these quantitative differences, both studies consistently identify substantial variation in lifetime cancer risk by sex, age group, and geographic region.

The lifetime risks estimated in this study for developing and dying from cancer from birth to death exceeded cumulative risk estimates restricted to the 0-to-74 age range. This finding is consistent with prior work demonstrating that cumulative risk tends to underestimate true risk in populations where life expectancy is relatively high or competing mortality from other causes is relatively low [4,29,30]. The cumulative risk for the 0-to-74 age group assumes survival to age 74 and is independent of population-level mortality probabilities and life expectancy, making it a useful tool for cross-population comparisons of cancer risk. It serves as a reasonable approximation of lifetime risk when a population’s average life expectancy approximates 74 years. Nevertheless, as of 2021, more than 70 countries reported life expectancies exceeding 74 years, meaning that the cumulative risk for ages 0 to 74 would systematically underestimate the true lifetime risk in these settings. Unlike approaches that fix the upper age boundary at 74 years, the AMP-method-derived lifetime risk accounts for the cancer burden beyond age 74 up to the population’s life expectancy. It further incorporates multiple primary cancers and the competing risk of death from non-cancer causes [28,31].

### Modifiable risk factors and attributable lifetime cancer death risk

Our attributable risk analysis reveals that tobacco remains the single most important modifiable risk factor for cancer lifetime death risk globally, particularly for TBL cancer, consistent with well-established epidemiological evidence linking tobacco carcinogens to lung carcinogenesis [20]. The disproportionately high tobacco-attributable TBL cancer burden in East Asia and High-income Asia Pacific likely reflects historically high smoking prevalence among males in these regions [22], compounded by the delayed manifestation of tobacco-related cancer mortality following peak smoking periods [32]. The concurrent substantial contribution of air pollution to TBL cancer lifetime death risk in East Asia underscores the synergistic carcinogenic effects of tobacco and environmental pollutants [33,34], suggesting that dual-targeted interventions addressing both tobacco control and air quality regulation are imperative in these regions. The predominance of unsafe sex as the leading risk factor for cervical cancer lifetime death risk in Sub-Saharan Africa directly reflects insufficient human papillomavirus (HPV) vaccination coverage and limited access to cervical cancer screening in these low-SDI settings, consistent with the inverse relationship between SDI and cervical cancer burden reported earlier in this study. These findings reinforce the urgent need for scalable HPV vaccination programs and visual-inspection-based screening strategies in resource-limited settings [35]. The broad contribution of high BMI across multiple cancer types—including colorectal, breast, uterine, kidney, and pancreatic cancers—in high-SDI regions highlights the growing cancer burden attributable to the global obesity epidemic [36]. This pattern is particularly pronounced in Australasia and High-income North America, where the obesity prevalence is among the highest globally, suggesting that population-level weight management interventions could yield substantial reductions in cancer mortality across multiple cancer types simultaneously [37,38]. The notable contribution of high fasting plasma glucose to TBL, colorectal, and pancreatic cancer lifetime death risks, particularly in East Asia and Southeast Asia, underscores the cancer prevention potential of metabolic disease management in regions experiencing rapid epidemiological transitions characterized by increasing rates of type 2 diabetes [8,24]. These findings suggest that integrated chronic disease prevention strategies targeting both obesity and glycemic control could generate co-benefits for cancer control in these regions. The consistent contribution of occupational risks to mesothelioma across regions reflects ongoing asbestos exposure in many countries despite regulatory restrictions, highlighting the need for strengthened occupational health policies and continued monitoring of asbestos-related cancer burden [23,39]. The relatively modest but geographically heterogeneous contributions of drug use and occupational risks to liver cancer lifetime death risk further emphasize the complexity of liver cancer etiology, wherein region-specific cofactors such as endemic hepatitis B and C infections likely interact with these modifiable risk factors to amplify cancer mortality burden [37].

### Age-specific lifetime cancer risk and clinical implication

Age-specific analyses reveal that the remaining lifetime risks of developing and dying from cancer decrease progressively with age. To facilitate clinically meaningful interpretation, we selected ages 50 and 70 as key thresholds, informed by both data patterns and established clinical practice. Prior to age 50, cancer risk accumulates minimally, with the remaining lifetime incidence risk declining only marginally from birth (21.70%) to age 50 (19.26%), a difference of merely 2.44% (Table S14), rendering lifetime risk estimates from birth comparable to those calculated from age 50 onward. This threshold also aligns with the recommended initiation age for cancer screening in major clinical guidelines, including those for colorectal and breast cancer [40,41]. Beyond age 70, risk reduction accelerates markedly, with remaining lifetime incidence risk dropping from 10.87% at age 70 to 4.67% at age 80 and 0.82% at age 90 (Table S14), consistent with age 70 being a widely adopted threshold for defining older adulthood in oncological research. Similar patterns were observed for lifetime death risk [42]. This observation reflects the mathematical nature of conditional probability in lifetime risk calculations. For individuals who have reached 70 years of age, their remaining lifetime cancer risk represents a conditional probability encompassing multiple criteria: survival to age 70, absence of prior cancer diagnosis, and avoidance of cancer-related mortality up to that point. As these conditions accumulate, the probability naturally decreases, explaining the lower observed risk in this age group [21]. This statistical property has important clinical implications for designing age-appropriate screening strategies that optimize the balance between early detection benefits and over diagnosis risks in elderly populations [43].

### Sex disparities in lifetime cancer risk across SDI levels

Our analysis reveals substantial sex-based disparities in the global cancer burden, with males exhibiting higher lifetime risks of both developing and dying from cancer relative to females. These differences likely arise from a combination of biological susceptibility, behavioral patterns, differential exposure to occupational carcinogens, and healthcare access disparities [31,44,45]. Further stratification by SDI highlights the complexity of risk distribution: Males predominantly face higher cancer risk in high-to-middle-SDI countries, while females show higher risks in low- and low-middle-SDI countries, reflecting the interplay of healthcare resource allocation and risk factor exposure. In high-SDI regions, prostate cancer and TBL emerge as major burdens among males [46,47] and breast cancer exhibits the highest incidence risk among females. In contrast, females in low-SDI regions face pressures from cervical cancer and its incidence and death risk is approximately 8 times and 9 times higher than in high-SDI countries, directly correlating with insufficient HPV vaccination and low cervical cytology screening coverage [48–50]. In East Asia, the overall cancer risk for males exceeds that for females by more than 20%, which is closely associated with smoking culture and occupational exposure. Conversely, in Sub-Saharan Africa, female risk surpasses male risk by more than 40%, predominantly due to breast and cervical cancers, which account for nearly half of the total cancer risk among women. Policy development must be tailored according to SDI levels. High-SDI regions should prioritize targeted screening for prostate and TBL cancer and strengthen primary prevention measures for breast cancer while advancing tobacco control legislation [51–53]. Low-SDI regions need to enhance early diagnosis capabilities for cervical cancer through scalable HPV vaccination programs and clinical cervical visual-inspection screening [54].

### Temporal trends and the impact of the COVID-19 pandemic

The temporal trends in cancer lifetime risks from 1990 to 2021 reveal a complex picture characterized by 2 distinct periods. While the overall period showed an increase in lifetime risk of developing cancer (from 19.04% to 21.70%) and a slight decrease in risk of dying from cancer (from 14.50% to 14.23%), the pre-pandemic years demonstrated steady increases in both risks before unprecedented decreases during the COVID-19 pandemic markedly influenced these long-term trends. From 1990 to 2021, distinct patterns emerged across SDI regions. High-SDI regions observed decreases in lifetime risks of both developing and dying from cancer after 2010 and 2011, respectively, indicating important improvements in cancer management and treatment in these areas [55,56]. The rapid increase in lifetime risk of developing and dying from cancer observed in low- and low-middle-SDI countries reveals the side effects of socioeconomic transformation, while infection-related cancers remain dominant and medical resources and preventive measures are insufficient. This phenomenon is particularly evident in Egypt and Uganda, highlighting the imperative for tailored national health system strategies and comprehensive cancer prevention and control initiatives. In Egypt, efforts should focus on controlling the prevalence of hepatitis C, hepatitis B, and schistosomiasis [57], while in Uganda, advancing antiretroviral therapy could help reduce the burden of Kaposi sarcoma and non-Hodgkin lymphoma [58]. The sustained increase in East Asia (AAPC: 1.26%) is likely driven by converging demographic and lifestyle transitions. Rapid population aging has substantially expanded the at-risk population, while the progressive adoption of Westernized dietary patterns, characterized by increased consumption of red meat, processed foods, and alcohol, has contributed to rising incidence of colorectal and breast cancers [59]. Concurrently, the expansion of organized screening programs has enhanced case ascertainment, further elevating observed incidence rates [60]. These intersecting forces suggest that East Asia’s rising lifetime cancer risk reflects both genuine epidemiological transitions and improved detection capacity.

Further analysis revealed that these overall trends were driven by distinct patterns across different cancer types. Neuroblastoma and other peripheral nervous system tumors showed the most substantial increases in both lifetime incidence and death risks, consistent with previous studies reporting rising trends in incidence and mortality [61]. Despite some success with the introduction of high-dose chemotherapy, stem cell transplantation, and immunotherapy in recent years, future research and treatment strategies should continue to focus on reducing long-term health risks and improving quality of life [62]. Stomach cancer exhibited the steepest decreases in both incidence and death risks, attributable to multiple factors, including popularization of cancer early detection programs, control of *Helicobacter pylori* infection, and dietary and lifestyle modifications [63]. Among men, breast cancer demonstrated the fastest increase in lifetime incidence risk, a previously overlooked trend that warrants attention despite its relatively low baseline risk. This finding highlights concerns about potential underdiagnosis and poor prognosis in male breast cancer patients, suggesting the need for adopting a healthier lifestyle, including reducing tobacco and alcohol use and improving dietary habits [64].

The global decline in cancer lifetime risk during the COVID-19 pandemic reflects 3 overlapping mechanisms. The first is COVID-19-related excess all-cause mortality functioning as a competing risk, which mathematically reduces estimated lifetime cancer risk within the AMP framework. The second is widespread healthcare disruptions, including suspension of screening programs and delays in diagnostic workup, which suppressed recorded cancer incidence independently of true disease occurrence. The third is potential underreporting arising from reduced surveillance capacity, particularly in low-SDI settings where pre-pandemic cancer registration completeness was already below 50% in many regions [65]. While all 3 mechanisms likely contributed, we argue that competing mortality was the predominant driver. Formally quantifying the relative contributions of all 3 pathways requires integration of excess mortality data with cancer registry records and screening utilization statistics at the national level, representing an important priority for post-pandemic cancer epidemiology [31]. Several lines of evidence support competing mortality as the predominant driver. First, the scale of COVID-19 excess mortality was sufficient to produce measurable competing risk effects. Global life expectancy declined by 1.6 years between 2019 and 2021, with excess mortality exceeding 150 deaths per 100,000 population in 80 countries during at least one pandemic year [66]. This magnitude of disruption mechanistically removes individuals from the at-risk pool before cancer onset or death, a phenomenon formally described by competing risk theory [67]. Second, the geographic pattern of declines contradicts a screening-disruption hypothesis. Screening disruption was most severe in high-SDI countries, where documented declines included a 39% reduction in screening participation and a 23% reduction in cancer diagnoses [68], with breast cancer screening volumes falling by 49% or more in many high-income settings [69]. If screening disruption were the primary driver, high-SDI regions where population-based cancer screening coverage is among the highest worldwide should have experienced the largest declines in lifetime risk. Instead, the steepest reductions occurred in low-SDI regions, particularly southern Sub-Saharan Africa, where COVID-19 excess mortality was among the highest globally at 308.6 deaths per 100,000 and lifetime cancer incidence risk declined at an AAPC of −13.81% (Table S20) [66]. This geographic inversion strongly favors competing mortality over screening disruption. Third, the simultaneous decline in both lifetime incidence and death risks is more parsimoniously explained by competing mortality, which suppresses both metrics in tandem. By contrast, screening disruption primarily depresses incidence through delayed diagnosis, with effects on mortality being only indirect and temporally lagged, consistent with evidence that pandemic disruptions disproportionately affected early-stage, screen-detected cancers rather than overall mortality [70–72]. Regarding underreporting, we acknowledge that low-SDI settings have long suffered from incomplete cancer registration, and that pandemic-related surveillance disruptions likely further suppressed reporting to some extent [73]. However, paradoxically, the contribution of underreporting may have been more limited in low-SDI regions than in high-SDI ones, as where baseline registration completeness is already severely deficient, the marginal capacity for further pandemic-related deterioration is constrained. We therefore treat underreporting as a contributing but secondary factor, while acknowledging that formally quantifying its contribution would require dedicated surveillance infrastructure analyses beyond the scope of the present study.

### Strengths and limitations

Several advantages characterize our investigation. First, we utilized the most recent and comprehensive data from GBD 2021, allowing for global lifetime risk estimation and providing comparable estimates across regions and countries [14]. Second, our concurrent calculation of lifetime risks for both cancer development and mortality provides an intuitive, population-level metric [4]. As demonstrated in this study, findings such as “one in five individuals will develop cancer during their lifetime” are far more comprehensible to policymakers and the public than age-standardized rates. By integrating competing causes of death across the entire lifespan, these estimates support long-term health system planning and risk communication [74]. The observed divergence between lifetime incidence and death risks across SDI levels further offers actionable signals for resource allocation: high-SDI regions should prioritize treatment capacity and survivorship care, while low-SDI regions require targeted investment in early detection [65]. Third, we analyzed cancer risk trends over a 32-year period, incorporating the COVID-19 pandemic’s impact, offering insights into the long-term evolution of the global cancer burden and strengthening healthcare system resilience.

Several limitations warrant consideration. First, data quality varies substantially across SDI levels. While high-SDI regions benefit from well-established cancer registry systems, low- and low-middle-SDI regions suffer from limited registry coverage, and GBD 2021 relies heavily on modeled estimates in these settings [8]. Therefore, observed increases in lifetime cancer risk in low-SDI regions should be interpreted with caution, as part of the apparent rise may reflect improved case ascertainment over time rather than true epidemiological changes. Findings from high- and high-middle-SDI regions, where registry coverage is more comprehensive, are comparatively more robust. Second, the observed decreases in cancer lifetime risks during the pandemic reflect a complex interplay of factors—COVID-19-related excess mortality, healthcare disruptions, and potential underreporting. Our study design cannot fully separate or quantify these effects. Notably, as the impact of COVID-19 on cancer risk is likely temporary in nature, lifetime cancer risks in the post-pandemic era may gradually return toward pre-pandemic trajectories. Future studies utilizing more recent data are strongly encouraged to evaluate post-pandemic recovery trajectories and further validate the long-term trends reported here. Third, the estimates of lifetime risk are based on current cross-sectional data [75]. With the ongoing evolution of lifestyle risk factors—such as obesity, physical inactivity, and changing dietary patterns—as well as the potential long-term effects of emerging risk factors and advances in cancer screening and treatment, future trends in lifetime risks may deviate from the patterns observed in this study. Therefore, regular updating of these estimates using prospective data remains essential for accurately reflecting the evolving global cancer burden. Finally, lifetime risk is intrinsically linked to life expectancy, warranting caution when interpreting variations across SDI regions and time periods [76]. Because longer life expectancy extends the window of cumulative cancer exposure, populations with higher life expectancy will inherently yield higher lifetime risk estimates even when underlying incidence and mortality rates are comparable [75]. Cross-regional and cross-temporal comparisons should therefore be interpreted alongside prevailing life expectancy data.

## Conclusion

Our study presents comprehensive estimates of lifetime cancer incidence and mortality risks at global, regional, and national levels, stratified by sex, age, SDI quintile, and cancer type, accounting for competing causes of death and multiple primary cancers, from 1990 to 2021. Globally, 1 in 5 individuals was diagnosed with cancer and 1 in 7 died from cancer during their lifetime in 2021. Lifetime cancer risks correlated positively with SDI and declined progressively with age, particularly after 70 years. Attributable risk analyses identified tobacco, unsafe sex, high BMI, air pollution, and high fasting plasma glucose as leading modifiable risk factors across specific cancer types and regions. The COVID-19 pandemic substantially disrupted these trajectories, primarily driven by competing excess mortality in low-SDI regions. Our findings underscore the need for SDI-tailored cancer control strategies that prioritize risk factor reduction and early detection in resource-limited settings and treatment capacity in high-SDI regions, supported by resilient healthcare systems capable of sustaining essential services during public health emergencies.

## Ethical Approval

This study is compliant with the Guidelines for Accurate and Transparent Health Estimates Reporting (GATHER) statement. The University of Washington institutional review board committee approved GBD 2021, and informed consent was waived because of the use of deidentified data.

## Data Availability

The raw data utilized in this study are publicly accessible through the 2021 Global Burden of Disease (GBD) Study database (https://vizhub.healthdata.org/gbd-results/). Most of the processed datasets and analytical results are presented in the Supplementary materials. Additional detailed data are available from the corresponding author upon reasonable request.
