# Supplementary material for: Global Patterns and Trends in Lifetime Risks of Developing and Dying from Cancer, 1990–2021
Source: Health Data Sci. 2026 Sep 10;6:0378. doi: 10.34133/hds.0378 (PMC13558824; doi:10.34133/hds.0378)
Supplement: Supplementary file 1 [file hds.0378.f1.docx]

**Supplementary Materials**

**Global patterns and trends in lifetime risks of developing and dying from cancer, 1990-2021**

**Zhongge Ji^1,2,#^, Yiming Song^1,#^, Zeyu Li^1,#^, Xiaojie Huang^3^, Sijia Zhai^2^, Qingran Liu^2^, Ruitian Zeng^4^, Ruijie Han^4^, Yufei Xiao^4^, Yuxin Yang^4^, Xiaolu Lin^3,*^, Qingwei Zhang^1,*^, Xiaobo Li^1,*^**

^1^Division of Gastroenterology and Hepatology, Shanghai Institute of Digestive Disease, NHC Key Laboratory of Digestive Diseases, Renji Hospital, Shanghai Jiao Tong University School of Medicine, Shanghai, China.

^2^The First School of Medicine, School of Information and Engineering, Wenzhou Medical University, Wenzhou, China.

^3^Department of Digestive Endoscopy Center, Fujian Provincial Hospital, Fuzhou University Affiliated Provincial Hospital, Shengli Clinical Medical College of Fujian Medical University, Fuzhou, Fujian, China

^4^School of Basic Medical Sciences, Fujian Medical University, Fuzhou, China

^#^ These authors contributed equally: Zhongge Ji, Yiming Song, Zeyu Li

**^*^ Corresponding authors**

Xiaolu Lin, Email: fjlinxiaolu@163.com

Qingwei Zhang, Email: zhangqingweif@hotmail.com

Xiaobo Li, Email: [lxb_1969@163.com](mailto:lxb_1969@163.com)

**CONTENT**

[Appendix 1. A brief introduction about the methods for lifetime risk of developing or dying from cancer	6](#_Toc10335)

[Table S1. International Classification of Diseases (ICD) codes of 32 GBD cancer causes used in lifetime risk analysis	9](#_Toc29877)

[Table S2. Initial age groups by cancer type for lifetime risk calculation	11](#_Toc26178)

[Table S3. Lifetime risk of developing cancer by sex globally and by SDI quintile in 1990, 2019 and 2021	13](#_Toc31966)

[Table S4. Lifetime risk of dying from cancer by sex globally and by SDI quintile in 1990, 2019 and 2021	14](#_Toc27249)

[Table S5. Lifetime risk of developing cancer by GBD region and sex in 1990, 2019 and 2021	15](#_Toc29684)

[Table S6. Lifetime risk of dying from cancer by GBD region and sex in 1990, 2019 and 2021	17](#_Toc28007)

[Table S7. Lifetime risk of developing cancer in 204 countries and territories by sex in 1990, 2019 and 2021	19](#_Toc22789)

[Table S8. Lifetime risk of dying from cancer in 204 countries and territories by sex in 1990, 2019 and 2021	34](#_Toc120)

[Table S9. Lifetime risks of developing and dying from different cancer types globally by sex in 2021	49](#_Toc13484)

[Table S10. Lifetime risks of developing and dying from different cancer types by SDI quintiles for males in 2021.	51](#_Toc7192)

[Table S11. Lifetime risks of developing and dying from different cancer types by SDI quintiles for females in 2021.	57](#_Toc3754)

[Table S12. Types of cancer with the highest lifetime incidence risk across 204 countries and territories by sex in 2021	64](#_Toc23969)

[Table S13. Types of cancer with the highest lifetime death risk across 204 countries and territories by sex in 2021	72](#_Toc22822)

[Table S14. Probability (%) of developing and dying from cancer from a cancer-free age to a specific age range for both sexes	80](#_Toc22535)

[Table S15. Probability (%) of developing and dying from cancer from a cancer-free age to a specific age range for males in 2021	82](#_Toc18182)

[Table S16. Probability (%) of developing and dying from cancer from a cancer-free age to a specific age range for females in 2021	84](#_Toc14162)

[Table S17. Lifetime incidence and death risk of specific cancer type by sex in 1990 and 2021	86](#_Toc28164)

[Table S18. Average annual percent change (AAPC) in lifetime risk of developing cancer by sex globally and by SDI quintile, 1990-2021, 1990-2019 and 2019-2021	88](#_Toc13536)

[Table S19. Average annual percent change (AAPC) in lifetime risk of dying from cancer by sex globally and by SDI quintile, 1990-2021, 1990-2019 and 2019-2021	88](#_Toc23140)

[Table S20. Average annual percent change (AAPC) in lifetime risk of developing cancer by GBD region and sex, 1990-2021, 1990-2019 and 2019-2021	90](#_Toc2747)

[Table S21. Average annual percent change (AAPC) in lifetime risk of dying from cancer by GBD region and sex, 1990-2021, 1990-2019 and 2019-2021	92](#_Toc10625)

[Table S22. Average annual percent change (AAPC) in lifetime risk of developing cancer in 204 countries and territories by sex, 1990-2021, 1990-2019 and 2019-2021	94](#_Toc18047)

[Table S23. Average annual percent change (AAPC) in lifetime risk of dying from cancer in 204 countries and territories by sex, 1990-2021 and 2019-2021	111](#_Toc10627)

[Table S24. Average annual percent change (AAPC) in lifetime risks of developing and dying from specific cancer by sex globally, 1990-2021, 1990-2019 and 2019-2021.	128](#_Toc8366)

[Figure S1. Lifetime risk of developing and dying from cancer at the national level in 2021 for males	133](#_Toc12282)

[Figure S2. Lifetime risk of developing and dying from cancer at the national level in 2021 for females	134](#_Toc25440)

[Figure S3. Global lifetime risks of developing and dying from cancer for males by age at diagnosis in 2021	135](#_Toc9788)

[Figure S4. Global lifetime risks of developing and dying from cancer for females by age at diagnosis in 2021	136](#_Toc944)

[Figure S5. Correlation between lifetime risk of developing and dying from cancer at national and regional SDI for male	137](#_Toc430)

[Figure S7. Lifetime death risks of different cancer types attributable to risk factors globally and by GBD region in 2021	139](#_Toc27479)

[Figure S8. Correlation between the lifetime risks of developing or dying from cancer and the cumulative risks during 0-74 years	140](#_Toc20324)

[Figure S9. The Distribution of lifetime incidence and death risk for the 10 Most Common Cancers in 2021 across SDI quintiles for males	141](#_Toc7248)

[Figure S10. The Distribution of lifetime incidence and death43 33 risk for the 10 Most Common Cancers in 2021 across SDI quintiles for females	142](#_Toc30146)

[Figure S11. Average annual percent changes (AAPCs) in lifetime risk of developing and dying from cancer at the national level for males, 1990-2021, 1990-2019 and 2019-2021](#_Toc26241)	[143](#_Toc9204)

[Figure S12. Average annual percent change (AAPC) in lifetime risk of developing and dying from cancer at the national level for females, 1990-2021, 1990-2019 and 2019-2021	144](#_Toc26551)

[Figure S13. Average annual percent changes (AAPCs) in lifetime risks of developing and dying from cancer for males, 1990-2021](#_Toc26248)	[145](#_Toc20839)

[Figure S14. Average annual percent changes (AAPCs) in lifetime risks of developing and dying from cancer for females, 1990-2021](#_Toc31873)	[146](#_Toc9168)

[Figure S15. Joinpoint regression analysis of the lifetime incidence and death risk globally by sex and for both sexes by SDI quintile	147](#_Toc8548)

[Figure S16. Joinpoint regression analysis of the lifetime incidence and death risk of cancer for males by SDI quintile, 1990-2021	148](#_Toc20939)

[Figure S17. Joinpoint regression analysis of the lifetime incidence and death risk of cancer for females by SDI quintile, 1990-2021	149](#_Toc31135)

[Figure S18. Joinpoint regression analysis of the lifetime incidence and death risk for selected cancer types globally among males, 1990-2021	150](#_Toc31160)

[Figure S19. Joinpoint regression analysis of the lifetime incidence and death risk for selected cancer types globally among females, 1990-2021	151](#_Toc27293)

Appendix 1. A brief introduction about the methods for lifetime risk of developing or dying from cancer

The lifetime risk of developing cancer (e.g. lifetime incidence risk) represents the probability of being diagnosed with cancer at some point during their life. Similarly, the lifetime risk of dying from cancer (e.g. lifetime death risk) denotes the probability of dying from cancer at some point during their life. This study utilizes the Adjusted for Multiple Primaries (AMP) method for such risk calculations, which is based on the life table method and adjusts for the proportion of multiple primary cancers in the population.

A detailed description of the AMP method has been previously published^1,2,3,4^. Here we give a brief introduction:

Basic assumptions:

(1) Individuals who have never been diagnosed with cancer have the same probability of developing a primary cancer as the general population.
(2) The risk of dying from causes other than cancer is equivalent between individuals without a cancer history and the general population.
(3) The age-specific rates observed in given year (including cancer incidence, cancer mortality, and all-cause mortality) are assumed to remain constant over time as a cohort ages forward.

Using all-cause mortality and cancer incidence, mortality, we calculated the lifetime risk by five-year age groups by the following formula:

$$P_{AMP}=\int_{0}^{\infty} \lambda_{c}\left( a \right)S_{0}\left( a \right)\mathrm{da}=\sum_{i=1}^{f} \frac{R_{i}}{R_{i}+M_{i}-D_{i}}\hat{S}_{0}^{*}\left( a_{i} \right)\times\left( 1-exp\left( -\frac{w_{i}}{N_{i}}\left( R_{i}+M_{i}-D_{i} \right) \right) \right)$$

where for age group i:

P_AMP_ denotes the probability of developing or dying from cancer

M_i_ denotes the annual number of deaths (all-cause mortality)

D_i_ denotes the annual number of cancer deaths (cancer mortality)

When estimating the risk of developing cancer, R_i_ denotes the annual number of cancer cases (cancer incidence); When estimating the risk of dying from cancer, R_i_ denotes the annual number of cancer cases (cancer mortality)

N_i_ denotes the population

W_i_ denotes the width of the i^th^ age group, the i^th^ interval is from a_i_ to a_i+1_, w_i_=(a_i+1_-a_i_)

λ_c_ denotes the cancer incidence rate, for each age group can be calculated as:

$$\lambda_{c,i} = \frac{R_{i}}{N_{i}}$$

$\hat{S}_{0}^{*}\left( a_{i} \right)$ denotes the probability of being alive and cancer free at age i, which can be approximately estimated as follows:

$$\hat{S}_{0}^{*}\left( a_{i} \right)=exp (-\sum_{j=1}^{i-1} \frac{R_{j}{+ (M}_{j}-D_{j})}{N_{j}})$$

Empirically, cancer incidence and mortality data are typically organized in frequency tables for statistical analyses, wherein the uppermost age category predominantly encompasses individuals aged 85 years and above. In this study, for the final age band (95+), the integral for the final age band is evaluated as follows:

$$\frac{R_{f}}{R_{f}+M_{f}-D_{f}}S_{0}\left( a_{f} \right)$$

The variance results are presented here, while detailed derivations can be found in References 3 and 4.

$$E\left( S_{x} \right)=\left( 1-\exp\left( -\frac{w_{i}}{N_{i}}\left( R_{i}+M_{i}-D_{i} \right) \right) \right),(Bionmial \& Poission)$$

$$Var\left( S_{x} \right)={(S_{x}^{'})}^{2}\times{w_{i}}^{2}\frac{p_{xi}}{N_{i}}, (Poission)$$

where:

$$S_{x}^{'} = \exp\left( -\frac{w_{i}}{N_{i}}\left( R_{i}+M_{i}-D_{i} \right) \right); P_{xi} = \frac{R_{i}}{R_{i}+M_{i}-D_{i}}$$

Reference:

1.	Sasieni PD, Shelton J, Ormiston-Smith N, Thomson CS, Silcocks PB. What is the lifetime risk of developing cancer?: the effect of adjusting for multiple primaries. Br J Cancer. Jul 26 2011;105(3):460-5. doi:10.1038/bjc.2011.250

2.	Ahmad AS, Ormiston-Smith N, Sasieni PD. Trends in the lifetime risk of developing cancer in Great Britain: comparison of risk for those born from 1930 to 1960. Br J Cancer. Mar 3 2015;112(5):943-7. doi:10.1038/bjc.2014.606

3.	Zheng R, Wang S, Zhang S, et al. Global, regional, and national lifetime probabilities of developing cancer in 2020. Sci Bull (Beijing). Nov 15 2023;68(21):2620-2628. doi:10.1016/j.scib.2023.09.041

4.	Wang S, Zheng R, Li J, et al. Global, regional, and national lifetime risks of developing and dying from gastrointestinal cancers in 185 countries: a population-based systematic analysis of GLOBOCAN. Lancet Gastroenterol Hepatol. Mar 2024;9(3):229-237. doi:10.1016/S2468-1253(23)00366-7

Table S1. International Classification of Diseases (ICD) codes of 32 GBD cancer causes used in lifetime risk analysis.

| **Cancer type** | **ICD-10** |
| --- | --- |
| **Lip and oral cavity cancer** | C00-C08.9 |
| **Nasopharynx cancer** | C11-C11.9 |
| **Other pharynx cancer** | C09-C10.9, C12-C13.9 |
| **Esophageal cancer** | C15-C15.9 |
| **Stomach cancer** | C16-C16.9 |
| **Colon and rectum cancer** | C18-C21.9 |
| **Liver cancer** | C22-C22.8 |
| **Gallbladder and biliary tract cancer** | C23-C24.9 |
| **Pancreatic cancer** | C25-C25.9 |
| **Larynx cancer** | C32-C32.9 |
| **Tracheal, bronchus, and lung cancer** | C33-C34.9 |
| **Malignant skin melanoma** | C43-C43.9 |
| **Soft tissue and other extraosseous sarcomas** | C49-C49.9 |
| **Malignant neoplasm of bone and articular cartilage** | C40-C41.9 |
| **Breast cancer** | C50-C50.9 |
| **Cervical cancer** | C53-C53.9 |
| **Uterine cancer** | C54-C54.9 |
| **Ovarian cancer** | C56-C56.9 |
| **Prostate cancer** | C61-C61.9 |
| **Testicular cancer** | C62-C62.9 |
| **Kidney cancer** | C64-C65.9 |
| **Bladder cancer** | C67-C67.9 |
| **Brain and central nervous system cancer** | C70-C72.9, C75.1-C75.3 |
| **Eye cancer** | C69.0-C69.8 |
| **Neuroblastoma and other peripheral nervous cell tumors** | C47-C47.9 |
| **Thyroid cancer** | C73-73.9 |
| **Mesothelioma** | C45-C45.9 |
| **Hodgkin lymphoma** | C81-C81.9 |
| **Non-Hodgkin lymphoma** | C82-C82.9, C83.0-C83.8, C84-C85.0, C85.2-C85.8, C86-C86.6, C96-C96.9 |
| **Multiple myeloma** | C88-C90.9 |
| **Leukemia** | C91-C91.0, C91.2-C91.3, C91.6, C92-C92.6, C93-C93.1, C93.3, C93.8, C94-94.5, C94.7-C95.9 |
| **Other malignant neoplasms** | C17-C17.9, C30-C31.9, C37-C38.8, C48-C48.9, C4A, C51-C52.9, C57-C57.8, C60-C60.9, C63-C63.8, C66-C66.9, C68.0-C68.8, C75-C75.0, C75.4-C75.8 |

Table S2. Initial age groups by cancer type for lifetime risk calculation.

| **Cancer type** | **Initial age group** |
| --- | --- |
| **Soft tissue and other extra osseous sarcomas** | <5 |
| **Malignant neoplasm of bone and articular cartilage** |  |
| **Liver cancer** |  |
| **Kidney cancer** |  |
| **Hodgkin lymphoma** |  |
| **Leukemia** |  |
| **Brain and central nervous system cancer** |  |
| **Non-Hodgkin lymphoma** |  |
| **Eye cancer** |  |
| **Neuroblastoma and other peripheral nervous cell tumors** |  |
| **Other malignant neoplasms** |  |
| **Nasopharynx cancer** | 5 to 9 |
| **Thyroid cancer** |  |
| **Cervical cancer** | 15 to 19 |
| **Colon and rectum cancer** |  |
| **Ovarian cancer** |  |
| **Bladder cancer** |  |
| **Breast cancer** |  |
| **Lip and oral cavity cancer** |  |
| **Stomach cancer** |  |
| **Malignant skin melanoma** |  |
| **Testicular cancer** |  |
| **Tracheal, bronchus, and lung cancer** |  |
| **Pancreatic cancer** |  |
| **Esophageal cancer** | 20 to 24 |
| **Larynx cancer** |  |
| **Gallbladder and biliary tract cancer** |  |
| **Prostate cancer** |  |
| **Uterine cancer** |  |
| **Other pharynx cancer** |  |
| **Mesothelioma** |  |
| **Multiple myeloma** |  |

Table S3. Lifetime risk of developing cancer by sex globally and by SDI quintile in 1990, 2019 and 2021.

| **Location** | **Male** | | | **Female** | | | **Both sexes** | | |
| --- | --- | --- | --- | --- | --- | --- | --- | --- | --- |
|  | **1990** | **2019** | **2021** | **1990** | **2019** | **2021** | **1990** | **2019** | **2021** |
| **Global** | 19.96  (19.87 to 20.04) | 25.12  (25.01 to 25.22) | 22.15  (22.07 to 22.23) | 18.16  (18.01 to 18.31) | 22.47  (22.28 to 22.66) | 21.03  (20.88 to 21.19) | 19.04  (18.97 to 19.12) | 23.84  (23.75 to 23.94) | 21.70  (21.63 to 21.78) |
| **High SDI** | 38.19  (37.61 to 38.76) | 44.24  (43.45 to 45.03) | 41.50  (40.82 to 42.18) | 33.47  (32.40 to 34.54) | 38.88  (37.63 to 40.13) | 37.14  (36.00 to 38.28) | 35.69  (35.15 to 36.23) | 41.52  (40.83 to 42.22) | 39.36  (38.76 to 39.97) |
| **High-middle SDI** | 25.22  (24.92 to 25.52) | 32.54  (32.06 to 33.02) | 30.18  (29.75 to 30.60) | 20.27  (19.72 to 20.82) | 26.43  (25.57 to 27.29) | 25.20  (24.44 to 25.97) | 22.63  (22.36 to 22.90) | 29.48  (29.05 to 29.92) | 27.73  (27.35 to 28.12) |
| **Middle SDI** | 15.07  (14.94 to 15.20) | 20.62  (20.44 to 20.79) | 18.73  (18.60 to 18.87) | 13.40  (13.17 to 13.62) | 17.72  (17.37 to 18.07) | 17.09  (16.80 to 17.38) | 14.26  (14.14 to 14.37) | 19.29  (19.13 to 19.46) | 18.07  (17.94 to 18.21) |
| **Low-middle SDI** | 6.72  (6.67 to 6.76) | 10.17  (10.10 to 10.24) | 8.60  (8.55 to 8.64) | 7.57  (7.46 to 7.67) | 11.34  (11.16 to 11.52) | 10.68  (10.54 to 10.83) | 7.15  (7.10 to 7.19) | 10.77  (10.69 to 10.84) | 9.66  (9.60 to 9.72) |
| **Low SDI** | 5.60  (5.56 to 5.64) | 8.20  (8.13 to 8.26) | 6.47  (6.44 to 6.51) | 7.37  (7.26 to 7.48) | 10.27  (10.10 to 10.45) | 9.48  (9.35 to 9.62) | 6.47  (6.42 to 6.51) | 9.23  (9.15 to 9.31) | 7.95  (7.90 to 8.00) |

Table S4. Lifetime risk of dying from cancer by sex globally and by SDI quintile in 1990, 2019 and 2021.

| **Location** | **Male** | | | **Female** | | | **Both** | | |
| --- | --- | --- | --- | --- | --- | --- | --- | --- | --- |
|  | **1990** | **2019** | **2021** | **1990** | **2019** | **2021** | **1990** | **2019** | **2021** |
| **Global** | 15.98  (15.92 to 16.04) | 17.80  (17.75 to 17.86) | 15.19  (15.16 to 15.23) | 13.01  (12.93 to 13.09) | 14.36  (14.28 to 14.45) | 13.06  (13.00 to 13.12) | 14.50  (14.46 to 14.55) | 16.12  (16.07 to 16.17) | 14.23  (14.19 to 14.26) |
| **High SDI** | 26.24  (25.94 to 26.54) | 27.38  (27.08 to 27.69) | 25.03  (24.78 to 25.27) | 20.57  (20.07 to 21.07) | 21.95  (21.48 to 22.43) | 20.56  (20.17 to 20.95) | 23.23  (22.98 to 23.48) | 24.51  (24.26 to 24.77) | 22.73  (22.53 to 22.94) |
| **High-middle SDI** | 21.80  (21.58 to 22.02) | 23.60  (23.37 to 23.84) | 21.33  (21.14 to 21.52) | 15.15  (14.84 to 15.45) | 16.39  (16.04 to 16.75) | 15.17  (14.89 to 15.45) | 18.40  (18.22 to 18.57) | 19.98  (19.79 to 20.17) | 18.29  (18.14 to 18.44) |
| **Middle SDI** | 14.41  (14.30 to 14.52) | 16.50  (16.40 to 16.61) | 14.48  (14.40 to 14.55) | 11.59  (11.44 to 11.73) | 12.55  (12.39 to 12.70) | 11.66  (11.54 to 11.77) | 13.06  (12.97 to 13.14) | 14.66  (14.58 to 14.75) | 13.24  (13.18 to 13.30) |
| **Low-middle SDI** | 6.49  (6.45 to 6.53) | 9.15  (9.10 to 9.20) | 7.40  (7.37 to 7.43) | 6.43  (6.37 to 6.50) | 8.87  (8.78 to 8.97) | 8.03  (7.96 to 8.10) | 6.49  (6.45 to 6.52) | 9.03  (8.98 to 9.08) | 7.75  (7.71 to 7.78) |
| **Low SDI** | 5.50  (5.46 to 5.54) | 7.88  (7.83 to 7.94) | 5.93  (5.90 to 5.95) | 6.41  (6.33 to 6.48) | 8.69  (8.59 to 8.80) | 7.70  (7.62 to 7.77) | 5.95  (5.91 to 5.99) | 8.29  (8.24 to 8.35) | 6.80  (6.76 to 6.83) |

Table S5. Lifetime risk of developing cancer by GBD region and sex in 1990, 2019 and 2021.

| **Location** | **Male** | | | **Female** | | | **Both** | | |
| --- | --- | --- | --- | --- | --- | --- | --- | --- | --- |
|  | **1990** | **2019** | **2021** | **1990** | **2019** | **2021** | **1990** | **2019** | **2021** |
| **Andean**  **Latin America** | 14.87 (13.98 to 15.76) | 22.68 (21.18 to 24.19) | 14.31 (13.70 to 14.92) | 17.34 (15.17 to 19.50) | 25.18 (21.98 to 28.38) | 19.96 (18.13 to 21.79) | 16.11 (15.13 to 17.10) | 23.96 (22.38 to 25.53) | 17.14 (16.40 to 17.87) |
| **Australasia** | 41.91 (37.15 to 46.66) | 51.62 (44.97 to 58.27) | 50.87 (44.39 to 57.36) | 35.69 (27.30 to 44.09) | 43.07 (34.11 to 52.03) | 41.89 (33.10 to 50.67) | 38.84 (34.43 to 43.26) | 47.54 (42.12 to 52.97) | 46.64 (41.34 to 51.95) |
| **Caribbean** | 19.95 (18.89 to 21.02) | 27.00 (25.73 to 28.27) | 21.77 (21.02 to 22.53) | 18.71 (16.68 to 20.75) | 24.02 (21.72 to 26.32) | 21.55 (19.82 to 23.28) | 19.47 (18.43 to 20.51) | 25.67 (24.46 to 26.88) | 21.95(21.14 to 22.76) |
| **Central Asia** | 16.91 (16.17 to 17.65) | 14.13 (13.51 to 14.74) | 11.86 (11.43 to 12.28) | 15.33 (13.65 to 17.00) | 14.12 (12.50 to 15.73) | 12.91 (11.70 to 14.11) | 15.92 (15.18 to 16.65) | 14.07 (13.40 to 14.73) | 12.39(11.91 to 12.87) |
| **Central Europe** | 22.99 (22.26 to 23.73) | 33.99 (32.28 to 35.71) | 29.40 (28.07 to 30.73) | 20.99 (19.16 to 22.81) | 29.84 (26.48 to 33.20) | 27.48 (24.79 to 30.17) | 21.99 (21.24 to 22.75) | 31.81 (30.19 to 33.44) | 28.49 (27.22 to 29.76) |
| **Central**  **Latin America** | 14.65 (14.24 to 15.06) | 20.13 (19.60 to 20.67) | 16.08 (15.77 to 16.39) | 17.59 (16.30 to 18.88) | 20.49 (19.11 to 21.87) | 18.45 (17.50 to 19.40) | 16.15 (15.65 to 16.65) | 20.43 (19.84 to 21.01) | 17.48 (17.11 to 17.85) |
| **Central**  **Sub-Saharan Africa** | 5.62 (5.51 to 5.72) | 7.90 (7.76 to 8.03) | 6.12 (6.05 to 6.19) | 7.38 (7.06 to 7.69) | 10.07 (9.67 to 10.46) | 9.36 (9.05 to 9.67) | 6.59(6.45 to 6.72) | 8.93 (8.77 to 9.09) | 7.71 (7.61 to 7.82) |
| **East Asia** | 20.97 (20.70 to 21.23) | 30.16 (29.69 to 30.64) | 30.55 (30.06 to 31.04) | 16.09 (15.74 to 16.44) | 22.98 (22.16 to 23.79) | 23.46 (22.62 to 24.30) | 18.62 (18.41 to 18.84) | 26.91 (26.49 to 27.33) | 27.32 (26.89 to 27.75) |
| **Eastern Europe** | 23.17 (22.70 to 23.63) | 25.82 (25.22 to 26.42) | 21.52 (21.06 to 21.97) | 20.17 (18.95 to 21.40) | 24.49 (22.99 to 25.99) | 22.43 (21.29 to 23.58) | 21.20 (20.73 to 21.67) | 24.96 (24.34 to 25.58) | 21.92 (21.45 to 22.39) |
| **Eastern**  **Sub-Saharan Africa** | 5.88 (5.82 to 5.95) | 9.49 (9.37 to 9.62) | 7.03 (6.97 to 7.10) | 8.99 (8.80 to 9.19) | 13.04 (12.69 to 13.39) | 11.56 (11.31 to 11.80) | 7.39 (7.31 to 7.47) | 11.25 (11.10 to 11.40) | 9.22 (9.13 to 9.31) |
| **High-income**  **Asia Pacific** | 38.51 (37.05 to 39.98) | 47.47 (44.98 to 49.96) | 47.15 (44.66 to 49.64) | 28.16 (25.86 to 30.46) | 37.30 (33.52 to 41.08) | 36.75 (32.96 to 40.55) | 33.20 (31.94 to 34.46) | 42.34 (40.16 to 44.52) | 41.97 (39.79 to 44.16) |
| **High-income North America** | 43.75 (42.62 to 44.88) | 43.26 (42.13 to 44.38) | 39.57 (38.67 to 40.47) | 40.78 (38.69 to 42.86) | 40.83 (39.01 to 42.66) | 38.75 (37.17 to 40.33) | 42.24 (41.17 to 43.31) | 42.06 (41.06 to 43.06) | 39.25 (38.42 to 40.07) |
| **North Africa and Middle East** | 11.33 (11.12 to 11.54) | 17.29 (16.98 to 17.61) | 14.24 (14.05 to 14.43) | 8.79 (8.47 to 9.11) | 15.60 (14.97 to 16.22) | 14.44 (13.97 to 14.91) | 10.19 (10.02 to 10.37) | 16.50 (16.20 to 16.81) | 14.42 (14.21 to 14.62) |
| **Oceania** | 7.40 (6.86 to 7.94) | 8.83 (8.30 to 9.35) | 6.99 (6.68 to 7.29) | 9.84 (7.88 to 11.81) | 11.00 (9.24 to 12.75) | 10.12 (8.77 to 11.46) | 8.58 (7.84 to 9.33) | 9.90 (9.20 to 10.59) | 8.52 (8.05 to 8.99) |
| **South Asia** | 6.09 (6.05 to 6.14) | 9.07 (9.00 to 9.14) | 7.90 (7.85 to 7.95) | 6.84 (6.75 to 6.94) | 10.15 (9.97 to 10.33) | 9.69 (9.53 to 9.84) | 6.46 (6.42 to 6.51) | 9.60 (9.53 to 9.68) | 8.79 (8.73 to 8.85) |
| **Southeast Asia** | 9.63 (9.51 to 9.75) | 14.06 (13.88 to 14.24) | 12.59 (12.46 to 12.72) | 10.41 (10.10 to 10.71) | 15.05 (14.54 to 15.55) | 14.40 (13.98 to 14.83) | 10.03 (9.90 to 10.17) | 14.54 (14.33 to 14.75) | 13.52 (13.35 to 13.68) |
| **Southern**  **Latin America** | 24.07 (22.66 to 25.48) | 30.03 (27.87 to 32.19) | 25.90 (24.21 to 27.59) | 23.17 (20.02 to 26.31) | 27.29 (23.43 to 31.14) | 24.58 (21.32 to 27.84) | 23.68 (22.24 to 25.13) | 28.59 (26.57 to 30.61) | 25.26 (23.62 to 26.89) |
| **Southern**  **Sub-Saharan Africa** | 12.07 (11.63 to 12.51) | 13.90 (13.58 to 14.22) | 9.11 (8.98 to 9.24) | 12.08 (11.20 to 12.95) | 15.18 (14.56 to 15.79) | 12.67 (12.28 to 13.06) | 12.09 (11.67 to 12.51) | 14.47 (14.16 to 14.77) | 10.91 (10.76 to 11.07) |
| **Tropical**  **Latin America** | 15.27 (14.87 to 15.68) | 21.42 (20.84 to 22.00) | 17.71 (17.32 to 18.11) | 15.54 (14.52 to 16.57) | 20.10 (18.67 to 21.52) | 18.50 (17.49 to 19.51) | 15.48 (15.06 to 15.90) | 20.75 (20.15 to 21.36) | 18.18 (17.76 to 18.60) |
| **Western Europe** | 37.96 (36.92 to 39.00) | 47.56 (45.73 to 49.38) | 44.56 (42.90 to 46.22) | 32.76 (30.93 to 34.58) | 40.41 (37.89 to 42.93) | 38.27 (35.89 to 40.65) | 35.14 (34.19 to 36.09) | 43.93 (42.42 to 45.44) | 41.45 (40.05 to 42.85) |
| **Western**  **Sub-Saharan Africa** | 5.54 (5.48 to 5.61) | 8.61 (8.52 to 8.70) | 6.80 (6.75 to 6.85) | 6.29 (6.12 to 6.46) | 9.55 (9.33 to 9.77) | 8.96 (8.79 to 9.14) | 5.84 (5.77 to 5.92) | 9.17 (9.07 to 9.28) | 7.99 (7.92 to 8.06) |

Table S6. Lifetime risk of dying from cancer by GBD region and sex in 1990, 2019 and 2021.

| **Location** | **Male** | | | **Female** | | | **Both** | | |
| --- | --- | --- | --- | --- | --- | --- | --- | --- | --- |
|  | **1990** | **2019** | **2021** | **1990** | **2019** | **2021** | **1990** | **2019** | **2021** |
| **Andean Latin America** | 14.78 (14.01 to 15.56) | 18.29 (17.43 to 19.16) | 10.03 (9.75 to 10.31) | 15.97 (14.44 to 17.50) | 19.40 (17.64 to 21.15) | 13.98 (13.19 to 14.78) | 15.39 (14.64 to 16.14) | 18.85 (18.01 to 19.69) | 11.95 (11.64 to 12.27) |
| **Australasia** | 26.91 (24.71 to 29.10) | 29.53 (26.89 to 32.16) | 28.93 (26.42 to 31.43) | 21.01 (16.80 to 25.21) | 22.50 (18.62 to 26.38) | 21.83 (18.11 to 25.54) | 23.83 (21.83 to 25.83) | 25.89 (23.73 to 28.04) | 25.31 (23.26 to 27.37) |
| **Caribbean** | 16.25 (15.54 to 16.97) | 18.70 (17.99 to 19.41) | 13.93 (13.56 to 14.30) | 13.56 (12.52 to 14.61) | 15.50 (14.43 to 16.58) | 13.27 (12.54 to 14.00) | 15.03 (14.44 to 15.62) | 17.19 (16.59 to 17.79) | 13.79 (13.44 to 14.15) |
| **Central Asia** | 16.06 (15.44 to 16.69) | 12.38 (11.96 to 12.80) | 10.15 (9.87 to 10.42) | 12.37 (11.37 to 13.37) | 10.26 (9.45 to 11.06) | 9.12 (8.57 to 9.67) | 14.02 (13.50 to 14.54) | 11.26 (10.89 to 11.64) | 9.65 (9.40 to 9.90) |
| **Central Europe** | 20.07 (19.57 to 20.57) | 25.52 (24.74 to 26.30) | 21.14 (20.62 to 21.67) | 15.73 (14.78 to 16.69) | 19.80 (18.23 to 21.37) | 17.57 (16.54 to 18.61) | 17.95 (17.51 to 18.39) | 22.55 (21.86 to 23.25) | 19.41 (18.94 to 19.88) |
| **Central Latin America** | 12.92 (12.61 to 13.23) | 13.61 (13.34 to 13.88) | 9.83 (9.69 to 9.96) | 14.44 (13.72 to 15.16) | 13.87 (13.26 to 14.48) | 11.53 (11.18 to 11.87) | 13.70 (13.39 to 14.01) | 13.78 (13.52 to 14.04) | 10.75 (10.61 to 10.89) |
| **Central Sub-Saharan Africa** | 5.60 (5.50 to 5.70) | 7.71 (7.59 to 7.82) | 5.72 (5.66 to 5.78) | 6.49 (6.26 to 6.71) | 8.43 (8.18 to 8.67) | 7.52 (7.34 to 7.69) | 6.14 (6.04 to 6.25) | 8.02 (7.91 to 8.14) | 6.61 (6.54 to 6.68) |
| **East Asia** | 20.13 (19.91 to 20.36) | 23.36 (23.10 to 23.63) | 23.37 (23.10 to 23.64) | 14.24 (14.00 to 14.47) | 15.53 (15.17 to 15.89) | 15.64 (15.28 to 16.01) | 17.32 (17.16 to 17.49) | 19.79 (19.58 to 19.99) | 19.83 (19.62 to 20.04) |
| **Eastern Europe** | 19.41 (19.09 to 19.73) | 17.39 (17.12 to 17.66) | 13.74 (13.56 to 13.92) | 13.65 (13.05 to 14.25) | 13.54 (13.02 to 14.07) | 11.66 (11.32 to 11.99) | 16.04 (15.78 to 16.31) | 15.24 (15.00 to 15.48) | 12.63 (12.48 to 12.79) |
| **Eastern Sub-Saharan Africa** | 5.70 (5.65 to 5.76) | 9.02 (8.92 to 9.12) | 6.31 (6.26 to 6.35) | 7.85 (7.71 to 7.99) | 11.23 (11.00 to 11.45) | 9.44 (9.30 to 9.58) | 6.74 (6.68 to 6.80) | 10.11 (10.01 to 10.22) | 7.79 (7.73 to 7.84) |
| **High-income Asia Pacific** | 28.34 (27.45 to 29.24) | 32.04 (31.01 to 33.07) | 31.77 (30.76 to 32.79) | 19.21 (17.98 to 20.44) | 22.84 (21.18 to 24.49) | 22.44 (20.82 to 24.06) | 23.57 (22.86 to 24.27) | 27.19 (26.32 to 28.06) | 26.91 (26.06 to 27.77) |
| **High-income North America** | 25.68 (25.18 to 26.18) | 23.01 (22.64 to 23.39) | 20.04 (19.77 to 20.30) | 21.60 (20.70 to 22.49) | 20.35 (19.76 to 20.95) | 18.63 (18.18 to 19.08) | 23.49 (23.05 to 23.94) | 21.58 (21.26 to 21.90) | 19.33 (19.09 to 19.56) |
| **North Africa and Middle East** | 10.51 (10.35 to 10.67) | 13.21 (13.04 to 13.38) | 10.22 (10.13 to 10.31) | 7.28 (7.10 to 7.46) | 9.73 (9.49 to 9.96) | 8.54 (8.38 to 8.69) | 9.05 (8.93 to 9.16) | 11.60 (11.47 to 11.73) | 9.53 (9.45 to 9.61) |
| **Oceania** | 7.25 (6.78 to 7.72) | 8.48 (8.05 to 8.92) | 6.48 (6.25 to 6.72) | 7.71 (6.66 to 8.75) | 8.61 (7.67 to 9.55) | 7.70 (7.01 to 8.38) | 7.51 (7.03 to 7.99) | 8.59 (8.16 to 9.03) | 7.12 (6.84 to 7.40) |
| **South Asia** | 5.89 (5.86 to 5.93) | 8.26 (8.21 to 8.31) | 6.89 (6.86 to 6.93) | 5.77 (5.71 to 5.83) | 7.95 (7.86 to 8.05) | 7.28 (7.21 to 7.35) | 5.86 (5.82 to 5.89) | 8.12 (8.08 to 8.17) | 7.11 (7.08 to 7.14) |
| **Southeast Asia** | 9.42 (9.31 to 9.52) | 12.36 (12.23 to 12.48) | 10.73 (10.65 to 10.82) | 8.79 (8.61 to 8.97) | 11.31 (11.07 to 11.56) | 10.51 (10.32 to 10.70) | 9.13 (9.04 to 9.22) | 11.84 (11.73 to 11.96) | 10.67 (10.58 to 10.75) |
| **Southern Latin America** | 21.85 (20.87 to 22.84) | 23.86 (22.77 to 24.96) | 19.74 (18.98 to 20.50) | 19.74 (17.82 to 21.66) | 20.46 (18.43 to 22.49) | 17.86 (16.34 to 19.37) | 20.87 (19.96 to 21.79) | 22.07 (21.07 to 23.06) | 18.80 (18.09 to 19.51) |
| **Southern Sub-Saharan Africa** | 11.84 (11.44 to 12.24) | 12.70 (12.44 to 12.96) | 7.67 (7.58 to 7.76) | 10.66 (10.06 to 11.25) | 12.25 (11.87 to 12.62) | 9.44 (9.24 to 9.64) | 11.28 (10.95 to 11.61) | 12.39 (12.18 to 12.61) | 8.59 (8.50 to 8.68) |
| **Tropical Latin America** | 14.38 (14.04 to 14.71) | 18.16 (17.77 to 18.55) | 14.29 (14.06 to 14.53) | 13.40 (12.77 to 14.02) | 15.75 (15.01 to 16.49) | 13.73 (13.27 to 14.20) | 13.98 (13.68 to 14.28) | 16.94 (16.59 to 17.29) | 14.08 (13.86 to 14.29) |
| **Western Europe** | 27.92 (27.39 to 28.44) | 30.03 (29.32 to 30.73) | 27.46 (26.88 to 28.04) | 21.12 (20.23 to 22.00) | 22.86 (21.74 to 23.97) | 21.37 (20.42 to 22.31) | 24.28 (23.84 to 24.73) | 26.21 (25.62 to 26.80) | 24.30 (23.81 to 24.80) |
| **Western Sub-Saharan Africa** | 5.58 (5.52 to 5.64) | 8.35 (8.27 to 8.43) | 6.27 (6.23 to 6.31) | 5.48 (5.37 to 5.59) | 8.04 (7.90 to 8.17) | 7.27 (7.17 to 7.36) | 5.49 (5.43 to 5.54) | 8.29 (8.22 to 8.36) | 6.88 (6.83 to 6.92) |

Table S7. Lifetime risk of developing cancer in 204 countries and territories by sex in 1990, 2019 and 2021.

| **Location** | **Male** | | | **Female** | | | **Both sexes** | | |
| --- | --- | --- | --- | --- | --- | --- | --- | --- | --- |
|  | **1990** | **2019** | **2021** | **1990** | **2019** | **2021** | **1990** | **2019** | **2021** |
| **Afghanistan** | 6.28 (5.98 to 6.57) | 8.30 (7.85 to 8.75) | 5.69 (5.51 to 5.87) | 9.27 (8.52 to 10.03) | 13.46 (12.28 to 14.63) | 12.07 (11.24 to 12.90) | 7.83 (7.45 to 8.21) | 11.01 (10.45 to 11.56) | 8.90 (8.59 to 9.21) |
| **Albania** | 18.35 (13.08 to 23.61) | 23.02 (12.43 to 33.61) | 19.31 (11.97 to 26.65) | 12.42 (2.92 to 21.91) | 16.78 (-0.52 to 34.08) | 15.05 (1.15 to 28.94) | 15.56 (10.77 to 20.35) | 20.08 (10.67 to 29.49) | 17.44 (10.56 to 24.31) |
| **Algeria** | 7.47 (6.85 to 8.09) | 9.82 (8.80 to 10.83) | 7.69 (7.14 to 8.25) | 7.21 (5.97 to 8.46) | 10.13 (8.12 to 12.14) | 9.33 (7.91 to 10.76) | 7.39 (6.76 to 8.02) | 9.99 (8.95 to 11.02) | 8.53 (7.91 to 9.16) |
| **American Samoa** | 16.08 (-4.15 to 36.31) | 18.41 (-2.83 to 39.66) | 18.55 (-2.78 to 39.89) | 15.68  (-47.50 to 78.86) | 19.94  (-40.92 to 80.79) | 20.02  (-42.20 to 82.24) | 16.01 (-6.60 to 38.63) | 19.16 (-5.93 to 44.26) | 19.28 (-6.13 to 44.69) |
| **Andorra** | 50.17  (-96.62 to 196.97) | 57.04  (-111.46 to 225.55) | 50.10  (-105.25 to 205.46) | 30.37  (-147.90 to 208.63) | 37.04  (-148.36 to 222.45) | 31.93  (-141.52 to 205.38) | 42.23  (-71.03 to 155.49) | 48.32  (-76.75 to 173.38) | 42.27  (-73.36 to 157.90) |
| **Angola** | 5.80 (5.56 to 6.05) | 9.56 (9.16 to 9.95) | 7.10 (6.91 to 7.29) | 6.78 (6.07 to 7.49) | 10.03 (9.20 to 10.87) | 9.05 (8.48 to 9.62) | 6.35 (6.06 to 6.64) | 9.87 (9.46 to 10.28) | 8.19 (7.95 to 8.43) |
| **Antigua**  **and Barbuda** | 26.02 (-6.51 to 58.55) | 34.25 (-8.12 to 76.61) | 30.19 (-1.59 to 61.98) | 20.39  (-60.90 to 101.69) | 29.83  (-54.56 to 114.22) | 26.97  (-47.67 to 101.60) | 23.58  (-13.14 to 60.29) | 31.97  (-11.06 to 75.01) | 29.00 (-7.30 to 65.30) |
| **Argentina** | 23.98 (22.19 to 25.76) | 28.27 (25.83 to 30.70) | 24.27 (22.36 to 26.17) | 22.59 (18.85 to 26.33) | 26.54 (22.04 to 31.05) | 23.74 (19.91 to 27.57) | 23.35 (21.56 to 25.13) | 27.36 (25.03 to 29.69) | 24.02 (22.13 to 25.92) |
| **Armenia** | 20.26 (15.47 to 25.05) | 24.94 (17.42 to 32.45) | 22.40 (16.46 to 28.33) | 19.06 (6.68 to 31.44) | 22.25 (4.34 to 40.16) | 20.60 (6.50 to 34.70) | 19.50 (14.28 to 24.72) | 23.51 (15.74 to 31.28) | 21.53 (15.57 to 27.50) |
| **Australia** | 42.27 (36.90 to 47.64) | 51.75 (44.33 to 59.17) | 50.93 (43.74 to 58.11) | 35.07 (25.67 to 44.46) | 42.81 (32.83 to 52.79) | 41.46 (31.73 to 51.19) | 38.74 (33.79 to 43.69) | 47.49 (41.45 to 53.53) | 46.49 (40.62 to 52.36) |
| **Austria** | 35.43 (29.00 to 41.86) | 42.82 (31.01 to 54.62) | 39.29 (28.52 to 50.07) | 32.44 (19.98 to 44.90) | 36.24 (18.78 to 53.69) | 33.75 (17.17 to 50.33) | 33.51 (27.38 to 39.64) | 39.44 (29.36 to 49.51) | 36.48 (27.19 to 45.77) |
| **Azerbaijan** | 14.76 (12.74 to 16.79) | 15.61 (13.11 to 18.12) | 12.29 (10.78 to 13.81) | 12.38 (7.61 to 17.14) | 13.29 (7.66 to 18.92) | 11.72 (8.10 to 15.35) | 13.39 (11.38 to 15.40) | 14.37 (11.87 to 16.87) | 12.04 (10.50 to 13.57) |
| **Bahamas** | 23.81 (7.49 to 40.13) | 28.94 (15.32 to 42.56) | 22.71 (13.85 to 31.58) | 22.25  (-12.31 to 56.81) | 25.92 (1.25 to 50.59) | 24.33 (-0.22 to 48.88) | 22.97 (6.66 to 39.29) | 27.58 (14.67 to 40.49) | 23.95 (13.63 to 34.27) |
| **Bahrain** | 17.35 (8.87 to 25.83) | 23.91 (12.36 to 35.46) | 19.81 (13.23 to 26.39) | 16.62 (-7.63 to 40.87) | 25.59 (-1.78 to 52.95) | 24.19 (3.06 to 45.31) | 16.87 (7.94 to 25.80) | 23.87 (12.33 to 35.41) | 21.03 (13.83 to 28.24) |
| **Bangladesh** | 6.78 (6.62 to 6.94) | 9.02 (8.70 to 9.34) | 8.15 (7.91 to 8.39) | 6.30 (5.99 to 6.61) | 8.47 (7.73 to 9.21) | 8.33 (7.68 to 8.98) | 6.67 (6.51 to 6.83) | 8.90 (8.54 to 9.25) | 8.39 (8.11 to 8.68) |
| **Barbados** | 26.94 (6.77 to 47.11) | 35.61 (9.26 to 61.97) | 32.85 (9.52 to 56.17) | 26.08  (-21.45 to 73.62) | 35.27  (-19.50 to 90.05) | 33.15  (-18.08 to 84.39) | 26.35 (4.10 to 48.61) | 35.45 (7.44 to 63.46) | 33.05 (7.31 to 58.80) |
| **Belarus** | 23.24 (21.03 to 25.44) | 29.21 (25.25 to 33.17) | 24.52 (21.58 to 27.46) | 18.89 (13.43 to 24.35) | 24.40 (15.24 to 33.56) | 22.46 (15.54 to 29.38) | 20.59 (18.44 to 22.73) | 26.66 (22.73 to 30.59) | 23.57 (20.64 to 26.51) |
| **Belgium** | 40.99 (34.39 to 47.59) | 44.21 (34.23 to 54.18) | 42.36 (32.68 to 52.04) | 35.16 (24.01 to 46.31) | 38.82 (24.61 to 53.02) | 37.07 (23.24 to 50.90) | 37.85 (31.86 to 43.84) | 41.37 (33.02 to 49.72) | 39.65 (31.50 to 47.80) |
| **Belize** | 13.41 (0.72 to 26.11) | 19.29 (7.67 to 30.91) | 16.12 (8.44 to 23.80) | 13.76  (-20.76 to 48.28) | 18.82 (-7.85 to 45.48) | 17.16 (-4.24 to 38.56) | 13.49 (-1.18 to 28.16) | 19.36 (7.19 to 31.53) | 16.94 (8.06 to 25.81) |
| **Benin** | 6.30 (5.70 to 6.90) | 8.55 (7.97 to 9.13) | 6.80 (6.47 to 7.13) | 6.69 (5.45 to 7.94) | 8.83 (7.47 to 10.20) | 8.20 (7.16 to 9.25) | 6.60 (5.97 to 7.22) | 8.72 (8.10 to 9.34) | 7.56 (7.15 to 7.97) |
| **Bermuda** | 35.66  (-13.96 to 85.28) | 52.63  (-54.17 to 159.42) | 47.11  (-39.30 to 133.52) | 33.99  (-87.34 to 155.31) | 39.79  (-149.67 to 229.25) | 37.15  (-128.33 to 202.64) | 35.19  (-17.97 to 88.35) | 47.04  (-53.95 to 148.03) | 43.08  (-41.59 to 127.74) |
| **Bhutan** | 6.34 (4.04 to 8.65) | 9.31 (4.52 to 14.10) | 9.25 (4.60 to 13.89) | 6.55 (2.60 to 10.50) | 9.84 (-1.01 to 20.69) | 9.92 (-0.77 to 20.61) | 6.37 (4.27 to 8.47) | 9.55 (4.58 to 14.51) | 9.56 (4.70 to 14.42) |
| **Bolivia (Plurinational State of)** | 13.92 (12.34 to 15.49) | 20.18 (17.73 to 22.63) | 11.18 (10.37 to 11.98) | 17.70 (14.15 to 21.26) | 23.84 (18.52 to 29.15) | 18.70 (15.88 to 21.52) | 15.83 (14.09 to 17.57) | 21.99 (19.33 to 24.65) | 14.83 (13.76 to 15.91) |
| **Bosnia**  **and Herzegovina** | 21.37 (16.90 to 25.84) | 31.13 (20.32 to 41.95) | 26.45 (18.51 to 34.39) | 16.36 (7.89 to 24.82) | 25.43 (6.46 to 44.40) | 22.60 (7.47 to 37.74) | 18.63 (14.69 to 22.56) | 28.14 (18.19 to 38.09) | 24.55 (17.09 to 32.01) |
| **Botswana** | 9.70 (8.14 to 11.27) | 12.90 (11.07 to 14.73) | 7.36 (6.80 to 7.93) | 10.70 (6.80 to 14.61) | 12.40 (9.73 to 15.08) | 9.47 (8.05 to 10.89) | 10.32 (8.62 to 12.01) | 12.55 (11.01 to 14.09) | 8.45 (7.83 to 9.08) |
| **Brazil** | 15.31 (14.90 to 15.72) | 21.44 (20.85 to 22.04) | 17.77 (17.37 to 18.17) | 15.57 (14.54 to 16.60) | 20.09 (18.65 to 21.54) | 18.52 (17.50 to 19.55) | 15.52 (15.09 to 15.94) | 20.76 (20.14 to 21.37) | 18.22 (17.79 to 18.64) |
| **Brunei Darussalam** | 22.02 (2.43 to 41.62) | 24.59 (2.46 to 46.72) | 23.06 (3.01 to 43.11) | 22.98  (-20.99 to 66.94) | 27.22  (-19.49 to 73.93) | 25.72  (-18.37 to 69.80) | 22.45 (2.23 to 42.68) | 25.66 (3.13 to 48.20) | 24.15 (3.58 to 44.72) |
| **Bulgaria** | 19.11 (16.77 to 21.46) | 28.96 (23.99 to 33.94) | 23.33 (19.87 to 26.80) | 19.62 (13.15 to 26.09) | 27.78 (16.58 to 38.99) | 25.08 (17.16 to 32.99) | 19.45 (16.85 to 22.06) | 28.40 (23.32 to 33.48) | 24.26 (20.74 to 27.79) |
| **Burkina Faso** | 5.75 (5.45 to 6.05) | 8.09 (7.72 to 8.47) | 6.46 (6.24 to 6.68) | 6.95 (6.31 to 7.59) | 9.90 (8.80 to 10.99) | 8.98 (8.15 to 9.81) | 6.41 (6.09 to 6.73) | 9.05 (8.59 to 9.51) | 7.74 (7.43 to 8.05) |
| **Burundi** | 5.79 (5.47 to 6.12) | 8.04 (7.51 to 8.58) | 6.66 (6.31 to 7.00) | 8.92 (7.89 to 9.95) | 11.01 (9.21 to 12.81) | 10.21 (8.78 to 11.63) | 7.41 (6.98 to 7.85) | 9.42 (8.73 to 10.11) | 8.27 (7.78 to 8.76) |
| **Cabo Verde** | 12.99 (5.56 to 20.42) | 23.62 (13.34 to 33.91) | 22.69 (13.52 to 31.85) | 13.91 (-3.04 to 30.86) | 18.29 (-4.39 to 40.97) | 17.81 (-3.17 to 38.79) | 13.60 (5.57 to 21.63) | 21.15 (11.38 to 30.93) | 20.40 (11.56 to 29.24) |
| **Cambodia** | 8.62 (8.05 to 9.18) | 13.43 (12.48 to 14.37) | 12.33 (11.57 to 13.08) | 8.92 (7.87 to 9.98) | 14.14 (11.62 to 16.66) | 13.85 (11.57 to 16.12) | 8.83 (8.27 to 9.39) | 13.74 (12.65 to 14.82) | 13.09 (12.16 to 14.01) |
| **Cameroon** | 7.13 (6.68 to 7.59) | 9.21 (8.85 to 9.57) | 6.88 (6.70 to 7.06) | 8.40 (7.33 to 9.46) | 9.94 (9.21 to 10.66) | 8.96 (8.40 to 9.52) | 7.80 (7.29 to 8.31) | 9.60 (9.24 to 9.96) | 7.95 (7.72 to 8.18) |
| **Canada** | 41.30 (36.98 to 45.62) | 45.98 (40.50 to 51.46) | 42.06 (37.16 to 46.95) | 38.44 (30.89 to 46.00) | 43.59 (35.97 to 51.21) | 40.85 (33.73 to 47.97) | 39.84 (35.84 to 43.84) | 44.73 (40.18 to 49.27) | 41.44 (37.30 to 45.58) |
| **Central**  **African Republic** | 4.95 (4.66 to 5.23) | 5.17 (4.94 to 5.39) | 4.14 (4.00 to 4.27) | 7.22 (6.19 to 8.25) | 7.72 (6.95 to 8.49) | 7.17 (6.52 to 7.81) | 6.10 (5.72 to 6.48) | 6.38 (6.09 to 6.68) | 5.56 (5.35 to 5.77) |
| **Chad** | 4.80 (4.46 to 5.15) | 7.02 (6.67 to 7.37) | 5.95 (5.71 to 6.18) | 5.33 (4.63 to 6.02) | 8.12 (7.26 to 8.97) | 7.75 (7.03 to 8.48) | 5.11 (4.76 to 5.47) | 7.61 (7.22 to 7.99) | 6.86 (6.56 to 7.15) |
| **Chile** | 21.78 (19.42 to 24.13) | 32.72 (27.69 to 37.74) | 27.84 (24.00 to 31.67) | 23.77 (17.41 to 30.12) | 28.07 (19.78 to 36.36) | 25.25 (18.46 to 32.04) | 22.81 (20.20 to 25.42) | 30.31 (25.84 to 34.77) | 26.56 (23.07 to 30.06) |
| **China** | 21.03 (20.76 to 21.30) | 30.19 (29.70 to 30.68) | 30.60 (30.09 to 31.10) | 16.11 (15.76 to 16.47) | 22.99 (22.15 to 23.83) | 23.50 (22.64 to 24.37) | 18.67 (18.46 to 18.89) | 26.94 (26.51 to 27.37) | 27.38 (26.94 to 27.83) |
| **Colombia** | 17.77 (16.50 to 19.03) | 24.87 (22.76 to 26.98) | 21.96 (20.67 to 23.25) | 20.46 (17.09 to 23.83) | 24.50 (19.99 to 29.02) | 22.58 (19.10 to 26.05) | 19.12 (17.75 to 20.49) | 24.66 (22.48 to 26.84) | 22.48 (20.98 to 23.98) |
| **Comoros** | 7.87 (5.37 to 10.37) | 11.31 (7.39 to 15.23) | 9.63 (6.91 to 12.35) | 11.83 (3.34 to 20.33) | 15.34 (3.25 to 27.43) | 14.71 (4.23 to 25.20) | 9.85 (6.28 to 13.43) | 13.34 (8.23 to 18.46) | 12.20 (8.16 to 16.23) |
| **Congo** | 7.41 (6.78 to 8.03) | 10.15 (9.26 to 11.03) | 8.01 (7.51 to 8.50) | 9.92 (8.19 to 11.66) | 12.82 (10.88 to 14.77) | 12.10 (10.44 to 13.77) | 8.72 (7.97 to 9.46) | 11.39 (10.42 to 12.35) | 10.02 (9.32 to 10.71) |
| **Cook Islands** | 17.56 (-8.99 to 44.12) | 27.00  (-28.20 to 82.20) | 27.58  (-30.82 to 85.99) | 14.24  (-56.78 to 85.26) | 19.44  (-128.38 to 167.25) | 19.72  (-131.96 to 171.40) | 16.41  (-12.85 to 45.67) | 24.21  (-38.26 to 86.67) | 24.61  (-40.93 to 90.15) |
| **Costa Rica** | 25.97 (17.47 to 34.47) | 35.54 (26.45 to 44.63) | 31.67 (24.81 to 38.52) | 23.49 (6.05 to 40.93) | 29.07 (12.71 to 45.43) | 27.90 (14.32 to 41.47) | 24.92 (16.38 to 33.46) | 32.65 (24.14 to 41.17) | 30.22 (23.52 to 36.93) |
| **Cote d’Ivoire** | 4.80 (4.62 to 4.98) | 7.11 (6.87 to 7.35) | 5.95 (5.79 to 6.10) | 5.76 (5.21 to 6.31) | 8.39 (7.64 to 9.14) | 8.04 (7.40 to 8.69) | 5.31 (5.10 to 5.52) | 7.73 (7.44 to 8.01) | 6.95 (6.74 to 7.16) |
| **Croatia** | 30.80 (25.33 to 36.28) | 43.33 (29.65 to 57.02) | 39.02 (27.40 to 50.64) | 26.69 (14.93 to 38.46) | 36.92 (13.68 to 60.16) | 34.64 (14.42 to 54.86) | 28.61 (23.30 to 33.92) | 39.93 (27.58 to 52.28) | 36.84 (26.20 to 47.48) |
| **Cuba** | 27.45 (23.89 to 31.02) | 39.09 (33.59 to 44.59) | 30.29 (26.82 to 33.77) | 24.84 (18.27 to 31.40) | 32.71 (22.82 to 42.61) | 28.43 (21.13 to 35.72) | 26.41 (22.97 to 29.84) | 36.25 (31.03 to 41.46) | 29.83 (26.37 to 33.30) |
| **Cyprus** | 24.78 (11.17 to 38.38) | 42.21 (14.53 to 69.88) | 39.32 (11.66 to 66.98) | 21.38 (-8.18 to 50.94) | 35.95 (-6.15 to 78.04) | 33.44 (-8.80 to 75.69) | 23.02 (8.98 to 37.06) | 39.26 (15.25 to 63.28) | 36.60 (12.87 to 60.33) |
| **Czechia** | 30.14 (26.54 to 33.75) | 40.45 (32.03 to 48.88) | 35.59 (28.85 to 42.32) | 27.12 (19.12 to 35.12) | 34.92 (21.15 to 48.69) | 32.21 (20.58 to 43.84) | 28.67 (25.11 to 32.24) | 37.56 (30.11 to 45.01) | 33.97 (27.89 to 40.05) |
| **Democratic People's Republic of Korea** | 16.06 (14.67 to 17.45) | 16.80 (15.51 to 18.08) | 16.75 (15.45 to 18.06) | 12.97 (10.86 to 15.07) | 15.01 (12.24 to 17.78) | 14.98 (12.14 to 17.82) | 14.16 (13.00 to 15.32) | 15.61 (14.37 to 16.86) | 15.59 (14.32 to 16.85) |
| **Democratic Republic of the Congo** | 5.34 (5.22 to 5.47) | 7.23 (7.09 to 7.38) | 5.67 (5.59 to 5.75) | 7.25 (6.87 to 7.64) | 9.92 (9.40 to 10.45) | 9.32 (8.90 to 9.74) | 6.43 (6.26 to 6.59) | 8.49 (8.30 to 8.69) | 7.42 (7.29 to 7.55) |
| **Denmark** | 32.06 (24.86 to 39.27) | 46.70 (31.74 to 61.66) | 45.16 (30.67 to 59.65) | 34.27 (19.86 to 48.69) | 43.10 (21.42 to 64.77) | 41.63 (20.40 to 62.86) | 33.09 (25.90 to 40.28) | 44.89 (32.17 to 57.60) | 43.42 (30.98 to 55.85) |
| **Djibouti** | 8.76 (5.42 to 12.11) | 11.82 (9.09 to 14.54) | 9.41 (7.83 to 11.00) | 11.75 (1.13 to 22.37) | 14.38 (7.86 to 20.90) | 13.24 (8.22 to 18.25) | 10.10 (6.03 to 14.18) | 12.95 (10.08 to 15.82) | 11.09 (9.19 to 13.00) |
| **Dominica** | 23.48 (-5.61 to 52.57) | 27.79 (-7.32 to 62.91) | 22.84 (-1.01 to 46.70) | 21.57  (-46.70 to 89.84) | 24.47  (-56.16 to 105.09) | 22.37  (-42.19 to 86.93) | 22.47 (-8.60 to 53.54) | 25.69  (-10.55 to 61.93) | 22.55 (-4.14 to 49.23) |
| **Dominican Republic** | 13.12 (11.23 to 15.01) | 17.11 (15.22 to 18.99) | 15.89 (14.40 to 17.39) | 12.56 (8.17 to 16.95) | 16.73 (11.98 to 21.49) | 15.99 (11.60 to 20.38) | 12.93 (10.96 to 14.91) | 17.01 (15.02 to 19.00) | 16.10 (14.38 to 17.82) |
| **Ecuador** | 14.36 (12.66 to 16.05) | 20.82 (18.32 to 23.32) | 15.44 (14.07 to 16.80) | 16.69 (12.21 to 21.17) | 23.25 (17.12 to 29.37) | 19.74 (15.55 to 23.93) | 15.52 (13.61 to 17.42) | 22.06 (19.31 to 24.80) | 17.63 (15.98 to 19.29) |
| **Egypt** | 7.11 (6.82 to 7.39) | 12.74 (12.24 to 13.23) | 10.70 (10.37 to 11.03) | 5.88 (5.39 to 6.37) | 13.57 (12.25 to 14.90) | 13.07 (12.05 to 14.09) | 6.53 (6.27 to 6.78) | 13.15 (12.59 to 13.71) | 11.83 (11.44 to 12.22) |
| **El Salvador** | 9.89 (8.60 to 11.18) | 18.51 (15.89 to 21.12) | 15.86 (14.01 to 17.72) | 14.08 (8.42 to 19.74) | 21.20 (12.78 to 29.62) | 19.87 (12.95 to 26.79) | 12.01 (10.19 to 13.83) | 20.21 (17.04 to 23.38) | 18.28 (15.82 to 20.74) |
| **Equatorial Guinea** | 5.27 (4.39 to 6.15) | 11.38 (8.87 to 13.89) | 8.48 (7.27 to 9.69) | 7.28 (4.11 to 10.46) | 12.15 (7.74 to 16.55) | 11.08 (7.85 to 14.30) | 6.37 (5.13 to 7.61) | 11.87 (9.46 to 14.28) | 9.92 (8.46 to 11.38) |
| **Eritrea** | 4.28 (4.02 to 4.54) | 8.84 (8.08 to 9.59) | 7.48 (6.97 to 8.00) | 9.63 (8.14 to 11.12) | 14.43 (11.62 to 17.25) | 13.65 (11.28 to 16.02) | 6.67 (6.21 to 7.13) | 11.67 (10.61 to 12.73) | 10.55 (9.75 to 11.36) |
| **Estonia** | 25.06 (18.92 to 31.19) | 44.24 (21.72 to 66.76) | 39.47 (20.77 to 58.18) | 23.66 (7.63 to 39.70) | 33.37 (-6.48 to 73.21) | 30.99 (-4.25 to 66.24) | 24.09 (17.89 to 30.29) | 38.30 (17.62 to 58.98) | 35.15 (17.87 to 52.43) |
| **Eswatini** | 10.63 (8.25 to 13.02) | 11.84 (10.19 to 13.50) | 7.18 (6.57 to 7.80) | 11.75 (5.04 to 18.46) | 13.22 (9.99 to 16.45) | 9.72 (8.14 to 11.30) | 11.28 (8.57 to 13.99) | 12.56 (10.96 to 14.16) | 8.44 (7.76 to 9.12) |
| **Ethiopia** | 5.19 (5.10 to 5.28) | 9.73 (9.39 to 10.07) | 7.07 (6.91 to 7.23) | 8.79 (8.49 to 9.10) | 13.07 (12.15 to 13.99) | 11.74 (11.10 to 12.37) | 6.84 (6.72 to 6.97) | 11.36 (10.96 to 11.77) | 9.24 (9.01 to 9.47) |
| **Fiji** | 7.10 (5.41 to 8.80) | 8.49 (6.38 to 10.61) | 6.95 (5.45 to 8.45) | 14.58 (3.27 to 25.88) | 15.50 (4.63 to 26.37) | 14.38 (5.53 to 23.23) | 10.70 (7.14 to 14.26) | 11.91 (8.10 to 15.72) | 10.51 (7.67 to 13.35) |
| **Finland** | 29.71 (24.66 to 34.76) | 42.55 (30.22 to 54.88) | 41.58 (29.63 to 53.53) | 29.28 (15.37 to 43.19) | 39.75 (18.12 to 61.38) | 38.55 (17.19 to 59.92) | 29.16 (23.63 to 34.70) | 41.07 (29.56 to 52.57) | 40.05 (28.87 to 51.22) |
| **France** | 43.00 (40.03 to 45.98) | 53.62 (48.66 to 58.58) | 50.33 (45.67 to 55.00) | 31.78 (27.10 to 36.47) | 43.34 (36.64 to 50.04) | 40.88 (34.47 to 47.30) | 37.40 (34.86 to 39.94) | 48.54 (44.49 to 52.58) | 45.73 (41.90 to 49.56) |
| **Gabon** | 9.41 (7.96 to 10.86) | 12.86 (10.88 to 14.84) | 10.32 (9.12 to 11.52) | 11.02 (6.22 to 15.82) | 13.70 (9.12 to 18.27) | 12.61 (8.94 to 16.28) | 10.30 (8.53 to 12.07) | 13.30 (11.17 to 15.42) | 11.54 (10.04 to 13.03) |
| **Gambia** | 5.28 (4.02 to 6.54) | 7.13 (5.91 to 8.35) | 5.98 (5.20 to 6.77) | 5.77 (3.19 to 8.35) | 8.17 (5.70 to 10.64) | 7.55 (5.62 to 9.49) | 5.51 (4.28 to 6.74) | 7.64 (6.42 to 8.86) | 6.75 (5.89 to 7.61) |
| **Georgia** | 16.54 (14.01 to 19.08) | 24.94 (19.55 to 30.34) | 20.97 (16.90 to 25.04) | 17.34 (9.33 to 25.35) | 23.20 (7.75 to 38.66) | 21.06 (9.48 to 32.64) | 16.79 (13.81 to 19.77) | 24.03 (18.21 to 29.85) | 21.07 (16.63 to 25.50) |
| **Germany** | 35.68 (33.68 to 37.67) | 45.67 (41.83 to 49.51) | 43.30 (39.75 to 46.86) | 32.67 (29.06 to 36.28) | 39.97 (34.38 to 45.56) | 38.08 (32.76 to 43.40) | 33.76 (31.92 to 35.59) | 42.78 (39.52 to 46.05) | 40.73 (37.68 to 43.78) |
| **Ghana** | 6.81 (6.47 to 7.15) | 8.78 (8.47 to 9.09) | 7.13 (6.94 to 7.33) | 7.71 (6.84 to 8.58) | 10.49 (9.65 to 11.34) | 9.81 (9.12 to 10.51) | 7.28 (6.89 to 7.68) | 9.73 (9.35 to 10.10) | 8.59 (8.32 to 8.87) |
| **Greece** | 38.25 (30.78 to 45.72) | 43.40 (32.59 to 54.20) | 41.00 (31.30 to 50.69) | 30.48 (18.10 to 42.86) | 35.25 (18.99 to 51.51) | 34.31 (19.20 to 49.42) | 34.39 (27.71 to 41.06) | 39.47 (30.30 to 48.64) | 37.88 (29.53 to 46.24) |
| **Greenland** | 27.86 (-6.32 to 62.04) | 31.31  (-13.82 to 76.44) | 29.27  (-11.86 to 70.41) | 31.22  (-40.30 to 102.75) | 30.66  (-64.22 to 125.54) | 29.25  (-58.74 to 117.23) | 28.97 (-2.82 to 60.76) | 31.07  (-13.00 to 75.14) | 29.33  (-11.46 to 70.12) |
| **Grenada** | 20.25 (-2.20 to 42.69) | 30.43 (-1.92 to 62.78) | 23.23 (3.92 to 42.55) | 20.01  (-36.14 to 76.16) | 26.71  (-45.19 to 98.60) | 23.82  (-31.17 to 78.82) | 20.07 (-5.20 to 45.34) | 27.81 (-6.41 to 62.04) | 23.47 (0.45 to 46.50) |
| **Guam** | 18.40 (-2.24 to 39.05) | 19.54 (-1.27 to 40.36) | 16.28 (0.41 to 32.14) | 15.01  (-25.20 to 55.21) | 16.29  (-26.31 to 58.89) | 13.13  (-19.92 to 46.18) | 16.60 (-1.37 to 34.56) | 17.91 (-2.18 to 38.01) | 14.80 (-0.20 to 29.79) |
| **Guatemala** | 7.43 (6.90 to 7.96) | 14.71 (13.39 to 16.04) | 10.60 (9.96 to 11.25) | 11.27 (9.33 to 13.22) | 16.99 (13.56 to 20.42) | 14.35 (11.95 to 16.76) | 9.28 (8.56 to 9.99) | 15.97 (14.50 to 17.43) | 12.64 (11.76 to 13.52) |
| **Guinea** | 6.22 (5.66 to 6.78) | 8.19 (7.65 to 8.74) | 6.33 (6.02 to 6.63) | 7.23 (6.15 to 8.31) | 9.78 (8.44 to 11.13) | 9.09 (8.01 to 10.16) | 6.74 (6.15 to 7.34) | 9.06 (8.41 to 9.71) | 7.77 (7.33 to 8.21) |
| **Guinea-Bissau** | 6.12 (5.21 to 7.03) | 8.21 (7.17 to 9.24) | 6.34 (5.76 to 6.92) | 7.10 (4.91 to 9.30) | 10.56 (7.73 to 13.39) | 9.59 (7.41 to 11.76) | 6.75 (5.72 to 7.79) | 9.44 (8.22 to 10.67) | 8.02 (7.19 to 8.84) |
| **Guyana** | 9.14 (7.39 to 10.88) | 13.08 (10.08 to 16.08) | 9.56 (7.87 to 11.24) | 12.53 (4.75 to 20.30) | 16.24 (5.64 to 26.84) | 14.45 (6.57 to 22.32) | 10.87 (8.33 to 13.40) | 14.72 (10.89 to 18.55) | 12.07 (9.53 to 14.61) |
| **Haiti** | 9.91 (9.21 to 10.61) | 13.42 (12.52 to 14.32) | 9.29 (8.88 to 9.69) | 12.07 (10.54 to 13.60) | 14.69 (12.90 to 16.48) | 12.99 (11.68 to 14.30) | 10.94 (10.18 to 11.71) | 14.12 (13.19 to 15.05) | 11.29 (10.72 to 11.85) |
| **Honduras** | 8.83 (7.58 to 10.08) | 12.75 (11.63 to 13.87) | 9.53 (8.93 to 10.12) | 12.29 (8.01 to 16.57) | 18.40 (14.37 to 22.43) | 16.56 (13.63 to 19.49) | 10.57 (8.86 to 12.28) | 15.73 (14.02 to 17.45) | 13.18 (12.09 to 14.27) |
| **Hungary** | 26.40 (23.81 to 28.99) | 36.21 (29.21 to 43.21) | 31.51 (26.02 to 37.00) | 24.76 (18.98 to 30.54) | 31.85 (20.10 to 43.60) | 29.57 (20.28 to 38.86) | 25.66 (23.12 to 28.20) | 33.83 (27.56 to 40.10) | 30.51 (25.60 to 35.41) |
| **Iceland** | 37.66  (-10.72 to 86.05) | 48.27  (-25.56 to 122.09) | 46.44  (-27.02 to 119.91) | 36.91  (-48.21 to 122.03) | 41.95  (-56.46 to 140.37) | 42.92  (-53.66 to 139.49) | 37.21 (-8.27 to 82.68) | 45.18  (-14.39 to 104.75) | 44.54  (-14.07 to 103.15) |
| **India** | 5.63 (5.59 to 5.67) | 8.74 (8.67 to 8.81) | 7.65 (7.59 to 7.70) | 6.58 (6.48 to 6.69) | 9.86 (9.67 to 10.06) | 9.41 (9.24 to 9.57) | 6.08 (6.04 to 6.13) | 9.28 (9.20 to 9.37) | 8.51 (8.45 to 8.58) |
| **Indonesia** | 7.14 (7.01 to 7.27) | 11.41 (11.20 to 11.62) | 9.96 (9.81 to 10.11) | 8.85 (8.51 to 9.19) | 13.31 (12.68 to 13.95) | 12.66 (12.13 to 13.19) | 7.99 (7.84 to 8.14) | 12.34 (12.07 to 12.61) | 11.31 (11.11 to 11.51) |
| **Iran**  **(Islamic Republic of)** | 11.75 (11.18 to 12.32) | 20.46 (19.05 to 21.86) | 14.88 (14.13 to 15.63) | 9.83 (8.73 to 10.92) | 18.28 (15.38 to 21.17) | 15.62 (13.65 to 17.60) | 10.87 (10.33 to 11.40) | 19.50 (18.11 to 20.89) | 15.39 (14.57 to 16.21) |
| **Iraq** | 9.57 (8.85 to 10.29) | 14.41 (13.43 to 15.39) | 12.53 (11.85 to 13.21) | 8.93 (7.22 to 10.63) | 15.81 (13.00 to 18.63) | 15.17 (12.90 to 17.44) | 9.32 (8.58 to 10.06) | 15.07 (13.92 to 16.22) | 13.84 (12.99 to 14.70) |
| **Ireland** | 32.60 (23.23 to 41.97) | 46.09 (28.34 to 63.84) | 41.51 (25.13 to 57.88) | 33.23 (14.07 to 52.40) | 42.61 (18.45 to 66.77) | 38.63 (15.55 to 61.72) | 32.95 (23.36 to 42.54) | 44.33 (29.63 to 59.03) | 40.10 (26.33 to 53.86) |
| **Israel** | 27.85 (19.25 to 36.46) | 38.01 (25.74 to 50.28) | 35.13 (24.79 to 45.48) | 29.18 (12.87 to 45.49) | 36.77 (18.95 to 54.59) | 33.92 (17.76 to 50.08) | 28.58 (19.98 to 37.18) | 37.29 (26.87 to 47.71) | 34.47 (25.41 to 43.54) |
| **Italy** | 41.03 (37.66 to 44.41) | 48.38 (42.38 to 54.39) | 45.77 (40.16 to 51.37) | 32.96 (27.49 to 38.42) | 40.86 (33.18 to 48.53) | 39.20 (31.82 to 46.57) | 36.90 (33.93 to 39.87) | 44.44 (39.65 to 49.23) | 42.42 (37.88 to 46.95) |
| **Jamaica** | 22.44 (15.30 to 29.58) | 31.88 (24.16 to 39.59) | 27.49 (21.98 to 33.01) | 19.84 (6.27 to 33.40) | 26.45 (12.59 to 40.31) | 24.93 (12.94 to 36.92) | 21.03 (13.70 to 28.36) | 29.27 (21.63 to 36.92) | 26.54 (20.52 to 32.56) |
| **Japan** | 40.70 (38.51 to 42.88) | 48.38 (45.22 to 51.54) | 48.29 (45.10 to 51.48) | 29.83 (26.62 to 33.04) | 39.09 (34.32 to 43.85) | 38.61 (33.80 to 43.42) | 35.12 (33.27 to 36.96) | 43.73 (40.97 to 46.50) | 43.51 (40.71 to 46.31) |
| **Jordan** | 12.55 (8.74 to 16.37) | 17.75 (12.66 to 22.84) | 14.01 (11.48 to 16.53) | 11.59 (5.22 to 17.96) | 18.91 (9.15 to 28.67) | 17.43 (11.01 to 23.85) | 11.89 (8.47 to 15.31) | 18.22 (13.35 to 23.08) | 15.54 (12.85 to 18.23) |
| **Kazakhstan** | 23.00 (21.14 to 24.87) | 15.00 (13.80 to 16.20) | 11.83 (11.09 to 12.58) | 19.74 (15.66 to 23.82) | 15.30 (11.38 to 19.21) | 13.60 (11.00 to 16.20) | 21.03 (19.27 to 22.80) | 15.07 (13.65 to 16.50) | 12.73 (11.81 to 13.65) |
| **Kenya** | 5.03 (4.79 to 5.27) | 7.88 (7.63 to 8.14) | 6.06 (5.92 to 6.20) | 7.98 (7.24 to 8.72) | 12.12 (11.24 to 12.99) | 10.86 (10.21 to 11.51) | 6.48 (6.17 to 6.79) | 9.95 (9.61 to 10.29) | 8.31 (8.10 to 8.53) |
| **Kiribati** | 7.06 (3.03 to 11.10) | 8.59 (4.15 to 13.03) | 8.71 (4.22 to 13.20) | 11.39 (-8.56 to 31.35) | 13.85 (-7.13 to 34.84) | 13.96 (-7.20 to 35.13) | 9.21 (2.55 to 15.87) | 11.25 (4.23 to 18.26) | 11.36 (4.29 to 18.44) |
| **Kuwait** | 13.00 (7.46 to 18.54) | 21.09 (12.52 to 29.67) | 19.67 (13.15 to 26.18) | 16.70 (-0.31 to 33.71) | 21.22 (-2.20 to 44.65) | 20.61 (1.78 to 39.45) | 14.14 (8.27 to 20.02) | 21.55 (12.18 to 30.91) | 20.40 (13.09 to 27.72) |
| **Kyrgyzstan** | 15.67 (13.26 to 18.07) | 12.84 (10.70 to 14.98) | 11.25 (9.67 to 12.83) | 14.50 (8.80 to 20.21) | 13.81 (6.89 to 20.73) | 13.06 (7.57 to 18.54) | 14.89 (12.49 to 17.28) | 13.28 (10.74 to 15.82) | 12.15 (10.21 to 14.09) |
| **Lao People's Democratic Republic** | 7.18 (6.59 to 7.78) | 10.76 (9.60 to 11.92) | 9.91 (8.97 to 10.85) | 7.87 (6.65 to 9.09) | 12.22 (8.90 to 15.54) | 11.91 (8.92 to 14.90) | 7.58 (6.97 to 8.19) | 11.51 (10.12 to 12.89) | 10.93 (9.75 to 12.12) |
| **Latvia** | 22.17 (18.82 to 25.51) | 33.46 (24.09 to 42.84) | 28.41 (20.99 to 35.84) | 20.27 (11.05 to 29.50) | 28.08 (8.13 to 48.03) | 25.89 (9.59 to 42.18) | 20.96 (17.50 to 24.42) | 30.33 (21.36 to 39.31) | 27.11 (20.03 to 34.18) |
| **Lebanon** | 17.10 (14.23 to 19.97) | 33.05 (23.02 to 43.09) | 25.12 (19.88 to 30.35) | 16.48 (9.36 to 23.60) | 27.77 (12.03 to 43.51) | 24.34 (13.98 to 34.70) | 17.02 (13.97 to 20.07) | 30.84 (21.84 to 39.85) | 25.22 (20.03 to 30.41) |
| **Lesotho** | 8.27 (7.21 to 9.32) | 8.39 (7.76 to 9.02) | 5.31 (5.05 to 5.57) | 8.81 (6.06 to 11.56) | 10.77 (9.44 to 12.10) | 8.41 (7.61 to 9.22) | 8.68 (7.47 to 9.89) | 9.57 (8.93 to 10.21) | 6.80 (6.48 to 7.12) |
| **Liberia** | 5.10 (4.48 to 5.72) | 8.95 (7.96 to 9.94) | 7.43 (6.82 to 8.05) | 5.72 (4.60 to 6.84) | 9.63 (7.75 to 11.51) | 9.22 (7.62 to 10.83) | 5.44 (4.86 to 6.03) | 9.25 (8.30 to 10.20) | 8.29 (7.59 to 9.00) |
| **Libya** | 19.28 (14.10 to 24.47) | 23.45 (19.47 to 27.42) | 19.88 (17.11 to 22.65) | 14.66 (6.82 to 22.51) | 20.62 (13.96 to 27.28) | 18.76 (13.11 to 24.41) | 17.14 (12.88 to 21.40) | 22.26 (18.76 to 25.76) | 19.55 (16.86 to 22.25) |
| **Lithuania** | 24.64 (21.04 to 28.23) | 37.19 (28.64 to 45.73) | 32.94 (26.10 to 39.78) | 21.74 (11.89 to 31.59) | 28.11 (9.56 to 46.66) | 26.33 (11.00 to 41.66) | 23.04 (19.28 to 26.79) | 32.47 (24.20 to 40.73) | 29.72 (23.05 to 36.39) |
| **Luxembourg** | 36.98 (6.58 to 67.38) | 44.85 (-3.61 to 93.32) | 41.54 (-1.64 to 84.72) | 33.09  (-20.46 to 86.64) | 40.47  (-26.61 to 107.55) | 37.54  (-22.98 to 98.05) | 34.80 (7.28 to 62.33) | 42.45 (2.51 to 82.39) | 39.43 (3.15 to 75.71) |
| **Madagascar** | 6.15 (5.83 to 6.46) | 7.15 (6.82 to 7.49) | 5.63 (5.44 to 5.82) | 9.74 (8.51 to 10.97) | 10.96 (9.84 to 12.09) | 10.27 (9.38 to 11.16) | 7.94 (7.45 to 8.43) | 9.07 (8.59 to 9.55) | 7.94 (7.61 to 8.26) |
| **Malawi** | 4.94 (4.70 to 5.18) | 9.41 (8.92 to 9.90) | 6.53 (6.30 to 6.76) | 7.71 (7.01 to 8.41) | 13.34 (11.84 to 14.83) | 11.19 (10.26 to 12.12) | 6.34 (6.03 to 6.64) | 11.37 (10.75 to 11.99) | 8.78 (8.44 to 9.12) |
| **Malaysia** | 12.78 (11.62 to 13.93) | 16.66 (15.40 to 17.93) | 14.90 (13.97 to 15.84) | 14.37 (11.09 to 17.64) | 19.99 (16.19 to 23.80) | 19.08 (15.91 to 22.25) | 13.58 (12.23 to 14.92) | 18.30 (16.80 to 19.80) | 16.94 (15.77 to 18.11) |
| **Maldives** | 8.12 (3.05 to 13.18) | 11.59 (0.29 to 22.89) | 10.37 (2.25 to 18.50) | 7.96 (-0.42 to 16.33) | 10.66  (-19.59 to 40.92) | 10.21  (-14.95 to 35.37) | 8.20 (3.47 to 12.93) | 11.09 (-0.51 to 22.69) | 10.20 (1.55 to 18.85) |
| **Mali** | 7.07 (6.56 to 7.59) | 10.24 (9.58 to 10.90) | 7.17 (6.89 to 7.44) | 7.33 (6.56 to 8.11) | 10.05 (9.02 to 11.08) | 8.91 (8.19 to 9.63) | 7.20 (6.74 to 7.66) | 10.20 (9.61 to 10.78) | 8.08 (7.76 to 8.40) |
| **Malta** | 30.36 (-4.25 to 64.97) | 38.33  (-21.87 to 98.52) | 36.07  (-21.41 to 93.55) | 28.24  (-31.52 to 87.99) | 33.48  (-48.50 to 115.47) | 34.54  (-45.48 to 114.55) | 29.29 (-3.30 to 61.88) | 35.72  (-13.95 to 85.40) | 35.08  (-12.36 to 82.51) |
| **Marshall Islands** | 8.23 (1.47 to 14.99) | 10.44 (2.48 to 18.40) | 10.66 (2.50 to 18.81) | 10.49 (-15.38 to 36.36) | 12.82 (-10.69 to 36.33) | 12.99 (-11.14 to 37.11) | 9.30 (0.29 to 18.30) | 11.66 (2.11 to 21.21) | 11.85 (2.07 to 21.64) |
| **Mauritania** | 9.78 (7.94 to 11.62) | 12.94 (10.59 to 15.29) | 11.36 (9.80 to 12.93) | 8.46 (6.67 to 10.26) | 12.29 (8.63 to 15.96) | 12.15 (8.87 to 15.43) | 9.01 (7.74 to 10.28) | 12.64 (10.51 to 14.77) | 11.86 (10.18 to 13.53) |
| **Mauritius** | 10.62 (8.17 to 13.08) | 15.97 (11.51 to 20.44) | 14.88 (11.10 to 18.66) | 13.54 (2.92 to 24.17) | 18.05 (3.05 to 33.06) | 17.48 (3.88 to 31.09) | 12.04 (8.66 to 15.42) | 16.92 (11.38 to 22.46) | 16.14 (11.26 to 21.02) |
| **Mexico** | 13.72 (13.19 to 14.25) | 17.79 (17.12 to 18.45) | 13.74 (13.35 to 14.13) | 16.98 (15.19 to 18.77) | 18.14 (16.39 to 19.89) | 16.25 (15.14 to 17.36) | 15.38 (14.72 to 16.05) | 18.10 (17.38 to 18.82) | 15.17 (14.74 to 15.60) |
| **Micronesia (Federated States of)** | 9.63 (4.08 to 15.19) | 12.14 (4.56 to 19.72) | 12.30 (4.68 to 19.91) | 12.08 (-6.96 to 31.12) | 14.86  (-10.01 to 39.74) | 15.00  (-10.11 to 40.10) | 10.75 (3.85 to 17.66) | 13.35 (4.14 to 22.57) | 13.50 (4.21 to 22.78) |
| **Monaco** | 46.25  (-147.60 to 240.10) | 59.35  (-188.16 to 306.87) | 55.05  (-175.09 to 285.20) | 38.32  (-231.58 to 308.21) | 53.52  (-253.21 to 360.26) | 51.39  (-236.48 to 339.26) | 42.27  (-118.47 to 203.01) | 56.55  (-138.58 to 251.69) | 53.43  (-128.25 to 235.11) |
| **Mongolia** | 19.46 (15.51 to 23.41) | 24.14 (19.57 to 28.71) | 22.22 (18.43 to 26.01) | 17.51 (10.70 to 24.31) | 24.50 (13.86 to 35.15) | 22.51 (13.58 to 31.45) | 18.56 (14.95 to 22.16) | 24.35 (19.79 to 28.91) | 22.38 (18.58 to 26.18) |
| **Montenegro** | 28.07 (8.18 to 47.97) | 34.62 (6.37 to 62.87) | 28.12 (7.81 to 48.43) | 22.81  (-15.45 to 61.07) | 29.31  (-18.62 to 77.25) | 25.45  (-13.29 to 64.19) | 25.43 (6.69 to 44.16) | 32.04 (6.66 to 57.42) | 27.01 (8.23 to 45.80) |
| **Morocco** | 6.55 (6.18 to 6.92) | 9.89 (9.32 to 10.47) | 8.87 (8.45 to 9.30) | 5.08 (4.51 to 5.66) | 8.85 (7.50 to 10.20) | 8.73 (7.51 to 9.94) | 5.90 (5.58 to 6.22) | 9.41 (8.74 to 10.07) | 8.88 (8.35 to 9.42) |
| **Mozambique** | 5.05 (4.86 to 5.24) | 7.31 (7.10 to 7.52) | 5.40 (5.29 to 5.51) | 8.16 (7.32 to 9.00) | 11.33 (10.57 to 12.10) | 9.65 (9.15 to 10.16) | 6.60 (6.29 to 6.92) | 9.32 (9.03 to 9.60) | 7.46 (7.28 to 7.63) |
| **Myanmar** | 6.20 (6.06 to 6.34) | 9.28 (8.99 to 9.58) | 8.33 (8.11 to 8.56) | 9.21 (8.60 to 9.81) | 12.21 (10.95 to 13.47) | 11.81 (10.70 to 12.91) | 7.70 (7.48 to 7.92) | 10.82 (10.37 to 11.27) | 10.15 (9.78 to 10.51) |
| **Namibia** | 5.98 (5.14 to 6.83) | 9.58 (8.44 to 10.73) | 6.51 (5.95 to 7.07) | 8.17 (5.06 to 11.28) | 12.45 (9.19 to 15.72) | 10.38 (8.34 to 12.42) | 7.07 (5.94 to 8.19) | 11.07 (9.71 to 12.43) | 8.46 (7.70 to 9.21) |
| **Nauru** | 10.00 (-4.92 to 24.92) | 11.04 (-6.47 to 28.55) | 11.31 (-6.99 to 29.62) | 14.44  (-50.30 to 79.18) | 15.69  (-51.38 to 82.76) | 16.03  (-53.86 to 85.91) | 12.05 (-8.56 to 32.67) | 13.35  (-10.57 to 37.27) | 13.65  (-11.33 to 38.62) |
| **Nepal** | 5.11 (4.87 to 5.35) | 8.08 (7.56 to 8.59) | 6.55 (6.23 to 6.87) | 6.10 (5.58 to 6.63) | 8.73 (7.56 to 9.91) | 8.06 (7.19 to 8.92) | 5.60 (5.35 to 5.85) | 8.49 (7.92 to 9.06) | 7.40 (7.01 to 7.78) |
| **Netherlands** | 42.04 (35.97 to 48.12) | 50.07 (40.36 to 59.78) | 47.37 (38.29 to 56.45) | 36.77 (26.69 to 46.85) | 43.91 (31.41 to 56.41) | 41.81 (29.77 to 53.85) | 39.27 (33.72 to 44.81) | 46.90 (39.06 to 54.73) | 44.61 (37.15 to 52.06) |
| **New Zealand** | 40.22 (30.19 to 50.25) | 50.96 (35.90 to 66.03) | 50.64 (35.58 to 65.71) | 38.56 (19.87 to 57.25) | 44.40 (23.95 to 64.85) | 44.06 (23.39 to 64.73) | 39.35 (29.58 to 49.13) | 47.83 (35.43 to 60.22) | 47.46 (34.97 to 59.95) |
| **Nicaragua** | 11.42 (8.58 to 14.25) | 16.56 (13.07 to 20.05) | 11.14 (9.65 to 12.64) | 13.27 (3.76 to 22.77) | 16.75 (7.84 to 25.65) | 13.51 (8.21 to 18.82) | 12.32 (8.47 to 16.16) | 16.67 (12.80 to 20.53) | 12.48 (10.54 to 14.43) |
| **Niger** | 4.50 (4.21 to 4.79) | 6.79 (6.47 to 7.12) | 5.43 (5.24 to 5.61) | 4.90 (4.30 to 5.50) | 7.62 (6.88 to 8.37) | 7.10 (6.51 to 7.69) | 4.69 (4.40 to 4.99) | 7.26 (6.90 to 7.61) | 6.32 (6.07 to 6.56) |
| **Nigeria** | 5.10 (5.02 to 5.18) | 8.85 (8.70 to 9.00) | 7.01 (6.92 to 7.10) | 5.70 (5.46 to 5.94) | 9.57 (9.22 to 9.92) | 9.12 (8.84 to 9.40) | 5.21 (5.12 to 5.29) | 9.39 (9.22 to 9.56) | 8.28 (8.16 to 8.40) |
| **Niue** | 11.40  (-35.87 to 58.67) | 14.59  (-63.01 to 92.19) | 14.48  (-61.99 to 90.95) | 13.83  (-192.30 to 219.97) | 16.66  (-296.07 to 329.39) | 16.95  (-282.69 to 316.58) | 12.51  (-55.16 to 80.18) | 15.52  (-92.12 to 123.15) | 15.63  (-100.17 to 131.43) |
| **North Macedonia** | 20.78 (13.66 to 27.90) | 28.68 (14.27 to 43.10) | 21.35 (13.27 to 29.43) | 18.17 (3.89 to 32.46) | 25.15 (2.28 to 48.03) | 21.89 (6.69 to 37.09) | 19.61 (12.62 to 26.60) | 26.89 (14.31 to 39.47) | 21.68 (14.17 to 29.19) |
| **Northern Mariana Islands** | 18.27 (-14.08 to 50.62) | 21.77 (-7.95 to 51.48) | 19.97 (-3.55 to 43.49) | 20.57  (-61.52 to 102.66) | 23.96  (-61.62 to 109.53) | 23.27  (-54.35 to 100.89) | 18.88  (-13.88 to 51.64) | 22.82  (-11.47 to 57.11) | 21.49 (-7.49 to 50.46) |
| **Norway** | 34.19 (25.40 to 42.98) | 48.71 (31.35 to 66.07) | 47.52 (30.39 to 64.65) | 32.09 (14.16 to 50.03) | 42.23 (18.91 to 65.54) | 41.32 (18.31 to 64.33) | 33.13 (24.39 to 41.86) | 45.38 (31.29 to 59.48) | 44.36 (30.40 to 58.31) |
| **Oman** | 6.92 (5.65 to 8.18) | 11.34 (8.54 to 14.14) | 8.47 (6.96 to 9.97) | 6.10 (2.65 to 9.54) | 9.35 (2.20 to 16.50) | 8.00 (3.88 to 12.12) | 6.48 (5.29 to 7.66) | 10.37 (7.78 to 12.96) | 8.19 (6.77 to 9.60) |
| **Pakistan** | 9.44 (9.14 to 9.73) | 12.46 (12.13 to 12.80) | 10.17 (9.97 to 10.37) | 9.89 (9.41 to 10.37) | 14.87 (14.20 to 15.54) | 13.90 (13.37 to 14.43) | 9.70 (9.43 to 9.97) | 13.63 (13.29 to 13.97) | 11.94 (11.71 to 12.17) |
| **Palau** | 14.09  (-10.84 to 39.01) | 15.60 (-9.92 to 41.12) | 15.71 (-9.53 to 40.95) | 25.20  (-96.39 to 146.79) | 26.26  (-87.22 to 139.73) | 26.29  (-88.73 to 141.32) | 19.28  (-20.09 to 58.65) | 20.50  (-13.16 to 54.16) | 20.51  (-12.83 to 53.85) |
| **Palestine** | 15.21 (10.29 to 20.14) | 20.70 (14.34 to 27.06) | 17.94 (13.99 to 21.89) | 13.99 (2.70 to 25.29) | 20.91 (6.51 to 35.31) | 20.13 (9.16 to 31.09) | 14.81 (9.43 to 20.19) | 20.61 (13.77 to 27.45) | 18.97 (14.34 to 23.60) |
| **Panama** | 19.85 (13.60 to 26.11) | 28.43 (20.99 to 35.86) | 26.27 (20.38 to 32.16) | 19.41 (2.75 to 36.06) | 24.42 (8.71 to 40.12) | 23.96 (10.03 to 37.89) | 19.75 (12.44 to 27.06) | 26.69 (18.97 to 34.41) | 25.41 (18.92 to 31.90) |
| **Papua New Guinea** | 6.46 (5.89 to 7.03) | 7.96 (7.41 to 8.50) | 6.03 (5.74 to 6.31) | 8.22 (6.30 to 10.13) | 9.35 (7.59 to 11.12) | 8.59 (7.29 to 9.89) | 7.32 (6.56 to 8.09) | 8.69 (7.98 to 9.41) | 7.32 (6.87 to 7.77) |
| **Paraguay** | 13.10 (9.95 to 16.26) | 20.39 (17.23 to 23.54) | 15.55 (13.78 to 17.32) | 14.24 (5.65 to 22.83) | 20.44 (11.74 to 29.15) | 17.73 (11.63 to 23.82) | 13.61 (9.80 to 17.41) | 20.49 (16.83 to 24.15) | 16.75 (14.47 to 19.03) |
| **Peru** | 15.40 (14.06 to 16.73) | 24.39 (21.96 to 26.83) | 14.99 (14.06 to 15.93) | 17.44 (14.16 to 20.71) | 26.57 (21.71 to 31.43) | 20.55 (17.88 to 23.21) | 16.45 (14.98 to 17.93) | 25.53 (23.06 to 28.00) | 17.78 (16.68 to 18.88) |
| **Philippines** | 9.86 (9.44 to 10.27) | 11.50 (11.17 to 11.83) | 9.19 (8.99 to 9.39) | 11.17 (9.84 to 12.50) | 14.36 (13.06 to 15.67) | 13.07 (12.14 to 14.01) | 10.54 (10.04 to 11.03) | 12.87 (12.41 to 13.32) | 11.06 (10.76 to 11.36) |
| **Poland** | 23.50 (22.18 to 24.82) | 34.98 (32.07 to 37.88) | 30.47 (28.15 to 32.79) | 21.32 (17.76 to 24.88) | 31.73 (25.59 to 37.88) | 29.28 (24.31 to 34.25) | 22.38 (21.01 to 23.75) | 33.11 (30.33 to 35.90) | 29.84 (27.62 to 32.07) |
| **Portugal** | 28.16 (24.05 to 32.27) | 43.77 (32.99 to 54.55) | 41.32 (31.17 to 51.47) | 25.61 (16.88 to 34.33) | 33.37 (18.14 to 48.60) | 31.64 (16.99 to 46.28) | 26.88 (22.79 to 30.96) | 38.53 (29.48 to 47.58) | 36.52 (27.95 to 45.10) |
| **Puerto Rico** | 24.21 (19.73 to 28.68) | 31.25 (22.27 to 40.23) | 30.70 (22.10 to 39.29) | 21.08 (10.65 to 31.50) | 27.53 (9.61 to 45.45) | 27.16 (9.73 to 44.59) | 23.10 (18.70 to 27.51) | 29.49 (21.00 to 37.98) | 29.08 (20.94 to 37.22) |
| **Qatar** | 15.99 (7.57 to 24.42) | 27.85 (13.42 to 42.28) | 26.85 (16.27 to 37.42) | 20.37  (-21.56 to 62.29) | 34.03 (-4.24 to 72.30) | 31.89 (-0.55 to 64.33) | 17.10 (6.96 to 27.23) | 29.05 (15.43 to 42.67) | 27.98 (17.42 to 38.54) |
| **Republic of Korea** | 29.52 (27.75 to 31.29) | 44.26 (39.96 to 48.55) | 43.59 (39.41 to 47.77) | 20.25 (17.28 to 23.22) | 30.49 (24.08 to 36.90) | 30.33 (24.01 to 36.66) | 24.68 (23.16 to 26.19) | 36.96 (33.27 to 40.65) | 36.69 (33.07 to 40.31) |
| **Republic of Moldova** | 18.88 (15.98 to 21.78) | 22.83 (18.24 to 27.43) | 20.83 (17.13 to 24.53) | 16.71 (10.66 to 22.75) | 18.53 (8.33 to 28.73) | 17.89 (9.47 to 26.32) | 17.63 (14.92 to 20.35) | 20.54 (16.20 to 24.89) | 19.35 (15.73 to 22.97) |
| **Romania** | 15.88 (14.66 to 17.09) | 29.88 (26.73 to 33.03) | 26.24 (23.78 to 28.71) | 15.05 (11.64 to 18.46) | 24.91 (17.88 to 31.95) | 23.58 (17.95 to 29.22) | 15.44 (14.09 to 16.80) | 27.42 (24.26 to 30.58) | 25.02 (22.53 to 27.50) |
| **Russian Federation** | 23.05 (22.49 to 23.60) | 26.82 (26.10 to 27.53) | 21.91 (21.39 to 22.43) | 20.22 (18.74 to 21.71) | 26.47 (24.66 to 28.28) | 24.02 (22.67 to 25.37) | 21.13 (20.57 to 21.69) | 26.44 (25.69 to 27.19) | 22.91 (22.36 to 23.46) |
| **Rwanda** | 6.62 (6.26 to 6.98) | 11.30 (10.33 to 12.27) | 8.63 (8.12 to 9.14) | 10.95 (9.67 to 12.23) | 14.37 (11.87 to 16.86) | 12.98 (11.17 to 14.79) | 8.87 (8.34 to 9.40) | 12.91 (11.75 to 14.07) | 10.92 (10.19 to 11.64) |
| **Saint Kitts and Nevis** | 17.22 (-3.59 to 38.03) | 28.85 (-3.34 to 61.03) | 25.64 (0.77 to 50.51) | 20.97  (-51.54 to 93.49) | 23.74  (-55.13 to 102.62) | 22.51  (-43.70 to 88.73) | 19.10  (-10.41 to 48.61) | 26.47 (-6.75 to 59.69) | 24.40 (-2.37 to 51.17) |
| **Saint Lucia** | 21.93 (2.79 to 41.06) | 31.02 (4.59 to 57.46) | 24.82 (7.67 to 41.98) | 20.51  (-25.62 to 66.65) | 22.09  (-32.43 to 76.61) | 21.15  (-21.45 to 63.75) | 21.26 (0.11 to 42.41) | 27.46 (1.31 to 53.62) | 23.60 (5.18 to 42.02) |
| **Saint Vincent and the Grenadines** | 20.57 (-1.51 to 42.64) | 27.09 (1.68 to 52.51) | 24.89 (3.66 to 46.11) | 21.19  (-35.63 to 78.01) | 23.51  (-36.79 to 83.80) | 22.82  (-33.20 to 78.83) | 20.59 (-4.40 to 45.58) | 25.77 (-1.56 to 53.09) | 24.49 (0.10 to 48.88) |
| **Samoa** | 9.18 (3.32 to 15.04) | 10.07 (3.62 to 16.51) | 10.32 (3.19 to 17.44) | 11.51  (-11.07 to 34.08) | 13.73 (-8.33 to 35.79) | 14.05 (-9.37 to 37.47) | 10.28 (2.29 to 18.28) | 11.76 (3.10 to 20.42) | 12.04 (2.69 to 21.40) |
| **San Marino** | 51.64  (-194.26 to 297.54) | 56.56  (-234.84 to 347.95) | 41.50  (-227.55 to 310.56) | 37.37  (-286.53 to 361.26) | 39.18  (-304.55 to 382.91) | 30.23  (-295.28 to 355.74) | 44.86  (-150.72 to 240.44) | 49.16  (-174.69 to 273.01) | 36.16  (-172.55 to 244.88) |
| **Sao Tome and Principe** | 8.41 (3.33 to 13.48) | 13.02 (4.96 to 21.08) | 11.58 (5.67 to 17.50) | 8.58 (-5.24 to 22.41) | 13.00 (-9.68 to 35.67) | 12.54 (-6.89 to 31.96) | 8.37 (2.19 to 14.56) | 12.94 (3.17 to 22.71) | 12.05 (4.29 to 19.81) |
| **Saudi Arabia** | 6.85 (6.41 to 7.30) | 11.82 (10.93 to 12.72) | 11.26 (10.45 to 12.06) | 6.79 (5.73 to 7.84) | 14.39 (12.18 to 16.61) | 14.14 (12.06 to 16.21) | 6.76 (6.32 to 7.19) | 12.82 (11.92 to 13.72) | 12.40 (11.58 to 13.22) |
| **Senegal** | 6.33 (5.88 to 6.77) | 10.34 (9.65 to 11.02) | 7.86 (7.50 to 8.22) | 6.90 (5.84 to 7.96) | 10.88 (9.25 to 12.51) | 9.86 (8.65 to 11.07) | 6.69 (6.20 to 7.19) | 10.67 (9.91 to 11.43) | 8.95 (8.47 to 9.43) |
| **Serbia** | 22.61 (19.63 to 25.60) | 31.92 (25.09 to 38.76) | 28.30 (22.75 to 33.86) | 20.38 (13.67 to 27.08) | 27.61 (15.68 to 39.53) | 26.28 (15.89 to 36.67) | 21.48 (18.43 to 24.53) | 29.75 (23.47 to 36.02) | 27.36 (22.13 to 32.59) |
| **Seychelles** | 18.94 (-0.54 to 38.41) | 27.85 (0.00 to 55.70) | 24.44 (2.46 to 46.42) | 17.18  (-50.19 to 84.55) | 22.50  (-49.52 to 94.52) | 19.98  (-38.13 to 78.08) | 18.27 (-5.24 to 41.79) | 25.20 (-3.88 to 54.29) | 22.28 (-2.17 to 46.72) |
| **Sierra Leone** | 5.09 (4.69 to 5.50) | 7.08 (6.59 to 7.57) | 6.11 (5.78 to 6.44) | 5.17 (4.30 to 6.04) | 8.36 (7.15 to 9.56) | 8.03 (7.00 to 9.07) | 5.21 (4.79 to 5.62) | 7.67 (7.13 to 8.22) | 7.04 (6.62 to 7.47) |
| **Singapore** | 25.75 (18.03 to 33.47) | 35.01 (19.96 to 50.07) | 33.42 (19.24 to 47.59) | 22.49 (8.27 to 36.72) | 31.76 (8.96 to 54.57) | 30.19 (8.09 to 52.29) | 24.10 (16.78 to 31.42) | 33.04 (19.87 to 46.20) | 31.52 (18.99 to 44.06) |
| **Slovakia** | 28.66 (24.41 to 32.90) | 38.99 (29.56 to 48.42) | 32.93 (26.02 to 39.84) | 23.96 (14.02 to 33.89) | 31.81 (15.61 to 48.02) | 28.81 (15.85 to 41.78) | 26.55 (22.34 to 30.77) | 35.18 (26.76 to 43.60) | 30.91 (24.55 to 37.27) |
| **Slovenia** | 29.37 (20.56 to 38.18) | 46.22 (21.96 to 70.49) | 42.02 (20.22 to 63.81) | 26.50 (6.85 to 46.16) | 38.17 (0.87 to 75.46) | 34.84 (1.17 to 68.52) | 27.65 (18.91 to 36.39) | 41.98 (21.27 to 62.68) | 38.40 (20.10 to 56.69) |
| **Solomon Islands** | 7.40 (5.35 to 9.45) | 9.27 (6.92 to 11.63) | 9.27 (6.97 to 11.56) | 8.48 (2.74 to 14.22) | 11.57 (4.04 to 19.11) | 11.51 (4.22 to 18.81) | 8.03 (5.73 to 10.33) | 10.44 (7.53 to 13.36) | 10.41 (7.58 to 13.25) |
| **Somalia** | 5.47 (5.21 to 5.73) | 6.62 (6.36 to 6.88) | 4.48 (4.37 to 4.59) | 8.78 (7.84 to 9.72) | 10.49 (9.59 to 11.39) | 8.81 (8.26 to 9.36) | 7.06 (6.70 to 7.42) | 8.56 (8.20 to 8.91) | 6.57 (6.39 to 6.75) |
| **South Africa** | 12.82 (12.18 to 13.46) | 14.85 (14.43 to 15.28) | 10.02 (9.84 to 10.20) | 12.06 (10.93 to 13.20) | 15.14 (14.36 to 15.93) | 12.83 (12.34 to 13.33) | 12.40 (11.84 to 12.97) | 14.86 (14.47 to 15.26) | 11.45 (11.25 to 11.65) |
| **South Sudan** | 6.51 (6.08 to 6.94) | 7.90 (7.40 to 8.39) | 6.38 (6.08 to 6.68) | 9.20 (7.47 to 10.92) | 10.90 (9.38 to 12.42) | 10.22 (9.01 to 11.43) | 7.66 (7.08 to 8.23) | 9.37 (8.76 to 9.99) | 8.20 (7.76 to 8.63) |
| **Spain** | 37.71 (34.37 to 41.05) | 48.81 (42.82 to 54.80) | 45.94 (40.37 to 51.51) | 28.74 (22.98 to 34.50) | 35.64 (27.47 to 43.80) | 33.71 (25.92 to 41.49) | 33.18 (30.22 to 36.14) | 42.30 (37.38 to 47.22) | 39.97 (35.34 to 44.60) |
| **Sri Lanka** | 8.44 (7.92 to 8.96) | 12.14 (10.94 to 13.34) | 10.96 (9.95 to 11.96) | 10.59 (8.46 to 12.73) | 13.07 (9.33 to 16.81) | 12.30 (9.07 to 15.52) | 9.50 (8.84 to 10.16) | 12.60 (11.19 to 14.01) | 11.64 (10.45 to 12.84) |
| **Sudan** | 6.67 (6.32 to 7.01) | 10.63 (9.95 to 11.32) | 8.66 (8.25 to 9.06) | 6.06 (5.53 to 6.58) | 9.62 (8.49 to 10.75) | 9.04 (8.15 to 9.93) | 6.43 (6.13 to 6.73) | 10.32 (9.69 to 10.95) | 9.01 (8.58 to 9.44) |
| **Suriname** | 13.71 (7.43 to 19.98) | 19.49 (11.74 to 27.24) | 14.95 (9.86 to 20.04) | 15.07 (-2.12 to 32.26) | 18.56 (0.12 to 37.00) | 16.03 (1.39 to 30.67) | 14.38 (7.34 to 21.42) | 19.10 (10.88 to 27.31) | 15.62 (9.61 to 21.63) |
| **Sweden** | 33.76 (26.85 to 40.67) | 42.90 (31.63 to 54.16) | 39.73 (29.11 to 50.34) | 32.96 (19.71 to 46.22) | 39.68 (23.17 to 56.19) | 37.46 (21.49 to 53.43) | 33.33 (26.58 to 40.07) | 41.22 (31.55 to 50.88) | 38.51 (29.28 to 47.74) |
| **Switzerland** | 38.29 (29.91 to 46.67) | 46.69 (32.38 to 61.00) | 44.80 (31.30 to 58.30) | 30.48 (16.68 to 44.27) | 38.10 (19.17 to 57.03) | 35.84 (17.58 to 54.10) | 34.26 (26.87 to 41.66) | 42.18 (30.63 to 53.73) | 40.24 (29.23 to 51.25) |
| **Syrian Arab Republic** | 8.96 (8.00 to 9.92) | 11.03 (10.14 to 11.92) | 11.21 (10.21 to 12.21) | 8.30 (6.35 to 10.26) | 12.38 (9.60 to 15.15) | 12.23 (9.18 to 15.29) | 8.73 (7.77 to 9.68) | 11.98 (10.86 to 13.10) | 11.91 (10.62 to 13.21) |
| **Taiwan (Province of China)** | 21.99 (19.55 to 24.42) | 39.86 (34.45 to 45.26) | 39.39 (34.01 to 44.78) | 18.69 (13.75 to 23.63) | 31.70 (23.09 to 40.32) | 30.80 (22.26 to 39.33) | 20.59 (18.28 to 22.90) | 36.10 (31.39 to 40.81) | 35.38 (30.68 to 40.09) |
| **Tajikistan** | 12.81 (10.53 to 15.10) | 9.65 (7.78 to 11.52) | 7.49 (6.56 to 8.43) | 11.07 (7.22 to 14.91) | 9.35 (5.77 to 12.93) | 8.43 (6.25 to 10.60) | 11.83 (9.80 to 13.85) | 9.52 (7.75 to 11.28) | 7.99 (7.03 to 8.94) |
| **Thailand** | 17.73 (16.86 to 18.60) | 23.25 (22.05 to 24.46) | 22.37 (21.30 to 23.43) | 16.80 (14.94 to 18.66) | 22.18 (19.47 to 24.89) | 21.76 (19.21 to 24.31) | 17.28 (16.44 to 18.13) | 22.74 (21.55 to 23.93) | 22.13 (21.05 to 23.22) |
| **Timor-Leste** | 5.91 (4.10 to 7.72) | 8.29 (6.19 to 10.39) | 7.26 (5.73 to 8.79) | 7.00 (3.88 to 10.11) | 10.52 (4.03 to 17.00) | 9.96 (4.56 to 15.35) | 6.41 (4.72 to 8.09) | 9.38 (6.71 to 12.04) | 8.56 (6.49 to 10.64) |
| **Togo** | 5.46 (4.92 to 6.01) | 8.22 (7.67 to 8.77) | 7.01 (6.64 to 7.37) | 6.93 (5.40 to 8.46) | 9.78 (8.27 to 11.29) | 9.35 (8.03 to 10.66) | 6.26 (5.56 to 6.96) | 9.00 (8.35 to 9.65) | 8.22 (7.71 to 8.72) |
| **Tokelau** | 10.82  (-46.77 to 68.41) | 14.53  (-92.16 to 121.22) | 14.34  (-90.00 to 118.69) | 13.25  (-178.05 to 204.56) | 16.41  (-322.79 to 355.61) | 16.35  (-296.10 to 328.80) | 12.16  (-72.75 to 97.08) | 15.36  (-125.89 to 156.62) | 15.26  (-125.85 to 156.37) |
| **Tonga** | 17.80 (-1.09 to 36.70) | 21.02 (-2.30 to 44.33) | 21.26 (-2.54 to 45.05) | 18.49  (-30.08 to 67.07) | 20.46  (-38.00 to 78.93) | 20.72  (-39.09 to 80.54) | 18.43 (-4.38 to 41.23) | 20.85 (-6.03 to 47.72) | 21.10 (-6.50 to 48.70) |
| **Trinidad**  **and Tobago** | 17.39 (13.28 to 21.50) | 24.03 (17.94 to 30.12) | 19.14 (15.14 to 23.14) | 18.38 (6.33 to 30.42) | 23.06 (6.75 to 39.37) | 21.08 (7.85 to 34.31) | 17.93 (13.01 to 22.85) | 23.66 (16.88 to 30.45) | 20.34 (15.37 to 25.31) |
| **Tunisia** | 13.24 (11.06 to 15.41) | 18.58 (15.24 to 21.91) | 14.64 (12.71 to 16.58) | 10.27 (6.25 to 14.29) | 15.51 (9.12 to 21.91) | 13.98 (9.58 to 18.37) | 12.03 (9.97 to 14.09) | 17.22 (13.99 to 20.45) | 14.54 (12.55 to 16.54) |
| **Turkey** | 22.53 (21.19 to 23.88) | 30.82 (28.43 to 33.22) | 27.04 (25.30 to 28.79) | 13.35 (11.55 to 15.14) | 21.60 (18.07 to 25.13) | 20.40 (17.61 to 23.19) | 18.46 (17.38 to 19.53) | 26.34 (24.32 to 28.36) | 24.00 (22.48 to 25.52) |
| **Turkmenistan** | 13.68 (11.22 to 16.15) | 10.31 (8.47 to 12.15) | 9.02 (7.59 to 10.46) | 12.69 (7.31 to 18.06) | 11.43 (6.42 to 16.44) | 10.75 (6.68 to 14.81) | 13.04 (10.63 to 15.45) | 10.80 (8.83 to 12.77) | 9.84 (8.29 to 11.39) |
| **Tuvalu** | 7.77 (-3.81 to 19.35) | 11.23 (-8.11 to 30.57) | 11.49 (-8.48 to 31.46) | 10.46  (-27.08 to 47.99) | 13.98  (-58.03 to 85.99) | 14.17  (-60.05 to 88.39) | 9.20 (-6.92 to 25.33) | 12.44  (-12.51 to 37.40) | 12.64  (-12.96 to 38.24) |
| **Uganda** | 6.50 (6.28 to 6.72) | 14.46 (13.73 to 15.19) | 10.52 (10.17 to 10.87) | 7.77 (7.31 to 8.23) | 16.46 (14.70 to 18.22) | 14.21 (13.10 to 15.32) | 7.19 (6.97 to 7.41) | 15.53 (14.74 to 16.33) | 12.44 (12.00 to 12.89) |
| **Ukraine** | 23.72 (22.65 to 24.79) | 20.85 (19.71 to 22.00) | 17.92 (16.97 to 18.86) | 20.32 (17.55 to 23.10) | 18.29 (15.45 to 21.14) | 16.69 (14.32 to 19.06) | 21.59 (20.51 to 22.67) | 19.42 (18.27 to 20.56) | 17.24 (16.30 to 18.19) |
| **United**  **Arab Emirates** | 15.31 (10.22 to 20.40) | 23.23 (17.56 to 28.91) | 17.94 (13.43 to 22.46) | 22.96 (2.92 to 43.01) | 35.82 (21.44 to 50.20) | 33.51 (20.61 to 46.41) | 17.76 (12.12 to 23.40) | 27.74 (22.64 to 32.85) | 23.23 (18.86 to 27.60) |
| **United Kingdom** | 36.84 (34.16 to 39.52) | 46.66 (42.43 to 50.88) | 41.76 (38.30 to 45.23) | 36.53 (31.52 to 41.53) | 43.50 (37.60 to 49.41) | 40.34 (35.16 to 45.52) | 36.48 (33.82 to 39.14) | 45.03 (41.50 to 48.57) | 41.11 (38.12 to 44.11) |
| **United Republic of Tanzania** | 7.59 (7.29 to 7.89) | 10.05 (9.65 to 10.45) | 7.69 (7.47 to 7.90) | 10.32 (9.54 to 11.11) | 13.77 (12.76 to 14.79) | 12.45 (11.69 to 13.20) | 8.97 (8.62 to 9.33) | 11.92 (11.46 to 12.39) | 10.03 (9.73 to 10.33) |
| **United States of America** | 43.94 (42.77 to 45.11) | 42.98 (41.85 to 44.12) | 39.33 (38.43 to 40.23) | 41.00 (38.83 to 43.17) | 40.55 (38.68 to 42.42) | 38.56 (36.96 to 40.17) | 42.45 (41.34 to 43.57) | 41.79 (40.77 to 42.81) | 39.05 (38.21 to 39.88) |
| **United States Virgin Islands** | 24.40 (-0.22 to 49.01) | 27.50 (-5.40 to 60.39) | 23.81 (-0.08 to 47.69) | 23.35  (-32.34 to 79.03) | 25.64  (-65.23 to 116.50) | 20.89  (-50.95 to 92.74) | 23.77 (-0.92 to 48.46) | 26.70 (-7.60 to 61.00) | 22.74 (-4.57 to 50.06) |
| **Uruguay** | 30.76 (23.86 to 37.65) | 38.09 (28.00 to 48.19) | 34.74 (26.39 to 43.08) | 26.67 (12.71 to 40.63) | 33.13 (15.38 to 50.88) | 31.25 (15.54 to 46.95) | 28.84 (22.04 to 35.65) | 35.53 (26.24 to 44.83) | 33.09 (25.15 to 41.02) |
| **Uzbekistan** | 12.02 (10.93 to 13.10) | 8.58 (7.82 to 9.34) | 8.00 (7.36 to 8.64) | 11.36 (8.89 to 13.82) | 10.34 (8.10 to 12.58) | 10.01 (8.04 to 11.99) | 11.53 (10.45 to 12.62) | 9.45 (8.54 to 10.37) | 9.01 (8.23 to 9.79) |
| **Vanuatu** | 7.47 (4.35 to 10.59) | 9.18 (6.00 to 12.35) | 8.12 (5.74 to 10.49) | 8.68 (-2.09 to 19.45) | 10.72 (-0.94 to 22.37) | 10.24 (0.47 to 20.01) | 8.15 (4.40 to 11.90) | 10.02 (6.02 to 14.02) | 9.24 (6.03 to 12.45) |
| **Venezuela (Bolivarian Republic of)** | 17.30 (15.70 to 18.89) | 23.14 (21.65 to 24.63) | 17.94 (17.12 to 18.76) | 19.58 (14.82 to 24.34) | 23.80 (19.38 to 28.21) | 21.66 (18.31 to 25.00) | 18.47 (16.53 to 20.40) | 23.63 (21.85 to 25.42) | 20.10 (18.93 to 21.26) |
| **Viet Nam** | 12.65 (12.11 to 13.19) | 16.63 (15.95 to 17.31) | 16.29 (15.66 to 16.92) | 9.61 (8.57 to 10.65) | 14.32 (12.53 to 16.12) | 14.27 (12.57 to 15.97) | 11.12 (10.61 to 11.63) | 15.47 (14.76 to 16.19) | 15.30 (14.63 to 15.97) |
| **Yemen** | 7.43 (6.96 to 7.89) | 9.46 (8.97 to 9.96) | 7.76 (7.47 to 8.04) | 5.87 (5.26 to 6.48) | 8.67 (7.76 to 9.58) | 8.02 (7.35 to 8.69) | 6.63 (6.27 to 7.00) | 9.19 (8.73 to 9.65) | 8.00 (7.70 to 8.30) |
| **Zambia** | 6.72 (6.33 to 7.11) | 12.47 (11.75 to 13.19) | 8.27 (7.98 to 8.57) | 9.91 (8.77 to 11.04) | 15.49 (13.73 to 17.26) | 12.91 (11.84 to 13.98) | 8.33 (7.83 to 8.82) | 13.96 (13.17 to 14.76) | 10.46 (10.07 to 10.86) |
| **Zimbabwe** | 11.24 (10.49 to 11.99) | 11.51 (10.98 to 12.03) | 6.91 (6.71 to 7.11) | 13.77 (11.95 to 15.59) | 17.36 (15.95 to 18.77) | 13.93 (13.05 to 14.81) | 12.46 (11.65 to 13.26) | 14.62 (13.97 to 15.27) | 10.42 (10.10 to 10.75) |

Table S8. Lifetime risk of dying from cancer in 204 countries and territories by sex in 1990, 2019 and 2021.

| **Location** | **Male** | | | **Female** | | | **Both** | | |
| --- | --- | --- | --- | --- | --- | --- | --- | --- | --- |
|  | **1990** | **2019** | **2021** | **1990** | **2019** | **2021** | **1990** | **2019** | **2021** |
| **Afghanistan** | 6.16 (5.90 to 6.42) | 7.94 (7.56 to 8.32) | 5.14 (5.00 to 5.28) | 8.53 (7.96 to 9.11) | 11.60 (10.79 to 12.41) | 9.94 (9.41 to 10.48) | 7.37 (7.07 to 7.67) | 9.87 (9.46 to 10.28) | 7.55 (7.34 to 7.76) |
| **Albania** | 17.94 (13.65 to 22.23) | 19.40 (13.70 to 25.11) | 15.80 (12.34 to 19.26) | 11.00 (5.14 to 16.85) | 11.98 (4.00 to 19.96) | 10.57 (5.09 to 16.05) | 14.72 (11.29 to 18.15) | 15.84 (11.32 to 20.36) | 13.43 (10.53 to 16.33) |
| **Algeria** | 7.11 (6.64 to 7.58) | 8.04 (7.53 to 8.54) | 5.85 (5.63 to 6.07) | 5.89 (5.25 to 6.52) | 6.40 (5.75 to 7.05) | 5.52 (5.13 to 5.92) | 6.56 (6.18 to 6.95) | 7.25 (6.85 to 7.65) | 5.74 (5.54 to 5.94) |
| **American Samoa** | 15.23 (-1.48 to 31.95) | 16.54 (1.33 to 31.75) | 16.59 (1.50 to 31.68) | 12.87  (-21.47 to 47.21) | 15.19  (-14.21 to 44.59) | 15.17  (-14.50 to 44.84) | 14.31 (-0.85 to 29.46) | 15.92 (1.94 to 29.89) | 15.95 (2.00 to 29.89) |
| **Andorra** | 38.51  (-46.58 to 123.61) | 37.72  (-41.23 to 116.68) | 32.28  (-30.46 to 95.01) | 19.63  (-67.76 to 107.02) | 20.26  (-55.46 to 95.99) | 17.48  (-43.43 to 78.40) | 30.54  (-30.59 to 91.66) | 29.34  (-24.60 to 83.29) | 25.35  (-17.70 to 68.40) |
| **Angola** | 5.80 (5.57 to 6.03) | 9.37 (9.02 to 9.73) | 6.65 (6.49 to 6.81) | 5.97 (5.47 to 6.46) | 8.44 (7.92 to 8.97) | 7.23 (6.90 to 7.56) | 5.99 (5.76 to 6.22) | 8.98 (8.67 to 9.28) | 7.05 (6.89 to 7.21) |
| **Antigua and Barbuda** | 19.96 (0.24 to 39.68) | 23.36 (3.03 to 43.69) | 19.22 (5.24 to 33.20) | 14.48  (-24.36 to 53.31) | 18.29  (-18.15 to 54.73) | 16.09  (-13.24 to 45.42) | 17.42 (-1.10 to 35.93) | 20.56 (2.19 to 38.92) | 17.79 (3.85 to 31.73) |
| **Argentina** | 21.65 (20.44 to 22.86) | 22.71 (21.45 to 23.98) | 18.79 (17.90 to 19.69) | 19.10 (16.85 to 21.36) | 19.89 (17.50 to 22.29) | 17.27 (15.46 to 19.08) | 20.46 (19.35 to 21.56) | 21.24 (20.07 to 22.41) | 18.04 (17.19 to 18.89) |
| **Armenia** | 18.69 (14.81 to 22.58) | 21.02 (16.44 to 25.61) | 18.42 (15.03 to 21.81) | 14.42 (6.83 to 22.01) | 15.82 (6.51 to 25.13) | 14.38 (8.01 to 20.75) | 16.40 (12.94 to 19.86) | 18.31 (14.23 to 22.39) | 16.42 (13.46 to 19.38) |
| **Australia** | 27.26 (24.79 to 29.73) | 29.77 (26.83 to 32.71) | 29.09 (26.33 to 31.85) | 20.70 (16.02 to 25.39) | 22.40 (18.13 to 26.67) | 21.66 (17.62 to 25.71) | 23.85 (21.63 to 26.08) | 25.96 (23.57 to 28.34) | 25.33 (23.08 to 27.58) |
| **Austria** | 25.67 (22.65 to 28.68) | 26.40 (22.27 to 30.53) | 23.74 (20.31 to 27.17) | 21.44 (15.65 to 27.23) | 21.10 (13.70 to 28.50) | 19.56 (13.24 to 25.88) | 23.17 (20.48 to 25.86) | 23.53 (19.88 to 27.19) | 21.51 (18.47 to 24.55) |
| **Azerbaijan** | 14.22 (12.49 to 15.95) | 14.02 (12.26 to 15.79) | 10.72 (9.73 to 11.71) | 10.34 (7.40 to 13.27) | 9.86 (6.96 to 12.76) | 8.43 (6.78 to 10.07) | 12.10 (10.65 to 13.55) | 11.86 (10.41 to 13.32) | 9.62 (8.78 to 10.46) |
| **Bahamas** | 19.30 (7.70 to 30.89) | 21.08 (12.90 to 29.26) | 15.15 (10.72 to 19.59) | 15.71 (-2.43 to 33.85) | 16.59 (4.96 to 28.22) | 14.64 (4.82 to 24.46) | 17.39 (7.81 to 26.97) | 18.91 (12.33 to 25.49) | 15.22 (10.81 to 19.62) |
| **Bahrain** | 15.20 (9.21 to 21.18) | 15.75 (10.74 to 20.76) | 12.41 (10.00 to 14.83) | 11.85 (0.40 to 23.30) | 12.78 (3.44 to 22.12) | 11.56 (5.75 to 17.38) | 13.71 (8.56 to 18.87) | 14.10 (9.91 to 18.28) | 11.84 (9.65 to 14.03) |
| **Bangladesh** | 6.62 (6.48 to 6.76) | 8.32 (8.10 to 8.53) | 7.24 (7.08 to 7.39) | 5.43 (5.22 to 5.63) | 6.64 (6.29 to 7.00) | 6.30 (6.01 to 6.59) | 6.20 (6.08 to 6.32) | 7.64 (7.44 to 7.83) | 6.92 (6.77 to 7.06) |
| **Barbados** | 21.31 (8.79 to 33.82) | 24.40 (11.18 to 37.61) | 22.19 (11.09 to 33.30) | 18.21 (-6.16 to 42.59) | 22.12 (-3.30 to 47.54) | 20.63 (-1.51 to 42.76) | 19.51 (7.87 to 31.15) | 23.19 (10.64 to 35.74) | 21.40 (10.59 to 32.21) |
| **Belarus** | 19.79 (18.20 to 21.38) | 18.40 (16.88 to 19.93) | 14.64 (13.66 to 15.61) | 13.02 (10.38 to 15.66) | 12.41 (9.38 to 15.45) | 10.74 (8.83 to 12.64) | 15.93 (14.67 to 17.20) | 15.21 (13.87 to 16.55) | 12.72 (11.85 to 13.60) |
| **Belgium** | 31.89 (28.33 to 35.45) | 29.87 (25.86 to 33.88) | 28.07 (24.37 to 31.78) | 23.09 (17.65 to 28.52) | 22.90 (17.03 to 28.77) | 21.49 (16.02 to 26.97) | 27.28 (24.32 to 30.24) | 26.15 (22.89 to 29.41) | 24.64 (21.60 to 27.68) |
| **Belize** | 12.24 (2.57 to 21.90) | 15.27 (7.98 to 22.56) | 12.09 (7.70 to 16.49) | 10.78 (-7.24 to 28.79) | 13.32 (0.35 to 26.29) | 11.80 (2.20 to 21.40) | 11.45 (2.65 to 20.25) | 14.52 (8.02 to 21.02) | 12.16 (7.82 to 16.51) |
| **Benin** | 6.39 (5.84 to 6.95) | 8.54 (8.02 to 9.06) | 6.51 (6.23 to 6.79) | 6.03 (5.17 to 6.88) | 7.84 (6.98 to 8.70) | 7.00 (6.38 to 7.61) | 6.31 (5.83 to 6.80) | 8.22 (7.77 to 8.68) | 6.82 (6.54 to 7.10) |
| **Bermuda** | 25.87 (-2.75 to 54.48) | 29.73  (-11.66 to 71.11) | 24.68  (-3.27 to 52.62) | 23.30  (-39.01 to 85.61) | 22.56  (-56.47 to 101.60) | 19.93  (-35.41 to 75.28) | 24.84 (-2.61 to 52.29) | 26.23  (-11.16 to 63.62) | 22.57 (-3.25 to 48.40) |
| **Bhutan** | 6.31 (4.26 to 8.37) | 8.81 (5.38 to 12.24) | 8.64 (5.42 to 11.86) | 5.68 (3.05 to 8.30) | 8.10 (2.45 to 13.74) | 8.05 (2.65 to 13.45) | 5.92 (4.33 to 7.51) | 8.47 (5.49 to 11.45) | 8.36 (5.54 to 11.18) |
| **Bolivia (Plurinational State of)** | 14.00 (12.57 to 15.42) | 18.74 (16.94 to 20.54) | 9.27 (8.76 to 9.77) | 16.29 (13.67 to 18.91) | 20.43 (17.09 to 23.76) | 14.74 (13.23 to 16.24) | 15.13 (13.76 to 16.51) | 19.55 (17.82 to 21.28) | 11.87 (11.27 to 12.46) |
| **Bosnia and Herzegovina** | 19.92 (16.43 to 23.40) | 25.25 (19.36 to 31.13) | 20.75 (16.89 to 24.61) | 13.29 (8.60 to 17.99) | 17.53 (8.01 to 27.04) | 15.34 (9.00 to 21.68) | 16.38 (13.79 to 18.98) | 21.25 (16.36 to 26.15) | 18.09 (14.83 to 21.35) |
| **Botswana** | 9.39 (8.01 to 10.78) | 12.20 (10.65 to 13.75) | 6.34 (5.94 to 6.73) | 9.44 (6.79 to 12.09) | 10.59 (8.80 to 12.38) | 7.38 (6.60 to 8.16) | 9.53 (8.22 to 10.84) | 11.27 (10.11 to 12.43) | 6.90 (6.51 to 7.29) |
| **Brazil** | 14.40 (14.07 to 14.74) | 18.18 (17.78 to 18.57) | 14.35 (14.11 to 14.59) | 13.43 (12.80 to 14.06) | 15.76 (15.00 to 16.51) | 13.77 (13.30 to 14.24) | 14.01 (13.71 to 14.31) | 16.95 (16.60 to 17.30) | 14.12 (13.90 to 14.34) |
| **Brunei Darussalam** | 20.26 (4.84 to 35.68) | 21.02 (6.64 to 35.41) | 19.52 (6.85 to 32.20) | 19.09 (-9.58 to 47.76) | 20.73 (-6.48 to 47.93) | 19.41 (-5.39 to 44.21) | 19.73 (5.60 to 33.87) | 20.68 (7.46 to 33.91) | 19.27 (7.54 to 31.00) |
| **Bulgaria** | 16.01 (14.55 to 17.47) | 20.87 (18.64 to 23.11) | 15.89 (14.54 to 17.24) | 12.92 (9.77 to 16.07) | 16.32 (11.19 to 21.45) | 13.78 (10.90 to 16.66) | 14.64 (13.28 to 16.00) | 18.70 (16.56 to 20.84) | 14.99 (13.72 to 16.25) |
| **Burkina Faso** | 5.81 (5.52 to 6.09) | 8.06 (7.72 to 8.40) | 6.23 (6.03 to 6.42) | 6.13 (5.69 to 6.57) | 8.73 (8.04 to 9.41) | 7.65 (7.17 to 8.14) | 6.01 (5.76 to 6.26) | 8.45 (8.11 to 8.78) | 6.96 (6.75 to 7.17) |
| **Burundi** | 5.56 (5.28 to 5.85) | 7.80 (7.34 to 8.25) | 6.26 (5.99 to 6.54) | 7.70 (6.98 to 8.42) | 9.62 (8.46 to 10.78) | 8.71 (7.83 to 9.59) | 6.67 (6.34 to 7.00) | 8.63 (8.14 to 9.13) | 7.36 (7.03 to 7.69) |
| **Cabo Verde** | 13.59 (6.69 to 20.49) | 21.58 (13.75 to 29.41) | 20.33 (13.57 to 27.10) | 13.31 (2.00 to 24.61) | 16.38 (3.51 to 29.25) | 15.65 (4.30 to 27.00) | 13.54 (7.39 to 19.68) | 19.27 (12.64 to 25.90) | 18.22 (12.44 to 23.99) |
| **Cambodia** | 8.65 (8.13 to 9.17) | 12.78 (12.02 to 13.55) | 11.50 (10.91 to 12.09) | 7.85 (7.13 to 8.57) | 11.26 (9.91 to 12.62) | 10.76 (9.60 to 11.92) | 8.28 (7.84 to 8.71) | 11.95 (11.26 to 12.63) | 11.11 (10.55 to 11.67) |
| **Cameroon** | 7.20 (6.78 to 7.62) | 8.94 (8.63 to 9.25) | 6.33 (6.18 to 6.48) | 7.40 (6.70 to 8.10) | 8.43 (7.97 to 8.89) | 7.22 (6.91 to 7.54) | 7.34 (6.95 to 7.72) | 8.73 (8.47 to 8.99) | 6.82 (6.67 to 6.97) |
| **Canada** | 28.20 (26.22 to 30.19) | 29.12 (27.11 to 31.14) | 25.99 (24.38 to 27.60) | 22.52 (18.90 to 26.14) | 24.80 (21.76 to 27.85) | 22.87 (20.30 to 25.43) | 25.31 (23.53 to 27.09) | 26.80 (25.12 to 28.47) | 24.35 (22.98 to 25.72) |
| **Central African Republic** | 4.86 (4.59 to 5.12) | 5.06 (4.85 to 5.27) | 3.95 (3.83 to 4.07) | 6.33 (5.59 to 7.07) | 6.65 (6.11 to 7.19) | 6.01 (5.59 to 6.43) | 5.63 (5.32 to 5.93) | 5.82 (5.59 to 6.05) | 4.92 (4.76 to 5.07) |
| **Chad** | 4.91 (4.59 to 5.23) | 7.08 (6.76 to 7.40) | 5.84 (5.63 to 6.05) | 4.75 (4.28 to 5.21) | 7.19 (6.61 to 7.77) | 6.72 (6.25 to 7.19) | 4.87 (4.60 to 5.14) | 7.20 (6.91 to 7.50) | 6.31 (6.10 to 6.53) |
| **Chile** | 20.27 (18.51 to 22.03) | 25.27 (22.91 to 27.63) | 20.37 (18.86 to 21.89) | 21.08 (16.99 to 25.16) | 21.13 (16.92 to 25.35) | 18.32 (15.38 to 21.27) | 20.71 (18.97 to 22.46) | 23.07 (21.01 to 25.14) | 19.34 (17.95 to 20.72) |
| **China** | 20.23 (20.00 to 20.46) | 23.41 (23.14 to 23.69) | 23.43 (23.15 to 23.71) | 14.31 (14.07 to 14.54) | 15.53 (15.16 to 15.90) | 15.66 (15.28 to 16.04) | 17.42 (17.25 to 17.58) | 19.83 (19.61 to 20.04) | 19.88 (19.67 to 20.10) |
| **Colombia** | 15.73 (14.76 to 16.71) | 16.15 (15.10 to 17.20) | 12.80 (12.26 to 13.33) | 17.14 (15.10 to 19.17) | 16.24 (14.05 to 18.43) | 13.82 (12.46 to 15.18) | 16.44 (15.52 to 17.37) | 16.16 (15.16 to 17.17) | 13.39 (12.83 to 13.95) |
| **Comoros** | 7.71 (5.53 to 9.90) | 10.98 (7.79 to 14.17) | 8.98 (6.92 to 11.03) | 10.29 (4.57 to 16.01) | 13.19 (5.69 to 20.70) | 12.26 (6.17 to 18.35) | 8.99 (6.43 to 11.56) | 12.10 (8.67 to 15.53) | 10.64 (8.14 to 13.14) |
| **Congo** | 7.28 (6.70 to 7.86) | 9.80 (9.04 to 10.57) | 7.40 (7.00 to 7.80) | 8.52 (7.33 to 9.70) | 10.11 (8.96 to 11.27) | 9.23 (8.31 to 10.15) | 7.95 (7.37 to 8.53) | 9.86 (9.20 to 10.51) | 8.30 (7.87 to 8.73) |
| **Cook Islands** | 15.82 (-4.41 to 36.05) | 20.41 (-9.06 to 49.89) | 20.65 (-9.39 to 50.70) | 10.68  (-21.44 to 42.80) | 13.06  (-40.85 to 66.98) | 13.12  (-41.07 to 67.31) | 13.82 (-3.13 to 30.77) | 17.50 (-8.17 to 43.17) | 17.58 (-8.44 to 43.60) |
| **Costa Rica** | 21.15 (15.52 to 26.79) | 22.26 (17.91 to 26.60) | 18.50 (15.62 to 21.38) | 17.74 (8.46 to 27.02) | 18.92 (11.11 to 26.74) | 17.03 (11.45 to 22.62) | 19.62 (14.79 to 24.45) | 20.68 (16.87 to 24.49) | 17.92 (15.30 to 20.55) |
| **Cote d’Ivoire** | 4.68 (4.51 to 4.85) | 6.57 (6.37 to 6.76) | 5.22 (5.11 to 5.33) | 5.02 (4.63 to 5.41) | 6.82 (6.38 to 7.25) | 6.29 (5.94 to 6.64) | 4.92 (4.75 to 5.09) | 6.72 (6.53 to 6.91) | 5.76 (5.63 to 5.89) |
| **Croatia** | 24.47 (21.34 to 27.59) | 29.32 (23.61 to 35.04) | 25.38 (21.16 to 29.60) | 17.11 (12.09 to 22.12) | 20.54 (9.95 to 31.12) | 18.70 (11.21 to 26.20) | 20.71 (18.16 to 23.26) | 24.64 (19.69 to 29.58) | 22.00 (18.34 to 25.65) |
| **Cuba** | 21.44 (19.26 to 23.62) | 25.25 (22.73 to 27.77) | 17.51 (16.23 to 18.79) | 16.83 (13.70 to 19.97) | 19.61 (15.39 to 23.83) | 15.79 (13.36 to 18.22) | 19.43 (17.64 to 21.22) | 22.61 (20.45 to 24.77) | 16.94 (15.78 to 18.10) |
| **Cyprus** | 18.94 (12.31 to 25.56) | 24.47 (15.58 to 33.36) | 22.62 (14.28 to 30.96) | 13.60 (0.81 to 26.39) | 18.12 (2.18 to 34.07) | 16.84 (1.23 to 32.44) | 16.21 (10.14 to 22.28) | 21.17 (13.40 to 28.93) | 19.69 (12.37 to 27.00) |
| **Czechia** | 24.63 (22.50 to 26.76) | 26.39 (23.33 to 29.45) | 22.25 (20.20 to 24.31) | 19.43 (15.41 to 23.45) | 21.06 (15.09 to 27.03) | 18.97 (14.85 to 23.08) | 22.10 (20.22 to 23.98) | 23.54 (20.79 to 26.29) | 20.63 (18.74 to 22.51) |
| **Democratic People's Republic of Korea** | 15.61 (14.43 to 16.79) | 15.06 (14.15 to 15.97) | 14.95 (14.04 to 15.86) | 11.35 (10.07 to 12.64) | 11.49 (10.13 to 12.84) | 11.38 (10.01 to 12.75) | 13.10 (12.25 to 13.94) | 13.00 (12.27 to 13.74) | 12.91 (12.18 to 13.65) |
| **Democratic Republic of the Congo** | 5.34 (5.23 to 5.46) | 7.08 (6.95 to 7.21) | 5.31 (5.24 to 5.37) | 6.38 (6.12 to 6.65) | 8.35 (8.03 to 8.68) | 7.55 (7.30 to 7.79) | 5.99 (5.86 to 6.12) | 7.64 (7.50 to 7.78) | 6.38 (6.30 to 6.47) |
| **Denmark** | 25.12 (21.64 to 28.59) | 31.31 (25.86 to 36.77) | 30.18 (25.09 to 35.28) | 23.44 (16.22 to 30.66) | 27.36 (17.72 to 36.99) | 26.42 (17.36 to 35.47) | 24.23 (20.88 to 27.58) | 29.21 (24.37 to 34.06) | 28.23 (23.66 to 32.79) |
| **Djibouti** | 8.58 (5.59 to 11.58) | 11.20 (8.96 to 13.44) | 8.49 (7.31 to 9.67) | 10.38 (2.88 to 17.88) | 12.33 (7.94 to 16.72) | 10.87 (7.80 to 13.94) | 9.38 (6.22 to 12.54) | 11.68 (9.58 to 13.78) | 9.51 (8.26 to 10.76) |
| **Dominica** | 20.34 (-0.54 to 41.23) | 23.02 (-0.00 to 46.04) | 17.95 (3.71 to 32.18) | 17.27  (-20.38 to 54.92) | 18.58  (-24.09 to 61.25) | 16.29  (-14.67 to 47.25) | 18.75 (-0.04 to 37.53) | 20.30 (0.10 to 40.50) | 17.01 (3.38 to 30.64) |
| **Dominican Republic** | 12.70 (11.16 to 14.23) | 14.72 (13.44 to 16.01) | 12.93 (11.99 to 13.87) | 10.79 (8.20 to 13.38) | 12.88 (10.46 to 15.30) | 11.98 (9.88 to 14.07) | 11.84 (10.50 to 13.17) | 13.92 (12.77 to 15.06) | 12.63 (11.71 to 13.54) |
| **Ecuador** | 14.43 (12.96 to 15.91) | 17.50 (16.00 to 19.01) | 11.84 (11.13 to 12.55) | 15.45 (12.39 to 18.50) | 18.41 (15.00 to 21.82) | 14.64 (12.67 to 16.60) | 14.94 (13.51 to 16.37) | 17.97 (16.45 to 19.48) | 13.26 (12.48 to 14.04) |
| **Egypt** | 6.13 (5.93 to 6.33) | 10.60 (10.29 to 10.91) | 8.46 (8.28 to 8.65) | 4.71 (4.45 to 4.97) | 8.89 (8.33 to 9.45) | 8.15 (7.76 to 8.54) | 5.44 (5.28 to 5.60) | 9.83 (9.56 to 10.10) | 8.38 (8.21 to 8.56) |
| **El Salvador** | 9.05 (8.03 to 10.08) | 12.40 (11.07 to 13.73) | 9.77 (8.92 to 10.62) | 11.96 (8.64 to 15.29) | 14.59 (10.84 to 18.34) | 12.90 (10.18 to 15.63) | 10.51 (9.31 to 11.72) | 13.65 (12.21 to 15.09) | 11.50 (10.50 to 12.50) |
| **Equatorial Guinea** | 5.19 (4.38 to 6.00) | 10.46 (8.42 to 12.49) | 7.34 (6.44 to 8.23) | 6.39 (4.20 to 8.57) | 9.38 (6.79 to 11.98) | 8.05 (6.36 to 9.74) | 5.88 (4.94 to 6.82) | 10.00 (8.37 to 11.63) | 7.82 (6.94 to 8.70) |
| **Eritrea** | 4.08 (3.84 to 4.31) | 8.39 (7.75 to 9.03) | 6.90 (6.49 to 7.32) | 8.27 (7.21 to 9.33) | 12.38 (10.53 to 14.22) | 11.38 (9.91 to 12.85) | 5.94 (5.58 to 6.29) | 10.41 (9.65 to 11.17) | 9.13 (8.59 to 9.68) |
| **Estonia** | 19.91 (16.44 to 23.39) | 27.37 (20.13 to 34.61) | 23.08 (18.08 to 28.08) | 15.72 (8.61 to 22.83) | 18.67 (4.98 to 32.37) | 16.92 (6.46 to 27.39) | 17.55 (14.49 to 20.61) | 22.29 (15.94 to 28.64) | 19.71 (15.19 to 24.24) |
| **Eswatini** | 10.27 (8.15 to 12.39) | 11.01 (9.57 to 12.44) | 6.26 (5.80 to 6.73) | 10.41 (5.90 to 14.93) | 10.92 (8.74 to 13.10) | 7.25 (6.36 to 8.13) | 10.44 (8.36 to 12.52) | 11.01 (9.77 to 12.25) | 6.77 (6.33 to 7.21) |
| **Ethiopia** | 5.04 (4.96 to 5.13) | 9.53 (9.26 to 9.81) | 6.49 (6.37 to 6.61) | 7.71 (7.48 to 7.93) | 11.50 (10.91 to 12.09) | 9.77 (9.40 to 10.14) | 6.25 (6.16 to 6.34) | 10.50 (10.22 to 10.78) | 7.98 (7.84 to 8.13) |
| **Fiji** | 6.66 (5.36 to 7.95) | 7.63 (6.23 to 9.02) | 6.00 (5.07 to 6.93) | 10.60 (4.96 to 16.23) | 11.31 (6.25 to 16.36) | 10.06 (6.23 to 13.88) | 8.53 (6.63 to 10.43) | 9.40 (7.54 to 11.27) | 7.92 (6.61 to 9.22) |
| **Finland** | 21.35 (18.97 to 23.72) | 23.91 (20.12 to 27.69) | 23.06 (19.55 to 26.58) | 18.25 (12.34 to 24.16) | 21.17 (13.03 to 29.31) | 20.43 (12.73 to 28.13) | 19.52 (17.25 to 21.79) | 22.38 (18.79 to 25.98) | 21.64 (18.29 to 24.99) |
| **France** | 32.60 (30.88 to 34.32) | 33.72 (31.64 to 35.81) | 30.80 (29.02 to 32.58) | 21.27 (18.95 to 23.59) | 24.41 (21.28 to 27.53) | 22.67 (19.95 to 25.39) | 26.78 (25.43 to 28.13) | 28.78 (27.08 to 30.49) | 26.56 (25.10 to 28.03) |
| **Gabon** | 9.20 (7.90 to 10.49) | 11.96 (10.35 to 13.56) | 9.18 (8.28 to 10.08) | 9.66 (6.53 to 12.78) | 11.00 (8.37 to 13.64) | 9.68 (7.75 to 11.60) | 9.56 (8.22 to 10.90) | 11.52 (10.10 to 12.93) | 9.52 (8.62 to 10.43) |
| **Gambia** | 5.29 (4.14 to 6.44) | 6.99 (5.93 to 8.05) | 5.72 (5.07 to 6.37) | 5.05 (3.40 to 6.69) | 6.74 (5.30 to 8.17) | 6.03 (4.98 to 7.08) | 5.19 (4.24 to 6.13) | 6.87 (6.02 to 7.73) | 5.88 (5.31 to 6.45) |
| **Georgia** | 14.30 (12.57 to 16.03) | 20.55 (17.36 to 23.74) | 16.71 (14.53 to 18.88) | 11.42 (7.39 to 15.46) | 15.73 (7.62 to 23.84) | 13.68 (8.61 to 18.75) | 12.68 (11.06 to 14.30) | 18.12 (15.09 to 21.14) | 15.28 (13.22 to 17.33) |
| **Germany** | 25.87 (24.88 to 26.86) | 27.55 (26.16 to 28.93) | 25.64 (24.44 to 26.84) | 21.18 (19.43 to 22.93) | 22.21 (19.74 to 24.67) | 20.98 (18.83 to 23.14) | 23.14 (22.28 to 23.99) | 24.68 (23.46 to 25.90) | 23.21 (22.15 to 24.27) |
| **Ghana** | 6.77 (6.47 to 7.08) | 8.21 (7.96 to 8.46) | 6.36 (6.21 to 6.50) | 6.47 (5.94 to 7.00) | 8.56 (8.08 to 9.04) | 7.67 (7.31 to 8.03) | 6.65 (6.38 to 6.93) | 8.46 (8.22 to 8.70) | 7.11 (6.95 to 7.27) |
| **Greece** | 28.49 (24.85 to 32.13) | 30.41 (26.00 to 34.83) | 27.94 (24.36 to 31.52) | 18.90 (13.01 to 24.79) | 21.11 (13.55 to 28.68) | 20.09 (13.89 to 26.29) | 23.69 (20.68 to 26.70) | 25.82 (22.08 to 29.56) | 24.17 (21.10 to 27.23) |
| **Greenland** | 26.21 (-3.11 to 55.53) | 27.92 (-5.53 to 61.36) | 25.80 (-3.56 to 55.16) | 26.74  (-22.90 to 76.37) | 25.19  (-33.52 to 83.89) | 23.67  (-28.42 to 75.75) | 26.08 (1.34 to 50.81) | 26.69 (-2.79 to 56.18) | 24.86 (-1.27 to 50.98) |
| **Grenada** | 17.04 (1.53 to 32.56) | 22.42 (4.35 to 40.48) | 15.72 (6.52 to 24.92) | 15.07  (-14.79 to 44.93) | 18.13  (-16.78 to 53.03) | 15.17 (-8.00 to 38.34) | 15.99 (1.49 to 30.50) | 19.27 (2.88 to 35.67) | 15.21 (5.58 to 24.83) |
| **Guam** | 16.97 (1.19 to 32.75) | 16.64 (3.36 to 29.92) | 13.41 (4.11 to 22.71) | 11.78 (-6.35 to 29.91) | 11.66 (-6.63 to 29.95) | 8.83 (-4.73 to 22.39) | 14.29 (3.55 to 25.03) | 14.17 (3.83 to 24.51) | 11.27 (3.76 to 18.77) |
| **Guatemala** | 7.28 (6.82 to 7.74) | 12.25 (11.38 to 13.12) | 7.94 (7.56 to 8.33) | 10.25 (8.95 to 11.55) | 13.66 (11.87 to 15.46) | 10.78 (9.67 to 11.90) | 8.69 (8.16 to 9.22) | 13.01 (12.17 to 13.86) | 9.41 (8.96 to 9.85) |
| **Guinea** | 6.33 (5.81 to 6.84) | 8.12 (7.64 to 8.60) | 6.02 (5.77 to 6.27) | 6.22 (5.52 to 6.92) | 8.23 (7.40 to 9.06) | 7.37 (6.75 to 7.98) | 6.27 (5.84 to 6.70) | 8.23 (7.78 to 8.68) | 6.74 (6.46 to 7.02) |
| **Guinea-Bissau** | 6.07 (5.23 to 6.91) | 8.09 (7.13 to 9.04) | 6.05 (5.54 to 6.56) | 6.17 (4.69 to 7.65) | 8.97 (7.14 to 10.80) | 7.87 (6.55 to 9.19) | 6.27 (5.48 to 7.07) | 8.58 (7.66 to 9.49) | 7.00 (6.43 to 7.58) |
| **Guyana** | 8.11 (6.73 to 9.49) | 10.75 (8.70 to 12.79) | 7.08 (6.09 to 8.07) | 9.74 (5.52 to 13.96) | 11.78 (6.53 to 17.04) | 9.75 (6.29 to 13.20) | 8.98 (7.41 to 10.56) | 11.32 (9.21 to 13.44) | 8.46 (7.24 to 9.68) |
| **Haiti** | 9.74 (9.10 to 10.39) | 12.83 (12.07 to 13.60) | 8.39 (8.07 to 8.71) | 10.11 (9.09 to 11.14) | 11.91 (10.82 to 13.01) | 10.15 (9.40 to 10.91) | 9.86 (9.29 to 10.43) | 12.41 (11.77 to 13.05) | 9.39 (9.04 to 9.75) |
| **Honduras** | 8.61 (7.53 to 9.69) | 11.08 (10.26 to 11.90) | 7.80 (7.39 to 8.21) | 10.63 (7.89 to 13.38) | 14.62 (12.41 to 16.84) | 12.47 (11.00 to 13.94) | 9.62 (8.41 to 10.83) | 12.92 (11.93 to 13.92) | 10.15 (9.57 to 10.72) |
| **Hungary** | 22.47 (20.83 to 24.11) | 26.20 (23.35 to 29.04) | 21.67 (19.73 to 23.60) | 18.49 (15.60 to 21.38) | 20.82 (15.47 to 26.17) | 18.51 (14.99 to 22.02) | 20.59 (19.16 to 22.01) | 23.28 (20.75 to 25.81) | 20.04 (18.33 to 21.75) |
| **Iceland** | 25.55 (4.47 to 46.63) | 29.06 (1.93 to 56.19) | 27.78 (0.17 to 55.39) | 22.88  (-19.56 to 65.32) | 24.30  (-21.50 to 70.10) | 24.49  (-20.45 to 69.43) | 24.13 (3.99 to 44.27) | 26.54 (3.02 to 50.06) | 25.84 (2.34 to 49.34) |
| **India** | 5.41 (5.37 to 5.44) | 7.90 (7.85 to 7.95) | 6.62 (6.59 to 6.66) | 5.49 (5.43 to 5.56) | 7.67 (7.57 to 7.76) | 7.01 (6.94 to 7.08) | 5.45 (5.42 to 5.48) | 7.78 (7.74 to 7.83) | 6.83 (6.80 to 6.87) |
| **Indonesia** | 7.10 (6.99 to 7.22) | 10.66 (10.50 to 10.82) | 9.03 (8.92 to 9.14) | 7.47 (7.26 to 7.68) | 10.44 (10.12 to 10.77) | 9.65 (9.39 to 9.91) | 7.28 (7.17 to 7.39) | 10.56 (10.40 to 10.71) | 9.37 (9.26 to 9.48) |
| **Iran (Islamic Republic of)** | 10.90 (10.45 to 11.34) | 15.18 (14.52 to 15.85) | 10.03 (9.76 to 10.30) | 8.13 (7.54 to 8.71) | 11.08 (10.02 to 12.15) | 8.85 (8.30 to 9.41) | 9.59 (9.25 to 9.94) | 13.31 (12.77 to 13.86) | 9.61 (9.36 to 9.87) |
| **Iraq** | 8.64 (8.10 to 9.17) | 11.05 (10.53 to 11.57) | 9.01 (8.70 to 9.31) | 6.82 (5.94 to 7.71) | 9.45 (8.37 to 10.53) | 8.49 (7.73 to 9.25) | 7.85 (7.39 to 8.31) | 10.32 (9.83 to 10.82) | 8.87 (8.56 to 9.18) |
| **Ireland** | 24.06 (19.63 to 28.49) | 28.41 (21.68 to 35.15) | 25.15 (19.51 to 30.80) | 22.07 (12.19 to 31.96) | 23.90 (12.72 to 35.08) | 21.40 (11.82 to 30.98) | 23.12 (18.70 to 27.54) | 26.02 (20.17 to 31.86) | 23.20 (18.26 to 28.15) |
| **Israel** | 22.43 (17.67 to 27.19) | 26.48 (20.94 to 32.01) | 24.13 (19.85 to 28.41) | 20.69 (11.87 to 29.52) | 23.09 (14.12 to 32.06) | 21.26 (14.04 to 28.48) | 21.61 (17.20 to 26.02) | 24.65 (19.96 to 29.35) | 22.63 (18.94 to 26.32) |
| **Italy** | 29.68 (27.97 to 31.39) | 30.64 (28.35 to 32.92) | 28.34 (26.44 to 30.24) | 20.77 (18.06 to 23.48) | 21.98 (18.40 to 25.57) | 20.85 (17.80 to 23.90) | 25.06 (23.65 to 26.48) | 25.95 (24.04 to 27.86) | 24.39 (22.78 to 25.99) |
| **Jamaica** | 18.60 (13.81 to 23.39) | 22.92 (18.56 to 27.28) | 18.53 (15.76 to 21.29) | 14.38 (7.76 to 21.00) | 16.85 (10.49 to 23.20) | 15.21 (10.20 to 20.21) | 16.35 (12.44 to 20.27) | 19.84 (16.20 to 23.48) | 17.05 (14.48 to 19.62) |
| **Japan** | 29.00 (27.82 to 30.17) | 32.42 (31.17 to 33.67) | 32.34 (31.08 to 33.59) | 19.68 (18.06 to 21.31) | 23.70 (21.58 to 25.81) | 23.36 (21.24 to 25.48) | 24.11 (23.18 to 25.05) | 27.87 (26.78 to 28.95) | 27.70 (26.61 to 28.79) |
| **Jordan** | 11.26 (8.52 to 13.99) | 12.85 (10.49 to 15.21) | 9.13 (8.23 to 10.03) | 8.25 (5.20 to 11.30) | 10.24 (6.53 to 13.95) | 8.76 (6.90 to 10.63) | 9.61 (7.67 to 11.55) | 11.60 (9.70 to 13.50) | 8.98 (8.16 to 9.81) |
| **Kazakhstan** | 21.90 (20.28 to 23.51) | 12.38 (11.66 to 13.10) | 9.42 (9.01 to 9.82) | 16.31 (13.77 to 18.84) | 10.20 (8.55 to 11.85) | 8.66 (7.71 to 9.61) | 18.77 (17.46 to 20.08) | 11.21 (10.53 to 11.89) | 9.06 (8.66 to 9.46) |
| **Kenya** | 4.85 (4.65 to 5.05) | 7.34 (7.13 to 7.54) | 5.35 (5.25 to 5.45) | 7.08 (6.56 to 7.60) | 10.42 (9.86 to 10.99) | 8.89 (8.51 to 9.27) | 5.93 (5.70 to 6.16) | 8.83 (8.59 to 9.06) | 6.98 (6.84 to 7.11) |
| **Kiribati** | 6.98 (3.34 to 10.62) | 8.37 (4.47 to 12.26) | 8.47 (4.55 to 12.39) | 9.31 (-2.46 to 21.07) | 11.24 (-0.73 to 23.20) | 11.30 (-0.69 to 23.29) | 8.12 (3.64 to 12.61) | 9.80 (5.19 to 14.41) | 9.88 (5.26 to 14.50) |
| **Kuwait** | 10.33 (6.99 to 13.68) | 12.34 (9.61 to 15.07) | 10.87 (9.04 to 12.69) | 10.59 (3.59 to 17.59) | 9.81 (1.91 to 17.71) | 9.22 (4.07 to 14.36) | 10.25 (7.30 to 13.20) | 11.67 (8.93 to 14.41) | 10.47 (8.64 to 12.31) |
| **Kyrgyzstan** | 14.96 (12.88 to 17.04) | 11.41 (9.93 to 12.88) | 9.77 (8.74 to 10.80) | 11.79 (8.46 to 15.12) | 9.96 (6.61 to 13.30) | 9.14 (6.70 to 11.59) | 13.19 (11.48 to 14.91) | 10.63 (9.23 to 12.02) | 9.45 (8.44 to 10.46) |
| **Lao People's Democratic Republic** | 7.19 (6.64 to 7.74) | 10.56 (9.57 to 11.54) | 9.54 (8.78 to 10.31) | 6.92 (6.07 to 7.77) | 10.11 (8.19 to 12.03) | 9.65 (7.99 to 11.30) | 7.12 (6.64 to 7.60) | 10.38 (9.45 to 11.31) | 9.65 (8.89 to 10.41) |
| **Latvia** | 18.50 (16.16 to 20.84) | 23.38 (18.98 to 27.78) | 18.74 (15.67 to 21.81) | 14.00 (9.53 to 18.46) | 16.97 (8.73 to 25.22) | 15.06 (9.19 to 20.93) | 15.99 (13.98 to 18.00) | 19.66 (15.85 to 23.47) | 16.80 (14.12 to 19.48) |
| **Lebanon** | 14.41 (12.45 to 16.36) | 22.27 (17.90 to 26.64) | 15.09 (13.42 to 16.75) | 12.37 (8.77 to 15.97) | 15.85 (9.23 to 22.46) | 12.61 (9.58 to 15.63) | 13.63 (11.87 to 15.40) | 19.44 (15.78 to 23.10) | 14.25 (12.73 to 15.76) |
| **Lesotho** | 8.10 (7.16 to 9.04) | 7.93 (7.37 to 8.49) | 4.78 (4.57 to 4.99) | 8.05 (6.16 to 9.94) | 8.93 (8.02 to 9.84) | 6.48 (6.01 to 6.96) | 8.19 (7.25 to 9.13) | 8.42 (7.92 to 8.93) | 5.61 (5.39 to 5.83) |
| **Liberia** | 5.26 (4.67 to 5.85) | 8.85 (7.98 to 9.73) | 7.08 (6.57 to 7.59) | 5.14 (4.35 to 5.92) | 8.00 (6.88 to 9.12) | 7.42 (6.52 to 8.32) | 5.28 (4.80 to 5.75) | 8.43 (7.75 to 9.11) | 7.27 (6.80 to 7.73) |
| **Libya** | 17.87 (13.79 to 21.94) | 18.25 (15.84 to 20.67) | 15.13 (13.63 to 16.64) | 11.87 (7.44 to 16.30) | 13.08 (10.33 to 15.82) | 11.83 (9.65 to 14.01) | 15.17 (12.25 to 18.09) | 15.98 (14.22 to 17.75) | 13.81 (12.58 to 15.04) |
| **Lithuania** | 19.40 (17.01 to 21.79) | 23.93 (19.97 to 27.89) | 20.01 (17.22 to 22.80) | 14.35 (9.97 to 18.74) | 16.98 (9.35 to 24.61) | 15.16 (9.81 to 20.52) | 16.76 (14.72 to 18.79) | 20.11 (16.64 to 23.58) | 17.51 (15.05 to 19.98) |
| **Luxembourg** | 28.87 (12.34 to 45.41) | 30.00 (9.73 to 50.27) | 27.22 (10.89 to 43.55) | 22.17 (-4.64 to 48.99) | 23.85 (-6.77 to 54.46) | 21.85 (-4.28 to 47.98) | 25.34 (11.46 to 39.21) | 26.60 (9.94 to 43.27) | 24.34 (10.51 to 38.17) |
| **Madagascar** | 5.98 (5.71 to 6.25) | 6.85 (6.57 to 7.13) | 5.15 (5.00 to 5.29) | 8.33 (7.52 to 9.13) | 9.12 (8.44 to 9.80) | 8.24 (7.73 to 8.74) | 7.14 (6.80 to 7.49) | 7.98 (7.66 to 8.29) | 6.66 (6.46 to 6.86) |
| **Malawi** | 4.69 (4.49 to 4.89) | 8.57 (8.19 to 8.96) | 5.59 (5.43 to 5.74) | 6.59 (6.11 to 7.07) | 11.00 (10.10 to 11.90) | 8.62 (8.13 to 9.11) | 5.64 (5.42 to 5.87) | 9.77 (9.36 to 10.18) | 7.01 (6.82 to 7.20) |
| **Malaysia** | 12.21 (11.28 to 13.14) | 13.94 (13.17 to 14.71) | 12.02 (11.49 to 12.55) | 11.72 (9.78 to 13.66) | 13.88 (12.07 to 15.69) | 12.79 (11.41 to 14.18) | 12.02 (11.12 to 12.91) | 13.97 (13.21 to 14.72) | 12.45 (11.90 to 12.99) |
| **Maldives** | 8.16 (3.70 to 12.62) | 10.21 (3.49 to 16.93) | 8.92 (4.41 to 13.44) | 6.56 (1.68 to 11.45) | 7.77 (-4.12 to 19.66) | 7.27 (-1.76 to 16.31) | 7.62 (4.27 to 10.98) | 9.13 (3.51 to 14.75) | 8.20 (4.28 to 12.12) |
| **Mali** | 7.10 (6.62 to 7.57) | 10.23 (9.63 to 10.82) | 6.80 (6.57 to 7.03) | 6.39 (5.87 to 6.92) | 8.65 (7.99 to 9.31) | 7.34 (6.92 to 7.77) | 6.73 (6.38 to 7.09) | 9.52 (9.07 to 9.96) | 7.14 (6.92 to 7.36) |
| **Malta** | 23.33 (7.42 to 39.24) | 24.69 (3.11 to 46.28) | 22.82 (3.74 to 41.91) | 18.34 (-9.92 to 46.59) | 18.15  (-17.72 to 54.01) | 18.54  (-14.00 to 51.09) | 20.80 (6.44 to 35.17) | 21.21 (2.57 to 39.84) | 20.42 (4.22 to 36.61) |
| **Marshall Islands** | 8.04 (2.08 to 14.00) | 9.99 (3.24 to 16.74) | 10.17 (3.32 to 17.02) | 8.53 (-6.09 to 23.15) | 9.84 (-2.50 to 22.18) | 9.93 (-2.57 to 22.44) | 8.32 (2.12 to 14.52) | 10.02 (3.86 to 16.18) | 10.16 (3.92 to 16.40) |
| **Mauritania** | 10.05 (8.31 to 11.79) | 12.69 (10.75 to 14.64) | 10.71 (9.48 to 11.93) | 7.57 (6.32 to 8.82) | 10.21 (8.17 to 12.24) | 9.77 (8.05 to 11.49) | 8.67 (7.64 to 9.70) | 11.46 (10.06 to 12.86) | 10.34 (9.31 to 11.37) |
| **Mauritius** | 9.46 (7.71 to 11.21) | 12.69 (10.22 to 15.16) | 11.44 (9.52 to 13.37) | 10.52 (5.40 to 15.64) | 12.11 (6.15 to 18.07) | 11.40 (6.39 to 16.42) | 9.97 (8.08 to 11.86) | 12.34 (9.92 to 14.76) | 11.42 (9.44 to 13.39) |
| **Mexico** | 12.17 (11.78 to 12.56) | 12.20 (11.87 to 12.54) | 8.56 (8.38 to 8.73) | 13.88 (12.92 to 14.84) | 12.31 (11.56 to 13.06) | 10.15 (9.76 to 10.55) | 13.05 (12.65 to 13.44) | 12.32 (12.00 to 12.64) | 9.42 (9.25 to 9.58) |
| **Micronesia (Federated States of)** | 9.54 (4.57 to 14.51) | 11.49 (5.24 to 17.74) | 11.59 (5.37 to 17.82) | 10.00 (-1.22 to 21.23) | 11.67 (-1.36 to 24.70) | 11.71 (-1.28 to 24.69) | 9.74 (4.84 to 14.64) | 11.49 (5.64 to 17.34) | 11.56 (5.73 to 17.39) |
| **Monaco** | 33.28  (-48.97 to 115.53) | 41.04  (-64.87 to 146.96) | 36.22  (-45.04 to 117.48) | 24.25  (-109.05 to 157.55) | 33.39  (-135.87 to 202.66) | 31.10  (-108.40 to 170.60) | 28.65  (-41.68 to 98.97) | 37.21  (-53.59 to 128.01) | 33.79  (-38.25 to 105.84) |
| **Mongolia** | 20.01 (16.21 to 23.81) | 24.26 (20.07 to 28.46) | 22.21 (18.83 to 25.60) | 17.00 (11.45 to 22.55) | 23.95 (15.92 to 31.98) | 21.89 (15.42 to 28.36) | 18.62 (15.43 to 21.82) | 24.15 (20.28 to 28.01) | 22.07 (18.96 to 25.19) |
| **Montenegro** | 22.51 (10.84 to 34.18) | 25.09 (12.97 to 37.21) | 19.23 (11.88 to 26.57) | 14.83 (-3.75 to 33.42) | 17.33 (-4.84 to 39.50) | 14.54 (-0.76 to 29.84) | 18.72 (9.17 to 28.27) | 21.30 (10.79 to 31.81) | 17.11 (10.58 to 23.64) |
| **Morocco** | 6.37 (6.07 to 6.68) | 8.51 (8.15 to 8.87) | 7.35 (7.11 to 7.59) | 4.02 (3.73 to 4.32) | 5.49 (5.05 to 5.94) | 5.17 (4.80 to 5.54) | 5.30 (5.08 to 5.51) | 7.06 (6.78 to 7.34) | 6.37 (6.16 to 6.58) |
| **Mozambique** | 5.08 (4.90 to 5.26) | 7.11 (6.92 to 7.30) | 5.03 (4.94 to 5.13) | 7.27 (6.71 to 7.82) | 9.84 (9.31 to 10.37) | 7.90 (7.59 to 8.21) | 6.16 (5.93 to 6.38) | 8.45 (8.23 to 8.66) | 6.38 (6.26 to 6.49) |
| **Myanmar** | 6.13 (6.01 to 6.26) | 8.76 (8.53 to 8.99) | 7.66 (7.49 to 7.83) | 7.82 (7.44 to 8.21) | 9.74 (9.07 to 10.40) | 9.13 (8.58 to 9.68) | 6.99 (6.84 to 7.15) | 9.30 (9.03 to 9.56) | 8.45 (8.24 to 8.65) |
| **Namibia** | 5.48 (4.82 to 6.13) | 7.90 (7.15 to 8.66) | 4.93 (4.61 to 5.24) | 6.84 (4.94 to 8.75) | 9.23 (7.49 to 10.98) | 7.11 (6.18 to 8.04) | 6.16 (5.40 to 6.91) | 8.61 (7.81 to 9.40) | 6.02 (5.65 to 6.40) |
| **Nauru** | 9.58 (-3.32 to 22.47) | 10.10 (-3.91 to 24.11) | 10.29 (-4.16 to 24.74) | 11.48  (-26.18 to 49.14) | 11.80  (-23.12 to 46.71) | 11.95  (-23.79 to 47.69) | 10.58 (-3.70 to 24.85) | 10.99 (-3.80 to 25.78) | 11.15 (-4.02 to 26.32) |
| **Nepal** | 5.04 (4.83 to 5.24) | 7.59 (7.22 to 7.96) | 5.87 (5.66 to 6.09) | 5.29 (4.94 to 5.64) | 7.19 (6.58 to 7.81) | 6.33 (5.92 to 6.74) | 5.17 (4.98 to 5.35) | 7.45 (7.12 to 7.79) | 6.16 (5.96 to 6.37) |
| **Netherlands** | 30.76 (27.77 to 33.76) | 31.19 (27.32 to 35.07) | 29.14 (25.85 to 32.43) | 22.46 (17.36 to 27.56) | 24.13 (18.42 to 29.83) | 22.83 (17.80 to 27.87) | 26.47 (23.84 to 29.10) | 27.40 (24.18 to 30.61) | 25.88 (23.09 to 28.68) |
| **New Zealand** | 25.24 (20.54 to 29.95) | 28.30 (22.33 to 34.27) | 28.04 (22.06 to 34.02) | 22.43 (12.75 to 32.10) | 22.99 (13.53 to 32.44) | 22.65 (13.14 to 32.17) | 23.72 (19.17 to 28.27) | 25.53 (20.43 to 30.63) | 25.22 (20.10 to 30.35) |
| **Nicaragua** | 10.49 (8.34 to 12.64) | 11.96 (10.09 to 13.82) | 6.95 (6.26 to 7.65) | 10.89 (5.55 to 16.24) | 11.82 (7.77 to 15.86) | 8.61 (6.67 to 10.55) | 10.63 (8.31 to 12.96) | 11.86 (10.07 to 13.65) | 7.84 (7.07 to 8.61) |
| **Niger** | 4.60 (4.33 to 4.88) | 6.97 (6.66 to 7.27) | 5.38 (5.22 to 5.55) | 4.38 (3.96 to 4.79) | 6.81 (6.32 to 7.29) | 6.18 (5.82 to 6.54) | 4.50 (4.26 to 4.73) | 6.94 (6.67 to 7.21) | 5.84 (5.66 to 6.01) |
| **Nigeria** | 5.13 (5.06 to 5.21) | 8.50 (8.38 to 8.63) | 6.35 (6.29 to 6.42) | 4.97 (4.82 to 5.13) | 7.97 (7.76 to 8.17) | 7.32 (7.17 to 7.48) | 4.90 (4.83 to 4.96) | 8.41 (8.30 to 8.53) | 7.05 (6.98 to 7.12) |
| **Niue** | 10.85  (-26.24 to 47.95) | 12.77  (-37.38 to 62.92) | 12.49  (-36.07 to 61.06) | 11.00  (-88.81 to 110.80) | 12.37  (-118.74 to 143.48) | 12.07  (-112.18 to 136.32) | 10.83  (-28.32 to 49.99) | 12.53  (-39.36 to 64.42) | 12.24  (-39.96 to 64.45) |
| **North Macedonia** | 18.93 (13.89 to 23.97) | 22.75 (15.58 to 29.92) | 16.05 (12.74 to 19.37) | 13.87 (5.90 to 21.84) | 16.26 (5.19 to 27.32) | 13.47 (7.67 to 19.27) | 16.59 (12.35 to 20.83) | 19.57 (13.73 to 25.41) | 14.92 (12.03 to 17.81) |
| **Northern Mariana Islands** | 17.23 (-10.01 to 44.46) | 18.73 (-1.27 to 38.73) | 16.68 (1.89 to 31.46) | 15.08 (-27.41 to 57.57) | 17.31 (-21.07 to 55.68) | 16.29 (-15.91 to 48.50) | 16.14 (-5.65 to 37.92) | 18.13 (0.18 to 36.08) | 16.54 (2.49 to 30.59) |
| **Norway** | 23.82 (20.15 to 27.48) | 30.18 (23.59 to 36.76) | 29.35 (23.04 to 35.67) | 20.45 (11.81 to 29.09) | 24.40 (13.93 to 34.87) | 23.88 (13.75 to 34.01) | 22.11 (18.47 to 25.75) | 26.99 (21.41 to 32.56) | 26.35 (20.96 to 31.73) |
| **Oman** | 6.03 (5.14 to 6.93) | 7.43 (6.33 to 8.53) | 5.30 (4.80 to 5.80) | 4.82 (3.17 to 6.47) | 5.26 (3.32 to 7.21) | 4.32 (3.35 to 5.29) | 5.47 (4.71 to 6.22) | 6.50 (5.59 to 7.40) | 4.90 (4.47 to 5.33) |
| **Pakistan** | 9.25 (9.00 to 9.50) | 11.83 (11.56 to 12.09) | 9.22 (9.07 to 9.36) | 8.74 (8.42 to 9.07) | 12.33 (11.92 to 12.73) | 11.07 (10.77 to 11.36) | 9.08 (8.88 to 9.28) | 12.08 (11.86 to 12.31) | 10.08 (9.94 to 10.23) |
| **Palau** | 13.06 (-6.81 to 32.92) | 13.64 (-4.64 to 31.93) | 13.71 (-4.19 to 31.61) | 20.77  (-50.34 to 91.89) | 21.17  (-37.09 to 79.42) | 21.16  (-36.95 to 79.28) | 16.57 (-8.33 to 41.47) | 17.14 (-2.50 to 36.79) | 17.13 (-2.11 to 36.37) |
| **Palestine** | 13.68 (10.11 to 17.25) | 16.16 (12.63 to 19.69) | 13.17 (11.23 to 15.12) | 10.50 (4.71 to 16.28) | 12.41 (6.09 to 18.73) | 11.26 (7.26 to 15.25) | 12.29 (9.17 to 15.41) | 14.14 (11.05 to 17.23) | 12.25 (10.44 to 14.06) |
| **Panama** | 16.12 (11.98 to 20.26) | 17.55 (14.21 to 20.89) | 15.02 (12.66 to 17.38) | 14.34 (5.78 to 22.89) | 15.47 (8.35 to 22.59) | 14.25 (8.59 to 19.92) | 15.33 (11.39 to 19.27) | 16.60 (13.39 to 19.81) | 14.75 (12.35 to 17.14) |
| **Papua New Guinea** | 6.45 (5.94 to 6.97) | 7.89 (7.41 to 8.36) | 5.74 (5.51 to 5.97) | 6.48 (5.43 to 7.53) | 7.44 (6.47 to 8.42) | 6.62 (5.95 to 7.30) | 6.52 (6.01 to 7.02) | 7.77 (7.30 to 8.24) | 6.25 (5.98 to 6.53) |
| **Paraguay** | 12.64 (10.13 to 15.15) | 17.40 (15.33 to 19.47) | 12.35 (11.33 to 13.37) | 11.71 (6.93 to 16.49) | 15.36 (10.94 to 19.78) | 12.40 (9.78 to 15.03) | 12.13 (9.77 to 14.49) | 16.49 (14.52 to 18.47) | 12.51 (11.44 to 13.58) |
| **Peru** | 15.19 (14.05 to 16.33) | 18.56 (17.29 to 19.84) | 9.63 (9.26 to 10.00) | 16.03 (13.71 to 18.35) | 19.54 (16.97 to 22.12) | 13.48 (12.43 to 14.53) | 15.66 (14.55 to 16.77) | 19.06 (17.82 to 20.31) | 11.49 (11.07 to 11.90) |
| **Philippines** | 9.59 (9.23 to 9.94) | 10.42 (10.17 to 10.67) | 7.94 (7.80 to 8.07) | 9.34 (8.58 to 10.10) | 10.91 (10.25 to 11.58) | 9.40 (8.98 to 9.82) | 9.53 (9.20 to 9.87) | 10.65 (10.39 to 10.91) | 8.65 (8.50 to 8.80) |
| **Poland** | 22.05 (21.06 to 23.03) | 29.54 (28.04 to 31.04) | 24.68 (23.65 to 25.72) | 17.45 (15.41 to 19.50) | 23.83 (20.69 to 26.96) | 21.25 (19.11 to 23.39) | 19.77 (18.89 to 20.66) | 26.43 (25.10 to 27.77) | 22.96 (22.03 to 23.89) |
| **Portugal** | 22.65 (20.33 to 24.97) | 29.00 (24.64 to 33.36) | 26.70 (22.98 to 30.43) | 18.21 (13.91 to 22.51) | 19.22 (12.68 to 25.77) | 17.98 (12.30 to 23.66) | 20.41 (18.36 to 22.46) | 23.89 (20.33 to 27.45) | 22.22 (19.15 to 25.29) |
| **Puerto Rico** | 18.01 (15.47 to 20.56) | 17.64 (14.42 to 20.86) | 16.72 (13.86 to 19.59) | 14.56 (9.84 to 19.28) | 15.05 (8.48 to 21.62) | 14.53 (8.55 to 20.51) | 16.63 (14.42 to 18.84) | 16.33 (13.42 to 19.24) | 15.66 (13.04 to 18.27) |
| **Qatar** | 13.09 (7.42 to 18.75) | 16.69 (10.05 to 23.34) | 15.56 (11.42 to 19.70) | 14.06 (-6.31 to 34.43) | 16.16 (0.59 to 31.73) | 15.09 (4.00 to 26.18) | 13.30 (7.45 to 19.15) | 15.87 (10.44 to 21.30) | 15.11 (11.37 to 18.85) |
| **Republic of Korea** | 26.28 (24.88 to 27.69) | 30.40 (28.45 to 32.35) | 29.71 (27.90 to 31.52) | 17.06 (15.15 to 18.97) | 18.74 (16.06 to 21.42) | 18.51 (16.00 to 21.02) | 21.44 (20.35 to 22.53) | 23.91 (22.39 to 25.44) | 23.59 (22.16 to 25.02) |
| **Republic of Moldova** | 16.47 (14.40 to 18.54) | 16.19 (13.97 to 18.40) | 14.23 (12.62 to 15.84) | 11.75 (8.84 to 14.66) | 10.77 (7.07 to 14.47) | 10.01 (7.26 to 12.76) | 13.93 (12.34 to 15.52) | 13.33 (11.52 to 15.13) | 12.10 (10.74 to 13.46) |
| **Romania** | 13.84 (12.98 to 14.70) | 21.82 (20.29 to 23.35) | 18.47 (17.39 to 19.55) | 10.87 (9.14 to 12.61) | 16.04 (12.82 to 19.25) | 14.61 (12.44 to 16.77) | 12.38 (11.60 to 13.16) | 18.95 (17.56 to 20.34) | 16.65 (15.68 to 17.62) |
| **Russian Federation** | 19.45 (19.07 to 19.84) | 17.98 (17.65 to 18.30) | 13.76 (13.56 to 13.95) | 13.63 (12.92 to 14.34) | 14.49 (13.88 to 15.11) | 12.17 (11.79 to 12.54) | 16.01 (15.69 to 16.33) | 15.99 (15.70 to 16.27) | 12.89 (12.72 to 13.07) |
| **Rwanda** | 6.41 (6.09 to 6.73) | 10.78 (9.99 to 11.58) | 7.79 (7.41 to 8.17) | 9.40 (8.52 to 10.28) | 12.41 (10.84 to 13.97) | 10.67 (9.64 to 11.70) | 7.96 (7.57 to 8.36) | 11.63 (10.84 to 12.42) | 9.30 (8.85 to 9.75) |
| **Saint Kitts and Nevis** | 14.90 (0.05 to 29.74) | 20.65 (2.88 to 38.43) | 17.44 (4.78 to 30.10) | 15.84  (-24.26 to 55.93) | 16.36  (-19.49 to 52.21) | 14.99  (-12.62 to 42.59) | 15.33 (-1.58 to 32.24) | 18.52 (2.60 to 34.45) | 16.36 (4.45 to 28.27) |
| **Saint Lucia** | 18.60 (5.28 to 31.92) | 23.41 (8.76 to 38.07) | 17.32 (8.96 to 25.68) | 15.28 (-9.25 to 39.82) | 15.45  (-10.22 to 41.12) | 13.92 (-3.52 to 31.37) | 16.95 (4.70 to 29.20) | 20.18 (7.25 to 33.12) | 16.07 (8.14 to 23.99) |
| **Saint Vincent and the Grenadines** | 17.24 (2.13 to 32.34) | 20.80 (6.25 to 35.36) | 18.32 (6.92 to 29.73) | 15.52  (-14.29 to 45.33) | 16.00  (-12.73 to 44.74) | 15.03  (-10.18 to 40.24) | 16.07 (2.09 to 30.04) | 18.76 (5.24 to 32.27) | 17.21 (5.92 to 28.49) |
| **Samoa** | 8.74 (4.27 to 13.20) | 8.98 (4.78 to 13.19) | 9.16 (4.70 to 13.62) | 9.15 (-2.31 to 20.61) | 9.80 (0.37 to 19.22) | 9.97 (0.04 to 19.89) | 8.92 (4.19 to 13.66) | 9.31 (5.06 to 13.55) | 9.48 (4.98 to 13.98) |
| **San Marino** | 38.36  (-84.88 to 161.59) | 38.78  (-90.22 to 167.77) | 26.71  (-74.66 to 128.09) | 24.41  (-135.96 to 184.77) | 22.62 (-141.42 to 186.65) | 17.43  (-113.94 to 148.80) | 31.48  (-63.15 to 126.11) | 31.71  (-68.10 to 131.52) | 22.15  (-56.10 to 100.41) |
| **Sao Tome and Principe** | 8.57 (4.07 to 13.08) | 12.44 (6.15 to 18.74) | 10.76 (6.35 to 15.18) | 7.42 (-0.54 to 15.38) | 10.27 (-0.80 to 21.35) | 9.62 (0.59 to 18.65) | 7.84 (3.69 to 11.98) | 11.33 (5.67 to 16.98) | 10.22 (5.96 to 14.49) |
| **Saudi Arabia** | 6.21 (5.87 to 6.55) | 7.89 (7.52 to 8.26) | 7.25 (6.94 to 7.55) | 5.11 (4.60 to 5.63) | 7.04 (6.44 to 7.65) | 6.70 (6.16 to 7.24) | 5.73 (5.45 to 6.00) | 7.56 (7.26 to 7.87) | 7.07 (6.81 to 7.33) |
| **Senegal** | 6.37 (5.96 to 6.78) | 10.16 (9.58 to 10.74) | 7.35 (7.07 to 7.63) | 6.04 (5.37 to 6.72) | 9.30 (8.35 to 10.25) | 8.09 (7.44 to 8.74) | 6.30 (5.93 to 6.67) | 9.80 (9.28 to 10.31) | 7.81 (7.51 to 8.11) |
| **Serbia** | 19.91 (17.92 to 21.91) | 23.89 (20.78 to 26.99) | 20.43 (18.22 to 22.63) | 15.53 (11.86 to 19.19) | 18.00 (12.17 to 23.84) | 16.63 (12.25 to 21.01) | 17.75 (15.99 to 19.52) | 20.93 (18.17 to 23.69) | 18.61 (16.60 to 20.62) |
| **Seychelles** | 17.25 (2.20 to 32.29) | 22.36 (4.60 to 40.12) | 19.31 (5.77 to 32.85) | 13.99  (-24.76 to 52.74) | 16.32  (-18.92 to 51.56) | 14.19  (-12.43 to 40.81) | 15.90 (0.78 to 31.02) | 19.35 (3.74 to 34.97) | 16.83 (4.60 to 29.07) |
| **Sierra Leone** | 5.14 (4.77 to 5.52) | 7.01 (6.58 to 7.44) | 5.88 (5.60 to 6.16) | 4.64 (4.06 to 5.22) | 7.06 (6.34 to 7.78) | 6.62 (6.03 to 7.22) | 5.00 (4.67 to 5.32) | 7.00 (6.62 to 7.39) | 6.24 (5.96 to 6.53) |
| **Singapore** | 21.24 (16.20 to 26.27) | 22.43 (16.51 to 28.35) | 21.32 (15.99 to 26.65) | 16.29 (8.73 to 23.85) | 17.80 (7.38 to 28.22) | 16.94 (7.33 to 26.54) | 18.74 (14.58 to 22.89) | 19.83 (14.65 to 25.01) | 18.90 (14.20 to 23.61) |
| **Slovakia** | 22.83 (20.16 to 25.50) | 25.98 (21.96 to 30.00) | 20.88 (18.41 to 23.35) | 16.67 (11.62 to 21.71) | 18.67 (11.29 to 26.06) | 16.27 (11.45 to 21.09) | 19.98 (17.65 to 22.31) | 22.04 (18.57 to 25.50) | 18.57 (16.36 to 20.77) |
| **Slovenia** | 23.89 (18.73 to 29.05) | 31.49 (22.86 to 40.12) | 27.85 (21.06 to 34.64) | 17.85 (9.38 to 26.32) | 22.30 (6.34 to 38.26) | 20.09 (8.15 to 32.02) | 20.63 (16.39 to 24.87) | 26.45 (19.06 to 33.84) | 23.77 (18.12 to 29.42) |
| **Solomon Islands** | 7.30 (5.45 to 9.14) | 8.86 (6.89 to 10.83) | 8.83 (6.92 to 10.75) | 7.06 (3.63 to 10.48) | 9.19 (4.98 to 13.40) | 9.11 (5.09 to 13.14) | 7.36 (5.65 to 9.06) | 9.11 (7.17 to 11.05) | 9.05 (7.18 to 10.92) |
| **Somalia** | 5.30 (5.06 to 5.54) | 6.40 (6.16 to 6.63) | 4.15 (4.06 to 4.25) | 7.63 (6.96 to 8.30) | 9.17 (8.55 to 9.79) | 7.39 (7.03 to 7.74) | 6.42 (6.14 to 6.70) | 7.78 (7.51 to 8.05) | 5.70 (5.57 to 5.83) |
| **South Africa** | 12.65 (12.08 to 13.23) | 13.53 (13.18 to 13.87) | 8.41 (8.29 to 8.54) | 10.63 (9.87 to 11.40) | 12.09 (11.63 to 12.55) | 9.44 (9.19 to 9.69) | 11.63 (11.18 to 12.08) | 12.67 (12.40 to 12.94) | 8.97 (8.85 to 9.09) |
| **South Sudan** | 6.35 (5.97 to 6.73) | 7.46 (7.04 to 7.87) | 5.81 (5.58 to 6.05) | 8.11 (6.92 to 9.31) | 9.26 (8.26 to 10.26) | 8.40 (7.65 to 9.15) | 7.10 (6.66 to 7.53) | 8.35 (7.91 to 8.79) | 7.01 (6.72 to 7.30) |
| **Spain** | 27.71 (25.89 to 29.53) | 30.81 (28.32 to 33.29) | 28.36 (26.24 to 30.48) | 18.02 (15.18 to 20.87) | 19.20 (15.53 to 22.86) | 17.91 (14.78 to 21.03) | 22.76 (21.28 to 24.23) | 24.76 (22.74 to 26.78) | 23.00 (21.28 to 24.72) |
| **Sri Lanka** | 7.71 (7.32 to 8.09) | 9.21 (8.63 to 9.80) | 8.06 (7.61 to 8.52) | 8.86 (7.64 to 10.08) | 8.64 (7.20 to 10.08) | 7.91 (6.79 to 9.03) | 8.30 (7.88 to 8.73) | 8.92 (8.34 to 9.49) | 7.99 (7.54 to 8.45) |
| **Sudan** | 6.47 (6.18 to 6.77) | 9.52 (9.04 to 9.99) | 7.29 (7.03 to 7.55) | 5.46 (5.09 to 5.83) | 7.56 (6.97 to 8.16) | 6.73 (6.30 to 7.16) | 6.04 (5.81 to 6.27) | 8.75 (8.37 to 9.13) | 7.18 (6.94 to 7.41) |
| **Suriname** | 12.55 (7.59 to 17.51) | 16.33 (11.06 to 21.61) | 11.89 (8.73 to 15.05) | 12.05 (2.49 to 21.61) | 14.05 (4.58 to 23.52) | 11.61 (4.79 to 18.44) | 12.28 (7.78 to 16.78) | 15.26 (10.60 to 19.91) | 11.87 (8.78 to 14.96) |
| **Sweden** | 22.81 (20.07 to 25.56) | 27.42 (23.41 to 31.43) | 25.50 (21.93 to 29.08) | 20.83 (14.63 to 27.03) | 24.03 (16.80 to 31.27) | 22.79 (16.20 to 29.38) | 21.77 (19.04 to 24.50) | 25.55 (21.91 to 29.19) | 23.99 (20.70 to 27.27) |
| **Switzerland** | 25.31 (21.77 to 28.84) | 27.93 (22.57 to 33.28) | 26.52 (21.79 to 31.26) | 18.02 (12.07 to 23.96) | 20.65 (12.54 to 28.76) | 19.42 (12.06 to 26.78) | 21.45 (18.43 to 24.46) | 23.86 (19.44 to 28.27) | 22.68 (18.72 to 26.63) |
| **Syrian Arab Republic** | 8.24 (7.51 to 8.97) | 7.92 (7.47 to 8.37) | 7.67 (7.24 to 8.11) | 6.72 (5.64 to 7.80) | 7.29 (6.38 to 8.20) | 6.92 (6.00 to 7.83) | 7.59 (6.98 to 8.20) | 7.89 (7.46 to 8.32) | 7.46 (7.03 to 7.89) |
| **Taiwan (Province of China)** | 18.62 (17.03 to 20.21) | 28.04 (25.40 to 30.69) | 27.62 (25.04 to 30.20) | 13.61 (11.14 to 16.09) | 20.98 (16.96 to 25.00) | 20.26 (16.38 to 24.14) | 16.41 (15.10 to 17.71) | 24.78 (22.60 to 26.95) | 24.15 (22.04 to 26.27) |
| **Tajikistan** | 12.74 (10.71 to 14.76) | 9.53 (8.00 to 11.06) | 7.15 (6.44 to 7.87) | 9.70 (7.17 to 12.23) | 7.86 (5.75 to 9.98) | 6.83 (5.64 to 8.03) | 11.12 (9.57 to 12.68) | 8.73 (7.51 to 9.95) | 7.04 (6.42 to 7.67) |
| **Thailand** | 17.33 (16.57 to 18.08) | 19.33 (18.57 to 20.09) | 18.13 (17.51 to 18.75) | 14.56 (13.39 to 15.73) | 16.24 (14.98 to 17.50) | 15.57 (14.46 to 16.68) | 15.99 (15.36 to 16.62) | 17.82 (17.18 to 18.45) | 16.93 (16.39 to 17.47) |
| **Timor-Leste** | 6.08 (4.37 to 7.78) | 8.07 (6.38 to 9.76) | 6.91 (5.72 to 8.10) | 6.22 (4.02 to 8.42) | 8.64 (5.10 to 12.18) | 8.01 (5.17 to 10.86) | 6.11 (4.76 to 7.46) | 8.37 (6.69 to 10.05) | 7.46 (6.20 to 8.72) |
| **Togo** | 5.47 (4.97 to 5.97) | 7.90 (7.42 to 8.38) | 6.50 (6.20 to 6.80) | 5.95 (4.98 to 6.92) | 8.21 (7.32 to 9.10) | 7.59 (6.87 to 8.31) | 5.75 (5.26 to 6.25) | 8.04 (7.59 to 8.48) | 7.07 (6.75 to 7.39) |
| **Tokelau** | 10.75  (-37.61 to 59.11) | 13.26  (-61.20 to 87.72) | 12.77  (-57.94 to 83.48) | 10.66  (-89.53 to 110.85) | 12.18  (-137.77 to 162.12) | 11.63  (-125.39 to 148.65) | 10.76  (-39.85 to 61.37) | 12.68  (-59.01 to 84.36) | 12.15  (-56.34 to 80.64) |
| **Tonga** | 17.73 (1.13 to 34.33) | 19.91 (1.36 to 38.45) | 20.03 (1.30 to 38.76) | 15.11  (-12.05 to 42.27) | 16.21  (-13.17 to 45.59) | 16.28  (-13.32 to 45.88) | 16.71 (1.76 to 31.67) | 18.20 (2.22 to 34.19) | 18.29 (2.15 to 34.43) |
| **Trinidad and Tobago** | 14.01 (11.32 to 16.69) | 16.99 (13.79 to 20.20) | 12.30 (10.48 to 14.11) | 13.24 (7.39 to 19.08) | 14.45 (7.54 to 21.35) | 12.38 (7.47 to 17.29) | 13.66 (11.01 to 16.30) | 15.81 (12.78 to 18.85) | 12.52 (10.59 to 14.45) |
| **Tunisia** | 11.95 (10.40 to 13.50) | 13.52 (11.97 to 15.06) | 9.91 (9.21 to 10.61) | 7.84 (5.94 to 9.74) | 8.77 (6.69 to 10.86) | 7.29 (6.16 to 8.41) | 10.20 (9.01 to 11.40) | 11.33 (10.11 to 12.55) | 8.84 (8.24 to 9.43) |
| **Turkey** | 21.33 (20.24 to 22.42) | 22.94 (21.76 to 24.11) | 19.01 (18.31 to 19.71) | 11.61 (10.46 to 12.76) | 13.25 (11.71 to 14.79) | 11.88 (10.90 to 12.86) | 17.02 (16.22 to 17.82) | 18.20 (17.30 to 19.11) | 15.70 (15.14 to 16.26) |
| **Turkmenistan** | 13.47 (11.30 to 15.65) | 9.14 (7.89 to 10.39) | 7.77 (6.85 to 8.69) | 11.00 (7.61 to 14.39) | 8.30 (5.85 to 10.75) | 7.58 (5.71 to 9.44) | 12.10 (10.30 to 13.89) | 8.69 (7.57 to 9.80) | 7.68 (6.84 to 8.52) |
| **Tuvalu** | 7.68 (-2.66 to 18.02) | 10.57 (-4.77 to 25.91) | 10.77 (-4.91 to 26.45) | 8.50 (-12.67 to 29.66) | 10.79  (-24.34 to 45.93) | 10.88  (-24.92 to 46.67) | 8.11 (-2.43 to 18.66) | 10.60 (-4.28 to 25.48) | 10.73 (-4.38 to 25.83) |
| **Uganda** | 6.18 (5.99 to 6.37) | 13.43 (12.84 to 14.02) | 9.14 (8.88 to 9.39) | 6.82 (6.47 to 7.18) | 14.29 (13.10 to 15.48) | 11.57 (10.91 to 12.23) | 6.57 (6.39 to 6.75) | 13.92 (13.34 to 14.50) | 10.40 (10.12 to 10.69) |
| **Ukraine** | 19.57 (18.82 to 20.32) | 15.08 (14.48 to 15.67) | 12.67 (12.21 to 13.13) | 13.84 (12.34 to 15.33) | 10.95 (9.77 to 12.14) | 9.79 (8.88 to 10.70) | 16.26 (15.62 to 16.91) | 12.86 (12.33 to 13.38) | 11.18 (10.77 to 11.59) |
| **United Arab Emirates** | 13.67 (9.80 to 17.54) | 17.59 (14.31 to 20.88) | 14.01 (11.48 to 16.54) | 17.58 (5.79 to 29.37) | 22.31 (15.46 to 29.17) | 21.13 (15.51 to 26.76) | 14.93 (11.13 to 18.73) | 19.13 (16.62 to 21.63) | 16.37 (14.29 to 18.45) |
| **United Kingdom** | 27.34 (26.14 to 28.55) | 30.97 (29.33 to 32.61) | 26.52 (25.36 to 27.67) | 23.46 (21.04 to 25.89) | 26.71 (24.17 to 29.25) | 24.05 (22.13 to 25.97) | 25.19 (24.05 to 26.33) | 28.67 (27.29 to 30.05) | 25.26 (24.26 to 26.27) |
| **United Republic of Tanzania** | 7.34 (7.08 to 7.60) | 9.53 (9.21 to 9.85) | 6.86 (6.70 to 7.01) | 9.02 (8.48 to 9.57) | 11.85 (11.19 to 12.50) | 10.18 (9.74 to 10.62) | 8.18 (7.92 to 8.45) | 10.67 (10.35 to 11.00) | 8.45 (8.26 to 8.63) |
| **United States of America** | 25.43 (24.92 to 25.95) | 22.36 (21.98 to 22.74) | 19.46 (19.19 to 19.72) | 21.51 (20.59 to 22.44) | 19.88 (19.28 to 20.47) | 18.22 (17.77 to 18.67) | 23.33 (22.87 to 23.78) | 21.02 (20.70 to 21.35) | 18.84 (18.60 to 19.07) |
| **United States Virgin Islands** | 19.03 (3.16 to 34.90) | 20.18 (3.17 to 37.19) | 16.81 (5.42 to 28.21) | 16.46  (-11.44 to 44.36) | 17.13  (-24.31 to 58.56) | 13.63  (-15.06 to 42.33) | 17.51 (4.05 to 30.97) | 18.70 (2.69 to 34.71) | 15.52 (4.05 to 26.98) |
| **Uruguay** | 27.59 (22.64 to 32.54) | 31.03 (25.17 to 36.90) | 27.29 (22.88 to 31.69) | 21.71 (13.38 to 30.03) | 24.83 (15.23 to 34.43) | 22.70 (15.03 to 30.37) | 24.81 (20.49 to 29.13) | 27.82 (22.81 to 32.83) | 25.05 (21.17 to 28.93) |
| **Uzbekistan** | 11.72 (10.78 to 12.66) | 7.74 (7.23 to 8.26) | 7.11 (6.69 to 7.53) | 9.49 (8.00 to 10.98) | 7.47 (6.34 to 8.61) | 7.12 (6.17 to 8.08) | 10.45 (9.67 to 11.23) | 7.58 (7.08 to 8.09) | 7.11 (6.70 to 7.53) |
| **Vanuatu** | 7.34 (4.57 to 10.11) | 8.87 (6.18 to 11.55) | 7.67 (5.74 to 9.60) | 7.20 (1.11 to 13.29) | 8.79 (2.40 to 15.19) | 8.20 (3.05 to 13.36) | 7.45 (4.76 to 10.14) | 8.97 (6.29 to 11.66) | 8.06 (6.00 to 10.12) |
| **Venezuela (Bolivarian Republic of)** | 14.78 (13.60 to 15.95) | 15.24 (14.47 to 16.01) | 10.82 (10.44 to 11.20) | 15.62 (12.93 to 18.31) | 15.42 (13.42 to 17.42) | 12.96 (11.71 to 14.21) | 15.21 (14.04 to 16.38) | 15.39 (14.59 to 16.19) | 12.00 (11.56 to 12.44) |
| **Viet Nam** | 12.41 (11.94 to 12.88) | 14.05 (13.63 to 14.48) | 13.50 (13.13 to 13.88) | 8.22 (7.62 to 8.83) | 10.19 (9.42 to 10.97) | 9.90 (9.22 to 10.59) | 10.30 (9.94 to 10.67) | 12.15 (11.78 to 12.52) | 11.75 (11.42 to 12.08) |
| **Yemen** | 7.19 (6.78 to 7.59) | 8.66 (8.28 to 9.05) | 6.79 (6.58 to 6.99) | 5.40 (4.96 to 5.85) | 7.22 (6.69 to 7.74) | 6.41 (6.04 to 6.77) | 6.27 (5.98 to 6.56) | 8.08 (7.77 to 8.39) | 6.72 (6.54 to 6.91) |
| **Zambia** | 6.57 (6.22 to 6.93) | 11.28 (10.71 to 11.85) | 6.93 (6.73 to 7.13) | 8.52 (7.72 to 9.33) | 12.72 (11.58 to 13.86) | 9.84 (9.25 to 10.44) | 7.56 (7.18 to 7.94) | 12.01 (11.45 to 12.56) | 8.28 (8.04 to 8.51) |
| **Zimbabwe** | 10.93 (10.26 to 11.60) | 10.78 (10.33 to 11.22) | 6.03 (5.88 to 6.18) | 12.26 (10.94 to 13.59) | 14.61 (13.66 to 15.55) | 10.97 (10.45 to 11.48) | 11.55 (10.91 to 12.19) | 12.83 (12.36 to 13.30) | 8.45 (8.25 to 8.65) |

Table S9. Lifetime risks of developing and dying from different cancer types globally by sex in 2021.

| **Cancer type** | **Male** | | **Female** | | **Both sexes** | |
| --- | --- | --- | --- | --- | --- | --- |
|  | **Lifetime incidence risk** | **Lifetime death risk** | **Lifetime incidence risk** | **Lifetime death risk** | **Lifetime incidence risk** | **Lifetime death risk** |
| **Liver cancer** | 0.91 (0.90 to 0.91) | 0.83 (0.83 to 0.83) | 0.50 (0.50 to 0.50) | 0.50 (0.50 to 0.50) | 0.71 (0.71 to 0.71) | 0.67 (0.67 to 0.67) |
| **Neuroblastoma and other peripheral nervous cell tumors** | 0.01 (0.01 to 0.01) | 0.01 (0.01 to 0.01) | 0.01 (0.01 to 0.01) | 0.00 (0.00 to 0.00) | 0.01 (0.01 to 0.01) | 0.01 (0.01 to 0.01) |
| **Malignant neoplasm of bone and articular cartilage** | 0.12 (0.11 to 0.12) | 0.09 (0.09 to 0.09) | 0.09 (0.09 to 0.09) | 0.07 (0.07 to 0.07) | 0.10 (0.10 to 0.10) | 0.08 (0.08 to 0.08) |
| **Soft tissue and other extraosseous sarcomas** | 0.12 (0.12 to 0.12) | 0.07 (0.07 to 0.07) | 0.12 (0.12 to 0.12) | 0.07 (0.07 to 0.07) | 0.12 (0.12 to 0.12) | 0.07 (0.07 to 0.07) |
| **Eye cancer** | 0.04 (0.04 to 0.04) | 0.01 (0.01 to 0.01) | 0.04 (0.04 to 0.04) | 0.01 (0.01 to 0.01) | 0.04 (0.04 to 0.04) | 0.01 (0.01 to 0.01) |
| **Other**  **malignant neoplasms** | 0.53 (0.53 to 0.53) | 0.29 (0.29 to 0.29) | 0.60 (0.60 to 0.60) | 0.34 (0.34 to 0.34) | 0.56 (0.56 to 0.56) | 0.31 (0.31 to 0.31) |
| **Leukemia** | 0.63 (0.63 to 0.63) | 0.46 (0.46 to 0.46) | 0.54 (0.53 to 0.54) | 0.40 (0.40 to 0.40) | 0.59 (0.59 to 0.59) | 0.43 (0.43 to 0.43) |
| **Non-Hodgkin lymphoma** | 0.88 (0.88 to 0.88) | 0.40 (0.40 to 0.40) | 0.71 (0.71 to 0.71) | 0.36 (0.35 to 0.36) | 0.80 (0.80 to 0.80) | 0.38 (0.38 to 0.38) |
| **Hodgkin lymphoma** | 0.08 (0.08 to 0.08) | 0.04 (0.04 to 0.04) | 0.06 (0.06 to 0.06) | 0.03 (0.03 to 0.03) | 0.07 (0.07 to 0.07) | 0.03 (0.03 to 0.03) |
| **Brain and central nervous system cancer** | 0.42 (0.42 to 0.43) | 0.34 (0.33 to 0.34) | 0.42 (0.42 to 0.43) | 0.31 (0.31 to 0.31) | 0.42 (0.42 to 0.43) | 0.32 (0.32 to 0.32) |
| **Kidney cancer** | 0.64 (0.63 to 0.64) | 0.29 (0.29 to 0.29) | 0.39 (0.39 to 0.39) | 0.18 (0.18 to 0.18) | 0.52 (0.52 to 0.52) | 0.24 (0.24 to 0.24) |
| **Thyroid cancer** | 0.19 (0.19 to 0.19) | 0.05 (0.05 to 0.05) | 0.40 (0.40 to 0.40) | 0.09 (0.09 to 0.09) | 0.29 (0.29 to 0.29) | 0.07 (0.07 to 0.07) |
| **Nasopharynx cancer** | 0.19 (0.19 to 0.19) | 0.13 (0.13 to 0.13) | 0.08 (0.08 to 0.08) | 0.06 (0.06 to 0.06) | 0.14 (0.14 to 0.14) | 0.10 (0.10 to 0.10) |
| **Tracheal, bronchus, and lung cancer** | 4.26 (4.25 to 4.26) | 3.88 (3.88 to 3.89) | 2.52 (2.51 to 2.52) | 2.25 (2.25 to 2.26) | 3.44 (3.43 to 3.44) | 3.12 (3.12 to 3.12) |
| **Stomach cancer** | 2.30 (2.29 to 2.30) | 1.79 (1.78 to 1.79) | 1.29 (1.29 to 1.29) | 1.12 (1.12 to 1.12) | 1.82 (1.82 to 1.82) | 1.47 (1.47 to 1.47) |
| **Bladder cancer** | 1.23 (1.22 to 1.23) | 0.53 (0.53 to 0.53) | 0.42 (0.42 to 0.42) | 0.22 (0.22 to 0.22) | 0.85 (0.85 to 0.85) | 0.39 (0.39 to 0.39) |
| **Ovarian cancer** | / | / | 0.79 (0.79 to 0.79) | 0.56 (0.55 to 0.56) | / | / |
| **Testicular cancer** | 0.17 (0.17 to 0.17) | 0.02 (0.02 to 0.02) | / | / | / | / |
| **Pancreatic cancer** | 0.77 (0.77 to 0.77) | 0.78 (0.78 to 0.78) | 0.81 (0.81 to 0.81) | 0.83 (0.83 to 0.83) | 0.79 (0.79 to 0.79) | 0.80 (0.80 to 0.80) |
| **Lip and oral cavity cancer** | 0.69 (0.69 to 0.69) | 0.36 (0.36 to 0.36) | 0.43 (0.43 to 0.44) | 0.22 (0.22 to 0.22) | 0.57 (0.57 to 0.57) | 0.29 (0.29 to 0.29) |
| **Colon and rectum cancer** | 3.47 (3.46 to 3.47) | 1.70 (1.70 to 1.71) | 2.99 (2.98 to 2.99) | 1.62 (1.62 to 1.62) | 3.24 (3.24 to 3.25) | 1.67 (1.67 to 1.67) |
| **Cervical cancer** | / | / | 1.54 (1.53 to 1.55) | 0.78 (0.78 to 0.79) | / | / |
| **Breast cancer** | 0.10 (0.10 to 0.10) | 0.04 (0.04 to 0.04) | 5.41 (5.37 to 5.44) | 1.94 (1.93 to 1.94) | 2.68 (2.67 to 2.68) | 0.94 (0.94 to 0.94) |
| **Larynx cancer** | 0.45 (0.45 to 0.45) | 0.27 (0.27 to 0.27) | 0.08 (0.08 to 0.08) | 0.05 (0.05 to 0.05) | 0.28 (0.28 to 0.28) | 0.17 (0.17 to 0.17) |
| **Esophageal cancer** | 1.18 (1.18 to 1.18) | 1.13 (1.13 to 1.13) | 0.48 (0.48 to 0.48) | 0.47 (0.47 to 0.47) | 0.85 (0.85 to 0.85) | 0.82 (0.82 to 0.82) |
| **Multiple myeloma** | 0.24 (0.24 to 0.24) | 0.19 (0.19 to 0.19) | 0.22 (0.22 to 0.22) | 0.19 (0.19 to 0.19) | 0.23 (0.23 to 0.23) | 0.19 (0.19 to 0.19) |
| **Mesothelioma** | 0.07 (0.07 to 0.07) | 0.07 (0.07 to 0.07) | 0.03 (0.03 to 0.03) | 0.03 (0.03 to 0.03) | 0.05 (0.05 to 0.05) | 0.05 (0.05 to 0.05) |
| **Malignant skin melanoma** | 0.44 (0.43 to 0.44) | 0.10 (0.10 to 0.10) | 0.40 (0.40 to 0.40) | 0.09 (0.09 to 0.09) | 0.42 (0.42 to 0.42) | 0.09 (0.09 to 0.09) |
| **Gallbladder and biliary tract cancer** | 0.30 (0.30 to 0.30) | 0.23 (0.23 to 0.23) | 0.40 (0.40 to 0.40) | 0.33 (0.33 to 0.33) | 0.35 (0.35 to 0.35) | 0.28 (0.28 to 0.28) |
| **Other pharynx cancer** | 0.34 (0.34 to 0.34) | 0.21 (0.21 to 0.21) | 0.09 (0.09 to 0.09) | 0.05 (0.05 to 0.05) | 0.22 (0.22 to 0.22) | 0.13 (0.13 to 0.13) |
| **Uterine cancer** | / | / | 1.31 (1.31 to 1.31) | 0.31 (0.31 to 0.31) | / | / |
| **Prostate cancer** | 3.86 (3.86 to 3.87) | 1.46 (1.46 to 1.46) | / | / | / | / |

Table S10. Lifetime risks of developing and dying from different cancer types by SDI quintiles for males in 2021.

| Location | Cancer type | Lifetime incidence risk | Lifetime death risk |
| --- | --- | --- | --- |
| **High SDI** | Liver cancer | 0.77 (0.77 to 0.77) | 0.70 (0.70 to 0.70) |
|  | Neuroblastoma and other peripheral nervous cell tumors | 0.02 (0.01 to 0.02) | 0.01 (0.01 to 0.01) |
|  | Malignant neoplasm of bone and articular cartilage | 0.08 (0.07 to 0.08) | 0.05 (0.05 to 0.05) |
|  | Soft tissue and other extraosseous sarcomas | 0.26 (0.25 to 0.26) | 0.12 (0.12 to 0.12) |
|  | Other malignant neoplasms | 1.23 (1.20 to 1.25) | 0.53 (0.53 to 0.53) |
|  | Leukemia | 1.07 (1.01 to 1.13) | 0.67 (0.66 to 0.68) |
|  | Non-Hodgkin lymphoma | 1.70 (1.66 to 1.73) | 0.70 (0.70 to 0.70) |
|  | Hodgkin lymphoma | 0.11 (0.10 to 0.12) | 0.02 (0.02 to 0.03) |
|  | Brain and central nervous system cancer | 0.72 (0.65 to 0.79) | 0.43 (0.42 to 0.45) |
|  | Kidney cancer | 0.97 (0.95 to 0.98) | 0.41 (0.41 to 0.41) |
|  | Eye cancer | 0.07 (0.07 to 0.07) | 0.01 (0.01 to 0.01) |
|  | Thyroid cancer | 0.67 (0.65 to 0.69) | 0.11 (0.11 to 0.11) |
|  | Nasopharynx cancer | 0.04 (0.04 to 0.04) | 0.03 (0.02 to 0.03) |
|  | Lip and oral cavity cancer | 0.59 (0.59 to 0.60) | 0.20 (0.20 to 0.20) |
|  | Colon and rectum cancer | 6.12 (6.05 to 6.19) | 2.90 (2.89 to 2.92) |
|  | Cervical cancer | 1.12 (1.06 to 1.18) | 0.41 (0.41 to 0.42) |
|  | Breast cancer | 10.53 (10.22 to 10.84) | 2.90 (2.86 to 2.93) |
|  | Tracheal, bronchus, and lung cancer | 4.89 (4.84 to 4.93) | 4.00 (3.97 to 4.02) |
|  | Stomach cancer | 1.50 (1.49 to 1.50) | 1.07 (1.07 to 1.08) |
|  | Bladder cancer | 1.00 (1.00 to 1.01) | 0.45 (0.45 to 0.45) |
|  | Ovarian cancer | 1.26 (1.24 to 1.28) | 0.94 (0.93 to 0.94) |
|  | Malignant skin melanoma | 1.38 (1.34 to 1.42) | 0.20 (0.20 to 0.20) |
|  | Pancreatic cancer | 1.87 (1.87 to 1.88) | 1.81 (1.81 to 1.82) |
|  | Uterine cancer | 2.84 (2.80 to 2.89) | 0.54 (0.53 to 0.54) |
|  | Larynx cancer | 0.11 (0.11 to 0.11) | 0.04 (0.04 to 0.04) |
|  | Esophageal cancer | 0.39 (0.39 to 0.39) | 0.34 (0.34 to 0.34) |
|  | Multiple myeloma | 0.51 (0.51 to 0.51) | 0.42 (0.42 to 0.42) |
|  | Mesothelioma | 0.05 (0.05 to 0.05) | 0.05 (0.05 to 0.05) |
|  | Gallbladder and biliary tract cancer | 0.74 (0.73 to 0.74) | 0.52 (0.52 to 0.52) |
|  | Other pharynx cancer | 0.16 (0.16 to 0.16) | 0.05 (0.05 to 0.05) |
| **High-middle SDI** | Liver cancer | 0.48 (0.48 to 0.48) | 0.48 (0.48 to 0.49) |
|  | Neuroblastoma and other peripheral nervous cell tumors | 0.01 (0.01 to 0.01) | 0.01 (0.01 to 0.01) |
|  | Malignant neoplasm of bone and articular cartilage | 0.11 (0.10 to 0.11) | 0.08 (0.08 to 0.08) |
|  | Soft tissue and other extraosseous sarcomas | 0.11 (0.11 to 0.11) | 0.06 (0.06 to 0.06) |
|  | Other malignant neoplasms | 0.75 (0.73 to 0.76) | 0.33 (0.33 to 0.33) |
|  | Leukemia | 0.68 (0.65 to 0.72) | 0.43 (0.42 to 0.44) |
|  | Non-Hodgkin lymphoma | 0.74 (0.73 to 0.75) | 0.32 (0.32 to 0.32) |
|  | Hodgkin lymphoma | 0.08 (0.07 to 0.08) | 0.03 (0.03 to 0.03) |
|  | Brain and central nervous system cancer | 0.62 (0.59 to 0.66) | 0.44 (0.43 to 0.45) |
|  | Kidney cancer | 0.57 (0.56 to 0.57) | 0.24 (0.24 to 0.24) |
|  | Eye cancer | 0.05 (0.05 to 0.05) | 0.01 (0.01 to 0.01) |
|  | Thyroid cancer | 0.43 (0.42 to 0.44) | 0.07 (0.07 to 0.07) |
|  | Nasopharynx cancer | 0.12 (0.12 to 0.12) | 0.06 (0.06 to 0.06) |
|  | Lip and oral cavity cancer | 0.31 (0.31 to 0.31) | 0.13 (0.13 to 0.13) |
|  | Colon and rectum cancer | 3.94 (3.91 to 3.98) | 2.05 (2.05 to 2.06) |
|  | Cervical cancer | 1.38 (1.33 to 1.44) | 0.60 (0.59 to 0.60) |
|  | Breast cancer | 6.09 (5.93 to 6.26) | 2.01 (1.99 to 2.03) |
|  | Tracheal, bronchus, and lung cancer | 3.22 (3.20 to 3.24) | 2.92 (2.91 to 2.94) |
|  | Stomach cancer | 1.71 (1.70 to 1.72) | 1.47 (1.47 to 1.48) |
|  | Bladder cancer | 0.47 (0.47 to 0.47) | 0.23 (0.23 to 0.23) |
|  | Ovarian cancer | 0.85 (0.83 to 0.86) | 0.60 (0.59 to 0.60) |
|  | Malignant skin melanoma | 0.45 (0.45 to 0.46) | 0.11 (0.11 to 0.11) |
|  | Pancreatic cancer | 0.99 (0.98 to 0.99) | 1.04 (1.04 to 1.04) |
|  | Uterine cancer | 1.84 (1.82 to 1.87) | 0.37 (0.37 to 0.38) |
|  | Larynx cancer | 0.08 (0.08 to 0.08) | 0.04 (0.04 to 0.04) |
|  | Esophageal cancer | 0.57 (0.57 to 0.57) | 0.54 (0.54 to 0.55) |
|  | Multiple myeloma | 0.23 (0.23 to 0.23) | 0.18 (0.18 to 0.18) |
|  | Mesothelioma | 0.03 (0.03 to 0.03) | 0.03 (0.03 to 0.03) |
|  | Gallbladder and biliary tract cancer | 0.41 (0.41 to 0.41) | 0.33 (0.33 to 0.33) |
|  | Other pharynx cancer | 0.06 (0.06 to 0.06) | 0.03 (0.03 to 0.03) |
| **Middle SDI** | Liver cancer | 0.52 (0.52 to 0.52) | 0.54 (0.54 to 0.54) |
|  | Neuroblastoma and other peripheral nervous cell tumors | 0.01 (0.01 to 0.01) | 0.00 (0.00 to 0.00) |
|  | Malignant neoplasm of bone and articular cartilage | 0.10 (0.10 to 0.10) | 0.09 (0.09 to 0.09) |
|  | Soft tissue and other extraosseous sarcomas | 0.07 (0.07 to 0.07) | 0.05 (0.05 to 0.05) |
|  | Other malignant neoplasms | 0.40 (0.40 to 0.40) | 0.26 (0.26 to 0.26) |
|  | Leukemia | 0.40 (0.39 to 0.41) | 0.33 (0.33 to 0.33) |
|  | Non-Hodgkin lymphoma | 0.46 (0.46 to 0.46) | 0.26 (0.26 to 0.26) |
|  | Hodgkin lymphoma | 0.04 (0.04 to 0.04) | 0.02 (0.02 to 0.02) |
|  | Brain and central nervous system cancer | 0.40 (0.39 to 0.41) | 0.31 (0.31 to 0.31) |
|  | Kidney cancer | 0.19 (0.19 to 0.19) | 0.10 (0.10 to 0.10) |
|  | Eye cancer | 0.02 (0.02 to 0.02) | 0.01 (0.01 to 0.01) |
|  | Thyroid cancer | 0.37 (0.36 to 0.37) | 0.09 (0.09 to 0.09) |
|  | Nasopharynx cancer | 0.09 (0.09 to 0.09) | 0.07 (0.07 to 0.07) |
|  | Lip and oral cavity cancer | 0.34 (0.34 to 0.35) | 0.19 (0.19 to 0.20) |
|  | Colon and rectum cancer | 2.06 (2.05 to 2.07) | 1.24 (1.24 to 1.25) |
|  | Cervical cancer | 1.65 (1.62 to 1.67) | 0.83 (0.83 to 0.83) |
|  | Breast cancer | 4.07 (4.01 to 4.13) | 1.59 (1.58 to 1.60) |
|  | Tracheal, bronchus, and lung cancer | 2.16 (2.15 to 2.17) | 2.09 (2.09 to 2.10) |
|  | Stomach cancer | 1.44 (1.43 to 1.44) | 1.31 (1.31 to 1.31) |
|  | Bladder cancer | 0.22 (0.22 to 0.22) | 0.14 (0.14 to 0.14) |
|  | Ovarian cancer | 0.63 (0.63 to 0.64) | 0.41 (0.41 to 0.41) |
|  | Malignant skin melanoma | 0.10 (0.10 to 0.10) | 0.05 (0.05 to 0.05) |
|  | Pancreatic cancer | 0.47 (0.47 to 0.47) | 0.51 (0.51 to 0.51) |
|  | Uterine cancer | 0.73 (0.72 to 0.73) | 0.23 (0.22 to 0.23) |
|  | Larynx cancer | 0.08 (0.08 to 0.08) | 0.06 (0.06 to 0.06) |
|  | Esophageal cancer | 0.60 (0.60 to 0.60) | 0.59 (0.59 to 0.59) |
|  | Multiple myeloma | 0.12 (0.12 to 0.12) | 0.11 (0.11 to 0.11) |
|  | Mesothelioma | 0.02 (0.02 to 0.02) | 0.02 (0.02 to 0.02) |
|  | Gallbladder and biliary tract cancer | 0.29 (0.29 to 0.29) | 0.28 (0.28 to 0.28) |
|  | Other pharynx cancer | 0.06 (0.06 to 0.06) | 0.04 (0.04 to 0.04) |
| **Low-middle SDI** | Liver cancer | 0.31 (0.31 to 0.31) | 0.34 (0.34 to 0.34) |
|  | Neuroblastoma and other peripheral nervous cell tumors | 0.01 (0.01 to 0.01) | 0.00 (0.00 to 0.00) |
|  | Malignant neoplasm of bone and articular cartilage | 0.07 (0.07 to 0.07) | 0.06 (0.06 to 0.07) |
|  | Soft tissue and other extraosseous sarcomas | 0.07 (0.07 to 0.07) | 0.05 (0.05 to 0.05) |
|  | Other malignant neoplasms | 0.30 (0.30 to 0.30) | 0.29 (0.28 to 0.29) |
|  | Leukemia | 0.26 (0.26 to 0.26) | 0.25 (0.25 to 0.25) |
|  | Non-Hodgkin lymphoma | 0.28 (0.28 to 0.28) | 0.23 (0.23 to 0.23) |
|  | Hodgkin lymphoma | 0.04 (0.04 to 0.04) | 0.03 (0.03 to 0.03) |
|  | Brain and central nervous system cancer | 0.18 (0.18 to 0.18) | 0.17 (0.17 to 0.17) |
|  | Kidney cancer | 0.09 (0.09 to 0.09) | 0.06 (0.06 to 0.06) |
|  | Eye cancer | 0.03 (0.03 to 0.03) | 0.01 (0.01 to 0.01) |
|  | Thyroid cancer | 0.26 (0.26 to 0.26) | 0.08 (0.08 to 0.08) |
|  | Nasopharynx cancer | 0.06 (0.06 to 0.06) | 0.06 (0.06 to 0.06) |
|  | Lip and oral cavity cancer | 0.59 (0.58 to 0.59) | 0.40 (0.40 to 0.40) |
|  | Colon and rectum cancer | 0.84 (0.84 to 0.85) | 0.69 (0.69 to 0.69) |
|  | Cervical cancer | 1.66 (1.64 to 1.68) | 0.99 (0.99 to 1.00) |
|  | Breast cancer | 2.75 (2.72 to 2.78) | 1.50 (1.49 to 1.51) |
|  | Tracheal, bronchus, and lung cancer | 0.60 (0.59 to 0.60) | 0.63 (0.63 to 0.64) |
|  | Stomach cancer | 0.62 (0.62 to 0.63) | 0.64 (0.64 to 0.64) |
|  | Bladder cancer | 0.14 (0.14 to 0.14) | 0.10 (0.10 to 0.10) |
|  | Ovarian cancer | 0.59 (0.59 to 0.60) | 0.40 (0.40 to 0.40) |
|  | Malignant skin melanoma | 0.04 (0.04 to 0.04) | 0.03 (0.03 to 0.03) |
|  | Pancreatic cancer | 0.22 (0.22 to 0.22) | 0.24 (0.24 to 0.24) |
|  | Uterine cancer | 0.44 (0.44 to 0.44) | 0.19 (0.19 to 0.19) |
|  | Larynx cancer | 0.08 (0.08 to 0.08) | 0.07 (0.07 to 0.07) |
|  | Esophageal cancer | 0.31 (0.31 to 0.31) | 0.33 (0.33 to 0.33) |
|  | Multiple myeloma | 0.09 (0.09 to 0.09) | 0.09 (0.09 to 0.09) |
|  | Mesothelioma | 0.01 (0.01 to 0.01) | 0.01 (0.01 to 0.01) |
|  | Gallbladder and biliary tract cancer | 0.26 (0.26 to 0.26) | 0.28 (0.27 to 0.28) |
|  | Other pharynx cancer | 0.11 (0.11 to 0.11) | 0.09 (0.09 to 0.09) |
| **Low SDI** | Liver cancer | 0.40 (0.40 to 0.40) | 0.43 (0.43 to 0.43) |
|  | Neuroblastoma and other peripheral nervous cell tumors | 0.00 (0.00 to 0.00) | 0.00 (0.00 to 0.00) |
|  | Malignant neoplasm of bone and articular cartilage | 0.05 (0.05 to 0.05) | 0.05 (0.05 to 0.05) |
|  | Soft tissue and other extraosseous sarcomas | 0.09 (0.09 to 0.09) | 0.07 (0.07 to 0.07) |
|  | Other malignant neoplasms | 0.28 (0.28 to 0.28) | 0.28 (0.28 to 0.28) |
|  | Leukemia | 0.21 (0.21 to 0.21) | 0.21 (0.21 to 0.21) |
|  | Non-Hodgkin lymphoma | 0.28 (0.28 to 0.28) | 0.25 (0.25 to 0.25) |
|  | Hodgkin lymphoma | 0.05 (0.05 to 0.05) | 0.04 (0.04 to 0.04) |
|  | Brain and central nervous system cancer | 0.11 (0.11 to 0.11) | 0.10 (0.10 to 0.10) |
|  | Kidney cancer | 0.07 (0.07 to 0.07) | 0.05 (0.05 to 0.05) |
|  | Eye cancer | 0.07 (0.07 to 0.07) | 0.04 (0.04 to 0.04) |
|  | Thyroid cancer | 0.21 (0.21 to 0.21) | 0.07 (0.07 to 0.07) |
|  | Nasopharynx cancer | 0.06 (0.06 to 0.06) | 0.06 (0.06 to 0.06) |
|  | Lip and oral cavity cancer | 0.35 (0.35 to 0.35) | 0.25 (0.25 to 0.25) |
|  | Colon and rectum cancer | 0.64 (0.63 to 0.64) | 0.58 (0.57 to 0.58) |
|  | Cervical cancer | 2.23 (2.21 to 2.26) | 1.51 (1.50 to 1.52) |
|  | Breast cancer | 2.16 (2.15 to 2.18) | 1.46 (1.45 to 1.47) |
|  | Tracheal, bronchus, and lung cancer | 0.30 (0.30 to 0.30) | 0.32 (0.32 to 0.32) |
|  | Stomach cancer | 0.65 (0.65 to 0.65) | 0.68 (0.68 to 0.68) |
|  | Bladder cancer | 0.18 (0.18 to 0.18) | 0.14 (0.14 to 0.14) |
|  | Ovarian cancer | 0.50 (0.49 to 0.50) | 0.36 (0.36 to 0.36) |
|  | Malignant skin melanoma | 0.06 (0.06 to 0.06) | 0.04 (0.04 to 0.04) |
|  | Pancreatic cancer | 0.15 (0.15 to 0.15) | 0.16 (0.16 to 0.16) |
|  | Uterine cancer | 0.35 (0.35 to 0.35) | 0.18 (0.18 to 0.18) |
|  | Larynx cancer | 0.06 (0.06 to 0.06) | 0.06 (0.06 to 0.06) |
|  | Esophageal cancer | 0.46 (0.46 to 0.46) | 0.50 (0.50 to 0.50) |
|  | Multiple myeloma | 0.07 (0.07 to 0.07) | 0.07 (0.07 to 0.07) |
|  | Mesothelioma | 0.01 (0.01 to 0.01) | 0.01 (0.01 to 0.01) |
|  | Gallbladder and biliary tract cancer | 0.16 (0.16 to 0.16) | 0.17 (0.17 to 0.17) |
|  | Other pharynx cancer | 0.08 (0.08 to 0.08) | 0.06 (0.06 to 0.06) |

Table S11. Lifetime risks of developing and dying from different cancer types by SDI quintiles for females in 2021.

| **Location** | **Cancer type** | **Lifetime incidence risk** | **Lifetime death risk** |
| --- | --- | --- | --- |
| **High SDI** | Liver cancer | 1.54 (1.54 to 1.55) | 1.28 (1.28 to 1.28) |
|  | Neuroblastoma and other peripheral nervous cell tumors | 0.02 (0.02 to 0.02) | 0.01 (0.01 to 0.01) |
|  | Malignant neoplasm of bone and articular cartilage | 0.10 (0.09 to 0.10) | 0.06 (0.06 to 0.06) |
|  | Soft tissue and other extraosseous sarcomas | 0.29 (0.28 to 0.29) | 0.13 (0.13 to 0.13) |
|  | Other malignant neoplasms | 0.94 (0.93 to 0.95) | 0.41 (0.41 to 0.41) |
|  | Leukemia | 1.42 (1.37 to 1.47) | 0.89 (0.89 to 0.90) |
|  | Non-Hodgkin lymphoma | 2.25 (2.22 to 2.29) | 0.84 (0.84 to 0.84) |
|  | Hodgkin lymphoma | 0.15 (0.15 to 0.16) | 0.03 (0.03 to 0.03) |
|  | Brain and central nervous system cancer | 0.82 (0.78 to 0.87) | 0.54 (0.53 to 0.55) |
|  | Kidney cancer | 1.71 (1.70 to 1.72) | 0.71 (0.71 to 0.71) |
|  | Eye cancer | 0.07 (0.07 to 0.08) | 0.01 (0.01 to 0.01) |
|  | Thyroid cancer | 0.38 (0.37 to 0.38) | 0.07 (0.07 to 0.07) |
|  | Nasopharynx cancer | 0.12 (0.12 to 0.12) | 0.07 (0.07 to 0.07) |
|  | Lip and oral cavity cancer | 1.03 (1.03 to 1.03) | 0.33 (0.33 to 0.33) |
|  | Colon and rectum cancer | 7.29 (7.24 to 7.34) | 3.11 (3.10 to 3.12) |
|  | Breast cancer | 0.11 (0.11 to 0.11) | 0.03 (0.03 to 0.03) |
|  | Tracheal, bronchus, and lung cancer | 7.16 (7.12 to 7.19) | 6.07 (6.05 to 6.10) |
|  | Stomach cancer | 2.58 (2.57 to 2.59) | 1.68 (1.68 to 1.69) |
|  | Bladder cancer | 2.82 (2.81 to 2.83) | 1.10 (1.10 to 1.10) |
|  | Testicular cancer | 0.45 (0.42 to 0.47) | 0.02 (0.02 to 0.02) |
|  | Malignant skin melanoma | 1.76 (1.75 to 1.77) | 0.29 (0.29 to 0.29) |
|  | Pancreatic cancer | 1.81 (1.80 to 1.81) | 1.74 (1.73 to 1.74) |
|  | Prostate cancer | 10.44 (10.38 to 10.50) | 3.03 (3.03 to 3.04) |
|  | Larynx cancer | 0.58 (0.58 to 0.58) | 0.21 (0.21 to 0.21) |
|  | Esophageal cancer | 1.26 (1.26 to 1.26) | 1.08 (1.08 to 1.09) |
|  | Multiple myeloma | 0.65 (0.64 to 0.65) | 0.49 (0.49 to 0.49) |
|  | Mesothelioma | 0.22 (0.22 to 0.22) | 0.20 (0.20 to 0.20) |
|  | Gallbladder and biliary tract cancer | 0.67 (0.67 to 0.67) | 0.45 (0.45 to 0.45) |
|  | Other pharynx cancer | 0.64 (0.64 to 0.64) | 0.23 (0.23 to 0.23) |
| **High-middle SDI** | Liver cancer | 1.02 (1.02 to 1.03) | 0.94 (0.94 to 0.94) |
|  | Neuroblastoma and other peripheral nervous cell tumors | 0.02 (0.02 to 0.02) | 0.01 (0.01 to 0.01) |
|  | Malignant neoplasm of bone and articular cartilage | 0.15 (0.15 to 0.16) | 0.11 (0.11 to 0.11) |
|  | Soft tissue and other extraosseous sarcomas | 0.13 (0.13 to 0.13) | 0.06 (0.06 to 0.06) |
|  | Other malignant neoplasms | 0.75 (0.75 to 0.76) | 0.32 (0.31 to 0.32) |
|  | Leukemia | 0.84 (0.81 to 0.87) | 0.52 (0.52 to 0.53) |
|  | Non-Hodgkin lymphoma | 0.99 (0.98 to 1.00) | 0.39 (0.39 to 0.39) |
|  | Hodgkin lymphoma | 0.09 (0.09 to 0.10) | 0.03 (0.03 to 0.03) |
|  | Brain and central nervous system cancer | 0.60 (0.59 to 0.62) | 0.48 (0.47 to 0.48) |
|  | Kidney cancer | 0.95 (0.95 to 0.96) | 0.42 (0.42 to 0.42) |
|  | Eye cancer | 0.04 (0.04 to 0.04) | 0.01 (0.01 to 0.01) |
|  | Thyroid cancer | 0.22 (0.22 to 0.22) | 0.05 (0.05 to 0.05) |
|  | Nasopharynx cancer | 0.35 (0.34 to 0.35) | 0.18 (0.18 to 0.18) |
|  | Lip and oral cavity cancer | 0.65 (0.65 to 0.65) | 0.29 (0.29 to 0.29) |
|  | Colon and rectum cancer | 5.12 (5.10 to 5.15) | 2.50 (2.50 to 2.51) |
|  | Breast cancer | 0.13 (0.13 to 0.13) | 0.03 (0.03 to 0.03) |
|  | Tracheal, bronchus, and lung cancer | 6.77 (6.74 to 6.80) | 6.20 (6.18 to 6.23) |
|  | Stomach cancer | 3.56 (3.55 to 3.57) | 2.75 (2.74 to 2.76) |
|  | Bladder cancer | 1.86 (1.85 to 1.86) | 0.77 (0.77 to 0.78) |
|  | Testicular cancer | 0.29 (0.27 to 0.30) | 0.03 (0.03 to 0.03) |
|  | Malignant skin melanoma | 0.38 (0.38 to 0.38) | 0.11 (0.11 to 0.11) |
|  | Pancreatic cancer | 1.10 (1.09 to 1.10) | 1.12 (1.12 to 1.12) |
|  | Prostate cancer | 3.75 (3.74 to 3.75) | 1.47 (1.47 to 1.48) |
|  | Larynx cancer | 0.56 (0.56 to 0.56) | 0.30 (0.30 to 0.30) |
|  | Esophageal cancer | 1.83 (1.83 to 1.84) | 1.74 (1.73 to 1.74) |
|  | Multiple myeloma | 0.26 (0.26 to 0.26) | 0.19 (0.19 to 0.19) |
|  | Mesothelioma | 0.06 (0.06 to 0.06) | 0.06 (0.06 to 0.06) |
|  | Gallbladder and biliary tract cancer | 0.36 (0.35 to 0.36) | 0.26 (0.26 to 0.26) |
|  | Other pharynx cancer | 0.30 (0.29 to 0.30) | 0.15 (0.15 to 0.15) |
| **Middle SDI** | Liver cancer | 0.96 (0.96 to 0.96) | 0.91 (0.91 to 0.91) |
|  | Neuroblastoma and other peripheral nervous cell tumors | 0.01 (0.01 to 0.01) | 0.01 (0.01 to 0.01) |
|  | Malignant neoplasm of bone and articular cartilage | 0.13 (0.13 to 0.13) | 0.11 (0.11 to 0.11) |
|  | Soft tissue and other extraosseous sarcomas | 0.08 (0.08 to 0.08) | 0.05 (0.05 to 0.05) |
|  | Other malignant neoplasms | 0.46 (0.46 to 0.46) | 0.28 (0.28 to 0.28) |
|  | Leukemia | 0.47 (0.47 to 0.48) | 0.38 (0.38 to 0.38) |
|  | Non-Hodgkin lymphoma | 0.62 (0.62 to 0.62) | 0.32 (0.32 to 0.32) |
|  | Hodgkin lymphoma | 0.05 (0.05 to 0.05) | 0.03 (0.03 to 0.03) |
|  | Brain and central nervous system cancer | 0.39 (0.39 to 0.40) | 0.33 (0.32 to 0.33) |
|  | Kidney cancer | 0.34 (0.33 to 0.34) | 0.18 (0.18 to 0.18) |
|  | Eye cancer | 0.02 (0.02 to 0.02) | 0.01 (0.01 to 0.01) |
|  | Thyroid cancer | 0.17 (0.17 to 0.17) | 0.06 (0.06 to 0.06) |
|  | Nasopharynx cancer | 0.22 (0.22 to 0.22) | 0.16 (0.16 to 0.16) |
|  | Lip and oral cavity cancer | 0.61 (0.60 to 0.61) | 0.35 (0.35 to 0.35) |
|  | Colon and rectum cancer | 2.65 (2.65 to 2.66) | 1.45 (1.45 to 1.45) |
|  | Breast cancer | 0.11 (0.11 to 0.11) | 0.04 (0.04 to 0.04) |
|  | Tracheal, bronchus, and lung cancer | 3.90 (3.89 to 3.91) | 3.74 (3.74 to 3.75) |
|  | Stomach cancer | 2.70 (2.69 to 2.70) | 2.16 (2.16 to 2.16) |
|  | Bladder cancer | 0.69 (0.69 to 0.69) | 0.35 (0.35 to 0.35) |
|  | Testicular cancer | 0.12 (0.12 to 0.12) | 0.03 (0.03 to 0.03) |
|  | Malignant skin melanoma | 0.09 (0.09 to 0.09) | 0.04 (0.04 to 0.04) |
|  | Pancreatic cancer | 0.50 (0.50 to 0.50) | 0.52 (0.52 to 0.52) |
|  | Prostate cancer | 2.16 (2.16 to 2.17) | 1.08 (1.08 to 1.08) |
|  | Larynx cancer | 0.39 (0.39 to 0.39) | 0.26 (0.26 to 0.26) |
|  | Esophageal cancer | 1.42 (1.41 to 1.42) | 1.41 (1.41 to 1.42) |
|  | Multiple myeloma | 0.13 (0.13 to 0.13) | 0.11 (0.11 to 0.11) |
|  | Mesothelioma | 0.03 (0.03 to 0.03) | 0.03 (0.03 to 0.03) |
|  | Gallbladder and biliary tract cancer | 0.23 (0.23 to 0.23) | 0.20 (0.20 to 0.20) |
|  | Other pharynx cancer | 0.22 (0.22 to 0.22) | 0.17 (0.17 to 0.17) |
| **Low-middle SDI** | Liver cancer | 0.43 (0.43 to 0.44) | 0.45 (0.45 to 0.45) |
|  | Neuroblastoma and other peripheral nervous cell tumors | 0.01 (0.01 to 0.01) | 0.00 (0.00 to 0.00) |
|  | Malignant neoplasm of bone and articular cartilage | 0.09 (0.09 to 0.09) | 0.08 (0.08 to 0.08) |
|  | Soft tissue and other extraosseous sarcomas | 0.07 (0.07 to 0.07) | 0.05 (0.05 to 0.05) |
|  | Other malignant neoplasms | 0.27 (0.27 to 0.27) | 0.25 (0.25 to 0.25) |
|  | Leukemia | 0.29 (0.29 to 0.29) | 0.28 (0.27 to 0.28) |
|  | Non-Hodgkin lymphoma | 0.33 (0.33 to 0.33) | 0.25 (0.25 to 0.25) |
|  | Hodgkin lymphoma | 0.06 (0.06 to 0.06) | 0.04 (0.04 to 0.04) |
|  | Brain and central nervous system cancer | 0.20 (0.19 to 0.20) | 0.18 (0.18 to 0.18) |
|  | Kidney cancer | 0.14 (0.14 to 0.14) | 0.10 (0.10 to 0.10) |
|  | Eye cancer | 0.02 (0.02 to 0.02) | 0.01 (0.01 to 0.01) |
|  | Thyroid cancer | 0.09 (0.08 to 0.09) | 0.04 (0.04 to 0.04) |
|  | Nasopharynx cancer | 0.11 (0.11 to 0.11) | 0.11 (0.11 to 0.11) |
|  | Lip and oral cavity cancer | 0.69 (0.69 to 0.69) | 0.49 (0.49 to 0.50) |
|  | Colon and rectum cancer | 0.75 (0.75 to 0.75) | 0.59 (0.59 to 0.59) |
|  | Breast cancer | 0.05 (0.05 to 0.05) | 0.03 (0.03 to 0.03) |
|  | Tracheal, bronchus, and lung cancer | 1.16 (1.16 to 1.16) | 1.22 (1.21 to 1.22) |
|  | Stomach cancer | 0.86 (0.86 to 0.86) | 0.85 (0.85 to 0.85) |
|  | Bladder cancer | 0.37 (0.37 to 0.37) | 0.22 (0.22 to 0.22) |
|  | Testicular cancer | 0.05 (0.05 to 0.05) | 0.02 (0.02 to 0.02) |
|  | Malignant skin melanoma | 0.04 (0.04 to 0.04) | 0.03 (0.03 to 0.03) |
|  | Pancreatic cancer | 0.22 (0.22 to 0.22) | 0.23 (0.23 to 0.23) |
|  | Prostate cancer | 1.35 (1.34 to 1.35) | 0.85 (0.85 to 0.85) |
|  | Larynx cancer | 0.39 (0.39 to 0.40) | 0.33 (0.33 to 0.33) |
|  | Esophageal cancer | 0.39 (0.39 to 0.40) | 0.41 (0.41 to 0.41) |
|  | Multiple myeloma | 0.09 (0.09 to 0.09) | 0.09 (0.09 to 0.09) |
|  | Mesothelioma | 0.03 (0.03 to 0.03) | 0.03 (0.03 to 0.03) |
|  | Gallbladder and biliary tract cancer | 0.12 (0.12 to 0.12) | 0.13 (0.13 to 0.13) |
|  | Other pharynx cancer | 0.37 (0.37 to 0.37) | 0.32 (0.32 to 0.32) |
| **Low SDI** | Liver cancer | 0.42 (0.42 to 0.42) | 0.43 (0.43 to 0.44) |
|  | Neuroblastoma and other peripheral nervous cell tumors | 0.00 (0.00 to 0.00) | 0.00 (0.00 to 0.00) |
|  | Malignant neoplasm of bone and articular cartilage | 0.08 (0.08 to 0.08) | 0.07 (0.07 to 0.07) |
|  | Soft tissue and other extraosseous sarcomas | 0.09 (0.09 to 0.09) | 0.07 (0.07 to 0.07) |
|  | Other malignant neoplasms | 0.18 (0.18 to 0.18) | 0.17 (0.17 to 0.17) |
|  | Leukemia | 0.21 (0.21 to 0.21) | 0.21 (0.21 to 0.21) |
|  | Non-Hodgkin lymphoma | 0.30 (0.30 to 0.30) | 0.26 (0.26 to 0.26) |
|  | Hodgkin lymphoma | 0.07 (0.07 to 0.07) | 0.06 (0.06 to 0.06) |
|  | Brain and central nervous system cancer | 0.10 (0.10 to 0.10) | 0.09 (0.09 to 0.09) |
|  | Kidney cancer | 0.10 (0.10 to 0.10) | 0.08 (0.08 to 0.08) |
|  | Eye cancer | 0.06 (0.06 to 0.06) | 0.03 (0.03 to 0.03) |
|  | Thyroid cancer | 0.06 (0.06 to 0.06) | 0.03 (0.03 to 0.03) |
|  | Nasopharynx cancer | 0.09 (0.09 to 0.09) | 0.09 (0.09 to 0.09) |
|  | Lip and oral cavity cancer | 0.41 (0.41 to 0.41) | 0.31 (0.31 to 0.31) |
|  | Colon and rectum cancer | 0.59 (0.59 to 0.59) | 0.53 (0.53 to 0.53) |
|  | Breast cancer | 0.08 (0.08 to 0.08) | 0.06 (0.06 to 0.06) |
|  | Tracheal, bronchus, and lung cancer | 0.62 (0.62 to 0.62) | 0.65 (0.65 to 0.65) |
|  | Stomach cancer | 0.71 (0.71 to 0.71) | 0.72 (0.72 to 0.72) |
|  | Bladder cancer | 0.29 (0.29 to 0.29) | 0.20 (0.20 to 0.20) |
|  | Testicular cancer | 0.03 (0.03 to 0.03) | 0.02 (0.02 to 0.02) |
|  | Malignant skin melanoma | 0.05 (0.05 to 0.05) | 0.04 (0.04 to 0.04) |
|  | Pancreatic cancer | 0.13 (0.13 to 0.13) | 0.13 (0.13 to 0.13) |
|  | Prostate cancer | 1.24 (1.24 to 1.24) | 1.04 (1.04 to 1.04) |
|  | Larynx cancer | 0.27 (0.27 to 0.27) | 0.24 (0.24 to 0.24) |
|  | Esophageal cancer | 0.49 (0.49 to 0.49) | 0.51 (0.51 to 0.51) |
|  | Multiple myeloma | 0.06 (0.06 to 0.06) | 0.06 (0.06 to 0.06) |
|  | Mesothelioma | 0.02 (0.02 to 0.02) | 0.02 (0.02 to 0.02) |
|  | Gallbladder and biliary tract cancer | 0.07 (0.07 to 0.07) | 0.07 (0.07 to 0.07) |
|  | Other pharynx cancer | 0.19 (0.19 to 0.19) | 0.17 (0.17 to 0.17) |

Table S12. Types of cancer with the highest lifetime incidence risk across 204 countries and territories by sex in 2021.

| **Location** | **Male** | | **Female** | |
| --- | --- | --- | --- | --- |
|  | **Cancer type** | **Lifetime incidence risk** | **Cancer type** | **Lifetime incidence risk** |
| **Afghanistan** | Stomach cancer | 1.5 (1.48 to 1.52) | Stomach cancer | 2.65 (2.58 to 2.72) |
| **Albania** | Tracheal, bronchus, and lung cancer | 4.63 (4.16 to 5.1) | Breast cancer | 3.61 (0.84 to 6.38) |
| **Algeria** | Tracheal, bronchus, and lung cancer | 1.15 (1.14 to 1.17) | Breast cancer | 3.13 (2.81 to 3.46) |
| **American Samoa** | Prostate cancer | 7.13 (4.08 to 10.19) | Breast cancer | 7.08 (-10.34 to 24.5) |
| **Andorra** | Prostate cancer | 13.56 (3.47 to 23.65) | Breast cancer | 11.7 (-48.91 to 72.32) |
| **Angola** | Prostate cancer | 1.41 (1.4 to 1.42) | Cervical cancer | 2.86 (2.71 to 3.01) |
| **Antigua and Barbuda** | Prostate cancer | 18.49 (9.52 to 27.45) | Breast cancer | 10.3 (-13.29 to 33.9) |
| **Argentina** | Prostate cancer | 4.72 (4.65 to 4.78) | Breast cancer | 6.86 (6.06 to 7.66) |
| **Armenia** | Tracheal, bronchus, and lung cancer | 5.76 (5.29 to 6.22) | Breast cancer | 6.16 (2.98 to 9.34) |
| **Australia** | Prostate cancer | 16.37 (15.36 to 17.38) | Breast cancer | 11.68 (8.61 to 14.75) |
| **Austria** | Prostate cancer | 11.09 (10.23 to 11.94) | Breast cancer | 9.16 (4.53 to 13.78) |
| **Azerbaijan** | Tracheal, bronchus, and lung cancer | 2.38 (2.31 to 2.46) | Breast cancer | 3.39 (2.59 to 4.18) |
| **Bahamas** | Prostate cancer | 10.83 (9.04 to 12.61) | Breast cancer | 9.76 (2.08 to 17.43) |
| **Bahrain** | Prostate cancer | 6.54 (5.82 to 7.26) | Breast cancer | 11.9 (2.27 to 21.52) |
| **Bangladesh** | Lip and oral cavity cancer | 1.01 (1 to 1.02) | Breast cancer | 1.49 (1.38 to 1.61) |
| **Barbados** | Prostate cancer | 15.67 (11.59 to 19.76) | Breast cancer | 12.03 (-3.54 to 27.6) |
| **Belarus** | Prostate cancer | 5.76 (5.65 to 5.87) | Breast cancer | 5.62 (4.6 to 6.64) |
| **Belgium** | Prostate cancer | 10.15 (9.59 to 10.7) | Breast cancer | 11.85 (7.68 to 16.02) |
| **Belize** | Prostate cancer | 7.5 (5.79 to 9.21) | Cervical cancer | 3.98 (-1.38 to 9.34) |
| **Benin** | Prostate cancer | 2.07 (2.03 to 2.1) | Cervical cancer | 2.33 (2.06 to 2.6) |
| **Bermuda** | Prostate cancer | 23.74 (9.27 to 38.2) | Breast cancer | 12.88 (-40.36 to 66.13) |
| **Bhutan** | Lip and oral cavity cancer | 0.97 (0.78 to 1.16) | Breast cancer | 1.72 (0.44 to 2.99) |
| **Bolivia (Plurinational State of)** | Prostate cancer | 2.55 (2.52 to 2.58) | Cervical cancer | 3.34 (2.94 to 3.75) |
| **Bosnia and Herzegovina** | Tracheal, bronchus, and lung cancer | 6.77 (6.21 to 7.33) | Breast cancer | 5.76 (2.97 to 8.55) |
| **Botswana** | Prostate cancer | 1.95 (1.91 to 2) | Breast cancer | 2.49 (2.29 to 2.68) |
| **Brazil** | Prostate cancer | 4.75 (4.72 to 4.78) | Breast cancer | 4.96 (4.77 to 5.16) |
| **Brunei Darussalam** | Colon and rectum cancer | 4.6 (3.09 to 6.1) | Breast cancer | 5.48 (-3.45 to 14.4) |
| **Bulgaria** | Tracheal, bronchus, and lung cancer | 4.95 (4.78 to 5.11) | Breast cancer | 7.77 (6 to 9.54) |
| **Burkina Faso** | Prostate cancer | 1.65 (1.63 to 1.66) | Breast cancer | 2.72 (2.54 to 2.9) |
| **Burundi** | Prostate cancer | 1.5 (1.48 to 1.52) | Cervical cancer | 3.02 (2.65 to 3.38) |
| **Cabo Verde** | Prostate cancer | 6.98 (5.94 to 8.01) | Breast cancer | 3.43 (-0.14 to 7.01) |
| **Cambodia** | Tracheal, bronchus, and lung cancer | 3.62 (3.53 to 3.7) | Breast cancer | 3.29 (2.91 to 3.68) |
| **Cameroon** | Prostate cancer | 1.93 (1.91 to 1.95) | Cervical cancer | 2.6 (2.47 to 2.74) |
| **Canada** | Prostate cancer | 8.65 (8.33 to 8.96) | Breast cancer | 11.25 (9.25 to 13.24) |
| **Central African Republic** | Tracheal, bronchus, and lung cancer | 0.7 (0.69 to 0.71) | Cervical cancer | 3.06 (2.83 to 3.29) |
| **Chad** | Prostate cancer | 1.61 (1.59 to 1.63) | Cervical cancer | 2.9 (2.66 to 3.14) |
| **Chile** | Prostate cancer | 7.8 (7.54 to 8.05) | Breast cancer | 5.13 (3.96 to 6.3) |
| **China** | Tracheal, bronchus, and lung cancer | 8.07 (8.02 to 8.11) | Tracheal, bronchus, and lung cancer | 4.79 (4.74 to 4.84) |
| **Colombia** | Prostate cancer | 9.36 (9.15 to 9.58) | Breast cancer | 7.38 (6.35 to 8.41) |
| **Comoros** | Prostate cancer | 2.5 (2.32 to 2.69) | Cervical cancer | 3.48 (1.21 to 5.75) |
| **Congo** | Prostate cancer | 1.82 (1.78 to 1.85) | Cervical cancer | 3.6 (3.19 to 4.01) |
| **Cook Islands** | Prostate cancer | 15 (0.61 to 29.39) | Breast cancer | 11.24 (-75.5 to 97.99) |
| **Costa Rica** | Prostate cancer | 13.46 (12.23 to 14.7) | Breast cancer | 9.76 (5.48 to 14.04) |
| **Cote d’Ivoire** | Prostate cancer | 3.39 (3.35 to 3.44) | Breast cancer | 3.22 (3.04 to 3.4) |
| **Croatia** | Prostate cancer | 8.08 (7.72 to 8.44) | Breast cancer | 8.43 (3.49 to 13.36) |
| **Cuba** | Prostate cancer | 12.29 (11.94 to 12.64) | Breast cancer | 7.18 (5.91 to 8.45) |
| **Cyprus** | Prostate cancer | 13.04 (10.59 to 15.48) | Breast cancer | 12.83 (-2.84 to 28.5) |
| **Czechia** | Prostate cancer | 7.55 (7.32 to 7.78) | Breast cancer | 7.64 (4.99 to 10.29) |
| **Democratic People's Republic of Korea** | Tracheal, bronchus, and lung cancer | 3.87 (3.78 to 3.97) | Breast cancer | 2.56 (2.19 to 2.93) |
| **Democratic Republic of the Congo** | Prostate cancer | 1.36 (1.36 to 1.37) | Cervical cancer | 3.21 (3.08 to 3.34) |
| **Denmark** | Prostate cancer | 12.49 (11.59 to 13.38) | Breast cancer | 10.64 (4.55 to 16.74) |
| **Djibouti** | Prostate cancer | 2.21 (2.1 to 2.31) | Breast cancer | 3.04 (2.41 to 3.67) |
| **Dominica** | Prostate cancer | 9.08 (6.21 to 11.96) | Breast cancer | 7.24 (-8.53 to 23.02) |
| **Dominican Republic** | Prostate cancer | 6.58 (6.35 to 6.82) | Breast cancer | 3.81 (3.08 to 4.53) |
| **Ecuador** | Prostate cancer | 4.56 (4.46 to 4.66) | Breast cancer | 3.65 (3.09 to 4.21) |
| **Egypt** | Liver cancer | 1.91 (1.89 to 1.92) | Breast cancer | 5.34 (5.01 to 5.66) |
| **El Salvador** | Prostate cancer | 7.33 (6.94 to 7.72) | Breast cancer | 5.02 (3.62 to 6.43) |
| **Equatorial Guinea** | Prostate cancer | 2.02 (1.92 to 2.13) | Breast cancer | 3.46 (2.81 to 4.12) |
| **Eritrea** | Prostate cancer | 1.46 (1.43 to 1.49) | Cervical cancer | 4.02 (3.42 to 4.62) |
| **Estonia** | Prostate cancer | 14.73 (13.19 to 16.27) | Breast cancer | 7.28 (1.8 to 12.76) |
| **Eswatini** | Liver cancer | 1.15 (1.11 to 1.19) | Cervical cancer | 3.16 (2.8 to 3.52) |
| **Ethiopia** | Colon and rectum cancer | 1.61 (1.6 to 1.62) | Breast cancer | 2.6 (2.52 to 2.68) |
| **Fiji** | Prostate cancer | 1.47 (1.42 to 1.51) | Breast cancer | 5.13 (3.01 to 7.25) |
| **Finland** | Prostate cancer | 15.26 (13.96 to 16.55) | Breast cancer | 11.56 (4.88 to 18.24) |
| **France** | Prostate cancer | 13.83 (13.43 to 14.23) | Breast cancer | 13.68 (11.29 to 16.08) |
| **Gabon** | Prostate cancer | 2.13 (2.07 to 2.2) | Breast cancer | 3.81 (3.08 to 4.55) |
| **Gambia** | Liver cancer | 3.21 (2.87 to 3.54) | Cervical cancer | 2.28 (1.73 to 2.84) |
| **Georgia** | Tracheal, bronchus, and lung cancer | 4.47 (4.25 to 4.69) | Breast cancer | 7.13 (4.3 to 9.95) |
| **Germany** | Prostate cancer | 12.59 (12.35 to 12.82) | Breast cancer | 12.12 (10.4 to 13.84) |
| **Ghana** | Prostate cancer | 2.7 (2.67 to 2.73) | Breast cancer | 2.89 (2.75 to 3.03) |
| **Greece** | Tracheal, bronchus, and lung cancer | 9.7 (9.02 to 10.38) | Breast cancer | 11.54 (7.2 to 15.89) |
| **Greenland** | Tracheal, bronchus, and lung cancer | 8.61 (3.02 to 14.2) | Tracheal, bronchus, and lung cancer | 7.37 (-1.54 to 16.27) |
| **Grenada** | Prostate cancer | 11.7 (8.67 to 14.74) | Breast cancer | 7.66 (-4.06 to 19.38) |
| **Guam** | Tracheal, bronchus, and lung cancer | 4.21 (2.61 to 5.81) | Breast cancer | 3.5 (-4.37 to 11.37) |
| **Guatemala** | Prostate cancer | 4.28 (4.18 to 4.38) | Cervical cancer | 3.36 (2.82 to 3.9) |
| **Guinea** | Liver cancer | 1.51 (1.46 to 1.56) | Cervical cancer | 3.53 (3.13 to 3.94) |
| **Guinea-Bissau** | Liver cancer | 1.41 (1.33 to 1.48) | Cervical cancer | 3.53 (2.83 to 4.23) |
| **Guyana** | Prostate cancer | 4.37 (4.1 to 4.65) | Breast cancer | 4.42 (2.87 to 5.96) |
| **Haiti** | Prostate cancer | 3.3 (3.25 to 3.35) | Cervical cancer | 3.56 (3.25 to 3.87) |
| **Honduras** | Prostate cancer | 2.82 (2.77 to 2.88) | Breast cancer | 3.17 (2.83 to 3.5) |
| **Hungary** | Colon and rectum cancer | 7.03 (6.74 to 7.32) | Breast cancer | 7.54 (5.59 to 9.49) |
| **Iceland** | Prostate cancer | 16.28 (7.81 to 24.75) | Breast cancer | 11.81 (-16.61 to 40.23) |
| **India** | Lip and oral cavity cancer | 1.03 (1.02 to 1.03) | Breast cancer | 2.34 (2.31 to 2.37) |
| **Indonesia** | Tracheal, bronchus, and lung cancer | 2.81 (2.8 to 2.83) | Breast cancer | 3.35 (3.25 to 3.45) |
| **Iran (Islamic Republic of)** | Prostate cancer | 3.44 (3.4 to 3.47) | Breast cancer | 5.58 (4.91 to 6.25) |
| **Iraq** | Tracheal, bronchus, and lung cancer | 2.44 (2.41 to 2.47) | Breast cancer | 6.24 (5.44 to 7.04) |
| **Ireland** | Prostate cancer | 11.25 (10 to 12.5) | Breast cancer | 11.63 (4.15 to 19.1) |
| **Israel** | Colon and rectum cancer | 6.33 (5.67 to 6.99) | Breast cancer | 10.4 (4.57 to 16.22) |
| **Italy** | Prostate cancer | 9.78 (9.5 to 10.06) | Breast cancer | 11.59 (9.19 to 14) |
| **Jamaica** | Prostate cancer | 14.45 (13.18 to 15.71) | Breast cancer | 8.6 (5.28 to 11.91) |
| **Japan** | Colon and rectum cancer | 10.89 (10.52 to 11.27) | Breast cancer | 8.59 (7.06 to 10.11) |
| **Jordan** | Prostate cancer | 3.25 (3.12 to 3.37) | Breast cancer | 8.07 (5.42 to 10.72) |
| **Kazakhstan** | Tracheal, bronchus, and lung cancer | 2.43 (2.39 to 2.47) | Breast cancer | 3.33 (2.96 to 3.7) |
| **Kenya** | Prostate cancer | 1.16 (1.16 to 1.17) | Breast cancer | 2.69 (2.59 to 2.8) |
| **Kiribati** | Stomach cancer | 2.14 (1.55 to 2.73) | Cervical cancer | 5.97 (-2.71 to 14.64) |
| **Kuwait** | Prostate cancer | 6.97 (6.07 to 7.88) | Breast cancer | 8.66 (0.98 to 16.34) |
| **Kyrgyzstan** | Stomach cancer | 2.41 (2.29 to 2.52) | Breast cancer | 2.81 (2.02 to 3.59) |
| **Lao People's Democratic Republic** | Tracheal, bronchus, and lung cancer | 2.84 (2.74 to 2.95) | Breast cancer | 2.78 (2.29 to 3.28) |
| **Latvia** | Prostate cancer | 7.83 (7.4 to 8.26) | Breast cancer | 6.42 (4.04 to 8.79) |
| **Lebanon** | Prostate cancer | 7.09 (6.74 to 7.45) | Breast cancer | 11.5 (7.39 to 15.61) |
| **Lesotho** | Tracheal, bronchus, and lung cancer | 1.08 (1.06 to 1.1) | Cervical cancer | 3.24 (3.02 to 3.47) |
| **Liberia** | Prostate cancer | 2.12 (2.06 to 2.18) | Cervical cancer | 2.87 (2.43 to 3.31) |
| **Libya** | Tracheal, bronchus, and lung cancer | 4.2 (3.99 to 4.41) | Breast cancer | 4.91 (3.95 to 5.87) |
| **Lithuania** | Prostate cancer | 11.99 (11.27 to 12.72) | Breast cancer | 6.34 (3.75 to 8.93) |
| **Luxembourg** | Prostate cancer | 9.9 (7.29 to 12.52) | Breast cancer | 11.03 (-8.05 to 30.11) |
| **Madagascar** | Prostate cancer | 1.26 (1.25 to 1.27) | Cervical cancer | 2.97 (2.75 to 3.2) |
| **Malawi** | Esophageal cancer | 1.85 (1.82 to 1.88) | Cervical cancer | 3.35 (3.11 to 3.59) |
| **Malaysia** | Colon and rectum cancer | 3.03 (2.97 to 3.09) | Breast cancer | 6.39 (5.39 to 7.39) |
| **Maldives** | Prostate cancer | 2.12 (1.74 to 2.5) | Breast cancer | 2.73 (-3.21 to 8.66) |
| **Mali** | Liver cancer | 2.37 (2.3 to 2.44) | Cervical cancer | 2.3 (2.13 to 2.46) |
| **Malta** | Prostate cancer | 8.25 (5.85 to 10.65) | Breast cancer | 11.23 (-14.97 to 37.43) |
| **Marshall Islands** | Tracheal, bronchus, and lung cancer | 3.06 (1.97 to 4.14) | Breast cancer | 3.69 (-0.57 to 7.94) |
| **Mauritania** | Prostate cancer | 3.79 (3.61 to 3.97) | Breast cancer | 3.04 (2.46 to 3.61) |
| **Mauritius** | Prostate cancer | 3.43 (3.24 to 3.62) | Breast cancer | 5.86 (2.01 to 9.71) |
| **Mexico** | Prostate cancer | 5.08 (5.04 to 5.12) | Breast cancer | 5.25 (4.99 to 5.51) |
| **Micronesia (Federated States of)** | Tracheal, bronchus, and lung cancer | 3.53 (2.51 to 4.55) | Breast cancer | 4.52 (-0.54 to 9.58) |
| **Monaco** | Tracheal, bronchus, and lung cancer | 13.56 (-5.23 to 32.36) | Breast cancer | 19.45 (-85.94 to 124.83) |
| **Mongolia** | Liver cancer | 7.4 (6.73 to 8.07) | Liver cancer | 8.1 (6.85 to 9.36) |
| **Montenegro** | Tracheal, bronchus, and lung cancer | 7.59 (6.16 to 9.02) | Breast cancer | 9.14 (-2.3 to 20.58) |
| **Morocco** | Tracheal, bronchus, and lung cancer | 2.67 (2.62 to 2.71) | Breast cancer | 2.94 (2.62 to 3.26) |
| **Mozambique** | Liver cancer | 1.32 (1.31 to 1.33) | Cervical cancer | 3.43 (3.27 to 3.59) |
| **Myanmar** | Tracheal, bronchus, and lung cancer | 2.26 (2.23 to 2.28) | Breast cancer | 3.11 (2.87 to 3.35) |
| **Namibia** | Prostate cancer | 1.86 (1.82 to 1.91) | Breast cancer | 4.06 (3.55 to 4.57) |
| **Nauru** | Tracheal, bronchus, and lung cancer | 3.45 (0.84 to 6.05) | Breast cancer | 4.71 (-8.56 to 17.97) |
| **Nepal** | Lip and oral cavity cancer | 0.78 (0.76 to 0.79) | Cervical cancer | 1.5 (1.37 to 1.63) |
| **Netherlands** | Colon and rectum cancer | 13.31 (12.09 to 14.53) | Breast cancer | 11.85 (7.83 to 15.87) |
| **New Zealand** | Prostate cancer | 16.2 (14 to 18.4) | Breast cancer | 12.5 (5.5 to 19.49) |
| **Nicaragua** | Prostate cancer | 4.87 (4.65 to 5.09) | Breast cancer | 3.49 (2.56 to 4.43) |
| **Niger** | Prostate cancer | 1.75 (1.73 to 1.77) | Cervical cancer | 2.62 (2.41 to 2.83) |
| **Nigeria** | Prostate cancer | 4.37 (4.34 to 4.4) | Breast cancer | 4.37 (4.27 to 4.47) |
| **Niue** | Prostate cancer | 3.93 (-1.25 to 9.12) | Breast cancer | 6.21 (-85.94 to 98.36) |
| **North Macedonia** | Tracheal, bronchus, and lung cancer | 4.79 (4.43 to 5.15) | Breast cancer | 6.88 (3.83 to 9.93) |
| **Northern Mariana Islands** | Tracheal, bronchus, and lung cancer | 5.35 (2.76 to 7.95) | Breast cancer | 6.31 (-10.25 to 22.87) |
| **Norway** | Prostate cancer | 14.71 (12.97 to 16.45) | Colon and rectum cancer | 8.95 (7.01 to 10.89) |
| **Oman** | Prostate cancer | 1.62 (1.56 to 1.69) | Breast cancer | 2.4 (1.6 to 3.2) |
| **Pakistan** | Tracheal, bronchus, and lung cancer | 1.81 (1.8 to 1.82) | Breast cancer | 4.21 (4.1 to 4.33) |
| **Palau** | Prostate cancer | 4.38 (1.81 to 6.94) | Breast cancer | 7.23 (-20.51 to 34.96) |
| **Palestine** | Prostate cancer | 4.79 (4.5 to 5.08) | Breast cancer | 8.6 (4.03 to 13.18) |
| **Panama** | Prostate cancer | 12.58 (11.2 to 13.96) | Breast cancer | 8.61 (3.9 to 13.31) |
| **Papua New Guinea** | Tracheal, bronchus, and lung cancer | 1.76 (1.72 to 1.79) | Breast cancer | 2.36 (2.1 to 2.62) |
| **Paraguay** | Prostate cancer | 4.49 (4.35 to 4.62) | Breast cancer | 4.91 (3.74 to 6.08) |
| **Peru** | Prostate cancer | 4.26 (4.21 to 4.32) | Breast cancer | 3.73 (3.39 to 4.06) |
| **Philippines** | Tracheal, bronchus, and lung cancer | 1.94 (1.93 to 1.95) | Breast cancer | 4.27 (4.04 to 4.49) |
| **Poland** | Tracheal, bronchus, and lung cancer | 6.72 (6.6 to 6.84) | Breast cancer | 7.45 (6.32 to 8.58) |
| **Portugal** | Prostate cancer | 10.59 (9.96 to 11.22) | Breast cancer | 9.26 (4.73 to 13.79) |
| **Puerto Rico** | Prostate cancer | 11.32 (10.26 to 12.38) | Breast cancer | 8.99 (3.63 to 14.35) |
| **Qatar** | Prostate cancer | 11.56 (9.1 to 14.02) | Breast cancer | 15.6 (-0.12 to 31.33) |
| **Republic of Korea** | Tracheal, bronchus, and lung cancer | 10.07 (9.79 to 10.35) | Colon and rectum cancer | 5.76 (5.32 to 6.2) |
| **Republic of Moldova** | Colon and rectum cancer | 4.12 (3.94 to 4.29) | Breast cancer | 5.48 (3.95 to 7.02) |
| **Romania** | Tracheal, bronchus, and lung cancer | 5.21 (5.08 to 5.34) | Breast cancer | 6.24 (5.13 to 7.34) |
| **Russian Federation** | Prostate cancer | 4.66 (4.64 to 4.68) | Breast cancer | 6.08 (5.88 to 6.28) |
| **Rwanda** | Prostate cancer | 2.03 (2 to 2.07) | Cervical cancer | 2.99 (2.65 to 3.34) |
| **Saint Kitts and Nevis** | Prostate cancer | 14.13 (8.19 to 20.07) | Breast cancer | 7.71 (-9.42 to 24.84) |
| **Saint Lucia** | Prostate cancer | 13.37 (9.85 to 16.9) | Breast cancer | 6.6 (-3.37 to 16.57) |
| **Saint Vincent and the Grenadines** | Prostate cancer | 13.46 (8.79 to 18.13) | Breast cancer | 7.64 (-5.1 to 20.38) |
| **Samoa** | Prostate cancer | 2.09 (1.79 to 2.39) | Breast cancer | 3.57 (0.32 to 6.83) |
| **San Marino** | Prostate cancer | 9.31 (-3.95 to 22.57) | Breast cancer | 7.76 (-72.3 to 87.83) |
| **Sao Tome and Principe** | Tracheal, bronchus, and lung cancer | 2.64 (2.06 to 3.21) | Cervical cancer | 3.82 (-2.84 to 10.48) |
| **Saudi Arabia** | Prostate cancer | 2.39 (2.34 to 2.43) | Breast cancer | 4.78 (4.32 to 5.25) |
| **Senegal** | Prostate cancer | 2.56 (2.52 to 2.6) | Cervical cancer | 2.76 (2.45 to 3.08) |
| **Serbia** | Tracheal, bronchus, and lung cancer | 7.19 (6.77 to 7.61) | Breast cancer | 8.5 (5.78 to 11.22) |
| **Seychelles** | Prostate cancer | 8.64 (5.84 to 11.45) | Breast cancer | 5.21 (-7.38 to 17.79) |
| **Sierra Leone** | Prostate cancer | 2.06 (2.02 to 2.1) | Cervical cancer | 2.71 (2.4 to 3.03) |
| **Singapore** | Colon and rectum cancer | 7.66 (6.29 to 9.03) | Breast cancer | 8.24 (0.86 to 15.62) |
| **Slovakia** | Colon and rectum cancer | 7.55 (7.14 to 7.95) | Breast cancer | 7.45 (4.99 to 9.91) |
| **Slovenia** | Prostate cancer | 10.4 (9.24 to 11.56) | Breast cancer | 8.59 (-0.09 to 17.27) |
| **Solomon Islands** | Tracheal, bronchus, and lung cancer | 2.17 (1.93 to 2.41) | Cervical cancer | 2.77 (1.3 to 4.24) |
| **Somalia** | Esophageal cancer | 0.69 (0.68 to 0.69) | Cervical cancer | 3.56 (3.37 to 3.76) |
| **South Africa** | Prostate cancer | 2.7 (2.68 to 2.72) | Breast cancer | 3.54 (3.46 to 3.62) |
| **South Sudan** | Prostate cancer | 1.23 (1.21 to 1.24) | Cervical cancer | 3.45 (3.1 to 3.81) |
| **Spain** | Colon and rectum cancer | 10.01 (9.6 to 10.43) | Breast cancer | 8.71 (6.52 to 10.9) |
| **Sri Lanka** | Prostate cancer | 1.66 (1.63 to 1.68) | Breast cancer | 3.64 (2.79 to 4.48) |
| **Sudan** | Stomach cancer | 1.6 (1.57 to 1.62) | Breast cancer | 2.1 (1.96 to 2.24) |
| **Suriname** | Prostate cancer | 5.65 (5.04 to 6.26) | Breast cancer | 4.12 (1.86 to 6.38) |
| **Sweden** | Prostate cancer | 14.23 (13.04 to 15.42) | Breast cancer | 9.93 (5.52 to 14.33) |
| **Switzerland** | Prostate cancer | 14.58 (13.21 to 15.95) | Breast cancer | 10.29 (4.74 to 15.84) |
| **Syrian Arab Republic** | Prostate cancer | 2.83 (2.78 to 2.89) | Breast cancer | 4.73 (3.67 to 5.78) |
| **Taiwan (Province of China)** | Colon and rectum cancer | 9.3 (8.72 to 9.87) | Colon and rectum cancer | 7.15 (6.3 to 8) |
| **Tajikistan** | Stomach cancer | 1.74 (1.68 to 1.8) | Breast cancer | 2.01 (1.64 to 2.38) |
| **Thailand** | Tracheal, bronchus, and lung cancer | 4.36 (4.3 to 4.43) | Breast cancer | 5.15 (4.55 to 5.74) |
| **Timor-Leste** | Tracheal, bronchus, and lung cancer | 1.9 (1.76 to 2.05) | Breast cancer | 2.15 (1.33 to 2.97) |
| **Togo** | Prostate cancer | 2.15 (2.11 to 2.19) | Cervical cancer | 2.83 (2.47 to 3.19) |
| **Tokelau** | Prostate cancer | 4.68 (-5.33 to 14.7) | Breast cancer | 5.7 (-81.42 to 92.82) |
| **Tonga** | Prostate cancer | 5.63 (3.96 to 7.31) | Breast cancer | 7.11 (-8.81 to 23.02) |
| **Trinidad and Tobago** | Prostate cancer | 9.78 (9.04 to 10.52) | Breast cancer | 7.27 (4.06 to 10.49) |
| **Tunisia** | Tracheal, bronchus, and lung cancer | 3.3 (3.18 to 3.42) | Breast cancer | 5.35 (4.07 to 6.62) |
| **Turkey** | Tracheal, bronchus, and lung cancer | 6.58 (6.44 to 6.72) | Breast cancer | 6.64 (5.75 to 7.53) |
| **Turkmenistan** | Tracheal, bronchus, and lung cancer | 1.19 (1.15 to 1.22) | Breast cancer | 2.63 (1.98 to 3.29) |
| **Tuvalu** | Tracheal, bronchus, and lung cancer | 3.04 (0.68 to 5.41) | Breast cancer | 4.15 (-11.14 to 19.44) |
| **Uganda** | Prostate cancer | 3.32 (3.28 to 3.36) | Breast cancer | 3.34 (3.18 to 3.49) |
| **Ukraine** | Tracheal, bronchus, and lung cancer | 3.47 (3.42 to 3.51) | Breast cancer | 4.16 (3.85 to 4.46) |
| **United Arab Emirates** | Prostate cancer | 5.02 (4.57 to 5.47) | Breast cancer | 11.14 (7.36 to 14.93) |
| **United Kingdom** | Prostate cancer | 11.8 (11.54 to 12.05) | Breast cancer | 11.73 (10.31 to 13.16) |
| **United Republic of Tanzania** | Prostate cancer | 1.84 (1.83 to 1.85) | Cervical cancer | 2.9 (2.77 to 3.04) |
| **United States of America** | Prostate cancer | 13.2 (13.07 to 13.33) | Breast cancer | 12.51 (12.08 to 12.94) |
| **United States Virgin Islands** | Prostate cancer | 10.2 (6.51 to 13.9) | Breast cancer | 7.91 (-19.22 to 35.05) |
| **Uruguay** | Prostate cancer | 7.65 (7.26 to 8.03) | Breast cancer | 9.35 (5.44 to 13.26) |
| **Uzbekistan** | Stomach cancer | 1.14 (1.12 to 1.16) | Breast cancer | 2.6 (2.23 to 2.96) |
| **Vanuatu** | Tracheal, bronchus, and lung cancer | 1.94 (1.71 to 2.17) | Breast cancer | 2.7 (1.06 to 4.35) |
| **Venezuela (Bolivarian Republic of)** | Prostate cancer | 8.7 (8.53 to 8.87) | Breast cancer | 7.56 (6.66 to 8.47) |
| **Viet Nam** | Tracheal, bronchus, and lung cancer | 3.57 (3.52 to 3.62) | Breast cancer | 2.98 (2.66 to 3.31) |
| **Yemen** | Stomach cancer | 1.82 (1.8 to 1.85) | Breast cancer | 1.69 (1.58 to 1.8) |
| **Zambia** | Prostate cancer | 2.03 (2.01 to 2.06) | Cervical cancer | 3.32 (3.13 to 3.52) |
| **Zimbabwe** | Prostate cancer | 1.54 (1.53 to 1.55) | Cervical cancer | 3.91 (3.73 to 4.1) |

Table S13. Types of cancer with the highest lifetime death risk across 204 countries and territories by sex in 2021.

| **Location** | **Male** | | **Female** | |
| --- | --- | --- | --- | --- |
|  | **Cancer type** | **Lifetime death risk** | **Cancer type** | **Lifetime death risk** |
| **Afghanistan** | Stomach cancer | 1.47 (1.45 to 1.49) | Stomach cancer | 2.75 (2.69 to 2.82) |
| **Albania** | Tracheal, bronchus, and lung cancer | 4.73 (4.31 to 5.15) | Tracheal, bronchus, and lung cancer | 1.8 (1.57 to 2.03) |
| **Algeria** | Tracheal, bronchus, and lung cancer | 1.26 (1.24 to 1.27) | Breast cancer | 0.92 (0.88 to 0.96) |
| **American Samoa** | Prostate cancer | 5.45 (3.7 to 7.19) | Breast cancer | 4.56 (-1.56 to 10.68) |
| **Andorra** | Tracheal, bronchus, and lung cancer | 7.38 (1.43 to 13.32) | Breast cancer | 3.6 (-4.78 to 11.98) |
| **Angola** | Tracheal, bronchus, and lung cancer | 1.27 (1.26 to 1.28) | Cervical cancer | 1.9 (1.84 to 1.96) |
| **Antigua and Barbuda** | Prostate cancer | 8.98 (6.65 to 11.31) | Breast cancer | 4.23 (-0.45 to 8.92) |
| **Argentina** | Tracheal, bronchus, and lung cancer | 3.83 (3.76 to 3.9) | Breast cancer | 3.57 (3.37 to 3.76) |
| **Armenia** | Tracheal, bronchus, and lung cancer | 5.9 (5.46 to 6.34) | Breast cancer | 2.92 (2.29 to 3.56) |
| **Australia** | Tracheal, bronchus, and lung cancer | 5.52 (5.29 to 5.75) | Tracheal, bronchus, and lung cancer | 3.77 (3.5 to 4.04) |
| **Austria** | Tracheal, bronchus, and lung cancer | 4.88 (4.57 to 5.18) | Breast cancer | 3.41 (2.84 to 3.99) |
| **Azerbaijan** | Tracheal, bronchus, and lung cancer | 2.43 (2.36 to 2.5) | Breast cancer | 1.6 (1.42 to 1.78) |
| **Bahamas** | Prostate cancer | 5.33 (4.8 to 5.85) | Breast cancer | 4.24 (2.5 to 5.97) |
| **Bahrain** | Tracheal, bronchus, and lung cancer | 3.05 (2.81 to 3.29) | Breast cancer | 2.83 (1.72 to 3.95) |
| **Bangladesh** | Tracheal, bronchus, and lung cancer | 0.91 (0.9 to 0.91) | Cervical cancer | 0.89 (0.87 to 0.91) |
| **Barbados** | Prostate cancer | 7.87 (6.85 to 8.9) | Breast cancer | 5.08 (1.94 to 8.21) |
| **Belarus** | Tracheal, bronchus, and lung cancer | 3.83 (3.74 to 3.91) | Colon and rectum cancer | 1.85 (1.8 to 1.91) |
| **Belgium** | Tracheal, bronchus, and lung cancer | 7.76 (7.28 to 8.24) | Breast cancer | 4.06 (3.53 to 4.59) |
| **Belize** | Prostate cancer | 4.42 (3.81 to 5.02) | Cervical cancer | 2.12 (0.91 to 3.32) |
| **Benin** | Prostate cancer | 1.8 (1.78 to 1.83) | Cervical cancer | 1.65 (1.55 to 1.75) |
| **Bermuda** | Prostate cancer | 6.86 (5.33 to 8.38) | Breast cancer | 3.97 (-2.2 to 10.14) |
| **Bhutan** | Tracheal, bronchus, and lung cancer | 0.93 (0.84 to 1.03) | Cervical cancer | 1.07 (0.64 to 1.5) |
| **Bolivia (Plurinational State of)** | Stomach cancer | 2.32 (2.28 to 2.36) | Stomach cancer | 2.86 (2.79 to 2.94) |
| **Bosnia and Herzegovina** | Tracheal, bronchus, and lung cancer | 6.77 (6.26 to 7.27) | Breast cancer | 2.38 (1.96 to 2.8) |
| **Botswana** | Prostate cancer | 1.37 (1.34 to 1.39) | Breast cancer | 1.58 (1.5 to 1.66) |
| **Brazil** | Prostate cancer | 2.71 (2.7 to 2.72) | Breast cancer | 2.29 (2.25 to 2.33) |
| **Brunei Darussalam** | Tracheal, bronchus, and lung cancer | 4.44 (3.45 to 5.43) | Tracheal, bronchus, and lung cancer | 3.67 (2.54 to 4.8) |
| **Bulgaria** | Tracheal, bronchus, and lung cancer | 4.64 (4.5 to 4.78) | Breast cancer | 2.53 (2.3 to 2.77) |
| **Burkina Faso** | Liver cancer | 1.54 (1.51 to 1.58) | Breast cancer | 1.98 (1.9 to 2.06) |
| **Burundi** | Prostate cancer | 1.34 (1.32 to 1.35) | Cervical cancer | 2.15 (1.99 to 2.31) |
| **Cabo Verde** | Prostate cancer | 4.75 (4.3 to 5.2) | Stomach cancer | 2.27 (1.98 to 2.57) |
| **Cambodia** | Tracheal, bronchus, and lung cancer | 3.85 (3.76 to 3.94) | Breast cancer | 2.05 (1.91 to 2.19) |
| **Cameroon** | Prostate cancer | 1.56 (1.55 to 1.57) | Cervical cancer | 1.73 (1.68 to 1.78) |
| **Canada** | Tracheal, bronchus, and lung cancer | 6.21 (6.05 to 6.37) | Tracheal, bronchus, and lung cancer | 5.63 (5.38 to 5.88) |
| **Central African Republic** | Tracheal, bronchus, and lung cancer | 0.71 (0.7 to 0.72) | Cervical cancer | 2.22 (2.11 to 2.34) |
| **Chad** | Prostate cancer | 1.49 (1.47 to 1.51) | Cervical cancer | 2.12 (2.01 to 2.23) |
| **Chile** | Prostate cancer | 4.18 (4.11 to 4.24) | Colon and rectum cancer | 2.53 (2.44 to 2.62) |
| **China** | Tracheal, bronchus, and lung cancer | 7.32 (7.29 to 7.36) | Tracheal, bronchus, and lung cancer | 4.39 (4.36 to 4.42) |
| **Colombia** | Prostate cancer | 2.55 (2.53 to 2.57) | Breast cancer | 2.02 (1.91 to 2.13) |
| **Comoros** | Prostate cancer | 2.15 (2.02 to 2.28) | Cervical cancer | 2.35 (1.53 to 3.18) |
| **Congo** | Prostate cancer | 1.5 (1.48 to 1.53) | Cervical cancer | 2.25 (2.1 to 2.39) |
| **Cook Islands** | Prostate cancer | 8.37 (3.75 to 13) | Breast cancer | 6.17 (-16.72 to 29.05) |
| **Costa Rica** | Prostate cancer | 3.82 (3.69 to 3.96) | Colon and rectum cancer | 2.76 (2.54 to 2.98) |
| **Cote d’Ivoire** | Prostate cancer | 2.83 (2.8 to 2.87) | Breast cancer | 2.2 (2.12 to 2.28) |
| **Croatia** | Tracheal, bronchus, and lung cancer | 6.69 (6.22 to 7.16) | Colon and rectum cancer | 3.06 (2.83 to 3.28) |
| **Cuba** | Tracheal, bronchus, and lung cancer | 4.49 (4.39 to 4.6) | Tracheal, bronchus, and lung cancer | 3.23 (3.1 to 3.36) |
| **Cyprus** | Tracheal, bronchus, and lung cancer | 5.34 (4.33 to 6.35) | Breast cancer | 3.7 (1.59 to 5.81) |
| **Czechia** | Tracheal, bronchus, and lung cancer | 5.19 (5.03 to 5.36) | Tracheal, bronchus, and lung cancer | 2.93 (2.74 to 3.12) |
| **Democratic People's Republic of Korea** | Tracheal, bronchus, and lung cancer | 4.05 (3.96 to 4.14) | Tracheal, bronchus, and lung cancer | 2.02 (1.96 to 2.08) |
| **Democratic Republic of the Congo** | Prostate cancer | 1.18 (1.17 to 1.18) | Cervical cancer | 2.22 (2.17 to 2.27) |
| **Denmark** | Tracheal, bronchus, and lung cancer | 6.62 (6.16 to 7.08) | Tracheal, bronchus, and lung cancer | 6.46 (5.57 to 7.35) |
| **Djibouti** | Prostate cancer | 1.76 (1.69 to 1.83) | Breast cancer | 2.1 (1.82 to 2.38) |
| **Dominica** | Prostate cancer | 5.27 (4.24 to 6.31) | Breast cancer | 3.71 (-0.41 to 7.82) |
| **Dominican Republic** | Prostate cancer | 4.39 (4.29 to 4.49) | Breast cancer | 2.03 (1.85 to 2.2) |
| **Ecuador** | Prostate cancer | 2.75 (2.72 to 2.78) | Stomach cancer | 2.5 (2.42 to 2.58) |
| **Egypt** | Liver cancer | 1.99 (1.98 to 2.01) | Breast cancer | 1.73 (1.69 to 1.78) |
| **El Salvador** | Prostate cancer | 2.46 (2.41 to 2.52) | Stomach cancer | 2.31 (2.19 to 2.42) |
| **Equatorial Guinea** | Prostate cancer | 1.43 (1.38 to 1.48) | Breast cancer | 2.01 (1.77 to 2.25) |
| **Eritrea** | Prostate cancer | 1.25 (1.23 to 1.27) | Cervical cancer | 2.79 (2.53 to 3.04) |
| **Estonia** | Tracheal, bronchus, and lung cancer | 5.42 (4.97 to 5.87) | Colon and rectum cancer | 2.64 (2.38 to 2.9) |
| **Eswatini** | Liver cancer | 1.13 (1.09 to 1.16) | Cervical cancer | 1.94 (1.8 to 2.09) |
| **Ethiopia** | Colon and rectum cancer | 1.5 (1.49 to 1.51) | Breast cancer | 1.86 (1.83 to 1.89) |
| **Fiji** | Prostate cancer | 1.06 (1.04 to 1.09) | Breast cancer | 3.46 (2.63 to 4.29) |
| **Finland** | Tracheal, bronchus, and lung cancer | 4.93 (4.63 to 5.23) | Breast cancer | 3.17 (2.46 to 3.87) |
| **France** | Tracheal, bronchus, and lung cancer | 7.22 (6.98 to 7.47) | Breast cancer | 4.15 (3.86 to 4.44) |
| **Gabon** | Tracheal, bronchus, and lung cancer | 1.63 (1.57 to 1.7) | Breast cancer | 2.35 (2.07 to 2.63) |
| **Gambia** | Liver cancer | 3.27 (2.95 to 3.6) | Liver cancer | 1.62 (1.5 to 1.74) |
| **Georgia** | Tracheal, bronchus, and lung cancer | 4.54 (4.33 to 4.75) | Breast cancer | 3.48 (2.82 to 4.15) |
| **Germany** | Tracheal, bronchus, and lung cancer | 5.73 (5.62 to 5.84) | Breast cancer | 3.79 (3.57 to 4.02) |
| **Ghana** | Prostate cancer | 2.12 (2.1 to 2.13) | Breast cancer | 1.87 (1.82 to 1.93) |
| **Greece** | Tracheal, bronchus, and lung cancer | 9.07 (8.53 to 9.6) | Breast cancer | 4.09 (3.48 to 4.7) |
| **Greenland** | Tracheal, bronchus, and lung cancer | 8.98 (3.44 to 14.52) | Tracheal, bronchus, and lung cancer | 7.58 (-0.73 to 15.88) |
| **Grenada** | Prostate cancer | 5.87 (4.95 to 6.79) | Breast cancer | 3.42 (0.82 to 6.02) |
| **Guam** | Tracheal, bronchus, and lung cancer | 4.49 (2.9 to 6.09) | Tracheal, bronchus, and lung cancer | 2.43 (1.03 to 3.82) |
| **Guatemala** | Stomach cancer | 2.22 (2.17 to 2.26) | Stomach cancer | 3.05 (2.94 to 3.16) |
| **Guinea** | Liver cancer | 1.55 (1.5 to 1.6) | Cervical cancer | 2.39 (2.23 to 2.55) |
| **Guinea-Bissau** | Liver cancer | 1.42 (1.35 to 1.5) | Cervical cancer | 2.42 (2.13 to 2.72) |
| **Guyana** | Prostate cancer | 2.67 (2.55 to 2.79) | Breast cancer | 2.32 (1.85 to 2.79) |
| **Haiti** | Prostate cancer | 2.63 (2.6 to 2.67) | Cervical cancer | 2.24 (2.14 to 2.35) |
| **Honduras** | Tracheal, bronchus, and lung cancer | 1.82 (1.78 to 1.86) | Stomach cancer | 2.12 (2.06 to 2.18) |
| **Hungary** | Tracheal, bronchus, and lung cancer | 6.39 (6.17 to 6.6) | Tracheal, bronchus, and lung cancer | 4.34 (4.04 to 4.64) |
| **Iceland** | Prostate cancer | 6.49 (5.42 to 7.56) | Tracheal, bronchus, and lung cancer | 6.21 (0.75 to 11.68) |
| **India** | Tracheal, bronchus, and lung cancer | 0.82 (0.82 to 0.82) | Breast cancer | 1.31 (1.3 to 1.32) |
| **Indonesia** | Tracheal, bronchus, and lung cancer | 3 (2.98 to 3.01) | Breast cancer | 1.99 (1.95 to 2.02) |
| **Iran (Islamic Republic of)** | Stomach cancer | 1.89 (1.88 to 1.91) | Stomach cancer | 1.48 (1.46 to 1.5) |
| **Iraq** | Tracheal, bronchus, and lung cancer | 2.64 (2.6 to 2.67) | Breast cancer | 1.75 (1.64 to 1.86) |
| **Ireland** | Tracheal, bronchus, and lung cancer | 5.26 (4.75 to 5.77) | Tracheal, bronchus, and lung cancer | 4.4 (3.64 to 5.17) |
| **Israel** | Tracheal, bronchus, and lung cancer | 4.95 (4.51 to 5.39) | Breast cancer | 4.09 (3.12 to 5.05) |
| **Italy** | Tracheal, bronchus, and lung cancer | 6.9 (6.74 to 7.07) | Breast cancer | 3.67 (3.36 to 3.99) |
| **Jamaica** | Prostate cancer | 7.21 (6.86 to 7.56) | Breast cancer | 3.61 (2.91 to 4.32) |
| **Japan** | Tracheal, bronchus, and lung cancer | 7.79 (7.68 to 7.89) | Colon and rectum cancer | 4.12 (4.03 to 4.21) |
| **Jordan** | Tracheal, bronchus, and lung cancer | 2.04 (1.96 to 2.12) | Breast cancer | 2.1 (1.79 to 2.42) |
| **Kazakhstan** | Tracheal, bronchus, and lung cancer | 2.44 (2.4 to 2.48) | Breast cancer | 1.43 (1.36 to 1.5) |
| **Kenya** | Prostate cancer | 0.88 (0.88 to 0.88) | Breast cancer | 1.74 (1.7 to 1.78) |
| **Kiribati** | Tracheal, bronchus, and lung cancer | 2.17 (1.74 to 2.6) | Cervical cancer | 4.02 (0.79 to 7.25) |
| **Kuwait** | Prostate cancer | 1.97 (1.9 to 2.04) | Breast cancer | 2 (1.25 to 2.74) |
| **Kyrgyzstan** | Stomach cancer | 2.35 (2.25 to 2.45) | Breast cancer | 1.36 (1.18 to 1.54) |
| **Lao People's Democratic Republic** | Tracheal, bronchus, and lung cancer | 3.05 (2.95 to 3.16) | Breast cancer | 1.86 (1.66 to 2.07) |
| **Latvia** | Tracheal, bronchus, and lung cancer | 4.3 (4.08 to 4.52) | Breast cancer | 2.48 (2.08 to 2.88) |
| **Lebanon** | Tracheal, bronchus, and lung cancer | 4.12 (3.91 to 4.33) | Breast cancer | 2.84 (2.39 to 3.3) |
| **Lesotho** | Tracheal, bronchus, and lung cancer | 1.07 (1.05 to 1.09) | Cervical cancer | 2.11 (2.02 to 2.21) |
| **Liberia** | Prostate cancer | 1.8 (1.76 to 1.84) | Cervical cancer | 1.89 (1.73 to 2.05) |
| **Libya** | Tracheal, bronchus, and lung cancer | 4.54 (4.32 to 4.76) | Colon and rectum cancer | 1.56 (1.49 to 1.62) |
| **Lithuania** | Tracheal, bronchus, and lung cancer | 4.69 (4.47 to 4.91) | Breast cancer | 2.4 (2.02 to 2.77) |
| **Luxembourg** | Tracheal, bronchus, and lung cancer | 6.75 (4.73 to 8.77) | Breast cancer | 3.8 (1.21 to 6.39) |
| **Madagascar** | Prostate cancer | 1.06 (1.05 to 1.06) | Cervical cancer | 1.95 (1.87 to 2.03) |
| **Malawi** | Esophageal cancer | 1.88 (1.85 to 1.9) | Cervical cancer | 2.1 (2.02 to 2.19) |
| **Malaysia** | Tracheal, bronchus, and lung cancer | 2.72 (2.68 to 2.77) | Breast cancer | 3.09 (2.83 to 3.35) |
| **Maldives** | Tracheal, bronchus, and lung cancer | 2 (1.61 to 2.4) | Breast cancer | 1.37 (0.16 to 2.58) |
| **Mali** | Liver cancer | 2.45 (2.38 to 2.51) | Cervical cancer | 1.5 (1.44 to 1.56) |
| **Malta** | Tracheal, bronchus, and lung cancer | 5.42 (3.45 to 7.38) | Breast cancer | 3.81 (-0.58 to 8.2) |
| **Marshall Islands** | Tracheal, bronchus, and lung cancer | 3.26 (2.12 to 4.39) | Breast cancer | 2.6 (0.67 to 4.53) |
| **Mauritania** | Prostate cancer | 3.07 (2.97 to 3.17) | Cervical cancer | 1.92 (1.69 to 2.15) |
| **Mauritius** | Prostate cancer | 1.96 (1.89 to 2.02) | Breast cancer | 2.92 (2.02 to 3.82) |
| **Mexico** | Prostate cancer | 1.71 (1.7 to 1.71) | Breast cancer | 1.61 (1.58 to 1.64) |
| **Micronesia (Federated States of)** | Tracheal, bronchus, and lung cancer | 3.76 (2.7 to 4.82) | Breast cancer | 3.14 (0.96 to 5.32) |
| **Monaco** | Tracheal, bronchus, and lung cancer | 12.27 (-1.51 to 26.05) | Tracheal, bronchus, and lung cancer | 7.9 (-6.43 to 22.23) |
| **Mongolia** | Liver cancer | 7.83 (7.15 to 8.52) | Liver cancer | 9.08 (7.77 to 10.38) |
| **Montenegro** | Tracheal, bronchus, and lung cancer | 7.25 (6.04 to 8.47) | Breast cancer | 3.07 (1.42 to 4.72) |
| **Morocco** | Tracheal, bronchus, and lung cancer | 2.89 (2.84 to 2.94) | Breast cancer | 1.01 (0.97 to 1.06) |
| **Mozambique** | Liver cancer | 1.36 (1.34 to 1.37) | Cervical cancer | 2.34 (2.26 to 2.41) |
| **Myanmar** | Tracheal, bronchus, and lung cancer | 2.4 (2.38 to 2.42) | Breast cancer | 1.89 (1.81 to 1.97) |
| **Namibia** | Prostate cancer | 1.2 (1.18 to 1.22) | Breast cancer | 2.41 (2.21 to 2.6) |
| **Nauru** | Tracheal, bronchus, and lung cancer | 3.58 (0.94 to 6.22) | Breast cancer | 3.1 (-2.44 to 8.64) |
| **Nepal** | Tracheal, bronchus, and lung cancer | 0.7 (0.69 to 0.71) | Cervical cancer | 0.94 (0.9 to 0.97) |
| **Netherlands** | Tracheal, bronchus, and lung cancer | 7.18 (6.83 to 7.54) | Tracheal, bronchus, and lung cancer | 4.7 (4.24 to 5.17) |
| **New Zealand** | Prostate cancer | 5.19 (5 to 5.37) | Tracheal, bronchus, and lung cancer | 4.45 (3.66 to 5.23) |
| **Nicaragua** | Prostate cancer | 1.63 (1.6 to 1.66) | Cervical cancer | 1.49 (1.3 to 1.69) |
| **Niger** | Prostate cancer | 1.65 (1.63 to 1.67) | Cervical cancer | 1.93 (1.84 to 2.02) |
| **Nigeria** | Prostate cancer | 3.81 (3.78 to 3.83) | Breast cancer | 3.04 (2.99 to 3.09) |
| **Niue** | Tracheal, bronchus, and lung cancer | 4.12 (-3.68 to 11.93) | Breast cancer | 3.81 (-25.24 to 32.87) |
| **North Macedonia** | Tracheal, bronchus, and lung cancer | 4.71 (4.39 to 5.03) | Breast cancer | 2.69 (2.22 to 3.17) |
| **Northern Mariana Islands** | Tracheal, bronchus, and lung cancer | 5.68 (3.07 to 8.28) | Breast cancer | 3.6 (-0.77 to 7.96) |
| **Norway** | Prostate cancer | 6.46 (6.25 to 6.67) | Tracheal, bronchus, and lung cancer | 4.71 (3.93 to 5.49) |
| **Oman** | Tracheal, bronchus, and lung cancer | 0.7 (0.68 to 0.72) | Breast cancer | 0.55 (0.49 to 0.62) |
| **Pakistan** | Tracheal, bronchus, and lung cancer | 1.91 (1.9 to 1.92) | Breast cancer | 2.82 (2.77 to 2.87) |
| **Palau** | Tracheal, bronchus, and lung cancer | 4.42 (1.28 to 7.56) | Tracheal, bronchus, and lung cancer | 5.48 (0.93 to 10.02) |
| **Palestine** | Tracheal, bronchus, and lung cancer | 3.43 (3.2 to 3.66) | Breast cancer | 2.62 (1.9 to 3.33) |
| **Panama** | Prostate cancer | 3.91 (3.75 to 4.06) | Breast cancer | 2.29 (1.78 to 2.79) |
| **Papua New Guinea** | Tracheal, bronchus, and lung cancer | 1.88 (1.84 to 1.92) | Breast cancer | 1.61 (1.49 to 1.73) |
| **Paraguay** | Tracheal, bronchus, and lung cancer | 2.62 (2.54 to 2.7) | Breast cancer | 2.31 (2.05 to 2.56) |
| **Peru** | Stomach cancer | 2.15 (2.12 to 2.18) | Stomach cancer | 2.42 (2.37 to 2.46) |
| **Philippines** | Tracheal, bronchus, and lung cancer | 2.04 (2.02 to 2.05) | Breast cancer | 2.53 (2.46 to 2.61) |
| **Poland** | Tracheal, bronchus, and lung cancer | 7.24 (7.12 to 7.36) | Tracheal, bronchus, and lung cancer | 3.98 (3.85 to 4.12) |
| **Portugal** | Tracheal, bronchus, and lung cancer | 5.15 (4.85 to 5.46) | Colon and rectum cancer | 3.1 (2.91 to 3.28) |
| **Puerto Rico** | Prostate cancer | 3.9 (3.77 to 4.04) | Breast cancer | 2.86 (2.17 to 3.54) |
| **Qatar** | Prostate cancer | 2.94 (2.71 to 3.17) | Breast cancer | 3.32 (1.48 to 5.17) |
| **Republic of Korea** | Tracheal, bronchus, and lung cancer | 8.17 (7.99 to 8.35) | Tracheal, bronchus, and lung cancer | 3.4 (3.29 to 3.5) |
| **Republic of Moldova** | Tracheal, bronchus, and lung cancer | 3.16 (3.03 to 3.29) | Breast cancer | 2.15 (1.88 to 2.41) |
| **Romania** | Tracheal, bronchus, and lung cancer | 5.02 (4.91 to 5.14) | Breast cancer | 2.65 (2.48 to 2.82) |
| **Russian Federation** | Tracheal, bronchus, and lung cancer | 3.64 (3.62 to 3.66) | Colon and rectum cancer | 2.06 (2.05 to 2.07) |
| **Rwanda** | Prostate cancer | 1.65 (1.62 to 1.67) | Breast cancer | 2.04 (1.94 to 2.13) |
| **Saint Kitts and Nevis** | Prostate cancer | 7.51 (5.47 to 9.55) | Breast cancer | 3.64 (-0.32 to 7.59) |
| **Saint Lucia** | Prostate cancer | 7.3 (6.26 to 8.35) | Breast cancer | 2.99 (0.89 to 5.1) |
| **Saint Vincent and the Grenadines** | Prostate cancer | 8.13 (6.48 to 9.79) | Breast cancer | 3.74 (0.51 to 6.97) |
| **Samoa** | Prostate cancer | 1.57 (1.41 to 1.73) | Breast cancer | 2.26 (1.09 to 3.43) |
| **San Marino** | Tracheal, bronchus, and lung cancer | 5.4 (-3.15 to 13.95) | Colon and rectum cancer | 2.85 (-0.15 to 5.86) |
| **Sao Tome and Principe** | Tracheal, bronchus, and lung cancer | 2.86 (2.27 to 3.46) | Cervical cancer | 2.4 (0.53 to 4.28) |
| **Saudi Arabia** | Colon and rectum cancer | 1 (0.99 to 1.01) | Breast cancer | 1.09 (1.04 to 1.14) |
| **Senegal** | Prostate cancer | 2.14 (2.11 to 2.16) | Cervical cancer | 1.89 (1.78 to 2) |
| **Serbia** | Tracheal, bronchus, and lung cancer | 6.75 (6.4 to 7.09) | Breast cancer | 3.39 (2.96 to 3.81) |
| **Seychelles** | Prostate cancer | 5.39 (4.28 to 6.5) | Breast cancer | 2.77 (-0.51 to 6.06) |
| **Sierra Leone** | Prostate cancer | 1.84 (1.8 to 1.87) | Cervical cancer | 1.86 (1.74 to 1.98) |
| **Singapore** | Tracheal, bronchus, and lung cancer | 5.37 (4.92 to 5.82) | Colon and rectum cancer | 3.43 (3.02 to 3.85) |
| **Slovakia** | Tracheal, bronchus, and lung cancer | 4.83 (4.63 to 5.03) | Breast cancer | 2.76 (2.4 to 3.12) |
| **Slovenia** | Tracheal, bronchus, and lung cancer | 6.21 (5.54 to 6.89) | Breast cancer | 3.39 (2.45 to 4.33) |
| **Solomon Islands** | Tracheal, bronchus, and lung cancer | 2.28 (2.03 to 2.52) | Breast cancer | 1.99 (1.49 to 2.48) |
| **Somalia** | Esophageal cancer | 0.69 (0.69 to 0.7) | Cervical cancer | 2.54 (2.45 to 2.64) |
| **South Africa** | Tracheal, bronchus, and lung cancer | 1.85 (1.83 to 1.86) | Breast cancer | 2.04 (2.02 to 2.07) |
| **South Sudan** | Esophageal cancer | 1.06 (1.04 to 1.08) | Cervical cancer | 2.31 (2.17 to 2.46) |
| **Spain** | Tracheal, bronchus, and lung cancer | 7.06 (6.81 to 7.31) | Colon and rectum cancer | 3.14 (3.05 to 3.24) |
| **Sri Lanka** | Tracheal, bronchus, and lung cancer | 1.48 (1.45 to 1.51) | Breast cancer | 1.69 (1.53 to 1.85) |
| **Sudan** | Stomach cancer | 1.55 (1.53 to 1.57) | Stomach cancer | 1.4 (1.37 to 1.43) |
| **Suriname** | Prostate cancer | 3.65 (3.38 to 3.92) | Breast cancer | 2.19 (1.58 to 2.8) |
| **Sweden** | Prostate cancer | 6.32 (6.18 to 6.46) | Tracheal, bronchus, and lung cancer | 3.97 (3.58 to 4.36) |
| **Switzerland** | Prostate cancer | 5.44 (5.3 to 5.57) | Breast cancer | 3.44 (2.74 to 4.15) |
| **Syrian Arab Republic** | Tracheal, bronchus, and lung cancer | 1.83 (1.79 to 1.87) | Breast cancer | 1.25 (1.14 to 1.37) |
| **Taiwan (Province of China)** | Tracheal, bronchus, and lung cancer | 6.2 (6.02 to 6.39) | Tracheal, bronchus, and lung cancer | 4.2 (3.94 to 4.46) |
| **Tajikistan** | Stomach cancer | 1.78 (1.72 to 1.83) | Stomach cancer | 1.28 (1.23 to 1.33) |
| **Thailand** | Tracheal, bronchus, and lung cancer | 4.67 (4.6 to 4.73) | Tracheal, bronchus, and lung cancer | 2.92 (2.87 to 2.98) |
| **Timor-Leste** | Tracheal, bronchus, and lung cancer | 2.05 (1.9 to 2.2) | Breast cancer | 1.42 (1.1 to 1.74) |
| **Togo** | Prostate cancer | 1.78 (1.75 to 1.81) | Cervical cancer | 1.91 (1.78 to 2.04) |
| **Tokelau** | Tracheal, bronchus, and lung cancer | 3.75 (-6.95 to 14.46) | Breast cancer | 3.52 (-25.4 to 32.45) |
| **Tonga** | Tracheal, bronchus, and lung cancer | 5.41 (3.13 to 7.69) | Breast cancer | 4.71 (-1.25 to 10.68) |
| **Trinidad and Tobago** | Prostate cancer | 4.86 (4.65 to 5.07) | Breast cancer | 3.12 (2.45 to 3.8) |
| **Tunisia** | Tracheal, bronchus, and lung cancer | 3.5 (3.38 to 3.62) | Breast cancer | 1.36 (1.23 to 1.49) |
| **Turkey** | Tracheal, bronchus, and lung cancer | 7.03 (6.89 to 7.17) | Breast cancer | 1.69 (1.59 to 1.78) |
| **Turkmenistan** | Tracheal, bronchus, and lung cancer | 1.21 (1.17 to 1.24) | Breast cancer | 1.27 (1.11 to 1.43) |
| **Tuvalu** | Tracheal, bronchus, and lung cancer | 3.27 (0.83 to 5.71) | Breast cancer | 2.83 (-3.24 to 8.9) |
| **Uganda** | Prostate cancer | 2.59 (2.56 to 2.61) | Breast cancer | 2.26 (2.19 to 2.33) |
| **Ukraine** | Tracheal, bronchus, and lung cancer | 2.99 (2.96 to 3.03) | Breast cancer | 2.04 (1.96 to 2.11) |
| **United Arab Emirates** | Prostate cancer | 2.45 (2.32 to 2.59) | Breast cancer | 3.43 (2.82 to 4.04) |
| **United Kingdom** | Tracheal, bronchus, and lung cancer | 5.56 (5.47 to 5.65) | Tracheal, bronchus, and lung cancer | 5.05 (4.92 to 5.18) |
| **United Republic of Tanzania** | Prostate cancer | 1.46 (1.45 to 1.47) | Cervical cancer | 1.94 (1.89 to 2) |
| **United States of America** | Tracheal, bronchus, and lung cancer | 4.92 (4.89 to 4.95) | Tracheal, bronchus, and lung cancer | 4.43 (4.39 to 4.47) |
| **United States Virgin Islands** | Prostate cancer | 5.75 (4.73 to 6.77) | Breast cancer | 3.56 (-1.84 to 8.96) |
| **Uruguay** | Tracheal, bronchus, and lung cancer | 6.58 (6.05 to 7.1) | Breast cancer | 4.55 (3.69 to 5.41) |
| **Uzbekistan** | Tracheal, bronchus, and lung cancer | 1.17 (1.15 to 1.19) | Breast cancer | 1.31 (1.22 to 1.4) |
| **Vanuatu** | Tracheal, bronchus, and lung cancer | 2.06 (1.82 to 2.29) | Breast cancer | 1.99 (1.22 to 2.75) |
| **Venezuela (Bolivarian Republic of)** | Prostate cancer | 2.74 (2.72 to 2.76) | Breast cancer | 2.38 (2.26 to 2.5) |
| **Viet Nam** | Tracheal, bronchus, and lung cancer | 3.65 (3.61 to 3.7) | Tracheal, bronchus, and lung cancer | 1.69 (1.66 to 1.73) |
| **Yemen** | Stomach cancer | 1.79 (1.76 to 1.81) | Stomach cancer | 1.55 (1.51 to 1.58) |
| **Zambia** | Prostate cancer | 1.46 (1.45 to 1.48) | Breast cancer | 2.08 (2.01 to 2.15) |
| **Zimbabwe** | Prostate cancer | 1.17 (1.16 to 1.17) | Cervical cancer | 2.5 (2.43 to 2.57) |

Table S14. Probability (%) of developing and dying from cancer from a cancer-free age to a specific age range for both sexes.

| **Start age** | **Probability (%) of developing cancer from a cancer-free age to a specific age range** | | | | | | | | | | | | |
| --- | --- | --- | --- | --- | --- | --- | --- | --- | --- | --- | --- | --- | --- |
|  | **40 years** | **45 years** | **50 years** | **55 years** | **60 years** | **65 years** | **70 years** | **75 years** | **80 years** | **85 years** | **90 years** | **95 years** | **Lifetime** |
| **Birth** | 1.01 (0.99 to 1.03) | 1.59 (1.57 to 1.62) | 2.44 (2.41 to 2.48) | 3.73 (3.69 to 3.77) | 5.54 (5.49 to 5.59) | 7.93 (7.88 to 7.99) | 10.84 (10.77 to 10.9) | 14.05 (13.98 to 14.12) | 17.03 (16.96 to 17.1) | 19.31 (19.24 to 19.39) | 20.89 (20.81 to 20.96) | 21.54 (21.46 to 21.61) | 21.7 (21.63 to 21.78) |
| **40 years** | / | 0.58 (0.57 to 0.59) | 1.43 (1.42 to 1.44) | 2.72 (2.7 to 2.74) | 4.53 (4.5 to 4.56) | 6.92 (6.89 to 6.96) | 9.83 (9.78 to 9.87) | 13.04 (12.99 to 13.09) | 16.02 (15.97 to 16.07) | 18.3 (18.25 to 18.36) | 19.88 (19.82 to 19.93) | 20.52 (20.47 to 20.58) | 20.69 (20.64 to 20.75) |
| **50 years** | / | / | / | 1.29(1.28 to 1.29) | 3.1 (3.08 to 3.12) | 5.49 (5.47 to 5.52) | 8.39 (8.36 to 8.42) | 11.61 (11.57 to 11.64) | 14.59 (14.55 to 14.63) | 16.87 (16.83 to 16.91) | 18.44 (18.4 to 18.49) | 19.09 (19.05 to 19.13) | 19.26 (19.22 to 19.3) |
| **60 years** | / | / | / | / | / | 2.39 (2.38 to 2.4) | 5.29 (5.28 to 5.31) | 8.51 (8.49 to 8.53) | 11.49 (11.47 to 11.51) | 13.77 (13.75 to 13.8) | 15.34 (15.32 to 15.37) | 15.99 (15.97 to 16.02) | 16.16 (16.14 to 16.19) |
| **70 years** | / | / | / | / | / | / | / | 3.21 (3.21 to 3.22) | 6.19 (6.19 to 6.2) | 8.48 (8.47 to 8.49) | 10.05 (10.04 to 10.06) | 10.7 (10.69 to 10.71) | 10.87 (10.85 to 10.88) |
| **80 years** | / | / | / | / | / | / | / | / | / | 2.28 (2.28 to 2.29) | 3.86 (3.85 to 3.86) | 4.51 (4.5 to 4.51) | (4.67 to 4.68) |
| **90 years** | / | / | / | / | / | / | / | / | / | / | / | 0.65 (0.65 to 0.65) | 0.82 (0.82 to 0.82) |
| **Start age** | **Probability (%) of dying from a cancer-free age to a specific age range** | | | | | | | | | | | | |
|  | **40 years** | **45 years** | **50 years** | **55 years** | **60 years** | **65 years** | **70 years** | **75 years** | **80 years** | **85 years** | **90 years** | **95 years** | **Lifetime** |
| **Birth** | 0.36 (0.35 to 0.36) | 0.57 (0.57 to 0.58) | 0.93 (0.92 to 0.94) | 1.54 (1.52 to 1.55) | 2.46 (2.45 to 2.48) | 3.75 (3.73 to 3.78) | 5.46 (5.44 to 5.49) | 7.53 (7.51 to 7.56) | 9.72 (9.69 to 9.75) | 11.71 (11.68 to 11.74) | 13.21 (13.17 to 13.24) | 13.99 (13.95 to 14.02) | 14.23 (14.19 to 14.26) |
| **40 years** | / | 0.22 (0.22 to 0.22) | 0.58 (0.57 to 0.58) | 1.18 (1.17 to 1.19) | 2.11 (2.09 to 2.12) | 3.4 (3.38 to 3.41) | 7.18 (7.16 to 7.2) | 9.36 (9.34 to 9.39) | 11.35 (11.32 to 11.38) | 12.85 (12.82 to 12.88) | 13.63 (13.6 to 13.66) | 13.87 (13.84 to 13.9) | 13.87 (13.84 to 13.9) |
| **50 years** | / | / | / | 0.6 (0.6 to 0.61) | 1.53 (1.52 to 1.54) | 2.82 (2.81 to 2.83) | 4.53 (4.51 to 4.54) | 6.6 (6.58 to 6.62) | 8.79 (8.77 to 8.81) | 10.77 (10.75 to 10.8) | 12.27 (12.25 to 12.3) | 13.05 (13.03 to 13.08) | 13.29 (13.27 to 13.32) |
| **60 years** | / | / | / | / | / | 1.29 (1.29 to 1.3) | 3 (2.99 to 3.01) | 5.07 (5.06 to 5.08) | 7.26 (7.24 to 7.27) | 9.24 (9.23 to 9.26) | 10.74 (10.73 to 10.76) | 11.52 (11.51 to 11.54) | 11.76 (11.75 to 11.78) |
| **70 years** | / | / | / | / | / | / | / | 2.07 (2.07 to 2.08) | 4.26 (4.25 to 4.26) | 6.24 (6.24 to 6.25) | 7.74 (7.74 to 7.75) | 8.52 (8.52 to 8.53) | 8.76 (8.76 to 8.77) |
| **80 years** | / | / | / | / | / | / | / | / | / | 1.99 (1.99 to 1.99) | 3.49 (3.48 to 3.49) | 4.27 (4.26 to 4.27) | 4.51 (4.5 to 4.51) |
| **90 years** | / | / | / | / | / | / | / | / | / | / | / | 0.78 (0.78 to 0.78) | 1.02 (1.02 to 1.02) |

Table S15. Probability (%) of developing and dying from cancer from a cancer-free age to a specific age range for males in 2021.

| **Start age** | **Probability (%) of developing cancer from a cancer-free age to a specific age range** | | | | | | | | | | | | |
| --- | --- | --- | --- | --- | --- | --- | --- | --- | --- | --- | --- | --- | --- |
|  | **40 years** | **45 years** | **50 years** | **55 years** | **60 years** | **65 years** | **70 years** | **75 years** | **80 years** | **85 years** | **90 years** | **95 years** | **Lifetime** |
| **Birth** | 0.82 (0.8 to 0.84) | 1.23 (1.21 to 1.25) | 1.89 (1.87 to 1.92) | 3.06 (3.03 to 3.1) | 4.89 (4.84 to 4.93) | 7.49 (7.44 to 7.55) | 10.77 (10.71 to 10.83) | 14.42 (14.35 to 14.48) | 17.68 (17.61 to 17.75) | 20.05 (19.98 to 20.12) | 21.55 (21.48 to 21.63) | 22.05 (21.98 to 22.13) | 22.15 (22.07 to 22.23) |
| **40 years** | / | 0.41 (0.4 to 0.41) | 1.07 (1.06 to 1.08) | 2.24 (2.22 to 2.26) | 4.06 (4.04 to 4.09) | 6.67 (6.63 to 6.71) | 9.95 (9.90 to 9.99) | 13.59 (13.54 to 13.64) | 16.86 (16.8 to 16.91) | 19.23 (19.17 to 19.29) | 20.73 (20.67 to 20.79) | 21.23 (21.17 to 21.29) | 21.33 (21.27 to 21.39) |
| **50 years** | / | / | / | 1.17 (1.16 to 1.17) | 2.99 (2.98 to 3.01) | 5.6 (5.57 to 5.62) | 8.88 (8.84 to 8.91) | 12.52 (12.48 to 12.56) | 15.79 (15.74 to 15.83) | 18.16 (18.11 to 18.2) | 19.66 (19.61 to 19.71) | 20.16 (20.11 to 20.21) | 20.26 (20.21 to 20.3) |
| **60 years** | / | / | / | / | / | 2.61 (2.6 to 2.61) | 5.88 (5.86 to 5.9) | 9.53 (9.5 to 9.55) | 12.79 (12.77 to 12.82) | 15.16 (15.13 to 15.19) | 16.66 (16.63 to 16.7) | 17.16 (17.13 to 17.2) | 17.26 (17.23 to 17.29) |
| **70 years** | / | / | / | / | / | / | / | 3.65 (3.64 to 3.65) | 6.91 (6.9 to 6.92) | 9.28 (9.27 to 9.29) | 10.78 (10.77 to 10.8) | 11.28 (11.27 to 11.3) | 11.38 (11.37 to 11.39) |
| **80 years** | / | / | / | / | / | / | / | / | / | 2.37 (2.37 to 2.37) | 3.87 (3.87 to 3.87) | 4.37 (4.37 to 4.37) | 4.47 (4.46 to 4.47) |
| **90 years** | / | / | / | / | / | / | / | / | / | / | / | 0.5 (0.5 to 0.5) | 0.6 (0.6 to 0.6) |
| **Start age** | **Probability (%) of dying from a cancer-free age to a specific age range** | | | | | | | | | | | | |
|  | **40 years** | **45 years** | **50 years** | **55 years** | **60 years** | **65 years** | **70 years** | **75 years** | **80 years** | **85 years** | **90 years** | **95 years** | **Lifetime** |
| **Birth** | 0.34 (0.34 to 0.35) | 0.54 (0.54 to 0.55) | 0.9 (0.89 to 0.91) | 1.54 (1.53 to 1.55) | 2.57 (2.55 to 2.59) | 4.05 (4.03 to 4.08) | 6.03 (6.01 to 6.06) | 8.4 (8.37 to 8.43) | 10.81 (10.78 to 10.85) | 12.91 (12.87 to 12.95) | 14.41 (14.37 to 14.45) | 15.05 (15.01 to 15.09) | 15.19 (15.16 to 15.23) |
| **40 years** | / | 0.2 (0.2 to 0.2) | 0.55 (0.55 to 0.56) | 1.2 (1.19 to 1.21) | 2.23 (2.21 to 2.24) | 3.71 (3.69 to 3.73) | 5.69 (5.67 to 5.71) | 8.06 (8.03 to 8.08) | 10.47 (10.44 to 10.5) | 12.57 (12.54 to 12.6) | 14.07 (14.04 to 14.1) | 14.71 (14.67 to 14.74) | 14.85 (14.82 to 14.88) |
| **50 years** | / | / | / | 0.64 (0.64 to 0.65) | 1.67 (1.66 to 1.68) | 3.16 (3.14 to 3.17) | 5.14 (5.12 to 5.16) | 7.5 (7.48 to 7.53) | 9.92 (9.89 to 9.94) | 12.01 (11.99 to 12.04) | 13.51 (13.49 to 13.54) | 14.15 (14.12 to 14.18) | 14.3 (14.27 to 14.33) |
| **60 years** | / | / | / | / | / | 1.48 (1.48 to 1.49) | 3.47 (3.46 to 3.48) | 5.83 (5.82 to 5.85) | 8.24 (8.23 to 8.26) | 10.34 (10.32 to 10.36) | 11.84 (11.82 to 11.86) | 12.48 (12.46 to 12.5) | 12.63 (12.61 to 12.65) |
| **70 years** | / | / | / | / | / | / | / | 2.37 (2.36 to 2.37) | 4.78 (4.77 to 4.79) | 6.88 (6.87 to 6.89) | 8.38 (8.37 to 8.39) | 9.01 (9 to 9.02) | 9.16 (9.15 to 9.17) |
| **80 years** | / | / | / | / | / | / | / | / | / | 2.1 (2.1 to 2.1) | 3.6 (3.59 to 3.6) | 4.24 (4.23 to 4.24) | 4.38 (4.38 to 4.38) |
| **90 years** | / | / | / | / | / | / | / | / | / | / | / | 0.64 (0.64 to 0.64) | 0.78 (0.78 to 0.78) |

Table S16. Probability (%) of developing and dying from cancer from a cancer-free age to a specific age range for females in 2021.

| **Start age** | **Probability (%) of developing cancer from a cancer-free age to a specific age range** | | | | | | | | | | | | |
| --- | --- | --- | --- | --- | --- | --- | --- | --- | --- | --- | --- | --- | --- |
|  | **40 years** | **45 years** | **50 years** | **55 years** | **60 years** | **65 years** | **70 years** | **75 years** | **80 years** | **85 years** | **90 years** | **95 years** | **Lifetime** |
| **Birth** | 1.21 (1.16 to 1.25) | 1.97 (1.9 to 2.03) | 3.01 (2.93 to 3.1) | 4.43 (4.33 to 4.52) | 6.23 (6.11 to 6.34) | 8.39 (8.27 to 8.52) | 10.89 (10.75 to 11.03) | 13.6 (13.46 to 13.75) | 16.21 (16.06 to 16.36) | 18.36 (18.21 to 18.51) | 19.98 (19.83 to 20.13) | 20.78 (20.63 to 20.94) | 21.03 (20.88 to 21.19) |
| **40 years** | / | 0.76 (0.74 to 0.78) | 1.81 (1.77 to 1.84) | 3.22 (3.16 to 3.27) | 5.02 (4.95 to 5.09) | 7.19 (7.1 to 7.27) | 9.68 (9.59 to 9.77) | 12.4 (12.3 to 12.5) | 15 (14.9 to 15.1) | 17.16 (17.05 to 17.26) | 18.77 (18.66 to 18.88) | 19.58 (19.47 to 19.68) | 19.82 (19.72 to 19.93) |
| **50 years** | / | / | / | 1.41 (1.39 to 1.43) | 3.21 (3.18 to 3.24) | 5.38 (5.33 to 5.43) | 7.88 (7.82 to 7.93) | 10.59 (10.52 to 10.65) | 13.19 (13.12 to 13.26) | 15.35 (15.28 to 15.42) | 16.97 (16.89 to 17.04) | 17.77 (17.7 to 17.84) | 18.02 (17.94 to 18.09) |
| **60 years** | / | / | / | / | / | 2.17 (2.16 to 2.18) | 4.66 (4.64 to 4.69) | 7.38 (7.35 to 7.41) | 9.98 (9.95 to 10.02) | 12.14 (12.1 to 12.18) | 13.75 (13.72 to 13.79) | 14.56 (14.52 to 14.6) | 14.81 (14.77 to 14.85) |
| **70 years** | / | / | / | / | / | / | / | 2.71 (2.71 to 2.72) | 5.32 (5.31 to 5.33) | 7.47 (7.46 to 7.49) | 9.09 (9.07 to 9.11) | 9.89 (9.88 to 9.91) | 10.14 (10.13 to 10.16) |
| **80 years** | / | / | / | / | / | / | / | / | / | 2.16 (2.15 to 2.16) | 3.77 (3.77 to 3.78) | 4.58 (4.57 to 4.58) | 4.82 (4.82 to 4.83) |
| **90 years** | / | / | / | / | / | / | / | / | / | / | / | 0.8 (0.8 to 0.8) | 1.05 (1.05 to 1.05) |
| **Start age** | **Probability (%) of dying from a cancer-free age to a specific age range** | | | | | | | | | | | | |
|  | **40 years** | **45 years** | **50 years** | **55 years** | **60 years** | **65 years** | **70 years** | **75 years** | **80 years** | **85 years** | **90 years** | **95 years** | **Lifetime** |
| **Birth** | 0.37 (0.36 to 0.38) | 0.61 (0.59 to 0.62) | 0.97 (0.95 to 0.99) | 1.53 (1.5 to 1.56) | 2.35 (2.31 to 2.39) | 3.44 (3.39 to 3.48) | 4.85 (4.8 to 4.89) | 6.58 (6.53 to 6.63) | 8.48 (8.42 to 8.54) | 10.32 (10.26 to 10.38) | 11.79 (11.73 to 11.85) | 12.71 (12.65 to 12.77) | 13.06 (13 to 13.12) |
| **40 years** | / | 0.24 (0.23 to 0.24) | 0.6 (0.59 to 0.61) | 1.16 (1.14 to 1.18) | 1.98 (1.95 to 2.01) | 3.07 (3.03 to 3.1) | 4.47 (4.44 to 4.51) | 6.21 (6.17 to 6.25) | 8.11 (8.06 to 8.15) | 9.95 (9.9 to 10) | 11.42 (11.37 to 11.47) | 12.34 (12.29 to 12.39) | 12.69 (12.64 to 12.74) |
| **50 years** | / | / | / | 0.56 (0.56 to 0.57) | 1.38 (1.37 to 1.39) | 2.47 (2.44 to 2.49) | 3.87 (3.85 to 3.9) | 5.61 (5.58 to 5.64) | 7.51 (7.47 to 7.54) | 9.35 (9.32 to 9.39) | 10.82 (10.79 to 10.86) | 11.74 (11.7 to 11.78) | 12.09 (12.05 to 12.13) |
| **60 years** | / | / | / | / | / | 1.09 (1.08 to 1.09) | 2.49 (2.48 to 2.51) | 4.23 (4.21 to 4.24) | 6.13 (6.11 to 6.15) | 7.97 (7.95 to 7.99) | 9.44 (9.42 to 9.46) | 10.36 (10.34 to 10.38) | 10.71 (10.69 to 10.73) |
| **70 years** | / | / | / | / | / | / | / | 1.73 (1.73 to 1.74) | 3.63 (3.63 to 3.64) | 5.48 (5.47 to 5.49) | 6.95 (6.94 to 6.96) | 7.87 (7.85 to 7.88) | 8.22 (8.21 to 8.23) |
| **80 years** | / | / | / | / | / | / | / | / | / | 1.84 (1.84 to 1.85) | 3.31 (3.31 to 3.32) | 4.23 (4.23 to 4.24) | 4.58 (4.58 to 4.59) |
| **90 years** | / | / | / | / | / | / | / | / | / | / | / | 0.92 (0.92 to 0.92) | 1.27 (1.27 to 1.27) |

Table S17. Lifetime incidence and death risk of specific cancer type by sex in 1990 and 2021.

| **Cancer type** | **Male** | | | | **Female** | | | |
| --- | --- | --- | --- | --- | --- | --- | --- | --- |
|  | **Lifetime incidence risk** | | **Lifetime death risk** | | **Lifetime incidence risk** | | **Lifetime death risk** | |
|  | **1990** | **2021** | **1990** | **2021** | **1990** | **2021** | **1990** | **2021** |
| **Neuroblastoma and other peripheral nervous cell tumors** | 0.01 (0.01 to 0.01) | 0.01 (0.01 to 0.01) | 0 (0 to 0) | 0.01 (0.01 to 0.01) | 0.01 (0.01 to 0.01) | 0.01 (0.01 to 0.01) | 0 (0 to 0) | 0 (0 to 0) |
| **Malignant neoplasm of bone and articular cartilage** | 0.09 (0.09 to 0.09) | 0.12 (0.11 to 0.12) | 0.08 (0.08 to 0.08) | 0.09 (0.09 to 0.09) | 0.07 (0.07 to 0.07) | 0.09 (0.09 to 0.09) | 0.06 (0.06 to 0.06) | 0.07 (0.07 to 0.07) |
| **Soft tissue and other extraosseous sarcomas** | 0.1 (0.1 to 0.1) | 0.12 (0.12 to 0.12) | 0.07 (0.07 to 0.07) | 0.07 (0.07 to 0.07) | 0.1 (0.1 to 0.1) | 0.12 (0.12 to 0.12) | 0.07 (0.07 to 0.07) | 0.07 (0.07 to 0.07) |
| **Eye cancer** | 0.03 (0.03 to 0.03) | 0.04 (0.04 to 0.04) | 0.01 (0.01 to 0.01) | 0.01 (0.01 to 0.01) | 0.03 (0.03 to 0.03) | 0.04 (0.04 to 0.04) | 0.01 (0.01 to 0.01) | 0.01 (0.01 to 0.01) |
| **Tracheal, bronchus, and lung cancer** | 4.53 (4.53 to 4.54) | 4.26 (4.25 to 4.26) | 4.45 (4.44 to 4.45) | 3.88 (3.88 to 3.89) | 1.75 (1.75 to 1.75) | 2.52 (2.51 to 2.52) | 1.69 (1.69 to 1.69) | 2.25 (2.25 to 2.26) |
| **Larynx cancer** | 0.56 (0.56 to 0.57) | 0.45 (0.45 to 0.45) | 0.4 (0.4 to 0.4) | 0.27 (0.27 to 0.27) | 0.09 (0.08 to 0.09) | 0.08 (0.08 to 0.08) | 0.06 (0.06 to 0.06) | 0.05 (0.05 to 0.05) |
| **Liver cancer** | 0.72 (0.72 to 0.72) | 0.91 (0.9 to 0.91) | 0.71 (0.71 to 0.72) | 0.83 (0.83 to 0.83) | 0.38 (0.38 to 0.38) | 0.5 (0.5 to 0.5) | 0.4 (0.39 to 0.4) | 0.5 (0.5 to 0.5) |
| **Stomach cancer** | 3.31 (3.3 to 3.31) | 2.3 (2.29 to 2.3) | 2.94 (2.93 to 2.94) | 1.79 (1.78 to 1.79) | 2.06 (2.06 to 2.07) | 1.29 (1.29 to 1.29) | 1.92 (1.91 to 1.92) | 1.12 (1.12 to 1.12) |
| **Esophageal cancer** | 1.29 (1.29 to 1.3) | 1.18 (1.18 to 1.18) | 1.33 (1.32 to 1.33) | 1.13 (1.13 to 1.13) | 0.63 (0.63 to 0.64) | 0.48 (0.48 to 0.48) | 0.67 (0.66 to 0.67) | 0.47 (0.47 to 0.47) |
| **Other malignant neoplasms** | 0.43 (0.42 to 0.43) | 0.53 (0.53 to 0.53) | 0.31 (0.31 to 0.31) | 0.29 (0.29 to 0.29) | 0.46 (0.46 to 0.46) | 0.6 (0.6 to 0.6) | 0.35 (0.34 to 0.35) | 0.34 (0.34 to 0.34) |
| **Leukemia** | 0.62 (0.62 to 0.62) | 0.63 (0.63 to 0.63) | 0.5 (0.5 to 0.5) | 0.46 (0.46 to 0.46) | 0.55 (0.55 to 0.55) | 0.54 (0.53 to 0.54) | 0.45 (0.45 to 0.45) | 0.4 (0.4 to 0.4) |
| **Multiple myeloma** | 0.17 (0.17 to 0.17) | 0.24 (0.24 to 0.24) | 0.15 (0.15 to 0.15) | 0.19 (0.19 to 0.19) | 0.17 (0.17 to 0.17) | 0.22 (0.22 to 0.22) | 0.15 (0.15 to 0.15) | 0.19 (0.19 to 0.19) |
| **Non-Hodgkin lymphoma** | 0.61 (0.61 to 0.61) | 0.88 (0.88 to 0.88) | 0.37 (0.37 to 0.37) | 0.4 (0.4 to 0.4) | 0.5 (0.5 to 0.5) | 0.71 (0.71 to 0.71) | 0.31 (0.31 to 0.31) | 0.36 (0.35 to 0.36) |
| **Hodgkin lymphoma** | 0.1 (0.1 to 0.1) | 0.08 (0.08 to 0.08) | 0.06 (0.06 to 0.06) | 0.04 (0.04 to 0.04) | 0.07 (0.07 to 0.07) | 0.06 (0.06 to 0.06) | 0.04 (0.04 to 0.04) | 0.03 (0.03 to 0.03) |
| **Mesothelioma** | 0.07 (0.07 to 0.07) | 0.07 (0.07 to 0.07) | 0.06 (0.06 to 0.06) | 0.07 (0.07 to 0.07) | 0.03 (0.03 to 0.03) | 0.03 (0.03 to 0.03) | 0.02 (0.02 to 0.02) | 0.03 (0.03 to 0.03) |
| **Thyroid cancer** | 0.11 (0.11 to 0.11) | 0.19 (0.19 to 0.19) | 0.04 (0.04 to 0.04) | 0.05 (0.05 to 0.05) | 0.29 (0.29 to 0.29) | 0.4 (0.4 to 0.4) | 0.09 (0.09 to 0.09) | 0.09 (0.09 to 0.09) |
| **Brain and central nervous system cancer** | 0.32 (0.32 to 0.32) | 0.42 (0.42 to 0.43) | 0.27 (0.27 to 0.27) | 0.34 (0.33 to 0.34) | 0.3 (0.3 to 0.3) | 0.42 (0.42 to 0.43) | 0.25 (0.25 to 0.25) | 0.31 (0.31 to 0.31) |
| **Bladder cancer** | 1.15 (1.15 to 1.15) | 1.23 (1.22 to 1.23) | 0.58 (0.58 to 0.58) | 0.53 (0.53 to 0.53) | 0.41 (0.41 to 0.41) | 0.42 (0.42 to 0.42) | 0.24 (0.24 to 0.24) | 0.22 (0.22 to 0.22) |
| **Kidney cancer** | 0.43 (0.43 to 0.43) | 0.64 (0.63 to 0.64) | 0.24 (0.24 to 0.24) | 0.29 (0.29 to 0.29) | 0.3 (0.3 to 0.3) | 0.39 (0.39 to 0.39) | 0.16 (0.16 to 0.16) | 0.18 (0.18 to 0.18) |
| **Ovarian cancer** | / | / | / | / | 0.79 (0.79 to 0.79) | 0.79 (0.79 to 0.79) | 0.57 (0.57 to 0.57) | 0.56 (0.55 to 0.56) |
| **Testicular cancer** | 0.11 (0.11 to 0.11) | 0.17 (0.17 to 0.17) | 0.03 (0.03 to 0.03) | 0.02 (0.02 to 0.02) | / | / | / | / |
| **Malignant skin melanoma** | 0.29 (0.29 to 0.3) | 0.44 (0.44 to 0.44) | 0.09 (0.09 to 0.09) | 0.1 (0.1 to 0.1) | 0.31 (0.31 to 0.31) | 0.4 (0.4 to 0.41) | 0.09 (0.09 to 0.09) | 0.09 (0.09 to 0.09) |
| **Pancreatic cancer** | 0.61 (0.61 to 0.61) | 0.77 (0.77 to 0.77) | 0.63 (0.63 to 0.63) | 0.78 (0.78 to 0.78) | 0.62 (0.62 to 0.62) | 0.81 (0.81 to 0.81) | 0.66 (0.66 to 0.66) | 0.83 (0.83 to 0.83) |
| **Gallbladder and biliary tract cancer** | 0.24 (0.24 to 0.24) | 0.3 (0.3 to 0.3) | 0.22 (0.22 to 0.22) | 0.23 (0.23 to 0.23) | 0.43 (0.43 to 0.43) | 0.4 (0.4 to 0.4) | 0.4 (0.4 to 0.4) | 0.33 (0.33 to 0.33) |
| **Other pharynx cancer** | 0.25 (0.25 to 0.25) | 0.34 (0.34 to 0.34) | 0.18 (0.18 to 0.18) | 0.21 (0.21 to 0.21) | 0.06 (0.06 to 0.06) | 0.09 (0.09 to 0.09) | 0.05 (0.05 to 0.05) | 0.05 (0.05 to 0.05) |
| **Nasopharynx cancer** | 0.21 (0.21 to 0.21) | 0.19 (0.19 to 0.19) | 0.19 (0.19 to 0.19) | 0.13 (0.13 to 0.13) | 0.11 (0.11 to 0.11) | 0.08 (0.08 to 0.08) | 0.1 (0.1 to 0.1) | 0.06 (0.06 to 0.06) |
| **Lip and oral cavity cancer** | 0.58 (0.58 to 0.58) | 0.69 (0.69 to 0.69) | 0.34 (0.34 to 0.34) | 0.36 (0.36 to 0.36) | 0.3 (0.3 to 0.3) | 0.43 (0.43 to 0.44) | 0.18 (0.18 to 0.18) | 0.22 (0.22 to 0.22) |
| **Colon and rectum cancer** | 2.62 (2.61 to 2.62) | 3.47 (3.46 to 3.47) | 1.69 (1.69 to 1.69) | 1.7 (1.7 to 1.71) | 2.74 (2.73 to 2.74) | 2.99 (2.98 to 2.99) | 1.84 (1.84 to 1.84) | 1.62 (1.62 to 1.62) |
| **Uterine cancer** | / | / | / | / | 1.03 (1.03 to 1.03) | 1.31 (1.31 to 1.31) | 0.33 (0.33 to 0.33) | 0.31 (0.31 to 0.31) |
| **Prostate cancer** | 3.2 (3.2 to 3.21) | 3.86 (3.86 to 3.87) | 1.54 (1.54 to 1.54) | 1.46 (1.46 to 1.46) | / | / | / | / |
| **Cervical cancer** | / | / | / | / | 1.74 (1.73 to 1.76) | 1.54 (1.53 to 1.55) | 1.04 (1.04 to 1.04) | 0.78 (0.78 to 0.79) |
| **Breast cancer** | 0.05 (0.05 to 0.05) | 0.1 (0.1 to 0.1) | 0.03 (0.03 to 0.03) | 0.04 (0.04 to 0.04) | 4.35 (4.32 to 4.37) | 5.41 (5.37 to 5.44) | 1.95 (1.94 to 1.95) | 1.94 (1.93 to 1.94) |

Table S18. Average annual percent change (AAPC) in lifetime risk of developing cancer by sex globally and by SDI quintile, 1990-2021, 1990-2019 and 2019-2021.

| **Location** | **Male** | | | **Female** | | | **Both sexes** | | |
| --- | --- | --- | --- | --- | --- | --- | --- | --- | --- |
|  | **1990-2021 AAPC** | **1990-2019 AAPC** | **2019-2021 AAPC** | **1990-2021 AAPC** | **1990-2019 AAPC** | **2019-2021 AAPC** | **1990-2021 AAPC** | **1990-2019 AAPC** | **2019-2021 AAPC** |
| **Global** | 0.32 (0.28 to 0.34) | 0.79 (0.73 to 0.85) | -6.27 (-6.88 to -5.83) | 0.46 (0.44 to 0.48) | 0.73 (0.7 to 0.77) | -3.31 (-3.69 to -3.05) | 0.41 (0.38 to 0.44) | 0.78 (0.73 to 0.83) | -4.73 (-5.18 to -4.4) |
| **High SDI** | 0.24 (0.18 to 0.29) | 0.51 (0.45 to 0.58) | -3.48 (-4.44 to -2.31) | 0.33 (0.29 to 0.36) | 0.52 (0.47 to 0.56) | -1.88 (-2.63 to -1.35) | 0.31 (0.27 to 0.35) | 0.53 (0.46 to 0.59) | -2.1 (-3.01 to -1.54) |
| **High-middle SDI** | 0.55 (0.53 to 0.58) | 0.86 (0.78 to 0.94) | -3.73 (-4.15 to -3.39) | 0.7 (0.68 to 0.71) | 0.91 (0.86 to 0.95) | -2.31 (-2.62 to -2.07) | 0.64 (0.62 to 0.67) | 0.9 (0.83 to 0.97) | -3.02 (-3.42 to -2.7) |
| **Middle SDI** | 0.71 (0.69 to 0.73) | 1.09 (1.02 to 1.16) | -4.64 (-5.04 to -4.25) | 0.79 (0.76 to 0.8) | 0.97 (0.89 to 1.04) | -1.77 (-2.12 to -1.49) | 0.77 (0.76 to 0.78) | 1.05 (1 to 1.1) | -3.2 (-3.41 to -2.98) |
| **Low-middle SDI** | 0.79 (0.74 to 0.81) | 1.46 (1.42 to 1.49) | -8.31 (-9.02 to -7.84) | 1.11 (1.08 to 1.13) | 1.42 (1.37 to 1.47) | -3.24 (-3.73 to -2.86) | 0.97 (0.94 to 1) | 1.45 (1.41 to 1.48) | -5.48 (-6.04 to -5.07) |
| **Low SDI** | 0.4 (0.22 to 0.55) | 1.33 (1.16 to 1.5) | -11.12 (-13.8 to -6.7) | 0.8 (0.75 to 0.84) | 1.19 (1.11 to 1.28) | -4.27 (-5 to -3.74) | 0.63 (0.52 to 0.72) | 1.25 (1.13 to 1.36) | -7.11 (-8.63 to -4.2) |

**Table S19. Average annual percent change (AAPC) in lifetime risk of dying from cancer by sex globally and by SDI quintile, 1990-2021, 1990-2019 and 2019-2021.**

| **Location** | **Male** | | | **Female** | | | **Both sexes** | | |
| --- | --- | --- | --- | --- | --- | --- | --- | --- | --- |
|  | **1990-2021 AAPC** | **1990-2019 AAPC** | **2019-2021 AAPC** | **1990-2021 AAPC** | **1990-2019 AAPC** | **2019-2021 AAPC** | **1990-2021 AAPC** | **1990-2019 AAPC** | **2019-2021 AAPC** |
| **Global** | -0.2 (-0.25 to -0.16) | 0.36 (0.3 to 0.43) | -8.11 (-8.88 to -7.7) | 0 (-0.02 to 0.02) | 0.35 (0.28 to 0.41) | -4.66 (-5.06 to -4.39) | -0.08 (-0.12 to -0.06) | 0.36 (0.29 to 0.42) | -6.24 (-6.74 to -5.92) |
| **High SDI** | -0.2 (-0.28 to -0.12) | 0.14 (0.12 to 0.16) | -4.81 (-6.14 to -3.19) | -0.02 (-0.07 to 0.03) | 0.22 (0.18 to 0.26) | -2.56 (-3.76 to -1.78) | -0.11 (-0.19 to -0.04) | 0.18 (0.15 to 0.2) | -4.03 (-5.2 to -2.62) |
| **High-middle SDI** | -0.1 (-0.13 to -0.08) | 0.25 (0.16 to 0.34) | -5.06 (-5.48 to -4.69) | 0 (-0.03 to 0.01) | 0.27 (0.19 to 0.35) | -3.72 (-4.09 to -3.48) | -0.04 (-0.07 to -0.02) | 0.26 (0.19 to 0.34) | -4.35 (-4.72 to -4.04) |
| **Middle SDI** | 0.03 (0 to 0.04) | 0.48 (0.38 to 0.58) | -6.25 (-6.63 to -5.93) | 0.01 (-0.01 to 0.02) | 0.27 (0.17 to 0.37) | -3.59 (-3.9 to -3.33) | 0.04 (0.03 to 0.06) | 0.4 (0.32 to 0.47) | -4.93 (-5.19 to -4.69) |
| **Low-middle SDI** | 0.41 (0.35 to 0.44) | 1.21 (1.17 to 1.25) | -10.38 (-11.29 to -9.82) | 0.71 (0.67 to 0.73) | 1.13 (1.09 to 1.17) | -5.19 (-5.67 to -4.81) | 0.57 (0.53 to 0.6) | 1.18 (1.14 to 1.21) | -7.66 (-8.29 to -7.23) |
| **Low SDI** | 0.17 (-0.01 to 0.33) | 1.26 (1.09 to 1.44) | -13.38 (-16.06 to -8.76) | 0.57 (0.52 to 0.62) | 1.11 (1.02 to 1.2) | -6.25 (-7.02 to -5.68) | 0.39 (0.27 to 0.48) | 1.17 (1.05 to 1.29) | -9.48 (-11.06 to -8.38) |

**Table S20. Average annual percent change (AAPC) in lifetime risk of developing cancer by GBD region and sex, 1990-2021, 1990-2019 and 2019-2021.**

| **Location** | **Male** | | | **Female** | | | **Both sexes** | | |
| --- | --- | --- | --- | --- | --- | --- | --- | --- | --- |
|  | **1990-2021 AAPC** | **1990-2019 AAPC** | **2019-2021 AAPC** | **1990-2021 AAPC** | **1990-2019 AAPC** | **2019-2021 AAPC** | **1990-2021 AAPC** | **1990-2019 AAPC** | **2019-2021 AAPC** |
| **Andean Latin America** | -0.21 (-0.55 to 0.08) | 1.51 (1.41 to 1.61) | -21.57 (-25.63 to -13.63) | 0.46 (0.33 to 0.59) | 1.36 (1.13 to 1.6) | -9.78 (-11.52 to -6.34) | 0.16 (-0.05 to 0.34) | 1.44 (1.26 to 1.62) | -15.37 (-18.13 to -9.66) |
| **Australasia** | 0.62 (0.56 to 0.7) | 0.73 (0.48 to 0.99) | -0.71 (-0.87 to -0.56) | 0.53 (0.49 to 0.58) | 0.62 (0.47 to 0.77) | -0.7 (-1.24 to -0.43) | 0.58 (0.53 to 0.63) | 0.71 (0.48 to 0.93) | -0.69 (-1.37 to -0.5) |
| **Caribbean** | 0.27 (-0.47 to 1.07) | 1.06 (0.77 to 1.36) | -11.37 (-21.06 to 0.43) | 0.78 (0.33 to 1.21) | 0.7 (0.18 to 1.22) | 0.78 (0.33 to 1.21) | 0.41 (-0.59 to 1.76) | 0.9 (0.51 to 1.29) | -7.85 (-20.5 to 1.54) |
| **Central Asia** | -1.19 (-1.3 to -1.1) | -0.64 (-0.88 to -0.41) | -8.94 (-10.44 to -7.32) | -0.56 (-0.63 to -0.5) | -0.26 (-0.46 to -0.06) | -4.71 (-5.72 to -2.97) | -0.85 (-0.93 to -0.77) | -0.42 (-0.7 to -0.14) | -6.64 (-7.91 to -4.55) |
| **Central Europe** | 0.8 (0.77 to 0.83) | 1.37 (1.29 to 1.44) | -7.01 (-7.52 to -6.59) | 0.87 (0.83 to 0.89) | 1.24 (1.13 to 1.35) | -4.22 (-4.76 to -3.78) | 0.83 (0.81 to 0.86) | 1.29 (1.21 to 1.37) | -5.5 (-5.87 to -5.16) |
| **Central Latin America** | 0.2 (-0.05 to 0.35) | 1.08 (0.94 to 1.23) | -11.75 (-15.07 to -7.3) | 0.11 (0.02 to 0.19) | 0.54 (0.41 to 0.68) | -5.55 (-7.15 to -3.34) | 0.18 (0.04 to 0.29) | 0.82 (0.65 to 0.99) | -7.75 (-9.82 to -4.68) |
| **Central Sub-Saharan Africa** | 0.2 (0.08 to 0.28) | 1.17 (1.01 to 1.33) | -12.89 (-14.36 to -11.88) | 0.75 (0.72 to 0.77) | 1.06 (0.98 to 1.15) | -3.69 (-4.19 to -3.24) | 0.46 (0.38 to 0.52) | 1.02 (0.84 to 1.2) | -7.73 (-8.82 to -6.96) |
| **East Asia** | 1.23 (1.21 to 1.25) | 1.25 (1.2 to 1.3) | 0.86 (0.76 to 0.94) | 1.25 (1.22 to 1.27) | 1.24 (1.14 to 1.34) | 1.11 (0.66 to 1.62) | 1.26 (1.24 to 1.28) | 1.28 (1.2 to 1.37) | 1.23 (1.1 to 1.54) |
| **Eastern Europe** | -0.31 (-0.41 to -0.21) | 0.31 (-0.2 to 0.83) | -9.39 (-10.76 to -8.15) | 0.32 (0.27 to 0.37) | 0.66 (0.44 to 0.89) | -4.6 (-5.32 to -4.04) | 0.08 (-0.01 to 0.14) | 0.56 (0.29 to 0.83) | -6.71 (-8.07 to -5.41) |
| **Eastern Sub-Saharan Africa** | 0.44 (0.04 to 0.79) | 1.48 (1.11 to 1.86) | -13.77 (-18.74 to -7) | 0.77 (0.65 to 0.88) | 1.39 (1.24 to 1.54) | -6.52 (-8.23 to -3.28) | 0.63 (0.34 to 0.89) | 1.58 (1.28 to 1.89) | -10.13 (-14.56 to -5.27) |
| **High-income Asia Pacific** | 0.64 (0.62 to 0.67) | 0.71 (0.62 to 0.81) | -0.41 (-0.57 to -0.28) | 0.86 (0.83 to 0.88) | 0.97 (0.88 to 1.05) | -0.36 (-0.59 to -0.16) | 0.75 (0.73 to 0.77) | 0.83 (0.75 to 0.92) | -0.29 (-0.42 to -0.16) |
| **High-income North America** | -0.38 (-0.48 to -0.31) | -0.05 (-0.12 to 0.02) | -4.69 (-6.26 to -3.2) | -0.19 (-0.24 to -0.14) | 0.01 (-0.08 to 0.1) | -2.36 (-3.32 to -1.75) | -0.28 (-0.36 to -0.21) | -0.02 (-0.08 to 0.05) | -3.72 (-5.03 to -2.44) |
| **North Africa and Middle East** | 0.66 (0.6 to 0.71) | 1.45 (1.34 to 1.56) | -9.42 (-10.38 to -8.71) | 1.59 (1.55 to 1.63) | 1.99 (1.88 to 2.09) | -3.8 (-4.47 to -3.22) | 1.05 (1 to 1.09) | 1.68 (1.6 to 1.75) | -6.76 (-7.6 to -6.13) |
| **Oceania** | -0.09 (-0.22 to 0.08) | 0.61 (0.55 to 0.68) | -10.25 (-12.3 to -7.22) | 0.1 (0.04 to 0.17) | 0.36 (0.24 to 0.47) | -3.66 (-4.58 to -2.03) | 0 (-0.1 to 0.11) | 0.48 (0.4 to 0.56) | -6.57 (-8.24 to -3.98) |
| **South Asia** | 0.83 (0.78 to 0.86) | 1.39 (1.32 to 1.46) | -6.92 (-7.62 to -6.43) | 1.13 (1.08 to 1.17) | 1.42 (1.32 to 1.51) | -2.88 (-3.63 to -2.28) | 1 (0.96 to 1.03) | 1.41 (1.33 to 1.49) | -4.7 (-5.31 to -4.25) |
| **Southeast Asia** | 0.91 (0.85 to 0.99) | 1.29 (1.21 to 1.37) | -4.56 (-5.59 to -2.98) | 1.07 (1 to 1.14) | 1.23 (1.14 to 1.32) | -1.27 (-2.43 to 0.11) | 0.99 (0.92 to 1.06) | 1.23 (1.14 to 1.32) | -3.41 (-4.57 to -1.91) |
| **Southern Latin America** | 0.22 (0.18 to 0.26) | 0.75 (0.65 to 0.86) | -7.08 (-7.69 to -6.64) | 0.18 (0.15 to 0.21) | 0.57 (0.46 to 0.68) | -5.11 (-5.61 to -4.65) | 0.2 (0.16 to 0.23) | 0.65 (0.56 to 0.74) | -6.12 (-6.62 to -5.73) |
| **Southern Sub-Saharan Africa** | -0.98 (-1.07 to -0.9) | 0.45 (0.24 to 0.67) | -19.65 (-20.89 to -18.63) | 0.15 (0.06 to 0.23) | 0.71 (0.44 to 0.99) | -8.9 (-10.05 to -7.8) | -0.38 (-0.46 to -0.3) | 0.64 (0.38 to 0.89) | -13.81 (-14.99 to -12.86) |
| **Tropical Latin America** | 0.48 (0.44 to 0.5) | 1.14 (1.04 to 1.23) | -8.88 (-9.34 to -8.43) | 0.56 (0.52 to 0.58) | 0.89 (0.8 to 0.97) | -3.82 (-4.39 to -3.4) | 0.51 (0.49 to 0.53) | 1 (0.92 to 1.09) | -6.18 (-6.47 to -5.9) |
| **Western Europe** | 0.49 (0.42 to 0.55) | 0.79 (0.7 to 0.87) | -3.61 (-4.77 to -2.26) | 0.5 (0.46 to 0.54) | 0.73 (0.67 to 0.8) | -2.21 (-2.93 to -1.56) | 0.53 (0.48 to 0.57) | 0.79 (0.72 to 0.86) | -2.29 (-3.36 to -1.59) |
| **Western Sub-Saharan Africa** | 0.59 (0.48 to 0.67) | 1.54 (1.46 to 1.63) | -11.67 (-13.24 to -10.47) | 1.11 (1.07 to 1.14) | 1.46 (1.36 to 1.56) | -3.41 (-4.05 to -2.89) | 0.96 (0.89 to 1.01) | 1.58 (1.48 to 1.69) | -7.09 (-8.17 to -6.23) |

**Table S21. Average annual percent change (AAPC) in lifetime risk of dying from cancer by GBD region and sex, 1990-2021, 1990-2019 and 2019-2021.**

| **Location** | **Male** | | | **Female** | | | **Both sexes** | | |
| --- | --- | --- | --- | --- | --- | --- | --- | --- | --- |
|  | **1990-2021 AAPC** | **1990-2019 AAPC** | **2019-2021 AAPC** | **1990-2021 AAPC** | **1990-2019 AAPC** | **2019-2021 AAPC** | **1990-2021 AAPC** | **1990-2019 AAPC** | **2019-2021 AAPC** |
| **Andean Latin America** | -1.43 (-1.84 to -1.07) | 0.77 (0.68 to 0.85) | -27.69 (-32.61 to -18.28) | -0.5 (-0.66 to -0.38) | 0.73 (0.51 to 0.95) | -14.7 (-16.79 to -12.85) | -0.94 (-1.22 to -0.73) | 0.76 (0.57 to 0.95) | -21.09 (-24.59 to -13.79) |
| **Australasia** | 0.25 (0.22 to 0.29) | 0.3 (0.14 to 0.46) | -0.31 (-0.38 to -0.24) | 0.08 (0.02 to 0.13) | 0.21 (0.14 to 0.27) | -1.39 (-2.32 to -0.26) | 0.18 (0.15 to 0.22) | 0.29 (0.13 to 0.44) | -0.93 (-1.45 to -0.19) |
| **Caribbean** | -0.42 (-1.23 to 0.07) | 0.55 (0.24 to 0.86) | -14.37 (-24.53 to -1.91) | 0.42 (-0.04 to 0.88) | 0.36 (-0.19 to 0.92) | 0.42 (-0.04 to 0.88) | -0.17 (-1.24 to 1.35) | 0.46 (0.05 to 0.87) | -10.34 (-23.6 to 0.91) |
| **Central Asia** | -1.53 (-1.65 to -1.43) | -0.93 (-1.24 to -0.61) | -10.04 (-11.66 to -8.31) | -1 (-1.06 to -0.94) | -0.61 (-0.76 to -0.46) | -6.17 (-7.18 to -5.21) | -1.25 (-1.33 to -1.18) | -0.77 (-1.02 to -0.51) | -7.81 (-8.99 to -6.63) |
| **Central Europe** | 0.16 (0.14 to 0.19) | 0.83 (0.73 to 0.93) | -8.96 (-9.42 to -8.59) | 0.35 (0.31 to 0.38) | 0.8 (0.69 to 0.92) | -6.09 (-6.66 to -5.66) | 0.26 (0.22 to 0.29) | 0.81 (0.73 to 0.89) | -7.3 (-7.95 to -6.8) |
| **Central Latin America** | -1.04 (-1.34 to -0.85) | 0.17 (-0.03 to 0.37) | -16.31 (-20.35 to -10.61) | -0.88 (-1.01 to -0.79) | -0.15 (-0.26 to -0.04) | -9.55 (-11.48 to -5.94) | -0.91 (-1.11 to -0.77) | 0.02 (-0.07 to 0.1) | -12.38 (-15.26 to -7.99) |
| **Central Sub-Saharan Africa** | -0.02 (-0.12 to 0.06) | 1.1 (0.93 to 1.27) | -14.93 (-16.23 to -13.93) | 0.45 (0.42 to 0.47) | 0.89 (0.81 to 0.97) | -5.67 (-6.14 to -5.26) | 0.19 (0.13 to 0.24) | 0.89 (0.7 to 1.07) | -9.31 (-10.34 to -8.52) |
| **East Asia** | 0.48 (0.46 to 0.5) | 0.51 (0.41 to 0.61) | 0.07 (0 to 0.12) | 0.35 (0.33 to 0.36) | 0.31 (0.18 to 0.43) | 0.91 (0.79 to 1.08) | 0.44 (0.43 to 0.46) | 0.46 (0.33 to 0.59) | 0.32 (0.24 to 0.5) |
| **Eastern Europe** | -1.19 (-1.29 to -1.1) | -0.48 (-0.84 to -0.13) | -11.23 (-12.58 to -9.93) | -0.54 (-0.59 to -0.5) | -0.07 (-0.28 to 0.14) | -7.05 (-7.79 to -6.43) | -0.8 (-0.86 to -0.76) | -0.23 (-0.49 to 0.04) | -8.9 (-9.63 to -8.18) |
| **Eastern Sub-Saharan Africa** | 0.18 (-0.23 to 0.53) | 1.44 (1.07 to 1.82) | -16.64 (-21.61 to -9.37) | 0.55 (0.41 to 0.68) | 1.41 (1.24 to 1.58) | -9.3 (-11.29 to -5.47) | 0.5 (0.06 to 0.74) | 1.58 (1.27 to 1.9) | -13.03 (-18.73 to -6.76) |
| **High-income Asia Pacific** | 0.36 (0.34 to 0.38) | 0.41 (0.29 to 0.53) | -0.43 (-0.68 to -0.28) | 0.49 (0.45 to 0.53) | 0.59 (0.45 to 0.74) | -0.6 (-1.4 to -0.11) | 0.42 (0.4 to 0.44) | 0.48 (0.4 to 0.56) | -0.32 (-0.49 to -0.2) |
| **High-income North America** | -0.91 (-1.05 to -0.84) | -0.39 (-0.49 to -0.29) | -7.2 (-9.39 to -4.97) | -0.55 (-0.63 to -0.5) | -0.21 (-0.27 to -0.15) | -3.7 (-5.19 to -2.77) | -0.73 (-0.85 to -0.67) | -0.3 (-0.36 to -0.24) | -5.9 (-7.72 to -4.02) |
| **North Africa and Middle East** | -0.18 (-0.27 to -0.13) | 0.77 (0.67 to 0.87) | -12.36 (-13.59 to -11.44) | 0.46 (0.41 to 0.49) | 0.99 (0.89 to 1.09) | -6.35 (-7.19 to -5.64) | 0.08 (0.01 to 0.13) | 0.85 (0.77 to 0.92) | -9.78 (-10.85 to -8.99) |
| **Oceania** | -0.25 (-0.4 to -0.05) | 0.54 (0.5 to 0.58) | -11.48 (-13.76 to -7.78) | 0.01 (-0.08 to 0.09) | 0.4 (0.29 to 0.5) | -5.12 (-6.46 to -3.15) | -0.11 (-0.19 to 0.03) | 0.45 (0.4 to 0.5) | -8.31 (-9.74 to -5.91) |
| **South Asia** | 0.5 (0.44 to 0.54) | 1.19 (1.1 to 1.28) | -8.95 (-9.79 to -8.36) | 0.77 (0.73 to 0.8) | 1.17 (1.07 to 1.26) | -4.76 (-5.33 to -4.3) | 0.63 (0.59 to 0.67) | 1.17 (1.09 to 1.26) | -6.72 (-7.33 to -6.26) |
| **Southeast Asia** | 0.48 (0.41 to 0.57) | 0.92 (0.84 to 1) | -5.99 (-7.12 to -4.38) | 0.61 (0.53 to 0.69) | 0.84 (0.74 to 0.94) | -2.72 (-4.01 to -1.12) | 0.55 (0.47 to 0.64) | 0.86 (0.76 to 0.96) | -3.95 (-5.1 to -1.98) |
| **Southern Latin America** | -0.34 (-0.4 to -0.29) | 0.3 (0.21 to 0.39) | -9.09 (-9.9 to -8.47) | -0.33 (-0.37 to -0.3) | 0.11 (0.04 to 0.18) | -6.47 (-7.07 to -5.94) | -0.34 (-0.39 to -0.31) | 0.2 (0.12 to 0.28) | -7.88 (-8.49 to -7.37) |
| **Southern Sub-Saharan Africa** | -1.46 (-1.58 to -1.37) | 0.21 (0.02 to 0.41) | -22.86 (-24.29 to -21.72) | -0.43 (-0.54 to -0.33) | 0.44 (0.16 to 0.73) | -12.93 (-14.53 to -11.72) | -0.95 (-1.03 to -0.88) | 0.32 (0.11 to 0.53) | -17.37 (-18.51 to -16.41) |
| **Tropical Latin America** | -0.04 (-0.07 to -0.01) | 0.79 (0.67 to 0.9) | -10.99 (-11.52 to -10.64) | 0.09 (0.05 to 0.12) | 0.55 (0.45 to 0.64) | -6.52 (-7.01 to -6.15) | 0 (-0.03 to 0.03) | 0.65 (0.56 to 0.74) | -8.59 (-9.04 to -8.24) |
| **Western Europe** | -0.13 (-0.23 to -0.07) | 0.26 (0.2 to 0.31) | -4.84 (-6.41 to -3.05) | -0.01 (-0.06 to 0.03) | 0.29 (0.25 to 0.34) | -2.57 (-3.64 to -1.75) | -0.04 (-0.09 to 0.01) | 0.27 (0.22 to 0.32) | -2.83 (-4.36 to -1.9) |
| **Western Sub-Saharan Africa** | 0.31 (0.19 to 0.38) | 1.4 (1.31 to 1.49) | -13.97 (-15.76 to -12.5) | 0.88 (0.82 to 0.91) | 1.34 (1.24 to 1.44) | -5.17 (-6.05 to -4.46) | 0.68 (0.59 to 0.73) | 1.43 (1.35 to 1.51) | -9.42 (-10.7 to -8.43) |

**Table S22. Average annual percent change (AAPC) in lifetime risk of developing cancer in 204 countries and territories by sex, 1990-2021, 1990-2019 and 2019-2021.**

| **Location** | **Male** | | | **Female** | | | **Both sexes** | | |
| --- | --- | --- | --- | --- | --- | --- | --- | --- | --- |
|  | **1990-2021 AAPC** | **1990-2019 AAPC** | **2019-2021 AAPC** | **1990-2021 AAPC** | **1990-2019 AAPC** | **2019-2021 AAPC** | **1990-2021 AAPC** | **1990-2019 AAPC** | **2019-2021 AAPC** |
| **Afghanistan** | -0.41 (-0.65 to -0.24) | 0.92 (0.69 to 1.16) | -17.26 (-20.33 to -11.86) | 0.8 (0.68 to 0.88) | 1.29 (0.97 to 1.6) | -5.82 (-7.52 to -2.97) | 0.35 (0.2 to 0.46) | 1.2 (0.89 to 1.52) | -10.29 (-12.35 to -6.66) |
| **Albania** | 0.13 (-0.01 to 0.24) | 0.76 (0.51 to 1.01) | -8.58 (-10.33 to -5.16) | 0.59 (0.5 to 0.68) | 1.02 (0.78 to 1.27) | -4.03 (-5.25 to -2.74) | 0.32 (0.21 to 0.42) | 0.91 (0.7 to 1.12) | -7.06 (-8.51 to -4.26) |
| **Algeria** | 0 (-0.13 to 0.08) | 0.92 (0.77 to 1.06) | -11.8 (-13.55 to -10.4) | 0.8 (0.76 to 0.84) | 1.17 (1.08 to 1.27) | -3.96 (-4.68 to -3.38) | 0.39 (0.31 to 0.45) | 1.02 (0.89 to 1.15) | -7.69 (-8.9 to -6.76) |
| **American Samoa** | 0.48 (0.41 to 0.55) | 0.49 (0.31 to 0.66) | 0.32 (0.14 to 0.45) | 0.81 (0.72 to 0.9) | 0.86 (0.62 to 1.09) | 0.39 (-1.1 to 1.55) | 0.62 (0.52 to 0.7) | 0.64 (0.47 to 0.8) | 0.35 (-0.33 to 0.64) |
| **Andorra** | -0.01 (-0.14 to 0.1) | 0.46 (0.28 to 0.64) | -5.08 (-7.57 to -3.51) | 0.17 (0.05 to 0.29) | 0.7 (0.59 to 0.81) | -5.55 (-8.46 to -3.76) | -0.04 (-0.21 to 0.07) | 0.5 (0.27 to 0.74) | -7.37 (-9.88 to -4.56) |
| **Angola** | 0.65 (0.44 to 0.8) | 1.82 (1.6 to 2.04) | -14.89 (-17.57 to -9.24) | 0.97 (0.91 to 1.03) | 1.37 (1.17 to 1.57) | -4.81 (-5.73 to -3.96) | 0.82 (0.7 to 0.91) | 1.51 (1.27 to 1.74) | -8.77 (-10.43 to -6.98) |
| **Antigua and Barbuda** | 0.64 (0.49 to 0.77) | 0.91 (0.2 to 1.62) | -1.22 (-1.93 to -0.66) | 0.84 (0.7 to 0.94) | 1.33 (0.91 to 1.75) | -5.59 (-7.67 to -2.38) | 0.79 (0.64 to 0.92) | 1.04 (0.55 to 1.53) | -0.72 (-1.65 to -0.09) |
| **Argentina** | 0.05 (0.01 to 0.09) | 0.56 (0.44 to 0.68) | -6.99 (-7.56 to -6.47) | 0.18 (0.13 to 0.21) | 0.59 (0.51 to 0.67) | -5.6 (-6.28 to -5.09) | 0.1 (0.06 to 0.13) | 0.55 (0.46 to 0.64) | -6.19 (-6.73 to -5.76) |
| **Armenia** | 0.06 (-0.42 to 0.51) | 0.64 (0.36 to 0.93) | -3.72 (-9.35 to -0.76) | 0.21 (0.02 to 0.4) | 0.5 (0.25 to 0.75) | -1.86 (-3.92 to -0.61) | 0.16 (-0.14 to 0.45) | 0.58 (0.38 to 0.78) | -2.79 (-5.97 to -0.93) |
| **Australia** | 0.61 (0.56 to 0.67) | 0.71 (0.43 to 0.99) | -0.52 (-0.89 to 0.52) | 0.55 (0.51 to 0.59) | 0.68 (0.53 to 0.84) | -0.73 (-1.31 to -0.49) | 0.58 (0.51 to 0.64) | 0.71 (0.51 to 0.91) | -0.84 (-1.83 to -0.61) |
| **Austria** | 0.33 (0.3 to 0.36) | 0.64 (0.56 to 0.72) | -4.28 (-4.79 to -3.82) | 0.16 (0.1 to 0.21) | 0.43 (0.25 to 0.61) | -3.2 (-4.06 to -2) | 0.27 (0.24 to 0.31) | 0.55 (0.45 to 0.65) | -3.65 (-4.18 to -3.19) |
| **Azerbaijan** | -0.64 (-0.85 to -0.5) | 0.2 (-0.02 to 0.43) | -12.11 (-15.28 to -7.99) | -0.23 (-0.33 to -0.13) | 0.24 (0.15 to 0.32) | -6.54 (-8.09 to -4.11) | -0.44 (-0.59 to -0.34) | 0.28 (0.08 to 0.48) | -9.31 (-11.7 to -5.95) |
| **Bahamas** | -0.05 (-0.23 to 0.16) | 0.72 (0.2 to 1.23) | -9.38 (-11.26 to -6.87) | 0.31 (0.22 to 0.4) | 0.57 (0.21 to 0.93) | -4.23 (-5.59 to -3.16) | 0.18 (0.1 to 0.27) | 0.66 (0.38 to 0.95) | -6.33 (-7.05 to -5.55) |
| **Bahrain** | 0.43 (0.35 to 0.47) | 1.13 (1.06 to 1.21) | -9.24 (-10.36 to -8.34) | 1.22 (1.17 to 1.26) | 1.49 (1.32 to 1.66) | -2.16 (-2.86 to -1.03) | 0.72 (0.66 to 0.75) | 1.24 (1.13 to 1.35) | -6.24 (-7.05 to -5.59) |
| **Bangladesh** | 0.58 (0.42 to 0.68) | 1 (0.93 to 1.07) | -4.99 (-7.52 to -1.88) | 1.02 (0.92 to 1.12) | 1.05 (0.93 to 1.16) | 1.02 (0.92 to 1.12) | 0.77 (0.56 to 1.09) | 1.01 (0.91 to 1.1) | -2.32 (-5.46 to 0.89) |
| **Barbados** | 0.75 (0.64 to 0.83) | 0.97 (0.57 to 1.36) | -3.14 (-4.71 to -1.03) | 0.79 (0.74 to 0.85) | 1.03 (0.81 to 1.26) | -2.88 (-3.62 to -1.68) | 0.77 (0.7 to 0.84) | 1.01 (0.66 to 1.36) | -2.72 (-3.72 to -1.29) |
| **Belarus** | 0.09 (-0.08 to 0.19) | 0.82 (0.48 to 1.16) | -8.65 (-10.95 to -4.8) | 0.63 (0.45 to 0.82) | 0.78 (-0.01 to 1.57) | 1.37 (0.64 to 1.85) | 0.44 (0.31 to 0.55) | 0.82 (0.29 to 1.35) | -5.2 (-6.83 to -2.63) |
| **Belgium** | -0.07 (-0.27 to 0.04) | 0.27 (0.15 to 0.39) | -2.35 (-4.48 to -1.17) | 0.11 (-0.01 to 0.23) | 0.34 (0.22 to 0.46) | -1.71 (-2.72 to -1.03) | -0.01 (-0.19 to 0.1) | 0.32 (0.25 to 0.4) | -2.59 (-5.2 to -1.13) |
| **Belize** | 0.54 (0.38 to 0.69) | 1.28 (0.7 to 1.86) | -8.47 (-10.56 to -5.35) | 0.68 (0.59 to 0.76) | 1.11 (0.67 to 1.54) | -4.91 (-6.34 to -3.11) | 0.7 (0.59 to 0.8) | 1.3 (0.88 to 1.71) | -6.33 (-7.78 to -4.03) |
| **Benin** | 0.31 (0.22 to 0.4) | 1.05 (0.96 to 1.13) | -10.16 (-11.1 to -8.8) | 0.66 (0.59 to 0.72) | 0.94 (0.8 to 1.07) | -2.99 (-4.12 to -1.59) | 0.49 (0.42 to 0.56) | 0.94 (0.83 to 1.06) | -6.32 (-7.08 to -5.25) |
| **Bermuda** | 0.91 (0.79 to 1.02) | 1.28 (0.75 to 1.81) | -4.71 (-6.17 to -2.46) | 0.33 (0.22 to 0.43) | 0.54 (0.16 to 0.93) | -3.21 (-4.86 to -1.45) | 0.73 (0.64 to 0.81) | 0.97 (0.57 to 1.37) | -3.84 (-5.22 to -1.94) |
| **Bhutan** | 1.25 (1.2 to 1.29) | 1.35 (1.24 to 1.46) | -0.3 (-1.05 to 0.6) | 1.38 (1.35 to 1.42) | 1.41 (1.26 to 1.55) | 1.25 (0.98 to 1.38) | 1.34 (1.3 to 1.38) | 1.41 (1.29 to 1.53) | 0.24 (-0.34 to 1.11) |
| **Bolivia (Plurinational State of)** | -0.98 (-1.41 to -0.73) | 1.3 (1.23 to 1.36) | -27.77 (-32.38 to -17.66) | 0.1 (-0.08 to 0.25) | 1.04 (1 to 1.09) | -12.29 (-14.55 to -7.6) | -0.34 (-0.62 to -0.06) | 1.16 (1.1 to 1.21) | -19.08 (-22.58 to -12.21) |
| **Bosnia and Herzegovina** | 0.99 (0.65 to 1.54) | 1.43 (0.1 to 2.78) | -0.09 (-1.3 to 0.87) | 1.03 (0.92 to 1.12) | 1.5 (1.37 to 1.63) | -5.63 (-7.18 to -3.25) | 0.89 (0.68 to 1.08) | 1.46 (1.09 to 1.83) | -6.26 (-9.33 to -2.51) |
| **Botswana** | -0.86 (-1.13 to -0.5) | 0.97 (0.75 to 1.19) | -23.72 (-26.91 to -18.54) | -0.33 (-0.56 to -0.08) | 0.38 (-0.16 to 0.93) | -11.82 (-15.01 to -7.31) | -0.7 (-1.08 to -0.42) | 0.69 (0.37 to 1.01) | -18 (-22.75 to -10.94) |
| **Brazil** | 0.48 (0.44 to 0.5) | 1.13 (1.03 to 1.23) | -8.78 (-9.28 to -8.32) | 0.56 (0.52 to 0.58) | 0.88 (0.8 to 0.96) | -3.74 (-4.31 to -3.31) | 0.51 (0.49 to 0.53) | 1 (0.91 to 1.08) | -6.1 (-6.39 to -5.82) |
| **Brunei Darussalam** | 0.16 (0.12 to 0.2) | 0.39 (0.2 to 0.58) | -3.28 (-3.97 to -2.44) | 0.37 (0.34 to 0.39) | 0.58 (0.49 to 0.67) | -2.88 (-3.24 to -2.55) | 0.24 (0.21 to 0.26) | 0.46 (0.37 to 0.55) | -3.11 (-3.55 to -2.68) |
| **Bulgaria** | 0.68 (0.6 to 0.76) | 1.44 (0.95 to 1.94) | -9.89 (-11.04 to -8.63) | 0.77 (0.66 to 0.87) | 1.2 (0.52 to 1.88) | -5.31 (-7.12 to -3.15) | 0.71 (0.62 to 0.79) | 1.29 (0.68 to 1.9) | -7.5 (-8.79 to -5.99) |
| **Burkina Faso** | 0.29 (0.16 to 0.39) | 1.17 (1.06 to 1.29) | -11.1 (-13.06 to -8.94) | 0.83 (0.74 to 0.92) | 1.26 (0.93 to 1.59) | -3.97 (-4.91 to -2.47) | 0.6 (0.51 to 0.69) | 1.2 (1.01 to 1.38) | -6.05 (-6.89 to -5.21) |
| **Burundi** | 0.46 (0.21 to 0.71) | 1.02 (0.03 to 2.01) | -9.5 (-12.39 to -4.93) | 0.4 (0.31 to 0.49) | 0.73 (0.49 to 0.98) | -3.86 (-5.27 to -1.83) | 0.25 (0.09 to 0.43) | 0.75 (0.16 to 1.35) | -6.69 (-8.95 to -3.16) |
| **Cabo Verde** | 1.82 (1.76 to 1.86) | 2.09 (1.85 to 2.33) | -1.93 (-2.75 to -0.62) | 0.79 (0.75 to 0.83) | 0.92 (0.79 to 1.06) | -1.25 (-1.93 to -0.33) | 1.31 (1.25 to 1.37) | 1.56 (1.45 to 1.66) | -1.48 (-2.33 to -0.16) |
| **Cambodia** | 1.19 (1.13 to 1.29) | 1.55 (1.47 to 1.63) | -3.9 (-4.91 to -1.99) | 1.45 (1.42 to 1.5) | 1.6 (1.55 to 1.66) | -1.01 (-1.54 to -0.17) | 1.29 (1.24 to 1.35) | 1.53 (1.46 to 1.61) | -2.29 (-3.05 to -1.01) |
| **Cameroon** | -0.1 (-0.19 to -0.02) | 0.89 (0.71 to 1.07) | -13.52 (-14.67 to -12.64) | 0.21 (0.15 to 0.28) | 0.58 (0.34 to 0.82) | -4.52 (-5.54 to -3.57) | 0.07 (-0.01 to 0.15) | 0.72 (0.52 to 0.93) | -9.09 (-10.19 to -8.13) |
| **Canada** | 0 (-0.1 to 0.09) | 0.35 (0.28 to 0.42) | -4.77 (-6.3 to -3.09) | 0.16 (0.1 to 0.21) | 0.44 (0.39 to 0.5) | -2.75 (-4.04 to -1.9) | 0.05 (-0.03 to 0.11) | 0.4 (0.33 to 0.47) | -4.22 (-5.57 to -2.62) |
| **Central African Republic** | -0.71 (-0.97 to -0.5) | 0.08 (-0.39 to 0.56) | -10.88 (-14.42 to -5.72) | -0.11 (-0.25 to -0.01) | 0.23 (-0.07 to 0.53) | -4.14 (-6.06 to -1.5) | -0.39 (-0.59 to -0.22) | 0.11 (-0.19 to 0.42) | -6.64 (-9.55 to -2.73) |
| **Chad** | 0.57 (0.34 to 0.76) | 1.32 (1.19 to 1.45) | -6.41 (-10.44 to -3.95) | 1.19 (1.1 to 1.27) | 1.5 (1.33 to 1.67) | -1.53 (-3.26 to -0.4) | 0.89 (0.72 to 1.01) | 1.37 (1.25 to 1.5) | -3.9 (-6.84 to -2.12) |
| **Chile** | 0.69 (0.57 to 0.77) | 1.4 (1.28 to 1.53) | -8.22 (-9.97 to -5.34) | 0.2 (0.12 to 0.28) | 0.59 (0.48 to 0.7) | -3.94 (-5.93 to -2.7) | 0.42 (0.32 to 0.51) | 0.97 (0.9 to 1.04) | -6.76 (-8.38 to -4.42) |
| **China** | 1.23 (1.2 to 1.25) | 1.26 (1.14 to 1.38) | 0.91 (0.79 to 1.01) | 1.25 (1.22 to 1.28) | 1.23 (1.11 to 1.35) | 1.2 (0.69 to 1.9) | 1.27 (1.24 to 1.29) | 1.28 (1.19 to 1.37) | 1.31 (1.18 to 1.64) |
| **Colombia** | 0.73 (0.61 to 0.82) | 1.17 (0.81 to 1.53) | -5.58 (-7.22 to -3.29) | 0.31 (0.25 to 0.37) | 0.62 (0.48 to 0.77) | -4.06 (-5.05 to -2.94) | 0.54 (0.47 to 0.6) | 0.9 (0.76 to 1.05) | -4.56 (-5.74 to -3.03) |
| **Comoros** | 0.67 (0.48 to 0.8) | 1.25 (1.17 to 1.33) | -7.51 (-10.2 to -3.78) | 0.68 (0.53 to 0.77) | 0.86 (0.68 to 1.04) | -0.5 (-2.47 to 0.73) | 0.67 (0.47 to 0.81) | 0.98 (0.9 to 1.05) | -3.87 (-6.79 to -0.22) |
| **Congo** | 0.17 (-0.53 to 0.63) | 1.16 (0.93 to 1.39) | -8.59 (-20.78 to -2.53) | 0.7 (0.57 to 0.78) | 1 (0.75 to 1.26) | -3.36 (-5.35 to -0.97) | 0.4 (0.12 to 0.63) | 0.95 (0.75 to 1.16) | -4.65 (-9.86 to -1.48) |
| **Cook Islands** | 1.39 (1.28 to 1.48) | 1.42 (1.27 to 1.58) | 0.79 (0.43 to 1.01) | 1.04 (0.96 to 1.12) | 1.06 (0.97 to 1.14) | 1.04 (0.96 to 1.12) | 1.25 (1.14 to 1.35) | 1.29 (1.13 to 1.45) | 0.8 (0.4 to 1.01) |
| **Costa Rica** | 0.69 (0.5 to 0.88) | 1.12 (0.92 to 1.33) | -5.43 (-8.5 to -1.43) | 0.66 (0.56 to 0.76) | 0.72 (0.63 to 0.82) | 0.66 (0.56 to 0.76) | 0.63 (0.42 to 0.75) | 1.02 (0.86 to 1.19) | -3.1 (-7.53 to -0.85) |
| **Coted'Ivoire** | 0.58 (0.37 to 0.73) | 1.35 (1.15 to 1.54) | -9.79 (-12.5 to -5.11) | 1.09 (0.99 to 1.19) | 1.37 (1.12 to 1.62) | -1.35 (-2.49 to -0.21) | 0.85 (0.7 to 0.98) | 1.35 (1.08 to 1.62) | -3.73 (-5.99 to -1.64) |
| **Croatia** | 0.95 (0.75 to 1.09) | 1.5 (1.38 to 1.63) | -2.84 (-5.81 to -0.99) | 0.99 (0.76 to 1.17) | 1.2 (-0.1 to 2.51) | -1.95 (-6.5 to 0.12) | 0.99 (0.79 to 1.13) | 1.43 (1.31 to 1.55) | -2.28 (-5.32 to -0.46) |
| **Cuba** | 0.57 (0.42 to 0.81) | 1.2 (1.09 to 1.32) | -10.43 (-12.77 to -6.1) | 0.54 (0.42 to 0.66) | 0.92 (0.67 to 1.17) | -5.38 (-6.89 to -2.98) | 0.6 (0.47 to 0.78) | 1.07 (0.97 to 1.16) | -7.89 (-9.89 to -4.48) |
| **Cyprus** | 1.49 (1.43 to 1.54) | 1.87 (1.78 to 1.96) | -3.76 (-4.5 to -2.66) | 1.42 (1.36 to 1.47) | 1.77 (1.54 to 2) | -3.41 (-4.39 to -2.3) | 1.46 (1.4 to 1.5) | 1.85 (1.75 to 1.95) | -3.49 (-4.43 to -2.34) |
| **Czechia** | 0.56 (0.52 to 0.58) | 1.01 (0.91 to 1.12) | -6.14 (-6.67 to -5.7) | 0.55 (0.48 to 0.61) | 0.83 (0.74 to 0.93) | -3.4 (-4.47 to -2.1) | 0.55 (0.51 to 0.58) | 0.92 (0.81 to 1.03) | -4.79 (-5.44 to -4.21) |
| **Democratic People's Republic of Korea** | 0.07 (-0.15 to 0.28) | 0.18 (-0.96 to 1.33) | 0.14 (-0.37 to 0.51) | 0.42 (0.22 to 0.61) | 0.49 (-0.2 to 1.19) | 0.37 (-0.12 to 0.74) | 0.26 (0.04 to 0.45) | 0.32 (-0.38 to 1.02) | 0.22 (-0.31 to 0.61) |
| **Democratic Republic of the Congo** | 0.1 (-0.04 to 0.21) | 1.05 (0.84 to 1.26) | -12.44 (-14.4 to -11.09) | 0.79 (0.75 to 0.82) | 1.07 (0.95 to 1.2) | -3.24 (-3.9 to -2.64) | 0.42 (0.35 to 0.47) | 0.94 (0.74 to 1.13) | -6.69 (-7.66 to -5.84) |
| **Denmark** | 1.14 (1.09 to 1.21) | 1.32 (1.13 to 1.52) | -0.76 (-1.16 to -0.45) | 0.66 (0.62 to 0.69) | 0.82 (0.64 to 1) | -1.47 (-2.02 to -1.07) | 0.92 (0.87 to 0.96) | 1.09 (0.9 to 1.27) | -1.52 (-2.4 to -0.99) |
| **Djibouti** | 0.14 (-0.05 to 0.26) | 1.03 (0.95 to 1.1) | -11.7 (-14.42 to -6.97) | 0.33 (0.22 to 0.41) | 0.64 (0.56 to 0.73) | -4.04 (-5.64 to -1.77) | 0.23 (0.09 to 0.32) | 0.82 (0.74 to 0.9) | -7.86 (-9.81 to -4.5) |
| **Dominica** | 0.12 (-0.06 to 0.37) | 0.67 (0.37 to 0.97) | -6.39 (-8.9 to -1.58) | 0.15 (-0.03 to 0.3) | 0.44 (0.02 to 0.87) | -4.11 (-6.9 to -0.44) | 0.14 (0 to 0.33) | 0.37 (0.09 to 0.65) | -4.16 (-6.35 to -0.57) |
| **Dominican Republic** | 0.63 (0.58 to 0.67) | 0.95 (0.8 to 1.1) | -4.03 (-4.67 to -3.43) | 0.82 (0.77 to 0.86) | 1.05 (0.93 to 1.17) | -1.72 (-2.51 to -1.26) | 0.74 (0.7 to 0.77) | 0.98 (0.85 to 1.11) | -2.1 (-2.56 to -1.73) |
| **Ecuador** | 0.13 (-0.28 to 0.47) | 1.23 (0.84 to 1.62) | -11.05 (-18.54 to -6.65) | 0.5 (0.29 to 0.66) | 1.15 (0.73 to 1.57) | -7.17 (-10.22 to -3.35) | 0.3 (-0.05 to 0.53) | 1.24 (0.84 to 1.64) | -11.69 (-16.6 to -6.23) |
| **Egypt** | 1.3 (1.24 to 1.36) | 2.03 (1.97 to 2.1) | -8.5 (-9.46 to -7.77) | 2.61 (2.55 to 2.65) | 2.93 (2.72 to 3.13) | -1.85 (-2.73 to -0.54) | 1.91 (1.87 to 1.94) | 2.45 (2.28 to 2.61) | -5.48 (-6.14 to -4.97) |
| **El Salvador** | 1.61 (1.48 to 1.71) | 2.33 (1.96 to 2.69) | -8.13 (-10.18 to -5.49) | 1.2 (1.07 to 1.33) | 1.5 (1.22 to 1.77) | -2.23 (-4.18 to 0.41) | 1.43 (1.34 to 1.52) | 1.87 (1.41 to 2.33) | -5.24 (-6.73 to -3.12) |
| **Equatorial Guinea** | 1.41 (1.11 to 1.63) | 2.7 (2.59 to 2.81) | -15.31 (-19.23 to -9.55) | 1.33 (1.21 to 1.41) | 1.8 (1.71 to 1.89) | -5.27 (-7.02 to -2.75) | 1.35 (1.15 to 1.48) | 2.18 (2.12 to 2.25) | -9.65 (-12.4 to -5.71) |
| **Eritrea** | 1.98 (1.28 to 2.7) | 2.71 (1.88 to 3.55) | -7.94 (-16.69 to 0.41) | 1.09 (0.94 to 1.19) | 1.4 (1.14 to 1.66) | -2.81 (-5.1 to 0.05) | 1.59 (1.14 to 2.06) | 2.07 (1.52 to 2.63) | -5.08 (-11.08 to 0.52) |
| **Estonia** | 1.45 (1.3 to 1.58) | 1.9 (1.65 to 2.14) | -5.12 (-7.16 to -2.63) | 0.8 (0.64 to 0.95) | 1.19 (1.02 to 1.36) | -4.24 (-6.54 to -1.02) | 1.2 (1.08 to 1.29) | 1.61 (1.29 to 1.93) | -4.61 (-6.2 to -2.32) |
| **Eswatini** | -1.25 (-1.38 to -1.15) | 0.34 (0.03 to 0.66) | -22.54 (-24.07 to -21.41) | -0.62 (-0.69 to -0.56) | 0.41 (0.15 to 0.67) | -14.6 (-15.55 to -13.79) | -0.93 (-1.03 to -0.85) | 0.42 (0.05 to 0.8) | -18.63 (-19.81 to -17.75) |
| **Ethiopia** | 0.95 (0.81 to 1.08) | 2.22 (2.01 to 2.44) | -15.59 (-17.31 to -14.44) | 0.92 (0.88 to 0.96) | 1.36 (1.2 to 1.53) | -5.29 (-6.01 to -4.81) | 0.9 (0.83 to 0.97) | 1.7 (1.43 to 1.97) | -9.97 (-10.94 to -9.19) |
| **Fiji** | 0.02 (-0.11 to 0.2) | 0.62 (0.49 to 0.75) | -8.57 (-10.31 to -5.19) | -0.01 (-0.07 to 0.06) | 0.22 (0.13 to 0.31) | -3.34 (-4.09 to -1.93) | -0.04 (-0.11 to 0.07) | 0.37 (0.31 to 0.42) | -5.42 (-6.52 to -3.3) |
| **Finland** | 1.11 (1.07 to 1.15) | 1.29 (1.05 to 1.53) | -1.08 (-1.71 to -0.53) | 0.91 (0.88 to 0.93) | 1.07 (0.92 to 1.22) | -1.29 (-1.56 to -0.98) | 1.04 (1 to 1.08) | 1.2 (1.05 to 1.35) | -1.06 (-1.53 to -0.56) |
| **France** | 0.5 (0.46 to 0.54) | 0.77 (0.66 to 0.88) | -2.55 (-3.04 to -1.87) | 0.82 (0.79 to 0.85) | 1.09 (0.97 to 1.2) | -2.91 (-3.45 to -2.37) | 0.64 (0.6 to 0.67) | 0.9 (0.8 to 1.01) | -2.59 (-3.01 to -1.99) |
| **Gabon** | 0.2 (0.07 to 0.29) | 1.08 (0.91 to 1.24) | -11.08 (-12.87 to -9.75) | 0.37 (0.33 to 0.41) | 0.7 (0.51 to 0.9) | -4.11 (-4.89 to -3.29) | 0.29 (0.21 to 0.34) | 0.87 (0.68 to 1.07) | -6.99 (-8.2 to -5.89) |
| **Gambia** | 0.16 (-0.04 to 0.29) | 1.03 (0.63 to 1.42) | -9.1 (-12.33 to -4.93) | 0.73 (0.51 to 0.93) | 1.12 (0.52 to 1.73) | -4.42 (-8.14 to -0.04) | 0.48 (0.33 to 0.62) | 1.07 (0.51 to 1.64) | -6.47 (-9.21 to -2.91) |
| **Georgia** | 0.88 (0.63 to 1.14) | 1.29 (0.63 to 1.95) | -2.78 (-4.95 to -0.95) | 0.7 (0.5 to 0.88) | 1.1 (-0.05 to 2.28) | -1.71 (-3.72 to -0.18) | 0.85 (0.62 to 1.06) | 1.18 (0.32 to 2.05) | -2.23 (-4.19 to -0.63) |
| **Germany** | 0.63 (0.59 to 0.67) | 0.88 (0.66 to 1.1) | -2.86 (-3.55 to -1.8) | 0.49 (0.44 to 0.53) | 0.71 (0.55 to 0.87) | -2.6 (-3.41 to -1.51) | 0.61 (0.56 to 0.65) | 0.84 (0.62 to 1.05) | -2.63 (-3.42 to -1.53) |
| **Ghana** | 0.14 (0.11 to 0.17) | 0.86 (0.72 to 1) | -9.74 (-10.24 to -9.32) | 0.75 (0.72 to 0.78) | 1.06 (0.94 to 1.18) | -3.58 (-4.09 to -3.2) | 0.52 (0.48 to 0.55) | 0.99 (0.94 to 1.05) | -6.12 (-6.71 to -5.7) |
| **Greece** | 0.21 (0.15 to 0.28) | 0.43 (0.26 to 0.59) | -2.6 (-3.56 to -1.15) | 0.42 (0.35 to 0.48) | 0.54 (0.35 to 0.73) | -0.51 (-1.41 to -0.08) | 0.32 (0.25 to 0.37) | 0.43 (0.12 to 0.74) | -0.73 (-1.59 to -0.28) |
| **Greenland** | 0.24 (0.18 to 0.35) | 0.4 (0.35 to 0.46) | -2.25 (-3.23 to -0.19) | -0.1 (-0.18 to -0.01) | -0.07 (-0.23 to 0.09) | -0.06 (-0.53 to 1.14) | 0.17 (0.1 to 0.23) | 0.24 (0.09 to 0.38) | -0.27 (-0.56 to -0.09) |
| **Grenada** | 0.67 (0.44 to 0.96) | 1.49 (1.21 to 1.78) | -11.83 (-15.02 to -6.2) | 0.63 (0.42 to 0.78) | 1.01 (0.49 to 1.53) | -5.06 (-7.99 to -1.37) | 0.72 (0.54 to 0.95) | 1.24 (0.93 to 1.55) | -6 (-8.61 to -1.24) |
| **Guam** | -0.23 (-0.84 to 0.7) | 0.49 (0.23 to 0.76) | -8.75 (-17.26 to 0.1) | -0.43 (-0.61 to -0.28) | 0.36 (-0.2 to 0.93) | -8.35 (-11.16 to -5.81) | -0.3 (-0.67 to -0.06) | 0.33 (-0.08 to 0.73) | -10.1 (-15.31 to -3.35) |
| **Guatemala** | 1.12 (0.97 to 1.26) | 2.34 (1.91 to 2.77) | -15.05 (-16.66 to -13.37) | 0.78 (0.62 to 0.98) | 1.39 (1.16 to 1.61) | -7.57 (-9.69 to -3.57) | 1.05 (0.9 to 1.22) | 1.88 (1.2 to 2.56) | -10.65 (-12.74 to -6.93) |
| **Guinea** | -0.11 (-0.32 to 0.02) | 0.94 (0.78 to 1.1) | -12.92 (-15.73 to -8.47) | 0.66 (0.59 to 0.72) | 1.05 (0.92 to 1.18) | -4 (-5.13 to -2.02) | 0.34 (0.21 to 0.43) | 1 (0.86 to 1.14) | -7.8 (-9.65 to -4.91) |
| **Guinea-Bissau** | -0.12 (-0.44 to 0.12) | 1 (0.67 to 1.34) | -9.79 (-15.34 to -6.08) | 0.88 (0.74 to 0.99) | 1.37 (1.21 to 1.53) | -3.7 (-6.11 to -1.89) | 0.4 (0.19 to 0.56) | 1.14 (0.92 to 1.37) | -6.21 (-10.21 to -3.61) |
| **Guyana** | 0.21 (0.02 to 0.36) | 1.16 (0.49 to 1.84) | -12.86 (-15.28 to -8.57) | 0.57 (0.34 to 0.75) | 1.04 (0.34 to 1.75) | -6.08 (-9.23 to -1.96) | 0.42 (0.27 to 0.57) | 1.09 (0.56 to 1.62) | -8.83 (-10.91 to -5.29) |
| **Haiti** | 0.73 (-0.65 to 2.12) | 1.19 (0.22 to 2.18) | 0.73 (-0.65 to 2.12) | 0.56 (-1.11 to 2.22) | 0.78 (-0.18 to 1.74) | 0.56 (-1.11 to 2.22) | 0.69 (-0.82 to 2.2) | 1 (0.07 to 1.94) | 0.69 (-0.82 to 2.2) |
| **Honduras** | 0.18 (-0.33 to 0.42) | 1.28 (1.11 to 1.45) | -14.48 (-21.08 to -6.48) | 0.99 (0.51 to 1.57) | 1.39 (1.19 to 1.59) | -5.62 (-12.51 to 1.36) | 0.69 (0.2 to 0.93) | 1.38 (1.21 to 1.56) | -6.15 (-15.37 to -1.49) |
| **Hungary** | 0.59 (0.53 to 0.64) | 1.1 (0.99 to 1.22) | -6.66 (-7.48 to -5.96) | 0.6 (0.53 to 0.65) | 0.9 (0.75 to 1.06) | -3.99 (-4.92 to -2.38) | 0.59 (0.5 to 0.65) | 0.98 (0.81 to 1.14) | -5.36 (-6.69 to -3.47) |
| **Iceland** | 0.75 (0.69 to 0.81) | 0.82 (0.52 to 1.12) | -0.01 (-0.12 to 0.09) | 0.44 (0.33 to 0.54) | 0.45 (0.2 to 0.7) | -0.59 (-1.16 to -0.19) | 0.58 (0.51 to 0.64) | 0.72 (0.43 to 1) | -0.69 (-1.63 to -0.33) |
| **India** | 0.99 (0.96 to 1.02) | 1.55 (1.48 to 1.62) | -6.65 (-7.09 to -6.3) | 1.16 (1.12 to 1.2) | 1.42 (1.29 to 1.56) | -2.63 (-3.33 to -2.07) | 1.09 (1.06 to 1.12) | 1.48 (1.36 to 1.61) | -4.71 (-5.23 to -4.28) |
| **Indonesia** | 1.1 (0.95 to 1.25) | 1.61 (1.47 to 1.75) | -6.07 (-8.06 to -3.16) | 1.15 (0.96 to 1.31) | 1.38 (1.21 to 1.55) | -1.92 (-4.65 to 0.96) | 1.13 (0.96 to 1.28) | 1.48 (1.32 to 1.65) | -3.66 (-6.03 to -1.13) |
| **Iran (Islamic Republic of)** | 0.79 (0.68 to 0.88) | 1.92 (1.84 to 2) | -15.01 (-16.11 to -13.93) | 1.47 (1.41 to 1.53) | 2.14 (2.01 to 2.28) | -7.61 (-8.49 to -6.76) | 1.11 (1.02 to 1.2) | 2.02 (1.82 to 2.22) | -11.81 (-13 to -10.95) |
| **Iraq** | 0.75 (0.5 to 0.95) | 1.44 (0.95 to 1.92) | -6.52 (-10.21 to -1.83) | 1.65 (1.53 to 1.74) | 1.95 (1.75 to 2.14) | -2.32 (-4.14 to -0.42) | 1.22 (1.04 to 1.36) | 1.67 (1.34 to 2.01) | -4.1 (-6.98 to -0.62) |
| **Ireland** | 0.75 (0.7 to 0.8) | 1.18 (1.09 to 1.27) | -5.2 (-5.92 to -4.53) | 0.46 (0.39 to 0.51) | 0.84 (0.56 to 1.11) | -4.75 (-5.74 to -3.47) | 0.61 (0.54 to 0.66) | 1.03 (0.79 to 1.27) | -5.3 (-6.26 to -3.59) |
| **Israel** | 0.78 (0.7 to 0.86) | 1.09 (0.8 to 1.37) | -3.61 (-4.73 to -1.9) | 0.47 (0.41 to 0.51) | 0.79 (0.63 to 0.96) | -4.1 (-4.84 to -2.74) | 0.6 (0.54 to 0.65) | 0.91 (0.76 to 1.07) | -3.85 (-4.69 to -2.56) |
| **Italy** | 0.35 (0.24 to 0.42) | 0.58 (0.5 to 0.65) | -2.6 (-4.73 to -1.58) | 0.56 (0.47 to 0.62) | 0.76 (0.64 to 0.87) | -2.04 (-3.77 to -1.14) | 0.45 (0.35 to 0.52) | 0.65 (0.56 to 0.74) | -2.28 (-4.14 to -1.35) |
| **Jamaica** | 0.77 (0.6 to 0.94) | 1.22 (0.33 to 2.11) | -5.96 (-8.24 to -2.77) | 0.82 (0.72 to 0.91) | 1.08 (0.67 to 1.49) | -2.89 (-4.35 to -0.87) | 1.1 (0.94 to 1.26) | 1.29 (0.49 to 2.09) | -0.57 (-1.15 to -0.2) |
| **Japan** | 0.55 (0.52 to 0.57) | 0.6 (0.45 to 0.76) | -0.4 (-0.63 to -0.25) | 0.83 (0.8 to 0.85) | 0.93 (0.82 to 1.03) | -0.27 (-0.54 to -0.06) | 0.68 (0.66 to 0.7) | 0.75 (0.65 to 0.85) | -0.25 (-0.42 to -0.11) |
| **Jordan** | 0.33 (0.25 to 0.38) | 1.18 (1.06 to 1.31) | -10.96 (-12.16 to -9.94) | 1.31 (1.25 to 1.38) | 1.7 (1.52 to 1.88) | -4.13 (-5.09 to -2.97) | 0.87 (0.82 to 0.91) | 1.49 (1.31 to 1.67) | -7.62 (-8.51 to -6.84) |
| **Kazakhstan** | -2.13 (-2.22 to -2.04) | -1.53 (-1.8 to -1.25) | -10.4 (-11.77 to -9) | -1.15 (-1.27 to -1.05) | -0.76 (-1.19 to -0.32) | -5.86 (-7.74 to -3.71) | -1.57 (-1.65 to -1.49) | -1.1 (-1.39 to -0.82) | -7.96 (-9.27 to -6.52) |
| **Kenya** | 0.5 (0.37 to 0.59) | 1.53 (1.32 to 1.75) | -12.33 (-14.12 to -10.94) | 0.93 (0.84 to 1) | 1.42 (1.23 to 1.61) | -5.67 (-7.03 to -4.17) | 0.72 (0.62 to 0.8) | 1.46 (1.27 to 1.65) | -8.91 (-10.43 to -7.71) |
| **Kiribati** | 0.73 (0.54 to 0.83) | 0.58 (0.43 to 0.73) | 2 (0.17 to 4.15) | 0.68 (0.64 to 0.7) | 0.66 (0.57 to 0.75) | 0.76 (0.33 to 1.27) | 0.66 (0.58 to 0.75) | 0.63 (0.54 to 0.72) | 0.45 (0.22 to 0.56) |
| **Kuwait** | 1.48 (1.03 to 1.97) | 1.85 (1.59 to 2.12) | -0.52 (-8.01 to 1.57) | 0.62 (0.31 to 0.86) | 0.92 (-0.71 to 2.58) | -2.69 (-5.15 to -1.2) | 1.24 (0.95 to 1.48) | 1.65 (0.59 to 2.72) | -1.51 (-5.01 to 0.17) |
| **Kyrgyzstan** | -1.22 (-1.47 to -1.02) | -0.69 (-1.04 to -0.33) | -6.18 (-9.83 to -3.37) | -0.43 (-0.61 to -0.26) | -0.18 (-0.74 to 0.37) | -2.26 (-5.34 to -0.31) | -0.79 (-0.97 to -0.62) | -0.37 (-1.02 to 0.28) | -3.96 (-7 to -1.89) |
| **Lao People's Democratic Republic** | 1.09 (1.01 to 1.19) | 1.4 (1.35 to 1.44) | -3.58 (-4.78 to -1.66) | 1.36 (1.33 to 1.4) | 1.53 (1.49 to 1.57) | -1.17 (-1.62 to -0.42) | 1.23 (1.18 to 1.3) | 1.44 (1.41 to 1.48) | -2.43 (-3.21 to -0.98) |
| **Latvia** | 0.81 (0.65 to 0.95) | 1.41 (0.89 to 1.93) | -7.86 (-9.95 to -4.93) | 0.85 (0.73 to 0.97) | 1.2 (0.92 to 1.47) | -4.2 (-5.88 to -2.04) | 0.86 (0.77 to 0.95) | 1.28 (1.06 to 1.51) | -5.69 (-6.87 to -4.06) |
| **Lebanon** | 1.21 (1.11 to 1.3) | 2.25 (2.08 to 2.43) | -12.61 (-13.66 to -11.56) | 1.27 (1.23 to 1.31) | 1.8 (1.66 to 1.95) | -5.8 (-6.42 to -5.29) | 1.25 (1.16 to 1.31) | 2 (1.83 to 2.17) | -9.3 (-10.5 to -8.32) |
| **Lesotho** | -1.52 (-1.89 to -1.19) | -0.05 (-0.87 to 0.78) | -18.14 (-22.93 to -11.21) | -0.24 (-0.48 to -0.05) | 0.53 (-0.14 to 1.21) | -10.52 (-13.75 to -5.83) | -0.87 (-1.12 to -0.67) | 0.11 (-0.57 to 0.8) | -13.94 (-17.04 to -8.51) |
| **Liberia** | 0.98 (0.34 to 1.58) | 2.01 (0.92 to 3.1) | -7.17 (-16.97 to 0.32) | 1.72 (1.42 to 1.91) | 2.02 (1.52 to 2.52) | 1 (-0.61 to 1.64) | 1.62 (1.35 to 1.82) | 1.84 (1 to 2.68) | 0.47 (-0.66 to 1.17) |
| **Libya** | 0.13 (-0.11 to 0.32) | 0.68 (0.43 to 0.93) | -7.12 (-10.82 to -2.52) | 0.79 (0.72 to 0.86) | 1.18 (0.7 to 1.67) | -4.01 (-5.02 to -2.58) | 0.35 (0.18 to 0.49) | 0.84 (0.32 to 1.37) | -5.6 (-7.94 to -2.77) |
| **Lithuania** | 0.91 (0.86 to 0.96) | 1.41 (1.27 to 1.55) | -6.05 (-6.84 to -5.42) | 0.61 (0.51 to 0.72) | 0.81 (0.74 to 0.87) | -3.12 (-4.41 to -1.18) | 0.81 (0.76 to 0.85) | 1.17 (1.02 to 1.32) | -4.21 (-5.02 to -3.61) |
| **Luxembourg** | 0.34 (0.24 to 0.44) | 0.7 (0.52 to 0.89) | -3.46 (-5.39 to -2.27) | 0.38 (0.28 to 0.45) | 0.7 (0.46 to 0.93) | -2.55 (-3.47 to -1.85) | 0.35 (0.23 to 0.45) | 0.69 (0.52 to 0.87) | -2.43 (-3.92 to -1.61) |
| **Madagascar** | -0.34 (-0.49 to -0.24) | 0.51 (0.42 to 0.6) | -11.77 (-14.09 to -7.41) | 0.14 (0.08 to 0.19) | 0.39 (0.25 to 0.54) | -3.01 (-3.98 to -1.53) | -0.03 (-0.12 to 0.02) | 0.45 (0.37 to 0.53) | -6.6 (-8.01 to -4.02) |
| **Malawi** | 1.03 (0.9 to 1.22) | 2.24 (2.1 to 2.39) | -15.87 (-17.66 to -13.23) | 1.27 (1.17 to 1.35) | 1.94 (1.74 to 2.14) | -8.52 (-9.8 to -7.22) | 1.13 (1.02 to 1.26) | 2.05 (1.83 to 2.26) | -11.65 (-13.13 to -9.66) |
| **Malaysia** | 0.55 (0.5 to 0.59) | 0.94 (0.86 to 1.02) | -5.13 (-5.85 to -4.51) | 0.95 (0.87 to 1.05) | 1.2 (1.06 to 1.33) | -1.91 (-3.18 to -0.15) | 0.73 (0.67 to 0.77) | 1.06 (0.96 to 1.17) | -3.8 (-4.64 to -3.02) |
| **Maldives** | 0.79 (0.7 to 0.86) | 1.16 (0.92 to 1.41) | -4.53 (-5.77 to -2.62) | 0.76 (0.69 to 0.82) | 0.99 (0.63 to 1.35) | -2.25 (-3.31 to -0.86) | 0.68 (0.6 to 0.75) | 1 (0.85 to 1.15) | -3.63 (-4.87 to -2.17) |
| **Mali** | -0.1 (-0.26 to 0.02) | 1.22 (1.04 to 1.41) | -17.11 (-19.21 to -15.17) | 0.58 (0.52 to 0.62) | 1.08 (0.93 to 1.23) | -6.01 (-6.89 to -5.21) | 0.31 (0.21 to 0.41) | 1.2 (0.94 to 1.46) | -11.47 (-12.94 to -10.24) |
| **Malta** | 0.59 (0.43 to 0.73) | 0.82 (0.54 to 1.09) | -2.3 (-4.88 to -0.68) | 0.5 (0.35 to 0.62) | 0.59 (0.28 to 0.89) | -0.45 (-1.73 to 0.16) | 0.6 (0.52 to 0.68) | 0.71 (0.38 to 1.04) | -0.44 (-0.89 to -0.1) |
| **Marshall Islands** | 0.84 (0.83 to 0.85) | 0.82 (0.79 to 0.86) | 0.98 (0.94 to 1.07) | 0.67 (0.66 to 0.69) | 0.68 (0.64 to 0.71) | 0.55 (0.49 to 0.59) | 0.78 (0.77 to 0.79) | 0.78 (0.75 to 0.81) | 0.79 (0.77 to 0.81) |
| **Mauritania** | 0.36 (0.1 to 0.51) | 0.94 (0.77 to 1.11) | -6.48 (-10.15 to -2.52) | 1.2 (1.12 to 1.29) | 1.27 (1.02 to 1.51) | 1.34 (1.28 to 1.49) | 0.81 (0.66 to 0.93) | 1.16 (0.96 to 1.36) | -2.72 (-4.91 to -0.09) |
| **Mauritius** | 1.11 (0.98 to 1.23) | 1.4 (0.97 to 1.83) | -3.3 (-5.08 to -1.25) | 0.92 (0.78 to 1.08) | 1 (0.08 to 1.93) | 1.84 (1.31 to 2.63) | 0.93 (0.83 to 1.02) | 1.16 (0.56 to 1.76) | -2.42 (-3.9 to -0.73) |
| **Mexico** | -0.17 (-0.55 to 0.08) | 0.9 (0.78 to 1.02) | -13.6 (-18.61 to -8.22) | -0.21 (-0.37 to -0.1) | 0.23 (0.17 to 0.29) | -5.96 (-8.53 to -3.43) | -0.15 (-0.39 to 0.02) | 0.58 (0.49 to 0.66) | -8.88 (-12.19 to -5.26) |
| **Micronesia (Federated States of)** | 0.79 (0.75 to 0.81) | 0.81 (0.74 to 0.89) | 0.5 (0.13 to 0.59) | 0.7 (0.66 to 0.73) | 0.71 (0.67 to 0.75) | 0.45 (0.26 to 0.57) | 0.73 (0.7 to 0.78) | 0.75 (0.71 to 0.79) | 0.46 (0.29 to 0.59) |
| **Monaco** | 0.59 (0.56 to 0.63) | 0.87 (0.85 to 0.89) | -3.49 (-3.89 to -2.86) | 0.97 (0.95 to 0.99) | 1.16 (1.14 to 1.19) | -1.9 (-2.24 to -1.49) | 0.78 (0.75 to 0.81) | 1.01 (0.99 to 1.04) | -2.78 (-3.11 to -2.36) |
| **Mongolia** | 0.41 (0.33 to 0.49) | 0.75 (0.65 to 0.84) | -3.8 (-4.95 to -1.82) | 0.83 (0.77 to 0.89) | 1.16 (1.07 to 1.26) | -4.05 (-5 to -2.89) | 0.64 (0.58 to 0.7) | 0.93 (0.8 to 1.05) | -4.16 (-4.89 to -3.21) |
| **Montenegro** | 0.04 (-0.06 to 0.13) | 0.68 (0.46 to 0.9) | -8.85 (-10.24 to -7.31) | 0.4 (0.35 to 0.45) | 0.89 (0.82 to 0.96) | -6.61 (-7.34 to -5.97) | 0.24 (0.15 to 0.33) | 0.77 (0.64 to 0.89) | -6.94 (-8.04 to -5.43) |
| **Morocco** | 0.92 (0.77 to 1.03) | 1.43 (1.37 to 1.5) | -3.7 (-6.69 to -1.85) | 1.72 (1.67 to 1.76) | 1.94 (1.85 to 2.02) | -0.31 (-1.29 to 0.41) | 1.28 (1.18 to 1.35) | 1.63 (1.59 to 1.68) | -1.7 (-3.75 to -0.44) |
| **Mozambique** | 0.33 (0.19 to 0.5) | 1.3 (1.2 to 1.39) | -13.37 (-15.39 to -10.93) | 0.59 (0.51 to 0.68) | 1.15 (1.01 to 1.28) | -7.86 (-9.18 to -6.66) | 0.47 (0.39 to 0.59) | 1.19 (1.09 to 1.3) | -10.3 (-11.59 to -8.63) |
| **Myanmar** | 1.02 (0.64 to 1.19) | 1.51 (1.43 to 1.58) | -5.84 (-11.69 to -0.21) | 0.87 (0.63 to 1.11) | 0.77 (0.53 to 1.02) | 0.87 (0.63 to 1.11) | 1.11 (0.89 to 1.32) | 1.05 (0.85 to 1.25) | 1.11 (0.89 to 1.32) |
| **Namibia** | 0.4 (0.2 to 0.6) | 1.6 (1.45 to 1.75) | -16.59 (-18.77 to -13.73) | 0.82 (0.69 to 0.95) | 1.42 (1.18 to 1.65) | -8.27 (-10.06 to -5.6) | 0.67 (0.5 to 0.84) | 1.52 (1.35 to 1.69) | -11.74 (-13.68 to -9.25) |
| **Nauru** | 0.4 (0.38 to 0.43) | 0.36 (0.22 to 0.5) | 1.52 (1.29 to 1.67) | 0.34 (0.32 to 0.34) | 0.27 (0.2 to 0.35) | 1.35 (1.25 to 1.41) | 0.41 (0.39 to 0.43) | 0.37 (0.26 to 0.48) | 1.37 (1.2 to 1.5) |
| **Nepal** | 0.81 (0.75 to 0.85) | 1.63 (1.53 to 1.74) | -10.42 (-11.17 to -9.89) | 0.91 (0.86 to 0.95) | 1.27 (1.2 to 1.33) | -4.25 (-4.93 to -3.77) | 0.9 (0.86 to 0.94) | 1.48 (1.41 to 1.55) | -7.13 (-7.72 to -6.69) |
| **Netherlands** | 0.33 (0.27 to 0.37) | 0.59 (0.49 to 0.7) | -2.32 (-3.06 to -1.76) | 0.38 (0.34 to 0.41) | 0.61 (0.49 to 0.74) | -1.94 (-2.32 to -1.58) | 0.38 (0.33 to 0.41) | 0.6 (0.49 to 0.72) | -1.87 (-2.32 to -1.48) |
| **New Zealand** | 0.74 (0.7 to 0.78) | 0.81 (0.44 to 1.17) | -0.07 (-0.4 to 0.15) | 0.39 (0.32 to 0.46) | 0.48 (0.21 to 0.75) | -0.83 (-1.47 to -0.36) | 0.62 (0.6 to 0.65) | 0.7 (0.48 to 0.92) | -0.33 (-0.52 to -0.16) |
| **Nicaragua** | -0.19 (-0.45 to -0.01) | 1.32 (1.15 to 1.48) | -18.94 (-22.41 to -12.28) | -0.12 (-0.32 to 0.02) | 0.7 (0.46 to 0.93) | -11.27 (-13.75 to -6.91) | -0.13 (-0.33 to 0.01) | 0.97 (0.81 to 1.12) | -14.55 (-17.12 to -9.47) |
| **Niger** | 0.59 (0.39 to 0.72) | 1.5 (1.33 to 1.67) | -12.06 (-14.77 to -7.3) | 1.23 (1.16 to 1.29) | 1.58 (1.33 to 1.83) | -2.47 (-3.72 to -1.51) | 0.95 (0.82 to 1.04) | 1.57 (1.41 to 1.74) | -7.95 (-9.89 to -4.58) |
| **Nigeria** | 0.97 (0.88 to 1.03) | 1.94 (1.77 to 2.11) | -11.45 (-12.89 to -10.33) | 1.49 (1.44 to 1.53) | 1.81 (1.69 to 1.94) | -2.63 (-3.47 to -1.39) | 1.47 (1.41 to 1.51) | 2.08 (1.93 to 2.23) | -6.44 (-7.38 to -5.64) |
| **Niue** | 0.78 (0.71 to 0.83) | 0.83 (0.74 to 0.91) | 0.39 (0.13 to 0.59) | 0.64 (0.59 to 0.71) | 0.64 (0.49 to 0.79) | 0.48 (0.29 to 0.58) | 0.68 (0.62 to 0.73) | 0.76 (0.71 to 0.82) | 0.47 (0.23 to 0.6) |
| **North Macedonia** | 0.09 (0.03 to 0.14) | 1.13 (1 to 1.25) | -14.27 (-15.22 to -13.54) | 0.57 (0.49 to 0.64) | 1.15 (0.98 to 1.33) | -7.54 (-8.68 to -6.53) | 0.31 (0.23 to 0.36) | 1.11 (0.96 to 1.26) | -10.92 (-12.15 to -10) |
| **Northern Mariana Islands** | 0.32 (0.25 to 0.42) | 0.6 (0.51 to 0.68) | -3.89 (-5.21 to -2.08) | 0.36 (0.24 to 0.45) | 0.53 (0.4 to 0.66) | -1.01 (-3.17 to 0.31) | 0.43 (0.39 to 0.47) | 0.65 (0.54 to 0.77) | -2.9 (-3.53 to -2.08) |
| **Norway** | 1.09 (1.06 to 1.12) | 1.25 (1.11 to 1.4) | -0.74 (-1.06 to -0.51) | 0.8 (0.76 to 0.85) | 1 (0.75 to 1.24) | -1.34 (-1.96 to -0.83) | 0.94 (0.9 to 0.97) | 1.12 (0.96 to 1.28) | -1.17 (-1.76 to -0.77) |
| **Oman** | 0.62 (0.55 to 0.68) | 1.69 (1.35 to 2.04) | -13.67 (-14.86 to -12.75) | 0.82 (0.73 to 0.9) | 1.5 (1.16 to 1.84) | -8.34 (-9.7 to -6.86) | 0.72 (0.64 to 0.77) | 1.63 (1.31 to 1.96) | -11.26 (-12.42 to -10.32) |
| **Pakistan** | 0.16 (-0.01 to 0.28) | 0.93 (0.86 to 1) | -10.2 (-12.56 to -6.18) | 1.02 (0.93 to 1.09) | 1.34 (1.25 to 1.43) | -3.23 (-4.67 to -1.35) | 0.6 (0.48 to 0.68) | 1.13 (1.05 to 1.2) | -6.65 (-8.35 to -3.75) |
| **Palau** | 0.34 (0.33 to 0.35) | 0.34 (0.32 to 0.37) | 0.4 (0.34 to 0.47) | 0.13 (0.11 to 0.14) | 0.13 (0.1 to 0.16) | 0.12 (0.1 to 0.16) | 0.2 (0.19 to 0.21) | 0.21 (0.18 to 0.23) | 0.16 (0.14 to 0.19) |
| **Palestine** | 0.55 (0.38 to 0.73) | 1.08 (0.39 to 1.78) | -6.92 (-9.29 to -3.35) | 1.16 (1.11 to 1.2) | 1.4 (1.21 to 1.58) | -2.1 (-2.96 to -0.91) | 0.8 (0.7 to 0.92) | 1.07 (0.65 to 1.48) | -4.05 (-5.57 to -1.84) |
| **Panama** | 0.95 (0.79 to 1.07) | 1.25 (0.98 to 1.53) | -2.97 (-6 to -1.18) | 0.74 (0.66 to 0.82) | 0.85 (0.57 to 1.13) | 0.74 (0.66 to 0.82) | 0.85 (0.73 to 0.95) | 1.11 (0.77 to 1.45) | -1.74 (-4.11 to -0.37) |
| **Papua New Guinea** | -0.13 (-0.31 to 0.08) | 0.71 (0.61 to 0.82) | -12.09 (-15 to -7.83) | 0.17 (0.09 to 0.26) | 0.4 (0.26 to 0.55) | -3.31 (-4.67 to -1.41) | 0.08 (-0.02 to 0.23) | 0.57 (0.45 to 0.69) | -7.19 (-8.71 to -4.56) |
| **Paraguay** | 0.69 (0.57 to 0.88) | 1.57 (1.5 to 1.65) | -11.16 (-12.85 to -7.96) | 0.74 (0.67 to 0.81) | 1.27 (1.16 to 1.39) | -6.85 (-8.07 to -5.81) | 0.76 (0.68 to 0.86) | 1.44 (1.37 to 1.52) | -9.32 (-10.56 to -7.71) |
| **Peru** | -0.09 (-0.32 to 0.11) | 1.62 (1.47 to 1.77) | -21.63 (-24.39 to -19.44) | 0.56 (0.4 to 0.7) | 1.56 (1.25 to 1.87) | -12.44 (-14.63 to -10.04) | 0.26 (0.09 to 0.4) | 1.63 (1.35 to 1.91) | -16.98 (-19.3 to -14.57) |
| **Philippines** | -0.08 (-0.25 to 0.15) | 0.53 (0.45 to 0.61) | -9.11 (-11.74 to -5.05) | 0.56 (0.51 to 0.65) | 0.86 (0.76 to 0.96) | -3.93 (-4.82 to -2.35) | 0.25 (0.15 to 0.4) | 0.69 (0.6 to 0.78) | -6.17 (-7.81 to -3.46) |
| **Poland** | 0.86 (0.79 to 0.91) | 1.42 (1.29 to 1.56) | -6.8 (-8 to -5.68) | 1 (0.94 to 1.04) | 1.41 (1.37 to 1.45) | -4.44 (-5.4 to -2.58) | 0.92 (0.86 to 0.97) | 1.41 (1.37 to 1.46) | -5.88 (-6.77 to -5.16) |
| **Portugal** | 1.27 (1.2 to 1.34) | 1.54 (1.33 to 1.75) | -2.23 (-3.63 to -1.31) | 0.68 (0.62 to 0.73) | 0.92 (0.76 to 1.08) | -2.04 (-3.24 to -1.27) | 1 (0.93 to 1.06) | 1.27 (1.02 to 1.53) | -2.03 (-3.34 to -1.21) |
| **Puerto Rico** | 0.78 (0.72 to 0.83) | 0.93 (0.8 to 1.07) | -1.12 (-1.41 to -0.83) | 0.78 (0.7 to 0.86) | 0.83 (0.67 to 0.98) | -0.32 (-0.74 to 0.01) | 0.72 (0.67 to 0.76) | 0.82 (0.73 to 0.92) | -0.76 (-0.99 to -0.53) |
| **Qatar** | 1.65 (1.52 to 1.76) | 1.88 (1.69 to 2.08) | -1.65 (-3.55 to 0.89) | 1.44 (1.33 to 1.51) | 1.8 (1.6 to 2) | -3.05 (-4.53 to -1.75) | 1.55 (1.47 to 1.64) | 1.79 (1.62 to 1.97) | -1.81 (-2.97 to -0.05) |
| **Republic of Korea** | 1.29 (1.26 to 1.32) | 1.41 (1.31 to 1.51) | -0.16 (-0.36 to 0) | 1.31 (1.26 to 1.35) | 1.45 (1.3 to 1.61) | -0.1 (-0.54 to 0.17) | 1.29 (1.26 to 1.32) | 1.41 (1.28 to 1.53) | -0.12 (-0.4 to 0.09) |
| **Republic of Moldova** | 0.32 (0.25 to 0.39) | 0.66 (0.42 to 0.91) | -4.66 (-5.83 to -3.47) | 0.31 (0.17 to 0.42) | 0.39 (-0.1 to 0.87) | -0.21 (-1.45 to 0.58) | 0.37 (0.28 to 0.44) | 0.58 (0.22 to 0.95) | -1.99 (-3.3 to -0.77) |
| **Romania** | 1.65 (1.6 to 1.69) | 2.22 (2.12 to 2.33) | -6.34 (-7.08 to -5.61) | 1.48 (1.4 to 1.56) | 1.8 (1.61 to 1.99) | -2.52 (-3.34 to -1.34) | 1.57 (1.51 to 1.62) | 2.01 (1.86 to 2.17) | -4.65 (-5.64 to -3.3) |
| **Russian Federation** | -0.28 (-0.42 to -0.16) | 0.45 (0.17 to 0.73) | -10.45 (-12.23 to -8.91) | 0.54 (0.47 to 0.61) | 0.97 (0.66 to 1.28) | -4.67 (-5.82 to -3.48) | 0.24 (0.15 to 0.31) | 0.77 (0.56 to 0.98) | -7.69 (-9.11 to -6.67) |
| **Rwanda** | -0.05 (-5 to 6.2) | -2.84 (-3.6 to -2.07) | 2.07 (-56.19 to 32.32) | 1.81 (0.03 to 3.59) | -1.2 (-2.14 to -0.25) | 1.81 (0.03 to 3.59) | -0.51 (-5.34 to 5.51) | -3.36 (-4.02 to -2.71) | 1.46 (-56.17 to 37.03) |
| **Saint Kitts and Nevis** | 1.33 (1.14 to 1.48) | 1.84 (1.67 to 2.01) | -6.48 (-9.18 to -2.93) | 0.31 (0.19 to 0.43) | 0.41 (0.01 to 0.82) | -0.83 (-2.26 to 0.19) | 0.84 (0.75 to 0.92) | 1.18 (0.84 to 1.52) | -4.27 (-5.55 to -2.57) |
| **Saint Lucia** | 0.5 (0.36 to 0.65) | 1.21 (0.87 to 1.55) | -9.92 (-11.99 to -6.69) | 0.11 (0.03 to 0.18) | 0.26 (0.02 to 0.5) | 0.11 (0.03 to 0.18) | 0.39 (0.27 to 0.5) | 0.89 (0.74 to 1.04) | -6.94 (-8.79 to -4.15) |
| **Saint Vincent and the Grenadines** | 0.71 (0.53 to 0.91) | 0.98 (0.33 to 1.63) | -3.29 (-5.93 to -0.21) | 0.23 (0.14 to 0.29) | 0.29 (-0.12 to 0.71) | -0.34 (-0.77 to -0.02) | 0.56 (0.45 to 0.65) | 0.75 (0.32 to 1.18) | -2.31 (-3.86 to -0.22) |
| **Samoa** | 0.34 (0.29 to 0.39) | 0.28 (0.19 to 0.38) | 0.44 (0.26 to 0.99) | 0.64 (0.54 to 0.74) | 0.67 (0.62 to 0.73) | 0.61 (-1.18 to 0.91) | 0.5 (0.42 to 0.59) | 0.49 (0.43 to 0.54) | 0.6 (0.35 to 1.5) |
| **San Marino** | -0.86 (-1.06 to -0.66) | 0.32 (0.27 to 0.36) | -11.55 (-16.82 to -8.06) | -0.82 (-1.03 to -0.64) | 0.2 (0.13 to 0.28) | -9.65 (-14.54 to -6.56) | -0.85 (-1.07 to -0.65) | 0.33 (0.27 to 0.39) | -11.45 (-16.85 to -7.88) |
| **Sao Tome and Principe** | 1 (0.93 to 1.05) | 1.53 (1.39 to 1.67) | -5.87 (-7.04 to -4.86) | 1.2 (1.11 to 1.28) | 1.42 (1.11 to 1.73) | -1.9 (-3.33 to -0.09) | 1.14 (1.07 to 1.2) | 1.51 (1.32 to 1.7) | -3.94 (-5.02 to -2.17) |
| **Saudi Arabia** | 1.53 (1.43 to 1.62) | 1.91 (1.83 to 1.98) | -1.57 (-3.58 to -0.36) | 2.39 (2.35 to 2.43) | 2.63 (2.52 to 2.74) | -0.4 (-0.79 to -0.02) | 1.9 (1.83 to 1.95) | 2.23 (2.15 to 2.32) | -1.58 (-2.45 to -0.53) |
| **Senegal** | 0.61 (0.48 to 0.7) | 1.62 (1.42 to 1.82) | -12.96 (-14.63 to -11.59) | 1.06 (0.85 to 1.21) | 1.54 (1.28 to 1.81) | -4.81 (-7.62 to -1.49) | 0.86 (0.69 to 0.99) | 1.56 (1.18 to 1.95) | -8.2 (-10.59 to -4.68) |
| **Serbia** | 0.77 (0.6 to 0.93) | 1.22 (1.06 to 1.39) | -6.41 (-8.9 to -3.57) | 0.85 (0.77 to 0.93) | 1.12 (0.88 to 1.35) | -1.91 (-3.52 to -0.91) | 0.81 (0.73 to 0.91) | 1.21 (0.99 to 1.43) | -4.69 (-6.07 to -2.62) |
| **Seychelles** | 0.86 (0.83 to 0.89) | 1.35 (1.29 to 1.41) | -6.17 (-6.68 to -5.65) | 0.49 (0.46 to 0.51) | 0.95 (0.86 to 1.05) | -5.95 (-6.36 to -5.66) | 0.65 (0.63 to 0.68) | 1.13 (1.07 to 1.2) | -6.05 (-6.47 to -5.72) |
| **Sierra Leone** | 0.5 (0.25 to 0.71) | 1.09 (0.84 to 1.34) | -7.5 (-10.96 to -2.59) | 1.44 (1.28 to 1.56) | 1.71 (1.53 to 1.89) | -0.93 (-4.17 to 0.85) | 0.92 (0.75 to 1.06) | 1.31 (1.12 to 1.51) | -4.63 (-6.94 to -1.13) |
| **Singapore** | 0.91 (0.78 to 1.01) | 1.15 (0.75 to 1.56) | -1.76 (-3.85 to -0.55) | 1.01 (0.89 to 1.14) | 1.21 (0.91 to 1.51) | -2.74 (-4.51 to -0.45) | 0.94 (0.84 to 1.02) | 1.14 (0.69 to 1.59) | -1.82 (-3.54 to -0.78) |
| **Slovakia** | 0.48 (0.42 to 0.54) | 1.08 (0.98 to 1.18) | -8.44 (-9.38 to -7.58) | 0.61 (0.54 to 0.65) | 1 (0.93 to 1.07) | -4.99 (-6.01 to -3.28) | 0.53 (0.49 to 0.57) | 0.98 (0.92 to 1.05) | -6.06 (-6.78 to -5.25) |
| **Slovenia** | 1.12 (1.04 to 1.19) | 1.63 (1.38 to 1.88) | -3.95 (-4.95 to -2.71) | 0.75 (0.66 to 0.82) | 1.27 (0.84 to 1.7) | -4.9 (-6.02 to -3.54) | 1 (0.9 to 1.08) | 1.44 (1.18 to 1.7) | -3.86 (-5.44 to -2.44) |
| **Solomon Islands** | 0.75 (0.71 to 0.79) | 0.77 (0.64 to 0.89) | 0.49 (0.2 to 0.71) | 0.99 (0.94 to 1.05) | 1.02 (0.82 to 1.22) | 0.85 (0.55 to 1.13) | 0.85 (0.8 to 0.9) | 0.87 (0.72 to 1.03) | 0.58 (0.24 to 0.84) |
| **Somalia** | -0.11 (-0.82 to 0.44) | 0.66 (-0.07 to 1.4) | -5.08 (-10.47 to -2.39) | 0.24 (-0.36 to 0.66) | 0.6 (0.27 to 0.92) | -2.4 (-7.07 to -0.3) | 0.12 (-0.52 to 0.59) | 0.65 (0.13 to 1.18) | -3.56 (-8.61 to -1.22) |
| **South Africa** | -0.91 (-1.11 to -0.77) | 0.51 (0.37 to 0.65) | -18.83 (-21.49 to -16.71) | 0.08 (-0.03 to 0.19) | 0.72 (0.38 to 1.07) | -8.53 (-10.34 to -5.94) | -0.38 (-0.5 to -0.29) | 0.58 (0.32 to 0.84) | -12.86 (-14.66 to -11.33) |
| **South Sudan** | -0.13 (-0.35 to 0.03) | 0.67 (0.42 to 0.92) | -11.48 (-14.69 to -6.14) | 0.31 (0.21 to 0.4) | 0.57 (0.36 to 0.77) | -3.27 (-4.65 to -1.33) | 0.16 (0.02 to 0.3) | 0.7 (0.47 to 0.93) | -7.18 (-9.23 to -3.91) |
| **Spain** | 0.59 (0.47 to 0.69) | 0.91 (0.82 to 1) | -2.75 (-5.12 to -1.44) | 0.49 (0.39 to 0.58) | 0.76 (0.64 to 0.88) | -2.69 (-4.4 to -1.7) | 0.57 (0.46 to 0.65) | 0.85 (0.77 to 0.94) | -2.63 (-4.7 to -1.54) |
| **Sri Lanka** | 0.9 (0.73 to 1.05) | 1.44 (0.83 to 2.05) | -2.8 (-5.18 to -1.16) | 0.5 (0.15 to 0.76) | 0.87 (0.66 to 1.08) | -2.66 (-8.82 to 0.74) | 0.66 (0.39 to 0.85) | 1.14 (0.86 to 1.41) | -3.52 (-8.94 to -0.74) |
| **Sudan** | 0.73 (0.54 to 0.85) | 1.63 (1.56 to 1.7) | -11.09 (-13.65 to -6.43) | 1.24 (1.17 to 1.3) | 1.61 (1.52 to 1.7) | -3.79 (-4.9 to -1.72) | 1.01 (0.87 to 1.09) | 1.66 (1.52 to 1.8) | -7.59 (-9.48 to -4.14) |
| **Suriname** | 0.42 (0.29 to 0.63) | 1.24 (1.05 to 1.44) | -11.45 (-13.55 to -7.87) | 0.27 (0.18 to 0.38) | 0.72 (0.46 to 0.99) | -6.7 (-7.94 to -4.96) | 0.32 (0.15 to 0.45) | 0.99 (0.78 to 1.2) | -9.9 (-12.2 to -6.13) |
| **Sweden** | 0.49 (0.42 to 0.54) | 0.83 (0.67 to 0.98) | -4.13 (-5.19 to -2.63) | 0.41 (0.35 to 0.48) | 0.63 (0.48 to 0.78) | -2.97 (-4.01 to -1.61) | 0.44 (0.37 to 0.49) | 0.73 (0.6 to 0.86) | -3.63 (-4.63 to -2.27) |
| **Switzerland** | 0.5 (0.4 to 0.58) | 0.69 (0.49 to 0.89) | -1.79 (-2.88 to -1.11) | 0.48 (0.39 to 0.57) | 0.8 (0.52 to 1.09) | -3.5 (-4.57 to -2.26) | 0.48 (0.39 to 0.58) | 0.73 (0.51 to 0.95) | -2.35 (-3.65 to -1.54) |
| **Syrian Arab Republic** | 0.8 (0.5 to 1.1) | 0.76 (0.31 to 1.22) | 2.38 (-3.09 to 10.96) | 1.26 (1.17 to 1.34) | 1.38 (0.98 to 1.78) | 0.02 (-1.46 to 1.96) | 1.02 (0.86 to 1.2) | 1.1 (0.62 to 1.58) | 0.21 (-2.85 to 5.11) |
| **Taiwan (Province of China)** | 1.88 (1.81 to 1.96) | 2.04 (1.77 to 2.31) | 0 (-0.21 to 0.19) | 1.57 (1.49 to 1.64) | 1.89 (1.71 to 2.07) | -1.37 (-2.54 to -0.71) | 1.77 (1.69 to 1.84) | 1.94 (1.7 to 2.19) | -0.15 (-0.36 to 0.04) |
| **Tajikistan** | -1.82 (-2.39 to -1.31) | -0.91 (-1.3 to -0.51) | -9.74 (-19.98 to -4.14) | -0.92 (-1.06 to -0.82) | -0.59 (-0.84 to -0.34) | -5.24 (-7.41 to -2.83) | -1.34 (-1.65 to -1.05) | -0.72 (-1.1 to -0.33) | -6.88 (-12.72 to -3.51) |
| **Thailand** | 0.75 (0.68 to 0.81) | 0.93 (0.77 to 1.09) | -1.62 (-2.72 to -0.39) | 0.85 (0.8 to 0.91) | 0.95 (0.77 to 1.13) | -0.68 (-1.53 to 0.69) | 0.82 (0.77 to 0.87) | 0.93 (0.82 to 1.05) | -1.06 (-1.9 to -0.18) |
| **Timor-Leste** | 1.19 (0.88 to 1.49) | 1.34 (1.09 to 1.6) | 1.19 (0.88 to 1.49) | 1.2 (1.09 to 1.28) | 1.33 (0.97 to 1.69) | 0.21 (-0.44 to 0.7) | 1.07 (0.58 to 1.78) | 1.42 (1.23 to 1.61) | -3.93 (-10.95 to 1.61) |
| **Togo** | 0.76 (0.71 to 0.81) | 1.42 (1.19 to 1.65) | -7.67 (-8.58 to -6.92) | 0.97 (0.91 to 1.03) | 1.15 (0.98 to 1.32) | -1.93 (-2.79 to -0.52) | 0.83 (0.78 to 0.87) | 1.27 (1.05 to 1.49) | -4.54 (-5.43 to -3.78) |
| **Tokelau** | 0.89 (0.85 to 0.91) | 1.02 (0.99 to 1.05) | -0.84 (-1.4 to -0.11) | 0.67 (0.65 to 0.67) | 0.74 (0.71 to 0.76) | -0.23 (-0.43 to 0.12) | 0.72 (0.7 to 0.74) | 0.81 (0.78 to 0.85) | -0.53 (-0.93 to 0.04) |
| **Tonga** | 0.56 (0.42 to 0.69) | 0.5 (0.24 to 0.76) | 0.68 (0.19 to 2.2) | 0.34 (0.32 to 0.38) | 0.33 (0.29 to 0.37) | 0.46 (0.36 to 0.59) | 0.42 (0.35 to 0.5) | 0.34 (0.15 to 0.52) | 0.49 (0.25 to 1.25) |
| **Trinidad and Tobago** | 0.4 (0.21 to 0.53) | 1.21 (0.79 to 1.63) | -11.69 (-14.28 to -7.13) | 0.46 (0.32 to 0.56) | 0.81 (0.39 to 1.22) | -4.66 (-6.57 to -2.35) | 0.49 (0.29 to 0.6) | 1.01 (0.62 to 1.39) | -7.81 (-10.59 to -4.31) |
| **Tunisia** | 0.36 (0.29 to 0.47) | 1.17 (1.12 to 1.23) | -10.48 (-11.56 to -8.99) | 1.03 (0.97 to 1.07) | 1.43 (1.34 to 1.52) | -4.77 (-5.39 to -4.09) | 0.61 (0.56 to 0.69) | 1.24 (1.18 to 1.3) | -7.64 (-8.47 to -6.58) |
| **Turkey** | 0.55 (0.44 to 0.62) | 1.1 (0.98 to 1.22) | -6.9 (-8.41 to -4.28) | 1.37 (1.31 to 1.43) | 1.65 (1.4 to 1.91) | -2.09 (-3.01 to -1.24) | 0.81 (0.74 to 0.87) | 1.25 (1.14 to 1.36) | -5 (-6.13 to -3.08) |
| **Turkmenistan** | -1.35 (-1.46 to -1.23) | -0.97 (-1.33 to -0.6) | -6.49 (-7.97 to -3.94) | -0.47 (-0.62 to -0.36) | -0.29 (-0.65 to 0.07) | -1.7 (-3.74 to -0.35) | -0.82 (-0.92 to -0.73) | -0.61 (-0.92 to -0.3) | -4.91 (-6.44 to -2.66) |
| **Tuvalu** | 1.22 (1.02 to 1.44) | 1.28 (1.09 to 1.47) | 0.76 (-0.6 to 1.65) | 0.99 (0.88 to 1.15) | 1.03 (0.86 to 1.2) | 0.73 (0.55 to 0.83) | 1 (0.85 to 1.15) | 0.97 (0.73 to 1.21) | 0.56 (-0.56 to 1.67) |
| **Uganda** | 1.67 (1.57 to 1.79) | 2.8 (2.65 to 2.95) | -14.12 (-15.49 to -12.5) | 2.04 (1.96 to 2.11) | 2.6 (2.32 to 2.88) | -6.87 (-8.06 to -5.64) | 1.87 (1.76 to 1.96) | 2.75 (2.54 to 2.97) | -10.46 (-11.98 to -9.1) |
| **Ukraine** | -0.93 (-1.04 to -0.84) | -0.48 (-0.9 to -0.06) | -6.7 (-8.15 to -4.33) | -0.74 (-0.88 to -0.62) | -0.45 (-1.02 to 0.13) | -4.02 (-6.18 to -0.81) | -0.76 (-0.86 to -0.68) | -0.43 (-0.86 to 0) | -5.94 (-7.6 to -3.69) |
| **United Arab Emirates** | 0.43 (0.29 to 0.54) | 1.43 (1.1 to 1.77) | -13.07 (-14.7 to -11.78) | 1.2 (1.06 to 1.32) | 1.5 (1.07 to 1.92) | -1.88 (-3.77 to -0.62) | 0.78 (0.61 to 0.92) | 1.51 (1.06 to 1.95) | -10.39 (-12.53 to -6.18) |
| **United Kingdom** | 0.32 (0.19 to 0.4) | 0.82 (0.69 to 0.94) | -6 (-7.96 to -3.8) | 0.3 (0.23 to 0.35) | 0.6 (0.5 to 0.7) | -2.99 (-4.4 to -2.03) | 0.35 (0.27 to 0.41) | 0.72 (0.65 to 0.8) | -3.62 (-5.2 to -2.49) |
| **United Republic of Tanzania** | 0.06 (0 to 0.11) | 0.94 (0.79 to 1.1) | -12.19 (-13.01 to -11.52) | 0.61 (0.56 to 0.65) | 1.01 (0.91 to 1.1) | -5.48 (-6.21 to -4.84) | 0.37 (0.33 to 0.4) | 0.98 (0.87 to 1.09) | -8.21 (-8.71 to -7.78) |
| **United States of America** | -0.41 (-0.52 to -0.34) | -0.09 (-0.16 to -0.01) | -4.71 (-6.26 to -3.18) | -0.22 (-0.28 to -0.17) | -0.03 (-0.12 to 0.05) | -2.22 (-3.28 to -1.57) | -0.31 (-0.39 to -0.24) | -0.06 (-0.12 to 0) | -3.67 (-4.98 to -2.36) |
| **United States Virgin Islands** | -0.09 (-0.15 to -0.03) | 0.4 (0.24 to 0.56) | -6.18 (-6.99 to -5.27) | -0.38 (-0.44 to -0.33) | 0.32 (0.17 to 0.46) | -9.29 (-9.99 to -8.63) | -0.15 (-0.21 to -0.09) | 0.41 (0.27 to 0.54) | -7.06 (-7.77 to -6.29) |
| **Uruguay** | 0.49 (0.43 to 0.57) | 0.75 (0.65 to 0.85) | -3.6 (-4.54 to -1.99) | 0.55 (0.51 to 0.59) | 0.78 (0.67 to 0.9) | -2.83 (-3.57 to -1.99) | 0.47 (0.43 to 0.5) | 0.73 (0.61 to 0.85) | -3.45 (-3.96 to -2.9) |
| **Uzbekistan** | -1.31 (-1.42 to -1.22) | -1.13 (-1.54 to -0.71) | -2.43 (-3.64 to -1.41) | -0.4 (-0.49 to -0.33) | -0.3 (-0.6 to 0) | -1.32 (-2.18 to -0.62) | -0.79 (-0.9 to -0.7) | -0.64 (-1.11 to -0.18) | -1.7 (-2.84 to -0.7) |
| **Vanuatu** | 0.33 (0.22 to 0.46) | 0.7 (0.57 to 0.84) | -6.05 (-7.45 to -3.52) | 0.55 (0.47 to 0.63) | 0.74 (0.45 to 1.03) | -2.28 (-3.51 to -0.74) | 0.45 (0.37 to 0.54) | 0.7 (0.52 to 0.89) | -3.77 (-4.94 to -2.01) |
| **Venezuela (Bolivarian Republic of)** | 0.06 (-0.16 to 0.27) | 1.05 (0.72 to 1.39) | -12.73 (-15.93 to -8.33) | 0.29 (0.02 to 0.47) | 0.77 (0.23 to 1.31) | -5.2 (-9.32 to -0.32) | 0.27 (-0.02 to 0.53) | 0.89 (0.5 to 1.27) | -6.73 (-12.32 to -3.99) |
| **Viet Nam** | 0.85 (0.82 to 0.89) | 0.95 (0.89 to 1.01) | -0.92 (-1.38 to -0.1) | 1.3 (1.26 to 1.34) | 1.39 (1.33 to 1.44) | -0.36 (-0.92 to 0.39) | 1.07 (1.04 to 1.11) | 1.15 (1.1 to 1.19) | -0.65 (-1.13 to 0.12) |
| **Yemen** | 0.15 (0.03 to 0.24) | 0.74 (0.46 to 1.02) | -8.13 (-9.96 to -5.8) | 1.01 (0.95 to 1.07) | 1.28 (1.1 to 1.46) | -2.9 (-3.92 to -1.54) | 0.61 (0.52 to 0.68) | 1.04 (0.83 to 1.26) | -5.52 (-6.93 to -3.8) |
| **Zambia** | 0.74 (0.62 to 0.86) | 2.18 (1.93 to 2.42) | -18.36 (-19.89 to -16.73) | 0.86 (0.77 to 0.93) | 1.54 (1.37 to 1.7) | -9.31 (-10.63 to -8.2) | 0.77 (0.67 to 0.87) | 1.8 (1.64 to 1.96) | -13.72 (-15.02 to -12.38) |
| **Zimbabwe** | -1.5 (-1.66 to -1.35) | -0.03 (-0.32 to 0.27) | -21.81 (-23.96 to -19.51) | 0.07 (-0.08 to 0.19) | 0.82 (0.6 to 1.04) | -10.1 (-12.24 to -6.58) | -0.57 (-0.67 to -0.46) | 0.57 (0.27 to 0.87) | -15.35 (-16.84 to -13.8) |

Table S23. Average annual percent change (AAPC) in lifetime risk of dying from cancer in 204 countries and territories by sex, 1990-2021 and 2019-2021.

| **Location** | **Male** | | | **Female** | | | **Both sexes** | | |
| --- | --- | --- | --- | --- | --- | --- | --- | --- | --- |
|  | **1990-2021 AAPC** | **1990-2019 AAPC** | **2019-2021 AAPC** | **1990-2021 AAPC** | **1990-2019 AAPC** | **2019-2021 AAPC** | **1990-2021 AAPC** | **1990-2019 AAPC** | **2019-2021 AAPC** |
| **Afghanistan** | -0.7 (-0.97 to -0.5) | 0.84 (0.6 to 1.07) | -19.82 (-23.32 to -13.73) | 0.42 (0.29 to 0.51) | 1.07 (0.74 to 1.39) | -8.23 (-10.17 to -4.7) | -0.02 (-0.2 to 0.11) | 0.97 (0.8 to 1.13) | -12.8 (-15.25 to -8.58) |
| **Albania** | -0.35 (-0.5 to -0.24) | 0.32 (-0.01 to 0.64) | -9.55 (-11.78 to -5.65) | -0.21 (-0.32 to -0.12) | 0.3 (0.15 to 0.46) | -7.16 (-8.72 to -4.39) | -0.36 (-0.46 to -0.27) | 0.26 (0.05 to 0.48) | -8.76 (-10.2 to -7.65) |
| **Algeria** | -0.75 (-0.9 to -0.64) | 0.4 (0.27 to 0.54) | -15.38 (-17.56 to -13.62) | -0.22 (-0.29 to -0.17) | 0.28 (0.18 to 0.37) | -7.31 (-8.36 to -6.45) | -0.51 (-0.63 to -0.43) | 0.31 (0.19 to 0.42) | -11.58 (-13.25 to -10.34) |
| **American Samoa** | 0.27 (0.17 to 0.36) | 0.33 (0.14 to 0.52) | -0.04 (-0.55 to 0.25) | 0.56 (0.45 to 0.64) | 0.58 (0.38 to 0.78) | 0.21 (-0.36 to 0.54) | 0.37 (0.26 to 0.45) | 0.39 (0.2 to 0.57) | 0.1 (-0.56 to 0.41) |
| **Andorra** | -0.7 (-0.9 to -0.57) | -0.06 (-0.16 to 0.04) | -6.78 (-9.53 to -4.36) | -0.44 (-0.61 to -0.36) | 0.13 (0.08 to 0.18) | -8.13 (-10.54 to -4.92) | -0.71 (-0.9 to -0.54) | -0.15 (-0.27 to -0.03) | -8.01 (-10.9 to -5.04) |
| **Angola** | 0.43 (0.21 to 0.59) | 1.76 (1.55 to 1.97) | -17.06 (-19.84 to -14.58) | 0.65 (0.58 to 0.72) | 1.21 (1.01 to 1.4) | -7.31 (-8.34 to -6.49) | 0.53 (0.4 to 0.63) | 1.39 (1.18 to 1.6) | -11.17 (-12.92 to -9.56) |
| **Antigua and Barbuda** | 0.01 (-0.19 to 0.19) | 0.5 (0.01 to 1) | -7.94 (-10.94 to -3.87) | 0.39 (0.22 to 0.51) | 0.9 (0.81 to 0.99) | -4.5 (-7.99 to -2.29) | 0.07 (-0.11 to 0.21) | 0.58 (0.04 to 1.11) | -6.21 (-8.69 to -3) |
| **Argentina** | -0.46 (-0.52 to -0.41) | 0.14 (-0.01 to 0.29) | -8.79 (-9.51 to -8.26) | -0.33 (-0.36 to -0.29) | 0.12 (0.02 to 0.23) | -6.66 (-7.29 to -6.11) | -0.41 (-0.46 to -0.36) | 0.15 (0.06 to 0.24) | -7.84 (-8.57 to -7.28) |
| **Armenia** | -0.3 (-0.82 to 0.18) | 0.31 (0 to 0.62) | -4.5 (-10.61 to -1.21) | -0.04 (-0.23 to 0.13) | 0.23 (0.02 to 0.44) | -1.67 (-3.39 to -0.7) | -0.17 (-0.51 to 0.16) | 0.32 (0.13 to 0.52) | -3.4 (-7.1 to -1.24) |
| **Australia** | 0.22 (0.18 to 0.26) | 0.3 (0.11 to 0.5) | -0.42 (-0.5 to -0.34) | 0.1 (0.03 to 0.16) | 0.24 (0.15 to 0.33) | -1.28 (-2.34 to -0.02) | 0.2 (0.17 to 0.24) | 0.29 (0.13 to 0.44) | -0.22 (-0.29 to -0.15) |
| **Austria** | -0.28 (-0.34 to -0.24) | 0.06 (-0.01 to 0.14) | -5.05 (-5.86 to -3.98) | -0.32 (-0.39 to -0.26) | -0.03 (-0.31 to 0.24) | -3.28 (-4.36 to -1.91) | -0.26 (-0.32 to -0.22) | 0.06 (-0.1 to 0.22) | -4.06 (-4.97 to -2.72) |
| **Azerbaijan** | -0.87 (-1.06 to -0.71) | -0.05 (-0.24 to 0.15) | -10.41 (-12.22 to -7.39) | -0.74 (-0.86 to -0.67) | -0.16 (-0.25 to -0.08) | -8.12 (-9.9 to -5.29) | -0.81 (-0.98 to -0.7) | -0.08 (-0.25 to 0.08) | -10.92 (-13.53 to -7.17) |
| **Bahamas** | -0.52 (-0.68 to -0.36) | 0.51 (0.12 to 0.9) | -12.21 (-14.17 to -10.28) | -0.07 (-0.2 to 0.04) | 0.32 (0.06 to 0.58) | -5.75 (-7.45 to -4.15) | -0.3 (-0.46 to -0.18) | 0.41 (0.14 to 0.68) | -10.18 (-11.73 to -7.28) |
| **Bahrain** | -0.65 (-0.74 to -0.61) | 0.16 (0.06 to 0.25) | -11.5 (-12.81 to -10.48) | -0.08 (-0.12 to -0.06) | 0.24 (0.13 to 0.35) | -4.66 (-5.24 to -4.2) | -0.48 (-0.53 to -0.44) | 0.12 (-0.01 to 0.24) | -8.63 (-9.46 to -7.92) |
| **Bangladesh** | 0.22 (0.02 to 0.32) | 0.8 (0.73 to 0.88) | -7.46 (-10.44 to -3.9) | 0.74 (0.45 to 0.94) | 0.73 (0.62 to 0.84) | 0.53 (-1.56 to 0.64) | 0.42 (0.23 to 0.62) | 0.73 (0.64 to 0.83) | -4.76 (-7.48 to -1.69) |
| **Barbados** | 0.19 (0.09 to 0.28) | 0.47 (0.22 to 0.73) | -3.86 (-5.29 to -2.03) | 0.4 (0.32 to 0.47) | 0.64 (0.34 to 0.95) | -3.3 (-4.39 to -1.76) | 0.33 (0.26 to 0.4) | 0.57 (0.36 to 0.78) | -3.26 (-4.22 to -1.83) |
| **Belarus** | -1.07 (-1.19 to -0.98) | -0.21 (-0.6 to 0.18) | -11.59 (-13.27 to -10.14) | -0.63 (-0.78 to -0.48) | -0.22 (-1.03 to 0.6) | -6.25 (-8.35 to -3.48) | -0.73 (-0.86 to -0.61) | -0.16 (-0.73 to 0.42) | -7.51 (-9.25 to -4.52) |
| **Belgium** | -0.63 (-0.88 to -0.49) | -0.24 (-0.37 to -0.1) | -3.11 (-5.8 to -1.66) | -0.31 (-0.45 to -0.16) | 0 (-0.28 to 0.27) | -2.09 (-3.32 to -1.17) | -0.5 (-0.68 to -0.38) | -0.14 (-0.37 to 0.08) | -2.33 (-3.88 to -1.36) |
| **Belize** | -0.08 (-0.24 to 0.06) | 0.76 (0.15 to 1.37) | -10.9 (-12.88 to -7.41) | 0.24 (0.13 to 0.35) | 0.72 (0.34 to 1.09) | -5.39 (-7 to -3.29) | 0.14 (0.02 to 0.25) | 0.77 (0.46 to 1.08) | -8.44 (-10 to -5.72) |
| **Benin** | 0.12 (0.05 to 0.22) | 1 (0.92 to 1.08) | -12.13 (-13.09 to -10.69) | 0.5 (0.44 to 0.56) | 0.86 (0.7 to 1.02) | -4.97 (-5.95 to -3.44) | 0.3 (0.23 to 0.37) | 0.9 (0.8 to 1) | -8.39 (-9.21 to -7.22) |
| **Bermuda** | -0.01 (-0.2 to 0.16) | 0.41 (-0.02 to 0.84) | -7.82 (-10.61 to -3.92) | -0.48 (-0.56 to -0.39) | -0.11 (-0.43 to 0.22) | -5.63 (-6.76 to -3.7) | -0.27 (-0.35 to -0.18) | 0.14 (-0.15 to 0.44) | -6.36 (-7.32 to -4.66) |
| **Bhutan** | 1.01 (0.95 to 1.06) | 1.17 (1.07 to 1.27) | -1.24 (-2.13 to -0.05) | 1.15 (1.12 to 1.18) | 1.24 (1.14 to 1.35) | -0.26 (-0.7 to 0.39) | 1.14 (1.11 to 1.18) | 1.26 (1.17 to 1.35) | -0.57 (-1.07 to 0.11) |
| **Bolivia**  **(Plurinational State of)** | -1.67 (-2.17 to -1.37) | 1.02 (0.98 to 1.05) | -32.44 (-37.59 to -21.03) | -0.44 (-0.66 to -0.23) | 0.79 (0.76 to 0.83) | -16.34 (-19.34 to -10.38) | -0.95 (-1.3 to -0.59) | 0.89 (0.85 to 0.93) | -23.75 (-27.98 to -15.46) |
| **Bosnia and Herzegovina** | 0.13 (-0.25 to 0.45) | 0.9 (-0.27 to 2.09) | -10.48 (-15.07 to -4.15) | 0.46 (0.36 to 0.54) | 0.98 (0.83 to 1.12) | -6.78 (-8.21 to -4.24) | 0.29 (-0.07 to 0.64) | 1 (0.11 to 1.88) | -7.44 (-12.73 to -2.05) |
| **Botswana** | -1.19 (-1.48 to -0.77) | 0.9 (0.7 to 1.1) | -27.01 (-30.34 to -21.48) | -0.69 (-0.93 to -0.41) | 0.34 (-0.06 to 0.74) | -15.91 (-19.06 to -10.88) | -1.05 (-1.43 to -0.72) | 0.6 (0.32 to 0.88) | -21.63 (-26.26 to -14.28) |
| **Brazil** | -0.03 (-0.07 to -0.01) | 0.78 (0.66 to 0.9) | -10.88 (-11.39 to -10.53) | 0.08 (0.05 to 0.11) | 0.54 (0.44 to 0.64) | -6.44 (-6.94 to -6.06) | 0 (-0.03 to 0.03) | 0.64 (0.55 to 0.73) | -8.49 (-8.96 to -8.14) |
| **Brunei Darussalam** | -0.08 (-0.13 to -0.04) | 0.14 (-0.01 to 0.29) | -2.69 (-3.19 to -1.95) | 0.05 (0.03 to 0.08) | 0.28 (0.21 to 0.35) | -3.33 (-3.73 to -2.97) | -0.07 (-0.1 to -0.04) | 0.16 (0.07 to 0.26) | -3.6 (-4.05 to -3.13) |
| **Bulgaria** | -0.01 (-0.12 to 0.08) | 0.89 (0.32 to 1.47) | -12.42 (-13.84 to -11.01) | 0.17 (0.06 to 0.27) | 0.79 (0.05 to 1.52) | -8.25 (-9.85 to -6.17) | 0.09 (-0.03 to 0.19) | 0.84 (0.26 to 1.43) | -10.31 (-12.03 to -8.63) |
| **Burkina Faso** | 0.19 (0.04 to 0.29) | 1.13 (0.98 to 1.28) | -12.57 (-14.94 to -9.2) | 0.73 (0.61 to 0.84) | 1.25 (0.87 to 1.63) | -4.95 (-6.17 to -3.18) | 0.42 (0.3 to 0.51) | 1.19 (1.01 to 1.38) | -9.6 (-11.45 to -7.41) |
| **Burundi** | 0.4 (0.13 to 0.65) | 1.05 (0.06 to 2.06) | -10.97 (-13.96 to -6.08) | 0.37 (0.28 to 0.46) | 0.77 (0.51 to 1.04) | -4.83 (-6.18 to -2.79) | 0.22 (0.05 to 0.4) | 0.82 (0.3 to 1.34) | -8.05 (-10.43 to -4.28) |
| **Cabo Verde** | 1.31 (1.24 to 1.37) | 1.63 (1.51 to 1.76) | -3.19 (-4.11 to -1.47) | 0.51 (0.46 to 0.55) | 0.72 (0.58 to 0.86) | -1.54 (-2.01 to -0.81) | 0.95 (0.88 to 1) | 1.25 (1.04 to 1.46) | -3.19 (-4.27 to -2.06) |
| **Cambodia** | 0.96 (0.88 to 1.08) | 1.36 (1.29 to 1.43) | -4.75 (-5.89 to -2.59) | 1.03 (0.99 to 1.09) | 1.25 (1.2 to 1.31) | -2.07 (-2.79 to -0.89) | 0.98 (0.92 to 1.06) | 1.27 (1.2 to 1.35) | -3.26 (-4.12 to -1.69) |
| **Cameroon** | -0.4 (-0.46 to -0.33) | 0.75 (0.54 to 0.97) | -15.86 (-16.73 to -15.1) | -0.07 (-0.13 to 0) | 0.46 (0.19 to 0.73) | -6.93 (-7.93 to -6.17) | -0.23 (-0.29 to -0.16) | 0.6 (0.41 to 0.79) | -11.68 (-12.64 to -10.83) |
| **Canada** | -0.38 (-0.5 to -0.29) | 0.1 (-0.07 to 0.27) | -6.11 (-7.98 to -3.92) | -0.01 (-0.11 to 0.05) | 0.31 (0.17 to 0.45) | -3.33 (-5.02 to -2.27) | -0.23 (-0.34 to -0.15) | 0.15 (0.03 to 0.27) | -5.36 (-6.95 to -3.32) |
| **Central African Republic** | -0.82 (-1.11 to -0.6) | 0.06 (-0.41 to 0.54) | -12.24 (-16.15 to -6.69) | -0.27 (-0.44 to -0.15) | 0.18 (-0.07 to 0.42) | -5.7 (-8.15 to -2.5) | -0.54 (-0.77 to -0.37) | 0.07 (-0.23 to 0.38) | -8.4 (-11.54 to -4.06) |
| **Chad** | 0.39 (0.12 to 0.57) | 1.27 (1.1 to 1.44) | -8.76 (-11.7 to -5.44) | 1.08 (0.97 to 1.17) | 1.46 (1.34 to 1.59) | -2.45 (-4.55 to -1.12) | 0.75 (0.56 to 0.9) | 1.39 (1.3 to 1.47) | -5.19 (-8.55 to -3.12) |
| **Chile** | -0.13 (-0.28 to -0.01) | 0.74 (0.59 to 0.9) | -10.79 (-12.91 to -7.33) | -0.51 (-0.63 to -0.41) | 0 (-0.12 to 0.12) | -8.01 (-9.8 to -5.04) | -0.31 (-0.44 to -0.22) | 0.33 (0.2 to 0.46) | -8.89 (-10.77 to -6.15) |
| **China** | 0.48 (0.46 to 0.5) | 0.5 (0.38 to 0.63) | 0.09 (-0.13 to 0.3) | 0.33 (0.31 to 0.36) | 0.28 (0.14 to 0.43) | 0.98 (0.84 to 1.15) | 0.43 (0.4 to 0.46) | 0.45 (0.28 to 0.61) | 0.18 (0.13 to 0.23) |
| **Colombia** | -0.72 (-0.84 to -0.64) | 0.08 (-0.18 to 0.34) | -10.8 (-12.44 to -9) | -0.7 (-0.74 to -0.65) | -0.18 (-0.38 to 0.01) | -7.8 (-8.53 to -7.22) | -0.68 (-0.73 to -0.63) | -0.06 (-0.25 to 0.13) | -9.11 (-9.87 to -8.43) |
| **Comoros** | 0.5 (0.33 to 0.61) | 1.23 (1.15 to 1.31) | -9.72 (-12.16 to -5.44) | 0.53 (0.38 to 0.65) | 0.85 (0.44 to 1.26) | -3.07 (-5.3 to 0.07) | 0.51 (0.35 to 0.62) | 0.98 (0.91 to 1.05) | -6.14 (-8.43 to -2.98) |
| **Congo** | -0.02 (-0.74 to 0.45) | 1.14 (0.87 to 1.41) | -10.33 (-22.86 to -4.02) | 0.27 (0.16 to 0.36) | 0.66 (0.42 to 0.89) | -4.93 (-6.44 to -2.7) | 0.1 (-0.21 to 0.33) | 0.81 (0.6 to 1.02) | -6.33 (-11.76 to -2.9) |
| **Cook Islands** | 0.82 (0.67 to 0.96) | 0.84 (0.66 to 1.02) | 0.41 (-1 to 0.89) | 0.71 (0.59 to 0.82) | 0.74 (0.63 to 0.85) | 0.71 (0.59 to 0.82) | 0.75 (0.46 to 1.01) | 0.84 (0.73 to 0.94) | 0.34 (-4.02 to 0.69) |
| **Costa Rica** | -0.32 (-0.48 to -0.2) | 0.27 (-0.14 to 0.68) | -9.34 (-11.57 to -5.86) | -0.12 (-0.27 to 0.04) | 0.18 (0.09 to 0.28) | -5.76 (-7.88 to -2.64) | -0.24 (-0.4 to -0.11) | 0.28 (0.13 to 0.44) | -7.55 (-9.84 to -4.51) |
| **Coted'Ivoire** | 0.23 (-0.01 to 0.41) | 1.18 (1.01 to 1.35) | -12.48 (-15.67 to -6.97) | 0.72 (0.54 to 0.87) | 1.14 (0.8 to 1.49) | -3.9 (-6.34 to -1.54) | 0.5 (0.34 to 0.63) | 1.11 (0.83 to 1.38) | -5.51 (-7.32 to -3.18) |
| **Croatia** | 0.26 (0.06 to 0.41) | 0.94 (0.82 to 1.06) | -5.85 (-10.34 to -3.1) | 0.33 (0.12 to 0.54) | 0.82 (0.53 to 1.11) | -3.05 (-5.95 to -1.17) | 0.37 (0.17 to 0.51) | 0.9 (0.78 to 1.02) | -4.75 (-9.24 to -2.2) |
| **Cuba** | -0.36 (-0.72 to -0.08) | 0.54 (0.37 to 0.72) | -17.11 (-21.62 to -10.51) | -0.08 (-0.23 to 0.1) | 0.52 (0.28 to 0.76) | -9.16 (-10.95 to -5.84) | -0.27 (-0.5 to -0.01) | 0.52 (0.39 to 0.66) | -12.18 (-14.55 to -7.72) |
| **Cyprus** | 0.57 (0.52 to 0.61) | 0.9 (0.68 to 1.12) | -4.11 (-4.84 to -3.05) | 0.67 (0.64 to 0.7) | 0.99 (0.91 to 1.06) | -3.7 (-4.27 to -2.73) | 0.61 (0.57 to 0.65) | 0.93 (0.77 to 1.09) | -3.87 (-4.46 to -3.24) |
| **Czechia** | -0.34 (-0.37 to -0.31) | 0.23 (0.11 to 0.34) | -8.1 (-8.67 to -7.69) | -0.1 (-0.16 to -0.05) | 0.25 (0.13 to 0.38) | -4.98 (-5.85 to -4.15) | -0.23 (-0.26 to -0.2) | 0.2 (0.09 to 0.31) | -6.23 (-6.66 to -5.88) |
| **Democratic People's Republic of Korea** | -0.21 (-0.44 to 0.01) | -0.1 (-1.28 to 1.1) | -0.18 (-0.71 to 0.21) | 0.01 (-0.22 to 0.21) | 0.1 (-1.1 to 1.31) | -0.07 (-0.57 to 0.29) | -0.09 (-0.32 to 0.11) | 0.02 (-1.15 to 1.19) | -0.04 (-0.55 to 0.33) |
| **Democratic Republic of the Congo** | -0.12 (-0.27 to -0.01) | 0.98 (0.78 to 1.17) | -14.45 (-16.42 to -13.13) | 0.51 (0.47 to 0.54) | 0.92 (0.81 to 1.02) | -5.13 (-5.7 to -4.61) | 0.15 (0.07 to 0.2) | 0.85 (0.7 to 1) | -8.8 (-9.96 to -7.81) |
| **Denmark** | 0.62 (0.55 to 0.7) | 0.83 (0.72 to 0.94) | -1.52 (-2.86 to -0.72) | 0.4 (0.35 to 0.45) | 0.6 (0.51 to 0.69) | -1.49 (-2.19 to -1.05) | 0.52 (0.46 to 0.58) | 0.73 (0.64 to 0.81) | -1.56 (-2.91 to -0.76) |
| **Djibouti** | -0.14 (-0.37 to 0) | 0.93 (0.86 to 1.01) | -14.22 (-17.52 to -8.74) | 0.07 (-0.03 to 0.16) | 0.56 (0.45 to 0.68) | -6.31 (-7.96 to -3.54) | -0.04 (-0.21 to 0.06) | 0.74 (0.66 to 0.81) | -10.48 (-12.9 to -6.27) |
| **Dominica** | -0.17 (-0.39 to 0.14) | 0.38 (0.19 to 0.58) | -8.41 (-11.63 to -2.85) | -0.09 (-0.3 to 0.06) | 0.34 (-0.08 to 0.76) | -5.53 (-8.75 to -1.56) | -0.14 (-0.32 to 0.1) | 0.25 (0.09 to 0.42) | -6.09 (-8.89 to -1.96) |
| **Dominican Republic** | 0.03 (-0.01 to 0.06) | 0.52 (0.38 to 0.65) | -6.6 (-7.27 to -6.07) | 0.33 (0.3 to 0.36) | 0.63 (0.57 to 0.69) | -3.86 (-4.43 to -3.29) | 0.23 (0.19 to 0.26) | 0.6 (0.51 to 0.69) | -4.97 (-5.54 to -4.51) |
| **Ecuador** | -0.83 (-1.38 to -0.33) | 0.63 (0.23 to 1.03) | -14.72 (-23.49 to -9.56) | -0.3 (-0.59 to -0.1) | 0.62 (0.03 to 1.21) | -10.99 (-15.17 to -6.08) | -0.63 (-1.08 to -0.34) | 0.64 (0.2 to 1.08) | -15.42 (-21.56 to -8.66) |
| **Egypt** | 1.01 (0.93 to 1.06) | 1.9 (1.7 to 2.09) | -10.88 (-12.07 to -9.98) | 1.78 (1.71 to 1.83) | 2.27 (2.05 to 2.48) | -4.96 (-5.91 to -3.66) | 1.38 (1.31 to 1.42) | 2.06 (1.87 to 2.26) | -7.76 (-8.83 to -6.87) |
| **El Salvador** | 0.25 (0.14 to 0.33) | 1.15 (0.79 to 1.51) | -11.91 (-13.57 to -10.59) | 0.28 (0.18 to 0.36) | 0.69 (0.59 to 0.79) | -5.47 (-6.98 to -3.17) | 0.31 (0.24 to 0.37) | 0.95 (0.76 to 1.14) | -8.46 (-9.55 to -7.55) |
| **Equatorial Guinea** | 0.97 (0.63 to 1.24) | 2.45 (2.34 to 2.57) | -17.91 (-22.27 to -11.49) | 0.69 (0.53 to 0.8) | 1.34 (1.17 to 1.52) | -7.95 (-10.32 to -4.93) | 0.81 (0.57 to 0.99) | 1.85 (1.74 to 1.97) | -12.97 (-16.26 to -8.1) |
| **Eritrea** | 1.88 (1.17 to 2.62) | 2.72 (1.87 to 3.57) | -9.4 (-18.23 to -0.48) | 1.08 (1 to 1.17) | 1.41 (1.13 to 1.7) | -3.67 (-4.87 to -1.7) | 1.51 (1.05 to 1.98) | 2.11 (1.54 to 2.67) | -6.72 (-12.76 to -0.64) |
| **Estonia** | 0.48 (0.28 to 0.66) | 1 (0.71 to 1.28) | -8.41 (-11.23 to -4.5) | 0.25 (0.12 to 0.37) | 0.57 (0.41 to 0.72) | -4.3 (-6.04 to -1.92) | 0.38 (0.27 to 0.48) | 0.83 (0.71 to 0.94) | -5.88 (-7.33 to -3.27) |
| **Eswatini** | -1.56 (-1.64 to -1.49) | 0.22 (-0.04 to 0.47) | -24.33 (-25.35 to -23.37) | -1.2 (-1.29 to -1.11) | 0.14 (-0.13 to 0.4) | -18.8 (-20.04 to -17.75) | -1.42 (-1.54 to -1.32) | 0.12 (-0.18 to 0.41) | -22.32 (-23.77 to -21.28) |
| **Ethiopia** | 0.78 (0.68 to 0.87) | 2.29 (2.07 to 2.52) | -17.74 (-18.94 to -16.62) | 0.75 (0.72 to 0.78) | 1.36 (1.21 to 1.5) | -7.79 (-8.24 to -7.39) | 0.77 (0.7 to 0.83) | 1.79 (1.52 to 2.06) | -12.96 (-13.8 to -12.21) |
| **Fiji** | -0.22 (-0.36 to -0.02) | 0.48 (0.42 to 0.54) | -10.72 (-12.52 to -7.22) | -0.15 (-0.23 to -0.06) | 0.23 (0.15 to 0.31) | -5.85 (-7.05 to -3.66) | -0.19 (-0.28 to -0.05) | 0.33 (0.26 to 0.41) | -7.94 (-9.3 to -5.44) |
| **Finland** | 0.25 (0.2 to 0.31) | 0.39 (0.12 to 0.66) | -1.35 (-1.87 to -0.8) | 0.39 (0.34 to 0.43) | 0.51 (0.34 to 0.69) | -1.01 (-1.39 to -0.65) | 0.35 (0.31 to 0.39) | 0.48 (0.27 to 0.69) | -1.13 (-1.48 to -0.78) |
| **France** | -0.2 (-0.26 to -0.13) | 0.14 (0.09 to 0.19) | -3.63 (-5.07 to -2.62) | 0.17 (0.11 to 0.21) | 0.48 (0.35 to 0.61) | -3.73 (-4.59 to -2.73) | -0.04 (-0.09 to 0) | 0.25 (0.16 to 0.34) | -3.49 (-3.98 to -2.48) |
| **Gabon** | -0.1 (-0.26 to 0) | 0.9 (0.7 to 1.1) | -13.4 (-15.59 to -11.61) | -0.06 (-0.13 to -0.02) | 0.43 (0.25 to 0.61) | -6.35 (-7.39 to -5.32) | -0.11 (-0.22 to -0.03) | 0.62 (0.44 to 0.81) | -9.66 (-11.29 to -8.2) |
| **Gambia** | 0.05 (-0.17 to 0.25) | 0.89 (0.35 to 1.42) | -10.66 (-14.33 to -5.94) | 0.42 (0.22 to 0.61) | 0.93 (0.32 to 1.55) | -6.35 (-9.71 to -1.96) | 0.22 (0.04 to 0.39) | 0.89 (0.32 to 1.47) | -8.5 (-11.71 to -4.25) |
| **Georgia** | 0.51 (0.32 to 0.71) | 1.35 (0.71 to 1.99) | -9.33 (-12.43 to -5.2) | 0.54 (0.34 to 0.74) | 1.19 (0.42 to 1.96) | -8.03 (-10.88 to -4.04) | 0.56 (0.31 to 0.8) | 1.18 (0.37 to 1.99) | -9.43 (-12.94 to -4.98) |
| **Germany** | -0.04 (-0.08 to 0) | 0.21 (0.01 to 0.41) | -3.48 (-4.21 to -2.73) | -0.03 (-0.09 to 0.02) | 0.17 (-0.01 to 0.35) | -2.9 (-3.74 to -1.88) | -0.01 (-0.05 to 0.03) | 0.23 (0.05 to 0.41) | -3.15 (-3.85 to -2.43) |
| **Ghana** | -0.22 (-0.26 to -0.19) | 0.63 (0.43 to 0.83) | -11.83 (-12.41 to -11.31) | 0.51 (0.47 to 0.55) | 0.96 (0.79 to 1.12) | -5.82 (-6.54 to -5.22) | 0.2 (0.17 to 0.23) | 0.81 (0.65 to 0.96) | -8.47 (-8.94 to -8.02) |
| **Greece** | -0.05 (-0.12 to 0.04) | 0.22 (-0.16 to 0.61) | -4.04 (-5.24 to -2.29) | 0.21 (0.13 to 0.28) | 0.36 (-0.06 to 0.78) | -1.12 (-2.08 to -0.47) | 0.06 (-0.01 to 0.16) | 0.28 (-0.1 to 0.68) | -3.23 (-4.52 to -1.24) |
| **Greenland** | 0.02 (-0.05 to 0.12) | 0.22 (0.16 to 0.27) | -3 (-4.12 to -0.95) | -0.29 (-0.38 to -0.2) | -0.22 (-0.38 to -0.06) | -0.46 (-0.99 to 0.97) | -0.04 (-0.11 to 0.03) | 0.05 (-0.06 to 0.16) | -0.64 (-0.9 to -0.44) |
| **Grenada** | 0.02 (-0.23 to 0.36) | 0.99 (0.37 to 1.61) | -15.01 (-18.39 to -9.04) | 0.18 (0 to 0.35) | 0.71 (0.49 to 0.93) | -8.32 (-10.85 to -4.37) | 0.13 (-0.06 to 0.41) | 0.68 (0.16 to 1.2) | -8.57 (-11.29 to -3.25) |
| **Guam** | -0.86 (-1.35 to -0.48) | 0.15 (-0.11 to 0.41) | -10.51 (-17.36 to -2.06) | -0.97 (-1.25 to -0.77) | 0.05 (-0.46 to 0.57) | -14.65 (-18.6 to -8.51) | -0.7 (-1.04 to -0.47) | 0.13 (-0.04 to 0.3) | -11.13 (-16.09 to -4.62) |
| **Guatemala** | 0.25 (0.12 to 0.39) | 1.77 (1.19 to 2.35) | -19.28 (-20.74 to -17.92) | 0.19 (0.04 to 0.36) | 0.96 (0.76 to 1.17) | -10.19 (-12.23 to -6.63) | 0.33 (0.16 to 0.48) | 1.38 (0.76 to 2) | -14.24 (-16.44 to -9.91) |
| **Guinea** | -0.26 (-0.49 to -0.11) | 0.89 (0.67 to 1.1) | -15.87 (-18.94 to -9.61) | 0.45 (0.35 to 0.51) | 0.97 (0.88 to 1.06) | -5.97 (-7.43 to -3.42) | 0.09 (-0.1 to 0.21) | 0.95 (0.79 to 1.1) | -11.11 (-13.81 to -6.74) |
| **Guinea-Bissau** | -0.27 (-0.62 to -0.01) | 0.99 (0.5 to 1.48) | -11.22 (-17.09 to -7.18) | 0.68 (0.52 to 0.81) | 1.29 (1.13 to 1.46) | -5.12 (-8.16 to -2.96) | 0.17 (-0.08 to 0.36) | 1.09 (0.84 to 1.35) | -7.84 (-12.34 to -4.85) |
| **Guyana** | -0.37 (-0.63 to -0.2) | 0.92 (0.19 to 1.65) | -17.53 (-20.58 to -12.19) | 0.09 (-0.12 to 0.26) | 0.82 (0.09 to 1.55) | -9.81 (-12.64 to -5.77) | -0.11 (-0.27 to 0.03) | 0.85 (0.3 to 1.41) | -13.24 (-15.21 to -10.94) |
| **Haiti** | 0.58 (-0.87 to 2.02) | 1.1 (0.13 to 2.08) | 0.58 (-0.87 to 2.02) | 0.41 (-1.38 to 2.2) | 0.67 (-0.3 to 1.64) | 0.41 (-1.38 to 2.2) | 0.55 (-1.06 to 2.17) | 0.92 (-0.01 to 1.86) | 0.55 (-1.06 to 2.17) |
| **Honduras** | -0.4 (-0.98 to -0.13) | 0.89 (0.7 to 1.08) | -17.2 (-24.64 to -8.09) | 0.45 (-0.11 to 0.75) | 1.14 (0.93 to 1.35) | -6.31 (-16.55 to -1.1) | 0.07 (-0.47 to 0.33) | 1.06 (0.88 to 1.24) | -13.22 (-20.35 to -4.56) |
| **Hungary** | -0.09 (-0.16 to -0.02) | 0.56 (0.34 to 0.78) | -9.22 (-10.14 to -8.39) | 0.02 (-0.02 to 0.07) | 0.44 (0.28 to 0.6) | -5.92 (-6.63 to -5.32) | -0.05 (-0.09 to -0.01) | 0.46 (0.28 to 0.63) | -7.4 (-8.08 to -6.79) |
| **Iceland** | 0.41 (0.32 to 0.5) | 0.41 (0.04 to 0.79) | 0.1 (-0.2 to 0.27) | 0.18 (0.03 to 0.28) | 0.24 (-0.11 to 0.59) | -1.45 (-3.38 to -0.47) | 0.23 (0.13 to 0.3) | 0.4 (0.29 to 0.51) | -1 (-2.2 to -0.33) |
| **India** | 0.66 (0.62 to 0.68) | 1.34 (1.26 to 1.42) | -8.67 (-9.2 to -8.27) | 0.8 (0.75 to 0.85) | 1.19 (1.05 to 1.33) | -4.91 (-5.69 to -4.34) | 0.74 (0.69 to 0.78) | 1.28 (1.18 to 1.37) | -6.66 (-7.37 to -6.16) |
| **Indonesia** | 0.8 (0.65 to 0.94) | 1.41 (1.28 to 1.54) | -7.73 (-9.69 to -4.32) | 0.83 (0.63 to 0.99) | 1.13 (0.96 to 1.3) | -3.52 (-6.35 to -0.56) | 0.82 (0.65 to 0.97) | 1.26 (1.12 to 1.41) | -5.38 (-7.83 to -2.75) |
| **Iran (Islamic Republic of)** | -0.31 (-0.42 to -0.21) | 1.13 (1.07 to 1.19) | -19.39 (-20.82 to -18.16) | 0.25 (0.17 to 0.33) | 1.05 (0.97 to 1.13) | -10.56 (-11.74 to -9.57) | 0.01 (-0.07 to 0.09) | 1.12 (1.06 to 1.18) | -14.9 (-15.99 to -13.86) |
| **Iraq** | -0.04 (-0.36 to 0.23) | 0.93 (0.49 to 1.38) | -9.83 (-14.32 to -4.37) | 0.63 (0.45 to 0.74) | 1.12 (1.01 to 1.24) | -5.73 (-8.59 to -2.81) | 0.27 (0.02 to 0.43) | 0.99 (0.63 to 1.36) | -7.54 (-11.19 to -3.53) |
| **Ireland** | 0.14 (0.1 to 0.18) | 0.58 (0.44 to 0.73) | -6.01 (-6.5 to -5.48) | -0.1 (-0.16 to -0.05) | 0.29 (0.1 to 0.47) | -5.51 (-6.37 to -4.66) | 0.01 (-0.03 to 0.05) | 0.41 (0.28 to 0.54) | -5.57 (-6.19 to -5.02) |
| **Israel** | 0.24 (0.18 to 0.3) | 0.63 (0.53 to 0.73) | -4.69 (-5.59 to -3.2) | 0.07 (0 to 0.13) | 0.38 (0.17 to 0.6) | -4.22 (-5.21 to -2.59) | 0.14 (0.1 to 0.19) | 0.49 (0.34 to 0.63) | -4.66 (-5.31 to -4.1) |
| **Italy** | -0.22 (-0.36 to -0.14) | 0.11 (-0.03 to 0.24) | -3.58 (-6.5 to -2.24) | -0.04 (-0.13 to 0.03) | 0.23 (0.07 to 0.39) | -2.53 (-4.29 to -1.54) | -0.15 (-0.26 to -0.07) | 0.13 (0 to 0.25) | -3 (-5.19 to -1.87) |
| **Jamaica** | 0.09 (-0.12 to 0.28) | 0.72 (0.14 to 1.3) | -10.22 (-12.49 to -6.41) | 0.26 (0.15 to 0.35) | 0.55 (0.18 to 0.91) | -5.16 (-6.74 to -2.89) | 0.29 (0.08 to 0.56) | 0.77 (0.12 to 1.43) | -7.38 (-10.42 to -2.36) |
| **Japan** | 0.34 (0.3 to 0.37) | 0.37 (0.21 to 0.53) | -0.32 (-0.61 to -0.17) | 0.55 (0.51 to 0.58) | 0.63 (0.46 to 0.8) | -0.26 (-0.62 to 0.01) | 0.44 (0.41 to 0.46) | 0.49 (0.38 to 0.59) | -0.21 (-0.4 to -0.09) |
| **Jordan** | -0.73 (-0.86 to -0.66) | 0.44 (0.33 to 0.55) | -16.13 (-17.87 to -14.89) | 0.17 (0.12 to 0.22) | 0.75 (0.64 to 0.85) | -7.67 (-8.42 to -7.03) | -0.26 (-0.35 to -0.2) | 0.66 (0.57 to 0.75) | -12.02 (-13.34 to -10.91) |
| **Kazakhstan** | -2.69 (-2.78 to -2.6) | -2.03 (-2.38 to -1.67) | -11.71 (-12.97 to -10.41) | -2 (-2.15 to -1.86) | -1.58 (-2.03 to -1.13) | -7.83 (-10 to -5.21) | -2.32 (-2.45 to -2.21) | -1.76 (-2.03 to -1.48) | -9.82 (-11.74 to -7.43) |
| **Kenya** | 0.21 (0.05 to 0.32) | 1.42 (1.18 to 1.67) | -14.85 (-17.05 to -13.24) | 0.67 (0.57 to 0.74) | 1.32 (1.19 to 1.46) | -8.11 (-9.57 to -6.92) | 0.44 (0.31 to 0.52) | 1.37 (1.18 to 1.57) | -11.6 (-13.44 to -10.2) |
| **Kiribati** | 0.68 (0.49 to 0.79) | 0.55 (0.44 to 0.65) | 1.85 (0.13 to 4.16) | 0.64 (0.61 to 0.67) | 0.61 (0.56 to 0.66) | 0.74 (0.23 to 1.31) | 0.61 (0.51 to 0.69) | 0.59 (0.49 to 0.68) | 0.36 (0 to 0.51) |
| **Kuwait** | 0.21 (-0.18 to 0.56) | 0.77 (0.56 to 0.97) | -4.9 (-12.47 to -0.19) | -0.34 (-0.6 to -0.13) | -0.31 (-1.42 to 0.82) | -2.79 (-5.05 to -1.5) | 0.17 (-0.21 to 0.42) | 0.57 (-0.53 to 1.69) | -3.67 (-9.52 to -0.81) |
| **Kyrgyzstan** | -1.54 (-1.77 to -1.34) | -1.1 (-1.62 to -0.58) | -7.07 (-10.65 to -4.19) | -0.92 (-1.12 to -0.75) | -0.61 (-1.11 to -0.11) | -3.37 (-6.64 to -1.23) | -1.24 (-1.45 to -1.05) | -0.85 (-1.59 to -0.1) | -5.3 (-8.75 to -2.91) |
| **Lao People's Democratic Republic** | 0.99 (0.92 to 1.1) | 1.33 (1.29 to 1.37) | -4.31 (-5.37 to -2.16) | 1.11 (1.08 to 1.17) | 1.31 (1.25 to 1.37) | -2.1 (-2.7 to -1.07) | 1.02 (0.96 to 1.09) | 1.3 (1.24 to 1.35) | -3.8 (-4.67 to -1.97) |
| **Latvia** | 0.05 (-0.1 to 0.2) | 0.77 (0.22 to 1.33) | -10.33 (-12.22 to -7.54) | 0.27 (0.18 to 0.37) | 0.69 (0.43 to 0.95) | -5.82 (-7.1 to -3.8) | 0.2 (0.12 to 0.29) | 0.73 (0.56 to 0.91) | -7.47 (-8.5 to -5.9) |
| **Lebanon** | 0.11 (0.03 to 0.19) | 1.47 (1.29 to 1.64) | -17.49 (-18.41 to -16.48) | 0.07 (0.01 to 0.11) | 0.82 (0.59 to 1.04) | -10.36 (-11.15 to -9.7) | 0.09 (0.02 to 0.14) | 1.19 (1.04 to 1.34) | -14.15 (-15.17 to -13.35) |
| **Lesotho** | -1.59 (-1.86 to -1.32) | -0.33 (-1.43 to 0.78) | -22.57 (-25.82 to -19.05) | -0.79 (-1.08 to -0.58) | 0.14 (-0.61 to 0.89) | -13.31 (-17 to -7.86) | -1.24 (-1.7 to -0.87) | -0.14 (-0.92 to 0.64) | -16.55 (-22.54 to -9.02) |
| **Liberia** | 0.69 (0 to 1.31) | 1.86 (0.78 to 2.94) | -8.46 (-18.62 to -0.85) | 1.38 (1.03 to 1.6) | 1.76 (1.24 to 2.28) | 0.71 (-1.16 to 1.45) | 1.01 (0.6 to 1.51) | 1.62 (0.76 to 2.49) | -6.83 (-12.75 to 0.25) |
| **Libya** | -0.51 (-0.71 to -0.37) | 0.06 (-0.16 to 0.27) | -8.35 (-11.34 to -4.57) | -0.04 (-0.11 to 0.02) | 0.33 (0.12 to 0.54) | -4.45 (-5.4 to -3.08) | -0.27 (-0.43 to -0.15) | 0.2 (0.03 to 0.37) | -6.54 (-9.12 to -3.45) |
| **Lithuania** | 0.05 (-0.02 to 0.12) | 0.73 (0.56 to 0.9) | -8.95 (-9.91 to -8.2) | 0.18 (0.08 to 0.26) | 0.57 (0.27 to 0.87) | -5.53 (-6.88 to -3.25) | 0.11 (0.05 to 0.16) | 0.6 (0.45 to 0.75) | -6.8 (-7.69 to -6.2) |
| **Luxembourg** | -0.31 (-0.43 to -0.17) | 0.06 (-0.06 to 0.18) | -4.8 (-6.91 to -2.33) | -0.08 (-0.19 to 0.01) | 0.19 (0.02 to 0.35) | -3.42 (-5.11 to -2.25) | -0.22 (-0.33 to -0.08) | 0.16 (0.02 to 0.3) | -4.58 (-6.59 to -2.33) |
| **Madagascar** | -0.55 (-0.74 to -0.44) | 0.46 (0.39 to 0.53) | -13.93 (-16.68 to -8.87) | -0.06 (-0.13 to -0.02) | 0.3 (0.22 to 0.37) | -5.02 (-6.05 to -3.1) | -0.27 (-0.39 to -0.2) | 0.38 (0.29 to 0.48) | -8.91 (-10.68 to -5.53) |
| **Malawi** | 0.7 (0.56 to 0.91) | 2.09 (1.87 to 2.31) | -18.54 (-20.44 to -15.69) | 0.95 (0.81 to 1.07) | 1.8 (1.6 to 2) | -11.79 (-13.56 to -9.95) | 0.81 (0.68 to 0.98) | 1.92 (1.66 to 2.19) | -14.82 (-16.62 to -12.48) |
| **Malaysia** | 0 (-0.06 to 0.06) | 0.47 (0.4 to 0.54) | -6.93 (-7.9 to -5.86) | 0.33 (0.27 to 0.4) | 0.59 (0.52 to 0.66) | -3.37 (-4.34 to -1.92) | 0.15 (0.11 to 0.2) | 0.52 (0.44 to 0.61) | -5.34 (-6.11 to -4.43) |
| **Maldives** | 0.29 (0.14 to 0.43) | 0.73 (0.56 to 0.9) | -5.9 (-8.09 to -3.56) | 0.32 (0.27 to 0.36) | 0.56 (0.31 to 0.81) | -3.17 (-3.82 to -2.04) | 0.22 (0.08 to 0.32) | 0.58 (0.43 to 0.72) | -4.81 (-6.85 to -2.79) |
| **Mali** | -0.28 (-0.46 to -0.15) | 1.22 (1.04 to 1.4) | -19.37 (-21.62 to -17.35) | 0.4 (0.33 to 0.44) | 1.05 (0.96 to 1.13) | -7.93 (-8.89 to -7.11) | 0.08 (-0.04 to 0.17) | 1.2 (0.95 to 1.44) | -14 (-15.63 to -12.54) |
| **Malta** | -0.05 (-0.14 to 0.04) | 0.19 (0.02 to 0.36) | -3.34 (-5.28 to -2.1) | -0.04 (-0.17 to 0.08) | -0.04 (-0.36 to 0.27) | -1.03 (-1.77 to -0.54) | -0.05 (-0.13 to 0.03) | 0.09 (-0.13 to 0.32) | -1.05 (-1.67 to -0.62) |
| **Marshall Islands** | 0.76 (0.75 to 0.77) | 0.75 (0.71 to 0.79) | 0.86 (0.83 to 0.93) | 0.49 (0.49 to 0.5) | 0.49 (0.44 to 0.53) | 0.5 (0.44 to 0.62) | 0.65 (0.64 to 0.65) | 0.64 (0.6 to 0.68) | 0.69 (0.65 to 0.72) |
| **Mauritania** | 0.06 (-0.24 to 0.29) | 0.8 (0.59 to 1.01) | -9.29 (-13.88 to -4.66) | 0.77 (0.66 to 0.87) | 1.01 (0.75 to 1.26) | -1.61 (-3.21 to 0.53) | 0.46 (0.25 to 0.6) | 0.96 (0.73 to 1.2) | -5.63 (-8.7 to -2.32) |
| **Mauritius** | 0.69 (0.49 to 0.9) | 1.02 (0.49 to 1.56) | 0.27 (-2.26 to 0.67) | 0.25 (0.02 to 0.44) | 0.52 (-0.29 to 1.33) | 1.19 (0.34 to 4.04) | 0.45 (0.35 to 0.55) | 0.74 (0.23 to 1.24) | -3.74 (-5.28 to -1.93) |
| **Mexico** | -1.38 (-1.86 to -1.02) | 0 (-0.18 to 0.18) | -18.36 (-24.41 to -11.65) | -1.12 (-1.34 to -0.91) | -0.43 (-0.5 to -0.37) | -9.6 (-12.53 to -6.13) | -1.24 (-1.58 to -0.96) | -0.2 (-0.32 to -0.09) | -13.87 (-18.51 to -8.84) |
| **Micronesia (Federated States of)** | 0.64 (0.62 to 0.67) | 0.63 (0.56 to 0.71) | 0.45 (0.39 to 0.49) | 0.51 (0.47 to 0.56) | 0.53 (0.48 to 0.57) | 0.25 (0.06 to 0.39) | 0.55 (0.52 to 0.6) | 0.58 (0.53 to 0.62) | 0.32 (0.19 to 0.42) |
| **Monaco** | 0.33 (0.28 to 0.39) | 0.73 (0.66 to 0.81) | -5.82 (-6.49 to -4.94) | 0.83 (0.8 to 0.86) | 1.12 (1.03 to 1.21) | -3.41 (-3.78 to -2.89) | 0.57 (0.52 to 0.63) | 0.92 (0.83 to 1.01) | -4.61 (-5.34 to -3.71) |
| **Mongolia** | 0.34 (0.25 to 0.42) | 0.7 (0.47 to 0.92) | -4.15 (-5.53 to -2.16) | 0.84 (0.78 to 0.9) | 1.18 (0.99 to 1.38) | -4.3 (-5.21 to -3.14) | 0.52 (0.43 to 0.59) | 0.89 (0.72 to 1.07) | -4.75 (-6.12 to -2.67) |
| **Montenegro** | -0.44 (-0.53 to -0.35) | 0.35 (0.23 to 0.47) | -11.65 (-13 to -10.24) | -0.01 (-0.08 to 0.05) | 0.55 (0.45 to 0.66) | -7.99 (-8.98 to -7.15) | -0.15 (-0.26 to -0.04) | 0.43 (0.28 to 0.58) | -8.78 (-10.35 to -5.83) |
| **Morocco** | 0.38 (0.2 to 0.51) | 1 (0.96 to 1.05) | -5.33 (-8.77 to -3.22) | 0.77 (0.69 to 0.83) | 1.08 (1.05 to 1.12) | -2.33 (-3.85 to -1.31) | 0.53 (0.41 to 0.62) | 1 (0.95 to 1.05) | -3.67 (-6.28 to -2.09) |
| **Mozambique** | 0.09 (-0.05 to 0.27) | 1.19 (1.06 to 1.32) | -15.22 (-17.09 to -12.63) | 0.33 (0.26 to 0.44) | 1.06 (0.82 to 1.29) | -10.37 (-11.56 to -8.9) | 0.2 (0.1 to 0.34) | 1.09 (0.97 to 1.22) | -12.84 (-14.4 to -10.8) |
| **Myanmar** | 0.77 (0.35 to 0.96) | 1.21 (1.02 to 1.4) | -7.15 (-13.56 to -1.11) | 0.65 (0.39 to 0.9) | 0.61 (0.34 to 0.87) | 0.65 (0.39 to 0.9) | 0.91 (0.68 to 1.14) | 0.91 (0.7 to 1.12) | 0.91 (0.68 to 1.14) |
| **Namibia** | -0.21 (-0.42 to 0.02) | 1.21 (1 to 1.42) | -20.03 (-22.35 to -16.93) | 0.19 (0.02 to 0.36) | 0.96 (0.72 to 1.2) | -11.63 (-13.81 to -8.79) | 0.02 (-0.14 to 0.2) | 1.09 (0.89 to 1.3) | -15.73 (-17.54 to -13.33) |
| **Nauru** | 0.24 (0.22 to 0.26) | 0.19 (0.09 to 0.28) | 1.13 (0.95 to 1.27) | 0.13 (0.12 to 0.14) | 0.09 (0.05 to 0.13) | 0.87 (0.79 to 0.92) | 0.18 (0.16 to 0.2) | 0.13 (0.07 to 0.2) | 0.94 (0.78 to 1.07) |
| **Nepal** | 0.49 (0.44 to 0.53) | 1.46 (1.36 to 1.56) | -12.5 (-13.15 to -12) | 0.59 (0.53 to 0.64) | 1.08 (1 to 1.16) | -6.46 (-7.3 to -5.87) | 0.57 (0.53 to 0.61) | 1.31 (1.23 to 1.38) | -9.52 (-10.12 to -9.08) |
| **Netherlands** | -0.23 (-0.3 to -0.18) | 0.04 (-0.03 to 0.12) | -2.72 (-3.67 to -2.12) | 0.02 (-0.03 to 0.05) | 0.23 (0.14 to 0.31) | -2.59 (-3.3 to -1.99) | -0.11 (-0.16 to -0.07) | 0.12 (0.05 to 0.18) | -2.26 (-2.97 to -1.79) |
| **New Zealand** | 0.4 (0.26 to 0.51) | 0.41 (0.04 to 0.79) | 0.27 (-0.17 to 0.33) | 0.01 (-0.04 to 0.04) | 0.09 (-0.1 to 0.29) | -1.11 (-1.68 to -0.79) | 0.19 (0.13 to 0.25) | 0.26 (0.04 to 0.47) | -0.72 (-1.22 to -0.4) |
| **Nicaragua** | -1.48 (-1.79 to -1.25) | 0.46 (0.29 to 0.62) | -24.94 (-28.88 to -16.62) | -0.92 (-1.13 to -0.76) | 0.17 (0 to 0.35) | -15.41 (-18.11 to -10.17) | -1.08 (-1.36 to -0.89) | 0.39 (0.21 to 0.57) | -19.43 (-23.3 to -12.78) |
| **Niger** | 0.46 (0.23 to 0.61) | 1.52 (1.34 to 1.7) | -13.71 (-16.76 to -8.51) | 1.14 (1.06 to 1.21) | 1.58 (1.3 to 1.85) | -3.45 (-4.49 to -2.34) | 0.82 (0.65 to 0.93) | 1.53 (1.35 to 1.71) | -9.67 (-11.95 to -5.75) |
| **Nigeria** | 0.63 (0.53 to 0.7) | 1.79 (1.63 to 1.95) | -14.11 (-15.6 to -12.97) | 1.22 (1.17 to 1.25) | 1.65 (1.53 to 1.77) | -4.45 (-5.16 to -3.87) | 1.13 (1.05 to 1.19) | 1.91 (1.74 to 2.07) | -8.84 (-10.26 to -7.73) |
| **Niue** | 0.47 (0.41 to 0.53) | 0.54 (0.44 to 0.64) | 0.09 (-0.2 to 0.3) | 0.3 (0.25 to 0.35) | 0.36 (0.18 to 0.54) | 0.02 (-0.19 to 0.16) | 0.4 (0.32 to 0.46) | 0.5 (0.21 to 0.8) | -0.08 (-0.49 to 0.19) |
| **North Macedonia** | -0.55 (-0.61 to -0.51) | 0.68 (0.63 to 0.72) | -16.37 (-17.22 to -15.78) | -0.14 (-0.22 to -0.09) | 0.57 (0.46 to 0.68) | -9.58 (-10.73 to -8.68) | -0.38 (-0.45 to -0.34) | 0.57 (0.5 to 0.65) | -13.26 (-14.28 to -12.57) |
| **Northern Mariana Islands** | -0.07 (-0.14 to 0.03) | 0.29 (0.18 to 0.39) | -5.17 (-6.36 to -3.33) | 0.17 (0.02 to 0.28) | 0.48 (0.4 to 0.56) | -2.61 (-5.62 to -1.24) | 0.09 (0.06 to 0.13) | 0.4 (0.26 to 0.55) | -4.45 (-4.97 to -3.98) |
| **Norway** | 0.68 (0.64 to 0.72) | 0.85 (0.67 to 1.02) | -1.16 (-1.85 to -0.75) | 0.52 (0.45 to 0.58) | 0.7 (0.43 to 0.96) | -1.25 (-2.53 to -0.5) | 0.57 (0.53 to 0.61) | 0.74 (0.47 to 1) | -1.19 (-1.8 to -0.76) |
| **Oman** | -0.43 (-0.47 to -0.4) | 0.71 (0.57 to 0.85) | -15.6 (-16.14 to -15.14) | -0.38 (-0.44 to -0.33) | 0.32 (0.14 to 0.51) | -9.45 (-10.39 to -8.6) | -0.37 (-0.41 to -0.33) | 0.59 (0.41 to 0.77) | -13.21 (-13.91 to -12.59) |
| **Pakistan** | -0.1 (-0.31 to 0.05) | 0.83 (0.76 to 0.91) | -12.51 (-15.27 to -7.73) | 0.69 (0.59 to 0.76) | 1.14 (1.06 to 1.22) | -5.48 (-7.03 to -3.02) | 0.27 (0.12 to 0.37) | 0.97 (0.89 to 1.04) | -9.2 (-11.32 to -5.52) |
| **Palau** | 0.15 (0.13 to 0.16) | 0.15 (0.1 to 0.2) | 0.09 (-0.2 to 0.28) | 0.05 (0.05 to 0.06) | 0.07 (0.03 to 0.11) | -0.07 (-0.2 to 0) | 0.1 (0.09 to 0.11) | 0.12 (0.07 to 0.16) | -0.14 (-0.34 to -0.02) |
| **Palestine** | -0.09 (-0.27 to 0.12) | 0.61 (0.23 to 0.99) | -9.8 (-12.27 to -5.93) | 0.23 (0.17 to 0.28) | 0.6 (0.39 to 0.81) | -4.98 (-5.83 to -3.89) | 0.01 (-0.1 to 0.12) | 0.5 (0.05 to 0.96) | -6.9 (-8.55 to -4.41) |
| **Panama** | -0.28 (-0.52 to -0.12) | 0.29 (-0.12 to 0.7) | -6.81 (-11.07 to -4.32) | -0.04 (-0.19 to 0.06) | 0.26 (-0.18 to 0.7) | -2.81 (-5.14 to -1.45) | -0.17 (-0.34 to -0.06) | 0.29 (0.07 to 0.5) | -5.12 (-8.05 to -3.32) |
| **Papua New Guinea** | -0.2 (-0.38 to 0.08) | 0.69 (0.57 to 0.82) | -11.81 (-14.38 to -6.8) | 0.12 (0.03 to 0.23) | 0.45 (0.32 to 0.58) | -4.87 (-6.27 to -2.67) | -0.07 (-0.19 to 0.11) | 0.6 (0.47 to 0.72) | -9.16 (-11.12 to -5.97) |
| **Paraguay** | 0.1 (-0.05 to 0.32) | 1.12 (0.95 to 1.29) | -14.42 (-16.39 to -10.84) | 0.26 (0.2 to 0.35) | 0.97 (0.92 to 1.02) | -9.64 (-10.49 to -8.36) | 0.23 (0.12 to 0.39) | 1.11 (1.07 to 1.15) | -11.86 (-13.32 to -9.49) |
| **Peru** | -1.56 (-1.86 to -1.35) | 0.71 (0.59 to 0.83) | -28.74 (-32.07 to -26.34) | -0.55 (-0.67 to -0.44) | 0.76 (0.14 to 1.39) | -17.2 (-19 to -15.65) | -1.04 (-1.22 to -0.91) | 0.73 (0.47 to 0.98) | -22.01 (-24.12 to -20.48) |
| **Philippines** | -0.41 (-0.63 to -0.12) | 0.29 (0.23 to 0.34) | -10.02 (-12.76 to -4.89) | 0.11 (0.03 to 0.24) | 0.55 (0.46 to 0.63) | -6.47 (-7.75 to -4.19) | -0.26 (-0.42 to -0.05) | 0.39 (0.34 to 0.44) | -7.99 (-10.26 to -3.89) |
| **Poland** | 0.38 (0.29 to 0.45) | 1.05 (0.81 to 1.29) | -9.56 (-10.8 to -8.52) | 0.61 (0.55 to 0.65) | 1.09 (1.01 to 1.16) | -5.9 (-6.85 to -5.19) | 0.48 (0.41 to 0.54) | 1.03 (0.83 to 1.23) | -7.08 (-8.15 to -6.19) |
| **Portugal** | 0.53 (0.47 to 0.59) | 0.88 (0.73 to 1.04) | -3.19 (-4.62 to -2.27) | -0.1 (-0.16 to -0.05) | 0.19 (0.06 to 0.32) | -3.71 (-4.77 to -2.35) | 0.26 (0.21 to 0.32) | 0.56 (0.4 to 0.72) | -2.82 (-4.08 to -2.03) |
| **Puerto Rico** | -0.27 (-0.34 to -0.2) | -0.13 (-0.38 to 0.13) | -2.4 (-2.86 to -2.03) | 0.02 (-0.06 to 0.1) | 0.11 (-0.22 to 0.43) | 0.13 (-0.82 to 1.89) | -0.25 (-0.32 to -0.19) | -0.06 (-0.41 to 0.29) | -1.83 (-2.21 to -1.52) |
| **Qatar** | 0.48 (0.35 to 0.57) | 0.81 (0.74 to 0.88) | -3.73 (-5.62 to -1.7) | 0.23 (0.17 to 0.29) | 0.51 (0.29 to 0.74) | -3.3 (-4.02 to -2.35) | 0.37 (0.29 to 0.44) | 0.58 (0.47 to 0.69) | -2.47 (-3.61 to -1.01) |
| **Republic of Korea** | 0.41 (0.38 to 0.43) | 0.52 (0.43 to 0.62) | -1.33 (-1.75 to -0.78) | 0.27 (0.24 to 0.3) | 0.35 (0.23 to 0.46) | -0.63 (-1.34 to -0.17) | 0.32 (0.3 to 0.34) | 0.39 (0.3 to 0.48) | -0.53 (-0.9 to -0.23) |
| **Republic of Moldova** | -0.48 (-0.55 to -0.41) | -0.03 (-0.25 to 0.19) | -6.75 (-7.8 to -5.87) | -0.47 (-0.55 to -0.39) | -0.32 (-0.64 to 0) | -3.59 (-4.97 to -1.78) | -0.47 (-0.53 to -0.4) | -0.15 (-0.32 to 0.02) | -5.32 (-6.25 to -4.35) |
| **Romania** | 0.93 (0.88 to 0.98) | 1.59 (1.48 to 1.7) | -8.13 (-8.97 to -7.37) | 0.98 (0.93 to 1.03) | 1.45 (1.42 to 1.49) | -3.54 (-4.11 to -2.9) | 0.96 (0.9 to 1) | 1.51 (1.31 to 1.72) | -6.43 (-7.39 to -5.52) |
| **Russian Federation** | -1.23 (-1.35 to -1.11) | -0.4 (-0.79 to -0.01) | -13.08 (-14.55 to -11.69) | -0.37 (-0.45 to -0.31) | 0.19 (0.01 to 0.38) | -8.57 (-9.7 to -7.74) | -0.76 (-0.83 to -0.7) | -0.08 (-0.29 to 0.12) | -10.44 (-11.29 to -9.67) |
| **Rwanda** | -0.19 (-5.31 to 6.19) | -3.03 (-3.93 to -2.12) | 1.96 (-56.11 to 29.2) | 1.92 (0.04 to 3.8) | -1.11 (-1.92 to -0.31) | 1.92 (0.04 to 3.8) | -0.55 (-5.46 to 5.64) | -3.31 (-4.01 to -2.61) | 1.45 (-56.51 to 37.16) |
| **Saint Kitts and Nevis** | 0.59 (0.44 to 0.74) | 1.14 (0.56 to 1.72) | -7.75 (-10.02 to -4.48) | -0.03 (-0.18 to 0.17) | 0.08 (-0.41 to 0.57) | -1.37 (-2.46 to -0.63) | 0.37 (0.21 to 0.51) | 0.64 (0.27 to 1) | -5.33 (-7.75 to -1.81) |
| **Saint Lucia** | -0.07 (-0.21 to 0.15) | 0.86 (0.71 to 1.01) | -12.29 (-14.2 to -8.59) | -0.26 (-0.4 to -0.12) | 0.04 (-0.12 to 0.2) | -4.74 (-7.01 to -2.15) | -0.06 (-0.18 to 0.08) | 0.66 (0.49 to 0.83) | -10.5 (-12.29 to -8) |
| **Saint Vincent and the Grenadines** | 0.26 (0.13 to 0.39) | 0.63 (0.05 to 1.21) | -5.29 (-6.93 to -2.71) | 0.01 (-0.09 to 0.09) | 0.01 (-0.38 to 0.4) | -0.7 (-1.25 to -0.34) | 0.23 (0.11 to 0.34) | 0.49 (0.05 to 0.93) | -4.3 (-6.03 to -2.19) |
| **Samoa** | 0.09 (0.03 to 0.15) | 0.08 (0.03 to 0.13) | 0.09 (0.03 to 0.15) | 0.24 (0.16 to 0.32) | 0.23 (0.16 to 0.3) | 0.24 (0.16 to 0.32) | 0.14 (0.07 to 0.2) | 0.13 (0.07 to 0.18) | 0.14 (0.07 to 0.2) |
| **San Marino** | -1.25 (-1.5 to -1.01) | 0.04 (0 to 0.07) | -13.27 (-19.56 to -9.35) | -1.28 (-1.63 to -1.08) | -0.23 (-0.39 to -0.07) | -13.67 (-18.47 to -7.81) | -1.23 (-1.53 to -0.98) | 0.03 (-0.03 to 0.08) | -12.74 (-19.07 to -8.74) |
| **Sao Tome and Principe** | 0.69 (0.59 to 0.77) | 1.29 (1.16 to 1.42) | -8.08 (-9.45 to -7.12) | 0.81 (0.73 to 0.86) | 1.13 (0.98 to 1.29) | -3.76 (-4.76 to -1.96) | 0.82 (0.75 to 0.87) | 1.3 (1.2 to 1.41) | -5.83 (-6.76 to -5.11) |
| **Saudi Arabia** | 0.42 (0.29 to 0.52) | 0.83 (0.69 to 0.97) | -3.22 (-5.67 to -1.8) | 0.84 (0.78 to 0.88) | 1.1 (1 to 1.2) | -2.32 (-3.2 to -1.67) | 0.61 (0.51 to 0.7) | 0.96 (0.9 to 1.02) | -2.71 (-4.54 to -1.69) |
| **Senegal** | 0.36 (0.23 to 0.46) | 1.57 (1.35 to 1.78) | -15.53 (-17.13 to -14) | 0.84 (0.65 to 0.99) | 1.48 (1.22 to 1.74) | -7.12 (-9.69 to -3.55) | 0.61 (0.45 to 0.73) | 1.45 (1.2 to 1.7) | -10.84 (-12.99 to -7.13) |
| **Serbia** | 0.07 (-0.17 to 0.21) | 0.67 (0.5 to 0.84) | -8.2 (-11.48 to -4.64) | 0.23 (0.17 to 0.29) | 0.57 (0.41 to 0.74) | -4.54 (-5.45 to -2.95) | 0.18 (0.07 to 0.29) | 0.6 (0.45 to 0.74) | -6.26 (-7.91 to -3.79) |
| **Seychelles** | 0.43 (0.38 to 0.51) | 0.89 (0.83 to 0.96) | -6.4 (-7.2 to -5.2) | 0.04 (0.01 to 0.07) | 0.55 (0.47 to 0.63) | -7.03 (-7.47 to -6.67) | 0.21 (0.17 to 0.23) | 0.7 (0.62 to 0.77) | -6.66 (-7.26 to -6.28) |
| **Sierra Leone** | 0.33 (0.05 to 0.54) | 1.1 (0.87 to 1.33) | -9.21 (-12.95 to -4.16) | 1.11 (0.95 to 1.24) | 1.48 (1.29 to 1.67) | -4.01 (-6.4 to -0.98) | 0.67 (0.47 to 0.81) | 1.18 (1 to 1.37) | -6.38 (-9.18 to -2.46) |
| **Singapore** | 0.08 (-0.04 to 0.17) | 0.23 (-0.16 to 0.62) | -2.35 (-4.35 to -1.16) | 0.22 (0.12 to 0.3) | 0.37 (0.01 to 0.73) | -1.31 (-2.78 to -0.58) | 0.11 (0.01 to 0.19) | 0.24 (-0.2 to 0.68) | -1.36 (-2.61 to -0.72) |
| **Slovakia** | -0.27 (-0.38 to -0.18) | 0.46 (0.41 to 0.52) | -10.42 (-11.92 to -8.84) | -0.06 (-0.12 to -0.01) | 0.38 (0.31 to 0.46) | -6.46 (-7.41 to -5.45) | -0.22 (-0.32 to -0.16) | 0.34 (0.27 to 0.42) | -8.56 (-9.89 to -7.25) |
| **Slovenia** | 0.44 (0.33 to 0.52) | 0.96 (0.65 to 1.26) | -5 (-6.56 to -3.46) | 0.29 (0.22 to 0.35) | 0.75 (0.35 to 1.15) | -5.27 (-6.13 to -4.51) | 0.41 (0.32 to 0.5) | 0.84 (0.55 to 1.13) | -4.24 (-5.42 to -2.78) |
| **Solomon Islands** | 0.64 (0.6 to 0.68) | 0.66 (0.53 to 0.79) | 0.34 (0.05 to 0.57) | 0.84 (0.78 to 0.9) | 0.87 (0.68 to 1.07) | 0.61 (0.3 to 0.87) | 0.69 (0.64 to 0.73) | 0.73 (0.57 to 0.88) | 0.35 (0.04 to 0.61) |
| **Somalia** | -0.19 (-0.93 to 0.38) | 0.67 (-0.05 to 1.39) | -5.59 (-11.08 to -2.78) | 0.19 (-0.45 to 0.64) | 0.63 (0.37 to 0.89) | -2.98 (-7.91 to -0.75) | 0.04 (-0.64 to 0.56) | 0.67 (0.19 to 1.15) | -4.16 (-9.41 to -1.65) |
| **South Africa** | -1.44 (-1.68 to -1.26) | 0.24 (0.09 to 0.39) | -22.13 (-25.2 to -19.59) | -0.49 (-0.63 to -0.39) | 0.39 (0.14 to 0.65) | -12.33 (-14.31 to -10.48) | -0.93 (-1.09 to -0.82) | 0.27 (0.05 to 0.49) | -16.55 (-18.69 to -14.79) |
| **South Sudan** | -0.38 (-0.62 to -0.2) | 0.48 (0.2 to 0.75) | -13.91 (-17.26 to -7.69) | -0.01 (-0.12 to 0.09) | 0.46 (0.27 to 0.65) | -5.35 (-6.96 to -2.77) | -0.11 (-0.27 to 0.04) | 0.61 (0.34 to 0.89) | -8.57 (-11.21 to -5.56) |
| **Spain** | -0.06 (-0.22 to 0.11) | 0.38 (0.26 to 0.49) | -3.79 (-6.87 to -1.99) | -0.1 (-0.2 to -0.03) | 0.25 (0.07 to 0.42) | -3.78 (-5.44 to -2.38) | -0.07 (-0.21 to 0.06) | 0.31 (0.19 to 0.44) | -3.45 (-5.97 to -1.99) |
| **Sri Lanka** | 0.19 (-0.03 to 0.36) | 0.76 (0.12 to 1.41) | -5.21 (-9.31 to -2.58) | -0.34 (-0.7 to -0.05) | 0.01 (-0.23 to 0.25) | -3.28 (-9.53 to -0.08) | -0.11 (-0.39 to 0.09) | 0.36 (-0.1 to 0.82) | -4.35 (-9.9 to -1.39) |
| **Sudan** | 0.26 (0.06 to 0.42) | 1.34 (1.21 to 1.48) | -13.05 (-16.14 to -8.79) | 0.61 (0.51 to 0.67) | 1.12 (1.04 to 1.21) | -6.12 (-7.54 to -3.82) | 0.46 (0.31 to 0.57) | 1.3 (1.16 to 1.45) | -9.93 (-12.29 to -6.54) |
| **Suriname** | -0.05 (-0.33 to 0.14) | 0.92 (0.71 to 1.13) | -15.23 (-18.73 to -9.55) | -0.06 (-0.17 to 0.07) | 0.54 (0.37 to 0.71) | -8.25 (-9.52 to -5.77) | 0.01 (-0.12 to 0.2) | 0.75 (0.6 to 0.89) | -10.83 (-12.82 to -7.47) |
| **Sweden** | 0.3 (0.19 to 0.37) | 0.67 (0.51 to 0.83) | -4.12 (-6.02 to -1.92) | 0.26 (0.17 to 0.33) | 0.52 (0.38 to 0.65) | -2.98 (-4.48 to -1.14) | 0.28 (0.18 to 0.35) | 0.59 (0.44 to 0.73) | -3.51 (-5.2 to -1.53) |
| **Switzerland** | 0.09 (-0.04 to 0.2) | 0.36 (0.14 to 0.58) | -2.74 (-4.28 to -1.78) | 0.24 (0.13 to 0.33) | 0.49 (0.13 to 0.84) | -3.25 (-5.15 to -1.88) | 0.16 (0.07 to 0.24) | 0.37 (0.08 to 0.66) | -2.55 (-3.56 to -1.7) |
| **Syrian Arab Republic** | -0.18 (-0.47 to 0.08) | -0.06 (-0.89 to 0.78) | -0.11 (-5.4 to 8.25) | 0.1 (0.03 to 0.17) | 0.28 (-0.11 to 0.68) | -2.07 (-3.39 to -0.53) | -0.05 (-0.21 to 0.09) | 0.16 (-0.45 to 0.76) | -2.31 (-5.22 to 0.59) |
| **Taiwan (Province of China)** | 1.29 (1.23 to 1.35) | 1.41 (1.23 to 1.58) | -0.35 (-0.64 to -0.14) | 1.23 (1.13 to 1.31) | 1.55 (1.35 to 1.76) | -1.56 (-3.05 to -0.79) | 1.24 (1.18 to 1.3) | 1.44 (1.28 to 1.6) | -0.56 (-0.86 to -0.26) |
| **Tajikistan** | -1.95 (-2.52 to -1.45) | -0.92 (-1.31 to -0.52) | -10.92 (-21.07 to -5.49) | -1.13 (-1.28 to -1.03) | -0.67 (-0.91 to -0.43) | -7.05 (-9.28 to -4.44) | -1.55 (-1.88 to -1.24) | -0.77 (-1.16 to -0.37) | -8.49 (-14.1 to -4.92) |
| **Thailand** | 0.16 (0.09 to 0.24) | 0.34 (0.21 to 0.46) | -2.8 (-4.1 to -1.29) | 0.24 (0.18 to 0.3) | 0.36 (0.21 to 0.51) | -1.84 (-2.84 to -0.64) | 0.19 (0.11 to 0.25) | 0.36 (0.2 to 0.53) | -2.22 (-3.57 to -0.3) |
| **Timor-Leste** | 0.56 (-0.22 to 1.68) | 1.1 (0.86 to 1.34) | -6.58 (-16.96 to 1.46) | 0.9 (0.77 to 0.99) | 1.06 (0.7 to 1.43) | -0.3 (-1.24 to 0.31) | 0.74 (0.26 to 1.48) | 1.16 (0.99 to 1.32) | -5.05 (-11.91 to 1.06) |
| **Togo** | 0.53 (0.48 to 0.57) | 1.24 (1.02 to 1.46) | -9.24 (-10.01 to -8.67) | 0.79 (0.74 to 0.84) | 1.08 (0.88 to 1.29) | -3.71 (-4.44 to -3.07) | 0.63 (0.58 to 0.66) | 1.12 (0.94 to 1.3) | -6.34 (-7.11 to -5.71) |
| **Tokelau** | 0.54 (0.5 to 0.58) | 0.72 (0.69 to 0.75) | -1.26 (-2.25 to -0.67) | 0.28 (0.24 to 0.32) | 0.46 (0.44 to 0.48) | -1.71 (-2.74 to -1.02) | 0.37 (0.33 to 0.41) | 0.56 (0.55 to 0.58) | -1.65 (-2.58 to -1.01) |
| **Tonga** | 0.41 (0.25 to 0.55) | 0.44 (0.3 to 0.57) | 0.54 (0 to 2.25) | 0.25 (0.2 to 0.29) | 0.23 (0.16 to 0.3) | 0.19 (0.04 to 0.75) | 0.29 (0.21 to 0.38) | 0.24 (0.06 to 0.42) | 0.33 (0.04 to 1.28) |
| **Trinidad and Tobago** | -0.25 (-0.41 to -0.07) | 0.75 (0.58 to 0.92) | -14.31 (-16.68 to -11.17) | -0.17 (-0.25 to -0.09) | 0.33 (0.05 to 0.61) | -7.37 (-8.47 to -5.89) | -0.2 (-0.35 to -0.09) | 0.56 (0.23 to 0.9) | -11.78 (-13.84 to -7.67) |
| **Tunisia** | -0.54 (-0.63 to -0.41) | 0.4 (0.31 to 0.5) | -13.63 (-15.03 to -11.82) | -0.2 (-0.25 to -0.13) | 0.38 (0.32 to 0.45) | -8.68 (-9.42 to -7.69) | -0.43 (-0.49 to -0.32) | 0.36 (0.28 to 0.45) | -11.15 (-12.22 to -9.72) |
| **Turkey** | -0.43 (-0.56 to -0.34) | 0.26 (0.14 to 0.38) | -9.52 (-11.38 to -6.41) | 0.03 (-0.05 to 0.11) | 0.45 (0.07 to 0.83) | -5.75 (-7.03 to -3.86) | -0.32 (-0.42 to -0.25) | 0.22 (0.1 to 0.33) | -7.57 (-9.02 to -5.19) |
| **Turkmenistan** | -1.78 (-1.92 to -1.62) | -1.34 (-1.73 to -0.94) | -7.68 (-9.73 to -4.78) | -1.16 (-1.25 to -1.09) | -0.89 (-1.17 to -0.61) | -4.71 (-6.04 to -2.81) | -1.39 (-1.51 to -1.3) | -1.03 (-1.27 to -0.79) | -6.19 (-7.98 to -3.69) |
| **Tuvalu** | 1.06 (0.85 to 1.28) | 1.12 (0.92 to 1.31) | 0.61 (-0.39 to 1.43) | 0.79 (0.69 to 0.91) | 0.77 (0.58 to 0.96) | 0.43 (-0.13 to 0.98) | 0.88 (0.75 to 1.04) | 0.91 (0.76 to 1.06) | 0.44 (-0.12 to 0.93) |
| **Uganda** | 1.4 (1.29 to 1.52) | 2.74 (2.55 to 2.93) | -17.06 (-18.36 to -15.34) | 1.78 (1.62 to 1.93) | 2.58 (2.19 to 2.98) | -11.05 (-13.16 to -8.68) | 1.6 (1.48 to 1.71) | 2.62 (2.43 to 2.82) | -13.77 (-15.34 to -12.33) |
| **Ukraine** | -1.41 (-1.52 to -1.32) | -0.97 (-1.33 to -0.6) | -7.95 (-9.5 to -5.45) | -1.12 (-1.19 to -1.04) | -0.86 (-1.23 to -0.49) | -5.44 (-6.75 to -3.7) | -1.23 (-1.34 to -1.15) | -0.85 (-1.19 to -0.51) | -6.05 (-7.65 to -3.92) |
| **United Arab Emirates** | 0.06 (0.02 to 0.1) | 0.88 (0.64 to 1.13) | -10.87 (-11.43 to -10.37) | 0.58 (0.45 to 0.7) | 0.81 (0.42 to 1.2) | -2.19 (-3.94 to -1.01) | 0.27 (0.18 to 0.34) | 0.84 (0.52 to 1.16) | -7.5 (-8.91 to -5.92) |
| **United Kingdom** | -0.19 (-0.35 to -0.09) | 0.39 (0.31 to 0.46) | -7.84 (-10.24 to -4.99) | 0.02 (-0.1 to 0.09) | 0.44 (0.35 to 0.52) | -5.83 (-7.53 to -3.33) | -0.03 (-0.13 to 0.06) | 0.42 (0.34 to 0.5) | -4.57 (-7.05 to -3.05) |
| **United Republic of Tanzania** | -0.21 (-0.26 to -0.17) | 0.89 (0.73 to 1.05) | -14.68 (-15.31 to -14.14) | 0.41 (0.35 to 0.47) | 0.95 (0.78 to 1.11) | -7.23 (-8.22 to -6.3) | 0.11 (0.06 to 0.15) | 0.93 (0.78 to 1.09) | -11.15 (-11.79 to -10.62) |
| **United States of America** | -0.41 (-0.45 to -0.38) | -0.46 (-0.55 to -0.36) | -8.66 (-9.29 to -8.05) | -0.62 (-0.66 to -0.58) | -0.28 (-0.33 to -0.23) | -10.34 (-11 to -9.87) | -0.8 (-0.92 to -0.73) | -0.37 (-0.41 to -0.33) | -8.8 (-9.38 to -8.21) |
| **United States Virgin Islands** | -0.98 (-1.12 to -0.9) | 0.2 (0.01 to 0.39) | -7.23 (-9.41 to -5) | -0.62 (-0.7 to -0.57) | 0.09 (-0.04 to 0.23) | -3.67 (-5.17 to -2.76) | -0.39 (-0.43 to -0.36) | 0.22 (0.06 to 0.39) | -5.86 (-7.69 to -3.98) |
| **Uruguay** | 0.08 (0 to 0.19) | 0.44 (0.37 to 0.5) | -5.25 (-6.48 to -3.13) | 0.2 (0.13 to 0.26) | 0.46 (0.39 to 0.53) | -3.61 (-4.64 to -2.06) | 0.1 (0.04 to 0.18) | 0.4 (0.34 to 0.45) | -4.26 (-5.11 to -2.68) |
| **Uzbekistan** | -1.59 (-1.7 to -1.5) | -1.38 (-1.82 to -0.95) | -2.82 (-4.02 to -1.73) | -0.91 (-1.01 to -0.82) | -0.79 (-1.18 to -0.41) | -1.71 (-2.74 to -0.79) | -1.21 (-1.33 to -1.12) | -1.04 (-1.5 to -0.59) | -2.2 (-3.46 to -1.17) |
| **Vanuatu** | 0.15 (0 to 0.28) | 0.65 (0.5 to 0.8) | -7.1 (-9.28 to -3.89) | 0.42 (0.34 to 0.51) | 0.69 (0.45 to 0.92) | -3.46 (-4.64 to -1.6) | 0.29 (0.2 to 0.39) | 0.64 (0.45 to 0.83) | -5.04 (-6.2 to -2.99) |
| **Venezuela (Bolivarian Republic of)** | -1.13 (-1.43 to -0.94) | 0.09 (-0.12 to 0.3) | -16.86 (-21.17 to -10.77) | -0.58 (-1 to -0.38) | 0.09 (-0.06 to 0.24) | -9.15 (-14.99 to -2.56) | -0.84 (-1.18 to -0.53) | 0.06 (-0.19 to 0.3) | -12.47 (-17.46 to -7.35) |
| **Viet Nam** | 0.31 (0.28 to 0.36) | 0.43 (0.38 to 0.48) | -1.89 (-2.41 to -0.97) | 0.62 (0.59 to 0.66) | 0.75 (0.71 to 0.79) | -1.5 (-1.93 to -0.78) | 0.48 (0.44 to 0.52) | 0.57 (0.53 to 0.62) | -1.58 (-2.07 to -0.74) |
| **Yemen** | -0.18 (-0.29 to -0.09) | 0.55 (0.28 to 0.83) | -10.2 (-11.89 to -7.64) | 0.56 (0.51 to 0.61) | 0.95 (0.79 to 1.11) | -4.98 (-5.82 to -3.94) | 0.24 (0.16 to 0.3) | 0.81 (0.6 to 1.01) | -7.71 (-8.96 to -5.77) |
| **Zambia** | 0.26 (0.13 to 0.39) | 1.92 (1.68 to 2.16) | -21.25 (-22.83 to -19.51) | 0.49 (0.41 to 0.57) | 1.4 (1.22 to 1.58) | -12.41 (-13.5 to -11.25) | 0.36 (0.25 to 0.46) | 1.63 (1.48 to 1.78) | -16.97 (-18.33 to -15.54) |
| **Zimbabwe** | -1.84 (-1.99 to -1.68) | -0.15 (-0.55 to 0.25) | -24.52 (-26.49 to -22.26) | -0.35 (-0.49 to -0.22) | 0.59 (0.41 to 0.77) | -13.06 (-14.99 to -11) | -0.98 (-1.14 to -0.84) | 0.29 (-0.02 to 0.6) | -18.56 (-20.59 to -16.64) |

Table S24. Average annual percent change (AAPC) in lifetime risks of developing and dying from specific cancer by sex globally, 1990-2021, 1990-2019 and 2019-2021.

| **Cause** | **Male** | | | | | | **Female** | | | | | | **Both sexes** | | | | | |
| --- | --- | --- | --- | --- | --- | --- | --- | --- | --- | --- | --- | --- | --- | --- | --- | --- | --- | --- |
|  | **AAPC of lifetime incidence risk** | | | **AAPC of lifetime death risk** | | | **AAPC of lifetime incidence risk** | | | **AAPC of lifetime death risk** | | | **AAPC of lifetime incidence risk** | | | **AAPC of lifetime death risk** | | |
|  | **1990-**  **2021** | **1990-**  **2019** | **2019-**  **2021** | **1990-**  **2021** | **1990-**  **2019** | **2019-**  **2021** | **1990-**  **2021** | **1990-**  **2019** | **2019-**  **2021** | **1990-**  **2021** | **1990-**  **2019** | **2019-**  **2021** | **1990-**  **2021** | **1990-**  **2019** | **2019-**  **2021** | **1990-**  **2021** | **1990-**  **2019** | **2019-**  **2021** |
| **Bladder cancer** | 0.17 (0.13 to 0.21) | 0.77 (0.6 to 0.93) | -7.8 (-8.55 to -7.22) | -0.35 (-0.42 to -0.3) | 0.46 (0.34 to 0.57) | -10.96 (-12.04 to -10.27) | 0.03 (0 to 0.06) | 0.43 (0.26 to 0.6) | -5.48 (-5.97 to -5.09) | -0.29 (-0.34 to -0.25) | 0.2 (0.05 to 0.36) | -7.15 (-7.94 to -6.65) | 0.22 (0.19 to 0.25) | 0.72 (0.63 to 0.81) | -6.22 (-6.77 to -5.75) | -0.22 (-0.26 to -0.18) | 0.4 (0.28 to 0.52) | -8.73 (-9.4 to -8.3) |
| **Brain and central nervous system cancer** | 0.93 (0.89 to 0.96) | 1.36 (1.28 to 1.44) | -5.04 (-5.63 to -4.61) | 0.64 (0.61 to 0.66) | 1.08 (1.02 to 1.14) | -5.3 (-5.8 to -4.87) | 1.13 (1.11 to 1.16) | 1.43 (1.32 to 1.53) | -3.13 (-3.63 to -2.71) | 0.73 (0.7 to 0.75) | 1.03 (0.94 to 1.12) | -3.44 (-3.82 to -3.1) | 1.02 (0.98 to 1.05) | 1.39 (1.3 to 1.48) | -4.27 (-4.87 to -3.82) | 0.68 (0.65 to 0.7) | 1.05 (0.98 to 1.13) | -4.44 (-4.89 to -4.06) |
| **Breast cancer** | 2.22 (2.16 to 2.27) | 2.76 (2.4 to 3.13) | -5.28 (-6.24 to -4.39) | 0.98 (0.92 to 1.02) | 1.57 (1.42 to 1.73) | -7.2 (-8.06 to -6.45) | 0.7 (0.67 to 0.72) | 0.94 (0.9 to 0.99) | -2.61 (-3.04 to -2.3) | -0.03 (-0.05 to 0) | 0.29 (0.24 to 0.34) | -4.29 (-4.66 to -4.01) | 0.67 (0.64 to 0.7) | 1 (0.91 to 1.09) | -3.7 (-4.2 to -3.34) | -0.12 (-0.16 to -0.1) | 0.31 (0.25 to 0.36) | -5.8 (-6.34 to -5.43) |
| **Cervical cancer** | / | / | / | / |  | / | -0.39 (-0.41 to -0.37) | -0.27 (-0.35 to -0.2) | / | -0.91 (-0.94 to -0.89) | -0.71 (-0.77 to -0.66) | -3.73 (-4.19 to -3.37) | -0.42 (-0.44 to -0.4) | -0.25 (-0.34 to -0.16) | -2.9 (-3.36 to -2.48) | -0.96 (-0.99 to -0.94) | -0.69 (-0.77 to -0.61) | -4.78 (-5.35 to -4.33) |
| **Colon and rectum cancer** | 0.89 (0.84 to 0.92) | 1.41 (1.36 to 1.47) | -6.4 (-7.12 to -5.77) | -0.01 (-0.06 to 0.04) | 0.63 (0.57 to 0.68) | -8.49 (-9.21 to -8.01) | 0.27 (0.24 to 0.29) | 0.61 (0.56 to 0.66) | -4.49 (-5 to -4.16) | -0.42 (-0.46 to -0.4) | -0.01 (-0.08 to 0.06) | -6.14 (-6.67 to -5.79) | 0.61 (0.57 to 0.64) | 1.04 (0.98 to 1.09) | -5.53 (-6.12 to -5.12) | -0.19 (-0.23 to -0.16) | 0.31 (0.27 to 0.36) | -7.43 (-8.1 to -7) |
| **Esophageal cancer** | -0.31 (-0.36 to -0.27) | 0.05 (-0.06 to 0.16) | -5.19 (-5.97 to -4.5) | -0.54 (-0.59 to -0.49) | -0.14 (-0.3 to 0.02) | -5.87 (-6.68 to -5.14) | -0.89 (-0.93 to -0.87) | -0.68 (-0.76 to -0.6) | -3.85 (-4.38 to -3.41) | -1.14 (-1.18 to -1.11) | -0.89 (-1.02 to -0.75) | -4.64 (-5.25 to -4.13) | -0.45 (-0.48 to -0.41) | -0.17 (-0.27 to -0.07) | -4.21 (-4.8 to -3.7) | -0.67 (-0.72 to -0.63) | -0.37 (-0.52 to -0.21) | -4.87 (-5.62 to -4.25) |
| **Eye cancer** | 0.57 (0.53 to 0.61) | 1.17 (1.12 to 1.22) | -6.93 (-7.73 to -6.32) | -0.28 (-0.32 to -0.25) | 0.25 (0.12 to 0.38) | -7.3 (-7.87 to -6.86) | 0.66 (0.61 to 0.69) | 1.07 (0.99 to 1.14) | -5.12 (-5.78 to -4.56) | -0.1 (-0.13 to -0.07) | 0.26 (0.16 to 0.35) | -5.22 (-5.81 to -4.83) | 0.61 (0.57 to 0.64) | 1.12 (1.04 to 1.19) | -6.35 (-7 to -5.84) | -0.19 (-0.23 to -0.17) | 0.26 (0.23 to 0.28) | -6.23 (-6.78 to -5.86) |
| **Gallbladder and biliary tract cancer** | 0.68 (0.61 to 0.72) | 1.27 (1.13 to 1.42) | -7.73 (-8.67 to -6.98) | 0.11 (0.03 to 0.16) | 0.7 (0.47 to 0.93) | -8.63 (-9.73 to -7.87) | -0.26 (-0.31 to -0.23) | 0.1 (0 to 0.2) | -5.31 (-5.95 to -4.85) | -0.64 (-0.7 to -0.6) | -0.23 (-0.38 to -0.07) | -5.93 (-6.8 to -5.21) | 0.1 (0.06 to 0.14) | 0.57 (0.44 to 0.7) | -6.56 (-7.29 to -6.01) | -0.37 (-0.43 to -0.33) | 0.14 (-0.06 to 0.35) | -7.29 (-8.15 to -6.69) |
| **Hodgkin lymphoma** | -0.87 (-0.93 to -0.81) | -0.62 (-0.78 to -0.46) | -4.26 (-5.17 to -2.99) | -1.69 (-1.74 to -1.66) | -1.36 (-1.41 to -1.31) | -5.99 (-6.78 to -5.38) | -0.76 (-0.8 to -0.72) | -0.59 (-0.73 to -0.46) | / | -1.46 (-1.51 to -1.41) | -1.27 (-1.36 to -1.19) | -4.09 (-4.74 to -3.08) | -0.82 (-0.87 to -0.78) | -0.61 (-0.73 to -0.49) | -3.75 (-4.45 to -2.69) | -1.6 (-1.64 to -1.57) | -1.35 (-1.39 to -1.31) | -5.12 (-5.73 to -4.6) |
| **Kidney cancer** | 1.22 (1.19 to 1.25) | 1.73 (1.61 to 1.85) | -5.73 (-6.3 to -5.22) | 0.67 (0.63 to 0.71) | 1.32 (1.26 to 1.39) | -7.84 (-8.56 to -7.19) | 0.8 (0.77 to 0.84) | 1.17 (1.06 to 1.29) | -4.07 (-4.69 to -3.48) | 0.37 (0.34 to 0.4) | 0.83 (0.78 to 0.88) | -6 (-6.55 to -5.54) | 1.08 (1.05 to 1.11) | 1.51 (1.41 to 1.62) | -4.78 (-5.3 to -4.32) | 0.59 (0.55 to 0.62) | 1.14 (1.08 to 1.19) | -6.92 (-7.54 to -6.41) |
| **Larynx cancer** | -0.74 (-0.77 to -0.71) | -0.38 (-0.42 to -0.34) | -5.68 (-6.14 to -5.36) | -1.25 (-1.28 to -1.23) | -0.85 (-0.9 to -0.79) | -6.87 (-7.26 to -6.59) | -0.02 (-0.07 to 0.02) | 0.18 (0.11 to 0.26) | -3.2 (-3.85 to -1.99) | -0.52 (-0.57 to -0.48) | -0.26 (-0.36 to -0.17) | -4.18 (-4.85 to -3.6) | -0.59 (-0.62 to -0.57) | -0.31 (-0.35 to -0.27) | -4.5 (-4.88 to -4.18) | -1.08 (-1.1 to -1.05) | -0.76 (-0.82 to -0.7) | -5.46 (-5.81 to -5.19) |
| **Leukemia** | 0.06 (0.01 to 0.1) | 0.56 (0.42 to 0.69) | -7.15 (-7.89 to -6.58) | -0.31 (-0.36 to -0.27) | 0.28 (0.14 to 0.41) | -8.42 (-9.13 to -7.98) | -0.09 (-0.12 to -0.07) | 0.28 (0.21 to 0.34) | -5.17 (-5.68 to -4.77) | -0.44 (-0.46 to -0.42) | -0.05 (-0.12 to 0.02) | -5.59 (-6.03 to -5.24) | 0.01 (-0.03 to 0.04) | 0.43 (0.3 to 0.56) | -6.08 (-6.69 to -5.63) | -0.35 (-0.4 to -0.31) | 0.13 (0.04 to 0.22) | -7.17 (-7.8 to -6.76) |
| **Lip and oral cavity cancer** | 0.56 (0.52 to 0.59) | 0.95 (0.88 to 1.02) | -4.81 (-5.37 to -4.32) | 0.13 (0.1 to 0.16) | 0.57 (0.5 to 0.63) | -5.78 (-6.34 to -5.34) | 1.15 (1.11 to 1.19) | 1.51 (1.46 to 1.57) | -3.85 (-4.52 to -3.39) | 0.77 (0.74 to 0.8) | 1.2 (1.11 to 1.28) | -4.73 (-5.28 to -4.28) | 0.79 (0.76 to 0.81) | 1.13 (1.07 to 1.19) | -3.99 (-4.44 to -3.6) | 0.37 (0.35 to 0.39) | 0.77 (0.69 to 0.85) | -5.12 (-5.58 to -4.75) |
| **Liver cancer** | 0.72 (0.69 to 0.75) | 1.18 (1.07 to 1.3) | -5.39 (-6.02 to -4.83) | 0.46 (0.41 to 0.51) | 0.93 (0.76 to 1.11) | -6.01 (-6.83 to -5.23) | 0.92 (0.89 to 0.94) | 1.27 (1.19 to 1.35) | -3.88 (-4.36 to -3.46) | 0.77 (0.71 to 0.83) | 1.16 (0.96 to 1.37) | -4.71 (-5.68 to -3.45) | 0.8 (0.76 to 0.83) | 1.2 (1.11 to 1.29) | -4.72 (-5.32 to -4.2) | 0.59 (0.54 to 0.64) | 1 (0.83 to 1.18) | -5.13 (-5.97 to -4.25) |
| **Malignant neoplasm of bone and articular cartilage** | 0.98 (0.93 to 1.01) | 1.36 (1.25 to 1.46) | -4.41 (-5.12 to -3.83) | 0.56 (0.52 to 0.6) | 1.03 (0.88 to 1.17) | -5.73 (-6.36 to -5.26) | 0.79 (0.75 to 0.81) | 1.1 (0.92 to 1.27) | -3.53 (-4.04 to -3.1) | 0.49 (0.45 to 0.52) | 0.82 (0.66 to 0.99) | -4.21 (-4.8 to -3.72) | 0.9 (0.86 to 0.93) | 1.25 (1.14 to 1.37) | -3.95 (-4.54 to -3.47) | 0.56 (0.49 to 0.6) | 0.94 (0.8 to 1.08) | -4.65 (-5.53 to -3.64) |
| **Malignant skin melanoma** | 1.29 (1.22 to 1.35) | 1.92 (1.66 to 2.18) | -7.27 (-8.35 to -5.69) | 0.14 (0.07 to 0.19) | 0.86 (0.69 to 1.03) | -9.14 (-10.23 to -8.23) | 0.94 (0.87 to 1.01) | 1.29 (1.04 to 1.53) | -4.54 (-5.65 to -3.15) | 0.03 (-0.02 to 0.07) | 0.49 (0.29 to 0.69) | -6.21 (-6.91 to -5.61) | 1.14 (1.07 to 1.2) | 1.6 (1.33 to 1.88) | -5.78 (-6.86 to -4.42) | 0.11 (0.07 to 0.16) | 0.67 (0.47 to 0.87) | -7.55 (-8.32 to -6.92) |
| **Mesothelioma** | -0.07 (-0.12 to -0.02) | 0.56 (0.41 to 0.7) | -8.45 (-9.31 to -7.75) | -0.02 (-0.08 to 0.03) | 0.66 (0.51 to 0.81) | -9.04 (-9.92 to -8.31) | 0.16 (0.12 to 0.19) | 0.53 (0.38 to 0.68) | -4.87 (-5.51 to -4.3) | 0.15 (0.11 to 0.18) | 0.56 (0.42 to 0.7) | -5.25 (-5.85 to -4.78) | 0.08 (0.04 to 0.11) | 0.57 (0.47 to 0.66) | -6.51 (-7.14 to -5.98) | 0.12 (0.08 to 0.15) | 0.64 (0.54 to 0.74) | -6.96 (-7.58 to -6.45) |
| **Multiple myeloma** | 1.16 (1.11 to 1.21) | 1.83 (1.72 to 1.93) | -7.75 (-8.56 to -7.11) | 0.83 (0.76 to 0.89) | 1.53 (1.4 to 1.66) | -9.1 (-10.28 to -8.39) | 0.81 (0.77 to 0.84) | 1.2 (1.1 to 1.31) | -5.01 (-5.68 to -4.45) | 0.57 (0.54 to 0.6) | 1.02 (0.9 to 1.13) | -5.54 (-6.02 to -5.11) | 0.99 (0.95 to 1.03) | 1.52 (1.42 to 1.62) | -6.26 (-6.86 to -5.76) | 0.71 (0.67 to 0.74) | 1.28 (1.18 to 1.37) | -7 (-7.65 to -6.5) |
| **Nasopharynx cancer** | -0.38 (-0.42 to -0.34) | -0.27 (-0.44 to -0.09) | -2.01 (-2.67 to -1) | -1.32 (-1.37 to -1.28) | -1.13 (-1.34 to -0.91) | -3.84 (-4.55 to -2.51) | -1.1 (-1.14 to -1.07) | -1.04 (-1.12 to -0.97) | -1.89 (-2.47 to -1.08) | -1.77 (-1.82 to -1.72) | -1.67 (-1.76 to -1.57) | -3.31 (-3.98 to -2.37) | -0.57 (-0.61 to -0.53) | -0.48 (-0.56 to -0.39) | -1.71 (-2.31 to -0.78) | -1.42 (-1.47 to -1.38) | -1.27 (-1.37 to -1.17) | -3.48 (-4.13 to -2.67) |
| **Neuroblastoma and other peripheral nervous cell tumors** | 1.78 (1.74 to 1.81) | 2.16 (2.08 to 2.24) | -3.66 (-4.32 to -3.11) | 1.71 (1.67 to 1.74) | 2.12 (2.07 to 2.17) | -3.92 (-4.53 to -3.44) | 1.35 (1.3 to 1.39) | 1.73 (1.65 to 1.82) | -3.54 (-4.25 to -2.97) | 1.3 (1.26 to 1.33) | 1.64 (1.57 to 1.71) | -3.11 (-3.62 to -2.69) | 1.58 (1.53 to 1.62) | 1.96 (1.92 to 2) | -3.75 (-4.44 to -3.31) | 1.52 (1.48 to 1.56) | 1.91 (1.87 to 1.94) | -3.77 (-4.4 to -3.38) |
| **Non-Hodgkin lymphoma** | 1.15 (1.1 to 1.2) | 1.71 (1.61 to 1.81) | -6.42 (-7.16 to -5.86) | 0.24 (0.19 to 0.27) | 0.85 (0.79 to 0.91) | -8.1 (-8.87 to -7.51) | 1.12 (1.08 to 1.15) | 1.46 (1.34 to 1.59) | -3.66 (-4.22 to -3.22) | 0.39 (0.36 to 0.42) | 0.8 (0.69 to 0.91) | -5.09 (-5.6 to -4.67) | 1.16 (1.12 to 1.2) | 1.6 (1.48 to 1.72) | -5.13 (-5.74 to -4.65) | 0.31 (0.28 to 0.34) | 0.82 (0.76 to 0.87) | -6.63 (-7.2 to -6.17) |
| **Other malignant neoplasms** | 0.66 (0.61 to 0.7) | 1.15 (0.93 to 1.36) | -5.99 (-6.77 to -5.34) | -0.25 (-0.3 to -0.21) | 0.29 (0.19 to 0.39) | -7.65 (-8.37 to -7.02) | 0.81 (0.77 to 0.84) | 1.2 (1.11 to 1.29) | -4.53 (-5.13 to -4.03) | -0.12 (-0.15 to -0.09) | 0.31 (0.23 to 0.39) | -6.02 (-6.59 to -5.61) | 0.74 (0.69 to 0.78) | 1.18 (1.1 to 1.27) | -5.21 (-5.99 to -4.58) | -0.18 (-0.22 to -0.15) | 0.29 (0.22 to 0.36) | -6.71 (-7.33 to -6.17) |
| **Other pharynx cancer** | 0.96 (0.9 to 1.01) | 1.36 (1.25 to 1.47) | -4.57 (-5.44 to -3.9) | 0.43 (0.39 to 0.46) | 0.86 (0.76 to 0.95) | -5.5 (-6.12 to -5.05) | 1.15 (1.08 to 1.21) | 1.48 (1.36 to 1.6) | -3.46 (-4.47 to -1.66) | 0.51 (0.46 to 0.56) | 0.86 (0.71 to 1.02) | -4.2 (-4.98 to -3.5) | 1.03 (0.98 to 1.07) | 1.37 (1.26 to 1.48) | -4.16 (-4.96 to -3.58) | 0.49 (0.45 to 0.52) | 0.85 (0.76 to 0.94) | -4.6 (-5.21 to -4.17) |
| **Ovarian cancer** | / | / | / | / | / | / | -0.02 (-0.05 to 0) | 0.15 (0.09 to 0.21) | -2.76 (-3.23 to -2.34) | -0.1 (-0.13 to -0.06) | 0.15 (0.09 to 0.22) | -3.65 (-4.15 to -3.18) | -0.09 (-0.14 to -0.05) | 0.19 (0.1 to 0.28) | -3.68 (-4.43 to -2.42) | -0.21 (-0.26 to -0.18) | 0.17 (0.01 to 0.33) | -5.09 (-5.81 to -4.38) |
| **Pancreatic cancer** | 0.74 (0.69 to 0.78) | 1.32 (1.27 to 1.38) | -7.21 (-7.92 to -6.68) | 0.65 (0.61 to 0.69) | 1.25 (1.19 to 1.32) | -7.54 (-8.24 to -7.01) | 0.84 (0.8 to 0.86) | 1.29 (1.24 to 1.34) | -5.42 (-5.95 to -5.02) | 0.73 (0.69 to 0.76) | 1.19 (1.13 to 1.25) | -5.61 (-6.19 to -5.17) | 0.78 (0.73 to 0.81) | 1.3 (1.21 to 1.39) | -6.43 (-7.08 to -5.97) | 0.68 (0.63 to 0.71) | 1.21 (1.16 to 1.27) | -6.68 (-7.36 to -6.22) |
| **Prostate cancer** | 0.58 (0.53 to 0.62) | 1.18 (1.05 to 1.32) | -7.56 (-8.33 to -7.01) | -0.27 (-0.41 to -0.19) | 0.65 (0.47 to 0.84) | -11.25 (-13.22 to -7.44) | / | / | / | / | / | / | 0.76 (0.72 to 0.79) | 1.23 (1.07 to 1.4) | -5.81 (-6.4 to -5.33) | 0.04 (-0.01 to 0.08) | 0.7 (0.56 to 0.85) | -9.03 (-9.75 to -8.38) |
| **Soft tissue and other extraosseous sarcomas** | 0.62 (0.57 to 0.66) | 1.2 (1.06 to 1.34) | -7.26 (-8.05 to -6.68) | 0.09 (0.03 to 0.13) | 0.68 (0.56 to 0.8) | -7.84 (-8.73 to -7.16) | 0.4 (0.33 to 0.45) | 0.77 (0.52 to 1.02) | -5.26 (-6.23 to -4.44) | -0.08 (-0.16 to -0.03) | 0.29 (0.06 to 0.51) | -5.55 (-6.73 to -4.42) | 0.52 (0.47 to 0.57) | 0.99 (0.8 to 1.17) | -6.19 (-7.03 to -5.57) | 0.02 (-0.04 to 0.06) | 0.5 (0.39 to 0.62) | -6.62 (-7.48 to -5.99) |
| **Stomach cancer** | -1.18 (-1.23 to -1.14) | -0.78 (-0.98 to -0.58) | -6.81 (-7.52 to -6.17) | -1.62 (-1.67 to -1.57) | -1.15 (-1.33 to -0.98) | -7.91 (-8.65 to -7.35) | -1.51 (-1.54 to -1.49) | -1.25 (-1.31 to -1.18) | -5.14 (-5.68 to -4.67) | -1.74 (-1.78 to -1.72) | -1.45 (-1.53 to -1.36) | -5.83 (-6.42 to -5.37) | -1.27 (-1.33 to -1.23) | -0.94 (-1.08 to -0.79) | -5.78 (-6.54 to -4.94) | -1.63 (-1.67 to -1.59) | -1.25 (-1.36 to -1.15) | -6.52 (-7.12 to -6.06) |
| **Testicular cancer** | 1.37 (1.29 to 1.43) | 1.57 (1.37 to 1.78) | -1.64 (-2.65 to -0.15) | -0.31 (-0.35 to -0.28) | 0.04 (-0.07 to 0.15) | -5.13 (-5.71 to -4.71) | / | / | / | / | / | / | 1.36 (1.29 to 1.43) | 1.56 (1.37 to 1.76) | -1.36 (-2.34 to 0.12) | -0.23 (-0.26 to -0.21) | 0.04 (-0.07 to 0.15) | -4.06 (-4.47 to -3.72) |
| **Thyroid cancer** | 1.82 (1.71 to 1.89) | 2.22 (1.97 to 2.48) | -3.73 (-5.25 to -1.69) | 0.79 (0.72 to 0.84) | 1.4 (1.28 to 1.53) | -7.55 (-8.63 to -6.6) | 1.08 (1.04 to 1.11) | 1.28 (1.16 to 1.4) | / | 0.02 (-0.02 to 0.05) | 0.38 (0.31 to 0.45) | -5.03 (-5.6 to -4.58) | 1.27 (1.2 to 1.31) | 1.56 (1.44 to 1.68) | -2.77 (-3.7 to -1.52) | 0.24 (0.2 to 0.28) | 0.73 (0.67 to 0.8) | -6.44 (-7.12 to -5.87) |
| **Tracheal bronchus and lung cancer** | -0.23 (-0.27 to -0.19) | 0.26 (0.2 to 0.31) | -6.89 (-7.55 to -6.4) | -0.45 (-0.5 to -0.42) | 0.06 (0 to 0.12) | -7.47 (-8.14 to -6.93) | 1.15 (1.11 to 1.18) | 1.48 (1.37 to 1.58) | -3.13 (-3.76 to -2.48) | 0.91 (0.88 to 0.94) | 1.25 (1.2 to 1.29) | -3.6 (-4.05 to -3.14) | 0.25 (0.22 to 0.28) | 0.63 (0.58 to 0.69) | -5.04 (-5.51 to -4.67) | 0.02 (-0.02 to 0.04) | 0.43 (0.38 to 0.47) | -5.66 (-6.2 to -5.24) |
| **Uterine cancer** | / | / | / | / | / | / | 0.74 (0.7 to 0.77) | 1.03 (0.92 to 1.13) | -2.92 (-3.57 to -2.26) | -0.17 (-0.21 to -0.14) | 0.15 (0.08 to 0.22) | -4.71 (-5.39 to -4.2) | 0.69 (0.64 to 0.72) | 1.02 (0.92 to 1.13) | -4.14 (-4.83 to -3.63) | -0.28 (-0.33 to -0.25) | 0.14 (0.06 to 0.23) | -6.38 (-7.08 to -5.81) |

Figure S1. Lifetime risk of developing and dying from cancer at the national level in 2021 for males. (A) Lifetime risk of developing cancer. (B) Lifetime risk of dying from cancer.

Figure S2. Lifetime risk of developing and dying from cancer at the national level in 2021 for females**.** (A) Lifetime risk of developing cancer. (B) Lifetime risk of dying from cancer.

Figure S3. Global lifetime risks of developing and dying from cancer for males by age at diagnosis in 2021**.** (A) Probability of developing cancer. (B) Probability of dying from cancer.

Note: The curves represent the probabilities of developing and dying from cancer at different baseline ages (at birth, 40, 50, 60, 70, 80, and 90 years) of cancer-free life. The risks through death represent lifetime risk from each baseline age.

Figure S4. Global lifetime risks of developing and dying from cancer for females by age at diagnosis in 2021**.** (A) Probability of developing cancer. (B) Probability of dying from cancer.

Note: The curves represent the probabilities of developing and dying from cancer at different baseline ages (at birth, 40, 50, 60, 70, 80, and 90 years) of cancer-free life. The risks through death represent lifetime risk from each baseline age.
Figure S5. Correlation between lifetime risk of developing and dying from cancer at national and regional SDI for males**.** (A) Association between lifetime incidence risk and national SDI in 2021. (B) Association between lifetime death risk and national SDI in 2021. (C) Association between lifetime incidence risk and regional SDI from 1990 to 2021. (D) Association between lifetime death risk and regional SDI from 1990 to 2021. The curve represents a quadratic regression fit. 32 points in (C) and (D) are plotted for each GBD region and showed observed lifetime risk from 1990 to 2021 for that region. Spearman's rank correlation coefficients (ρ) and corresponding P-values are shown in the upper left of each panel.
Figure S6. Correlation between lifetime risk of developing and dying from cancer at national and regional SDI for females**.** (A) Association between lifetime incidence risk and national SDI in 2021. (B) Association between lifetime death risk and national SDI in 2021. (C) Association between lifetime incidence risk and regional SDI from 1990 to 2021. (D) Association between lifetime death risk and regional SDI from 1990 to 2021. The curve represents a quadratic regression fit. 32 points in (C) and (D) are plotted for each GBD region and showed observed lifetime risk from 1990 to 2021 for that region. Spearman's rank correlation coefficients (ρ) and corresponding P-values are shown in the upper left of each panel.

**Figure S7. Lifetime death risks of different cancer types attributable to risk factors globally and by GBD region in 2021.** Note: Blank cells in the heatmap denote risk below 0.01, not zero.

**Figure S8. Correlation between the lifetime risks of developing or dying from cancer and the cumulative risks during 0-74 years.** (A) Correlation between the lifetime risks of developing cancer and the cumulative risks. (B) Correlation between the lifetime risks of dying from cancer and the cumulative risks. Note: The red line indicates the fitted risks using linear regression with a shaded area showing a 95% confidence interval; the short dashed black line marks the reference line, where the risk estimates of two measures are equal; the colored circles show the risks in different countries/regions with different life expectancies.

Figure S9. The Distribution of lifetime incidence and death risk for the 10 Most Common Cancers in 2021 across SDI quintiles for males. (A)-(E) show the distribution of lifetime incidence risk across SDI quintiles, ranging from high to low SDI quintile. (F)-(J) show the distribution of lifetime death risk across SDI quintiles ranging from high to low SDI quintile.

Figure S10. The Distribution of lifetime incidence and death43 33 risk for the 10 Most Common Cancers in 2021 across SDI quintiles for females. (A)-(E) show the distribution of lifetime incidence risk across SDI quintiles, ranging from high to low SDI quintile. (F)-(J) show the distribution of lifetime death risk across SDI quintiles ranging from high to low SDI quintile.

Figure S11. Average annual percent changes (AAPCs) in lifetime risk of developing and dying from cancer at the national level for males, 1990-2021, 1990-2019 and 2019-2021**.** (A) AAPCs in lifetime incidence risk, 1990-2021. (B) AAPCs in lifetime death risk, 1990-2021. (C) AAPCs in lifetime incidence risk, 1990-2019. (D) AAPCs in lifetime death risk, 1990-2019. (E) AAPCs in lifetime incidence risk, 2019-2021. (F) AAPCs in lifetime death risk, 2019-2021.

Figure S12. Average annual percent change (AAPC) in lifetime risk of developing and dying from cancer at the national level for females, 1990-2021, 1990-2019 and 2019-2021**.** (A) AAPCs in lifetime incidence risk, 1990-2021. (B) AAPCs in lifetime death risk, 1990-2021. (C) AAPCs in lifetime incidence risk, 1990-2019. (D) AAPCs in lifetime death risk, 1990-2019. (E) AAPCs in lifetime incidence risk, 2019-2021. (F) AAPCs in lifetime death risk, 2019-2021.

Figure S13. Average annual percent changes (AAPCs) in lifetime risks of developing and dying from cancer for males, 1990-2021. (A) Lifetime incidence risk by cancer type. (B) Lifetime death risk by cancer type. (C) Lifetime incidence risk by GBD region. (D) Lifetime death risk by GBD region. (E) Lifetime incidence risk by SDI quintile. (F) Lifetime death risk by SDI quintile.

Note: In the figure, CNS refers to Brain and central nervous system cancer, GBTC refers to Gallbladder and biliary tract cancer, MNBAC refers to Malignant neoplasm of bone and articular cartilage, NB & other PNETs refers to Neuroblastoma and other peripheral nervous cell tumors, STS refers to Soft tissue and other extraosseous sarcomas and TBL refers to Tracheal bronchus and lung cancer.

Figure S14. Average annual percent changes (AAPCs) in lifetime risks of developing and dying from cancer for females, 1990-2021. (A) Lifetime incidence risk by cancer type. (B) Lifetime death risk by cancer type. (C) Lifetime incidence risk by GBD region. (D) Lifetime death risk by GBD region. (E) Lifetime incidence risk by SDI quintile. (F) Lifetime death risk by SDI quintile.

Note: In the figure, CNS refers to Brain and central nervous system cancer, GBTC refers to Gallbladder and biliary tract cancer, MNBAC refers to Malignant neoplasm of bone and articular cartilage, NB & other PNETs refers to Neuroblastoma and other peripheral nervous cell tumors, STS refers to Soft tissue and other extraosseous sarcomas and TBL refers to Tracheal bronchus and lung cancer.

Figure S15. Joinpoint regression analysis of the lifetime incidence and death risk globally by sex and for both sexes by SDI quintile. (A) Global Lifetime incidence risk by sex. (B) Global lifetime death risk by sex. (C) Lifetime incidence risk for both sexes by SDI quintile. (D) Lifetime death risk of both sexes by SDI quintile

Figure S16. Joinpoint regression analysis of the lifetime incidence and death risk of cancer for males by SDI quintile, 1990-2021. (A) Lifetime incidence risk. (B) Lifetime death risk。

Figure S17. Joinpoint regression analysis of the lifetime incidence and death risk of cancer for females by SDI quintile, 1990-2021. (A) Lifetime incidence risk. (B) Lifetime death risk.

Figure S18. Joinpoint regression analysis of the lifetime incidence and death risk for selected cancer types globally among males, 1990-2021. (A) Lifetime incidence risk. (B) Lifetime death risk.

Note: Select five cancers with the highest incidence risk and death risk in 2021, respectively

Figure S19. Joinpoint regression analysis of the lifetime incidence and death risk for selected cancer types globally among females, 1990-2021. (A) Lifetime incidence risk. (B) Lifetime death risk.

Note: Select five cancers with the highest incidence risk and death risk in 2021, respectively

1.	Sasieni PD, Shelton J, Ormiston-Smith N, Thomson CS, Silcocks PB. What is the lifetime risk of developing cancer?: the effect of adjusting for multiple primaries. Br J Cancer. Jul 26 2011;105(3):460-5. doi:10.1038/bjc.2011.250

1.	Sasieni PD, Shelton J, Ormiston-Smith N, Thomson CS, Silcocks PB. What is the lifetime risk of developing cancer?: the effect of adjusting for multiple primaries. Br J Cancer. Jul 26 2011;105(3):460-5. doi:10.1038/bjc.2011.250

2.	Ahmad AS, Ormiston-Smith N, Sasieni PD. Trends in the lifetime risk of developing cancer in Great Britain: comparison of risk for those born from 1930 to 1960. Br J Cancer. Mar 3 2015;112(5):943-7. doi:10.1038/bjc.2014.606

1.	Sasieni PD, Shelton J, Ormiston-Smith N, Thomson CS, Silcocks PB. What is the lifetime risk of developing cancer?: the effect of adjusting for multiple primaries. Br J Cancer. Jul 26 2011;105(3):460-5. doi:10.1038/bjc.2011.250

2.	Ahmad AS, Ormiston-Smith N, Sasieni PD. Trends in the lifetime risk of developing cancer in Great Britain: comparison of risk for those born from 1930 to 1960. Br J Cancer. Mar 3 2015;112(5):943-7. doi:10.1038/bjc.2014.606

3.	Zheng R, Wang S, Zhang S, et al. Global, regional, and national lifetime probabilities of developing cancer in 2020. Sci Bull (Beijing). Nov 15 2023;68(21):2620-2628. doi:10.1016/j.scib.2023.09.041
